# Integrating common and rare variants improves polygenic risk prediction across diverse populations

**DOI:** 10.1038/s41467-026-72185-2

**Published:** 2026-04-24

**Authors:** Jacob Williams, Tony Chen, Xing Hua, Wendy Wong, Kai Yu, Peter Kraft, Xihao Li, Haoyu Zhang

**Affiliations:** 1 Division of Cancer Epidemiology and Genetics, National Cancer Institute, Bethesda, MD USA; 2 Department of Biostatistics, Harvard T.H. Chan School of Public Health, Boston, MA USA; 3 Cancer Genomics Research Laboratory, Frederick National Laboratory for Cancer Research, Leidos Biomedical Research Inc, Rockville, MD USA; 4 Department of Biostatistics, University of North Carolina at Chapel Hill, Chapel Hill, NC USA; 5 Department of Genetics, University of North Carolina at Chapel Hill, Chapel Hill, NC USA

**Keywords:** Statistical methods, Computational models, Genome-wide association studies, Quantitative trait

## Abstract

PRSs predict complex traits by aggregating genetic effects across the genome, yet most models focus on common variants, overlooking rare variants that may contribute to hidden heritability. Here, we develop RICE, a PRS framework integrating both common and rare variants to improve genetic risk prediction across diverse ancestries. RICE constructs separate PRSs: for common variants, it integrates methods using ensemble learning; for rare variants, it uses gene-level testing with functional annotations and penalized regression. We evaluate RICE using simulated datasets and sequencing data from UK Biobank and All of Us, involving up to 740 million genetic variants from 361,939 individuals across diverse ancestries and 11 complex traits. In real data analysis, RICE improves predictive accuracy compared to leading common variant methods for traits with distinct rare variant architectures, particularly lipids and height. For lipid traits, incorporating rare variants increased R^2^ by up to ~11.2% in Europeans and ~60.7% in African ancestry compared to common variant PRS alone. Notably, for lipid traits, RICE captures substantial predictive signal beyond established high-penetrance genes, validating its ability to leverage the broader polygenic architecture of rare variation.

## Abstract (web summary)

Most PRS models focus on common variants, overlooking rare variant contributions. Here, the authors present RICE, a PRS framework that integrates common and rare genetic variants to strengthen polygenic risk prediction across ancestries.

## Introduction

Polygenic risk scores (PRSs) aggregate the effect sizes of numerous genetic variants across the genome to predict an individual’s risk of developing complex traits and diseases^1–6^. PRSs have shown promise in clinical applications, enabling personalized risk prediction and informing prevention strategies for conditions such as cardiovascular diseases, type 2 diabetes, and breast cancer^7–10^. By identifying individuals at higher genetic risk, PRSs can facilitate early interventions and improve patient outcomes.

Existing PRS methods focus primarily on common genetic variants, which are well-represented in large genome-wide association studies (GWAS). Due to their higher allele frequencies, common variants provide robust effect size estimates from large-scale GWAS, supporting the development of reliable risk prediction models. Advances in genotyping technologies, imputation methods, and the availability of extensive GWAS data have contributed to the success of common variant-based PRS models. To further refine risk prediction, methods accounting for linkage disequilibrium (LD) between variants, such as clumping and thresholding^11–13^, penalization-based models^10,14–16^, and Bayesian approaches^17–21^, have been developed. Recent approaches that jointly model data across multiple ancestries have improved performance in diverse cohorts^22–26^.

However, rare variants (minor allele frequency [MAF] <1%) are often excluded from PRSs due to low statistical power in single-variant association testing, historical limitations in large-scale sequencing data, limited integration methods, and challenges in accurately estimating their effect sizes. While some rare variants have larger, well-documented effect sizes (e.g., mutations in *BRCA1/2*^27^, *APOB*^28^, *PCSK9* ^28,29^ or *BSN*^30^), many others tend to have modest effects that collectively contribute to disease risk^31–35^. Excluding these variants may underestimate risk, as the cumulative effect of multiple modest-effect rare variants can be significant^36,37^. Incorporating rare variants into PRSs can enhance risk prediction and provide a more comprehensive understanding of genetic risk, especially for complex traits influenced by numerous genetic factors.

Advancements in whole-genome sequencing (WGS) and whole-exome sequencing (WES) biobanks, such as UK Biobank (UKB) and All of Us Research Program (AoU), have greatly expanded opportunities for evaluating rare variants in both coding and noncoding regions^32,38–41^. To address analytical challenges posed by rare variants, methods such as the burden test, sequence kernel association test (SKAT), and variant-Set Test for Association using Annotation infoRmation (STAAR)^42–47^ have been developed to analyze groups of rare variants collectively. Approaches like STAARpipeline leverage functional annotations to define variant sets for both coding and noncoding rare variants^46,48–51^, improving statistical power and interpretability of identified genes. Despite these advancements in association testing for rare variants, methods that simultaneously integrate rare variants into PRSs and support multi-ancestry data are notably limited. In parallel, several studies have explored the use of rare variant burden scores for risk prediction. For example, Lali et al. developed RV-EXCALIBER to integrate rare variant burden into cardiovascular disease prediction^52^, while Chan et al. introduced the Genome-wide Rare Variant Score for autism spectrum disorders^53^. These approaches highlight the potential of rare variant burden scores in disease risk stratification, but they have not been broadly extended to integrate rare and common variants within a unified, multi-ancestry framework, nor have they been systematically evaluated across multiple large-scale biobanks and a wide range of complex traits.

To address these limitations, we present RICE (polygenic Risk predictions Integrating Common and rarE variants), a framework designed for biobank sequencing data that efficiently integrates both common and rare variants. RICE combines ensemble learning techniques with advanced association testing methods to construct comprehensive PRSs that capture genetic risk from both common and rare variants. RICE significantly enhances PRS, providing a more accurate and inclusive approach to genetic risk prediction.

We evaluated RICE using extensive simulated datasets and large-scale WES, Imputed, and WGS data from UKB and AoU, with up to 740 million genetic variants from 361,939 unrelated individuals across African (AFR), Admixed American or Latino (AMR), European (EUR), Middle Eastern (MID) and South Asian (SAS) ancestries. Our analyses spanned 11 complex traits, including height, body mass index (BMI), breast cancer, and type 2 diabetes (T2D). Simulation studies demonstrated that RICE effectively models rare variant signals, achieving substantial improvements in PRS accuracy across all ancestries compared to leading common variant methods. In real data analyses, RICE improved predictive accuracy compared to top existing common variant PRS methods for traits with distinct rare variant architectures, particularly lipid traits and height, demonstrating the utility of a comprehensive common and rare variant PRS framework.

## Results

### Method overview

**Fig. 1:**
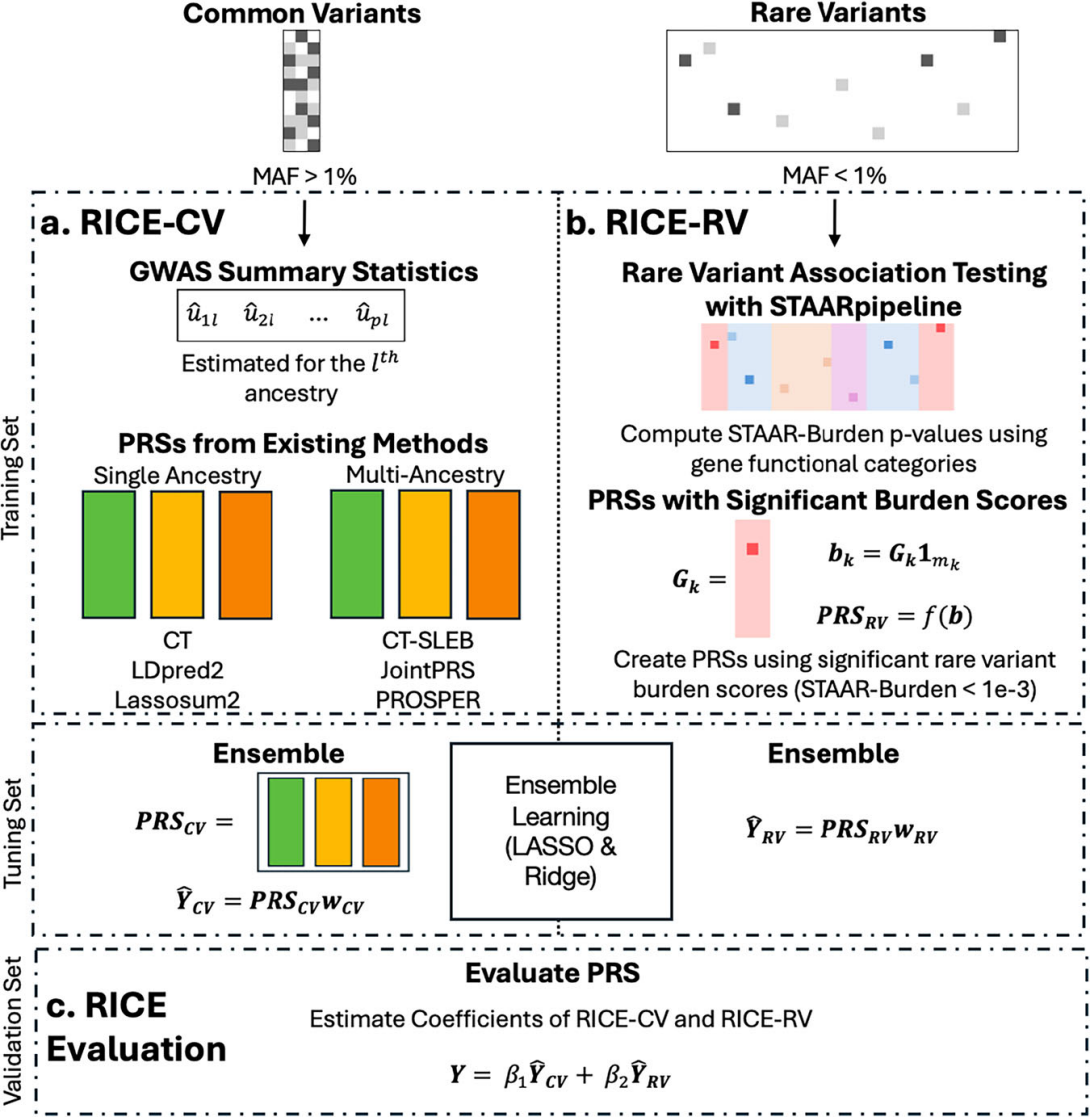
Overview of the RICE framework for polygenic risk prediction. RICE integrates common and rare variants in three steps: **a** RICE-CV (common variant PRS) is generated by combining PRSs from multiple existing methods, such as clumping and thresholding (CT), LDpred2, and Lassosum2 for single-ancestry data, or CT-SLEB, JointPRS, and PROSPER for multi-ancestry data. These PRSs are then combined using ensemble learning (LASSO and ridge regression) to produce a single robust common variant PRS. **b** RICE-RV (rare variant PRS) identifies significant rare variant sets using STAARpipeline ( $p < 1\times {10}^{-3}$) conditioned on the RICE-CV PRS. These significant sets are collapsed into burden scores and modeled using LASSO and ridge regression. The resulting PRSs are combined through ensemble learning (LASSO and ridge regression) to create a single rare variant PRS. **c** Final evaluation: The pre-trained RICE-CV and RICE-RV models (with combination weights fixed from the tuning step) are applied to the validation set to evaluate overall predictive performance (${R}^{2}$ or AUC). Additionally, a regression model is used solely to estimate individual standardized effect sizes (Beta per SD) of the components, adjusting for covariates such as age, sex, and principal components. The data are divided into three independent sets: a training set used to derive genome-wide association study (GWAS) summary statistics and perform rare variant association testing; a tuning set used to optimize model parameters and train the ensemble models; and a validation set used to evaluate the final predictive performance of the integrated PRS.

RICE predicts risk of complex traits and diseases by incorporating common (RICE-CV) and rare variants (RICE-RV). The framework has three steps (Fig. 1): (1) RICE-CV combines multiple PRSs for common variants using ensemble learning to generate a single PRS; (2) RICE-RV identifies significant rare variant sets conditioned on RICE-CV, creates PRSs based on burden scores, and combines them via ensemble learning; (3) RICE-CV and RICE-RV PRSs are jointly evaluated in a regression model with covariates. RICE requires three independent datasets: (1) training for generating GWAS summary statistics, rare variant *p* values, and PRS models; (2) tuning for optimizing parameters; (3) validation for assessing prediction performance.

In the first step (Fig. 1a), RICE-CV uses the training dataset to identify common variants (MAF > 0.01 in any ancestry group) and compute ancestry-specific GWAS summary statistics. Using these summary statistics, existing PRS methods are used to generate multiple PRSs. To leverage the advantages of different methodological approaches, we select a representative method from each category: clumping-based approach, penalization-based approach, and Bayesian-based approach. For single-ancestry data (e.g., primarily EUR populations), PRSs are obtained from clumping and thresholding (CT)^11–13^, LDpred2^54^, and Lassosum2^15^. For multi-ancestry data, PRSs are obtained from CT-SLEB^22^, JointPRS^55^, and PROSPER^25^. After computing PRSs from different approaches under various tuning parameters, RICE-CV performs ensemble learning ^54^ to combine all PRSs into a single robust common variant PRS, using LASSO and ridge regression as base learners.

In the second step (Fig. 1b), RICE-RV uses the training set to fit a baseline model adjusting for covariates, including top 10 principal components (PCs), sex, age, age squared, and RICE-CV to prioritize rare variant signals independent of common variant effects. Using residuals from this baseline model, RICE-RV performs variant set analyses with STAARpipeline to identify significant rare variant sets^48^, which defines rare variant sets using functional categories within each protein-coding gene. In WES datasets, RICE-RV conducts gene-centric analysis with rare variants in coding region only. In WGS datasets, RICE-RV analyzes rare variants in both coding and noncoding regions. Significant rare variant sets are selected as those with a STAAR-Burden *p* value less than ${1\times 10}^{-3}$. These sets are then collapsed into burden scores, which approximately represent the combined allele frequencies (Methods). These burden scores are jointly modeled using LASSO and ridge regression under different tuning parameters. PRSs are then generated on the tuning dataset using these burden scores and weights estimated from different penalized regression models. Finally, the PRSs are combined through ensemble learning with LASSO and ridge regression as base learners, to create a single rare variant PRS.

To evaluate RICE, in the final step (Fig. 1c), RICE-CV and RICE-RV are jointly evaluated on the validation set using a linear or logistic regression model, adjusting for covariates including top 10 PCs, sex, age, and age squared. The estimated regression coefficients of RICE-CV and RICE-RV are reported separately as measures of predictive performance.

### Evaluation of prediction performance

PRSs are traditionally evaluated using metrics like the coefficient of determination (${R}^{2}$) for continuous traits or area under the receiver operating characteristic curve (AUC) for binary traits^2^. These measures assess how well the PRS explains variation in a trait on average across the entire population. However, because rare variants are present in only a relatively small subset of individuals, their impact may not significantly influence these average-based metrics. Despite this, rare variants can have substantial effects on individuals who carry them, which may be overlooked when using traditional evaluation methods.

To better capture the contribution of rare variants, we report an additional, complementary metric: the estimated regression coefficient of the standardized PRS on the standardized trait (Methods). For continuous traits, this coefficient represents the expected change in standardized phenotype per standard deviation (SD) increase in PRS. For binary traits, it corresponds to the log odds ratio (log OR) per SD increase in PRS, a widely used and interpretable measure in clinical genetics and genetic epidemiology. Throughout this manuscript, we refer to this as the standardized effect size of PRS, denoted as “Beta per SD of PRS” for continuous traits and “log OR per SD of PRS” for binary traits. As detailed in the Supplementary Note, this coefficient can also be expressed as the square root of the heritability explained by the PRS. We use this metric to quantify the effect sizes of both common and rare variant PRSs, particularly when rare variants may have limited influence on average-based metrics, offering an interpretable per-SD effect that complements standard PRS measures like *R*^2^ or AUC. RICE’s predictive performance was evaluated using three complementary metrics: (1) standardized effect size, for assessing statistical significance and relative improvements; (2) *R*^2^ (continuous traits) or AUC (binary traits), for absolute predictive accuracy; and (3) trait differences across PRS quantiles, to illustrate potential clinical relevance in the UKB and AoU datasets.

**Fig. 2:**
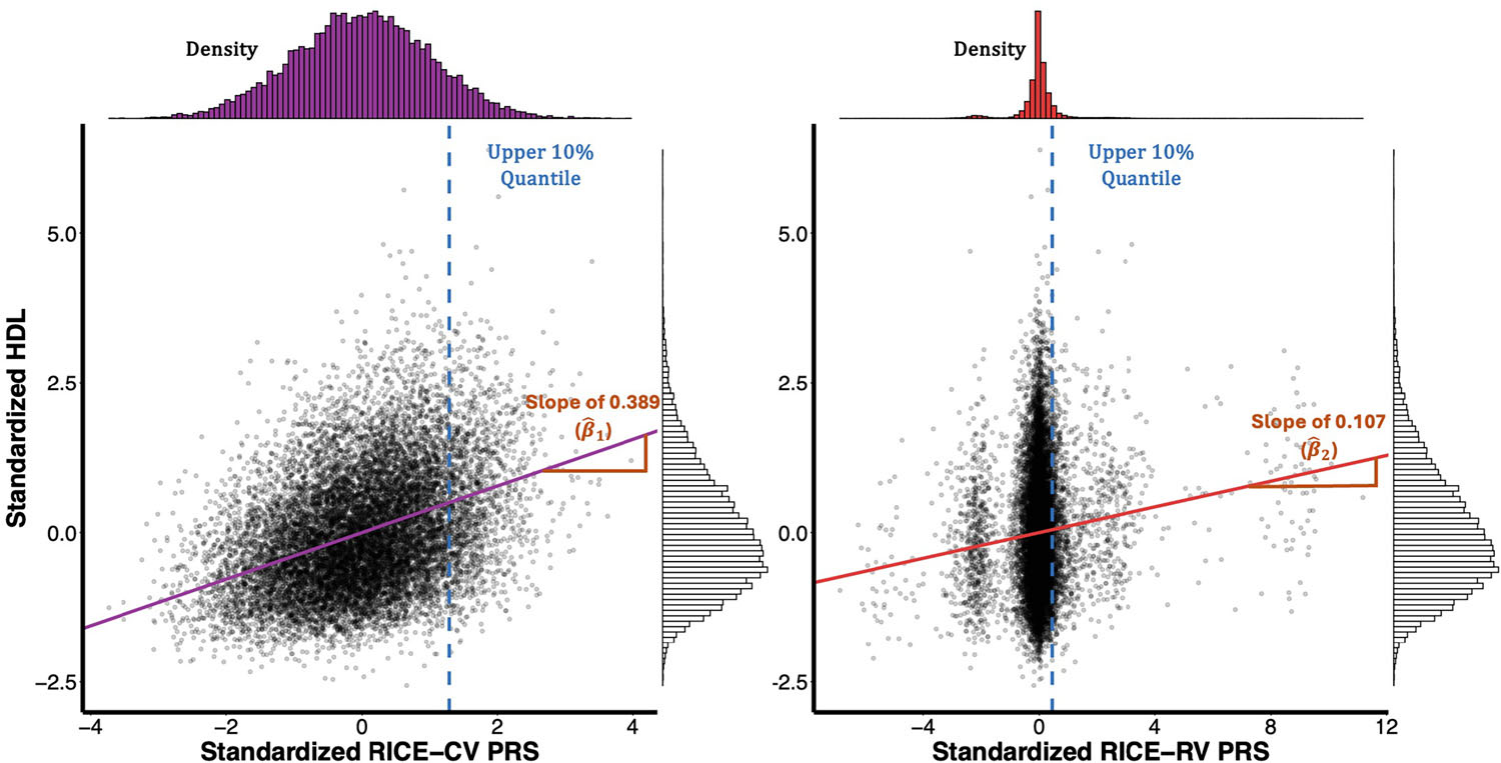
Comparison of standardized PRSs from RICE-CV and RICE-RV for high-density lipoprotein cholesterol (HDL). Standardized PRSs for HDL were computed using UK Biobank whole genome sequencing data for 13,839 individuals of European ancestry from the validation dataset. The left panel shows the standardized observed HDL levels plotted against the standardized PRS from RICE-CV (common variants), while the right panel shows the same comparison using RICE-RV (rare variants). In both figures, the blue dashed line represents the upper 10% quantile cutoff. The estimated regression coefficients (${\beta }_{1}$ for RICE-CV and ${\beta }_{2}$ for RICE-RV) are displayed in orange.

Figure 2 illustrates the estimated coefficients of the standardized PRS for both RICE-CV and RICE-RV using high-density lipoprotein cholesterol (HDL) levels from the UKB WGS dataset. For RICE-CV, a one-unit increase in the PRS is associated with a 0.389 standard deviation (SD) increase in HDL levels $({\beta }_{1}=0.389)$, while a one-unit increase in the RICE-RV PRS corresponds to a 0.107 SD increase $({\beta }_{2}=0.107)$. Notably, the distribution of RICE-RV PRS values is more dispersed compared to RICE-CV, with approximately 8.1% of individuals exhibiting PRS values greater than five units above the population mean. This demonstrates that while rare variants affect a smaller segment of the population, their impact on those individuals can be substantial.

### Simulation Study Results

We evaluated RICE’s ability to detect and predict rare variant effects using extensive simulation studies based on UKB WES data with 140,080 unrelated individuals (Methods). Genetic ancestries were inferred using 1000 Genomes Phase 3 Project 3 data (1000G, Methods)^56^. Since over 90% of individuals were of EUR ancestry, we designed simulations with an EUR-only training dataset (*N* = 98,343), while the tuning (*N* = 20,869) and validation datasets (*N* = 20,868) included individuals from AFR, AMR, EUR, and SAS ancestries (sample size details in Supplementary Data 1).

To evaluate RICE’s performance under realistic genetic architectures, we calibrated the relative contribution of rare variants by adopting a 1:12 ratio of rare variant burden heritability to common variant heritability, consistent with recent median estimates based on 22 complex traits ^36^. For our single-chromosome simulation framework, we set the common variant heritability $({h}_{{CV}}^{2})$ at 5% to ensure sufficient signal strength on chromosome 22 for evaluating model mechanics, such as weight estimation and prevention of overfitting, within a manageable computational burden. Accordingly, the rare variant heritability (${h}_{{RV}}^{2}$) was set at 0.42%.

**Fig. 3:**
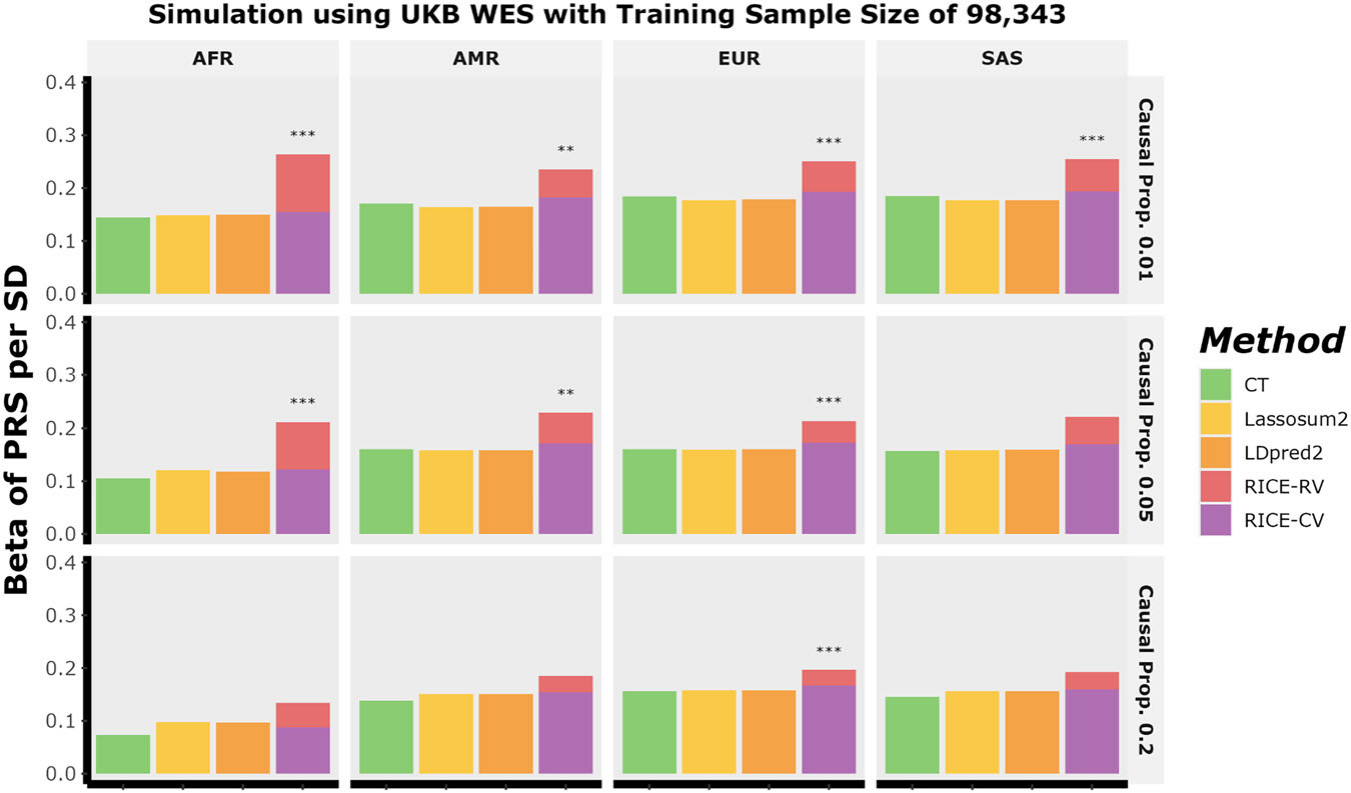
Simulation results comparing the predictive performance of PRSs for four ancestral groups from the UK Biobank (UKB). The training data (*N* = 98,343) included only individuals of European ancestry (EUR), while the tuning (*N* = 20,869) and validation datasets (*N* = 20,868) contained individuals of African (AFR), Admixed American or Latino (AMR), European (EUR), and South Asian (SAS) ancestries (Supplementary Data 1). Simulations assumed a common variant heritability of 0.05 and a rare variant set heritability of $4.17\times {10}^{-3}$, under the assumption of no negative selection effect size distribution (Methods). Causal proportions for both common variants and rare variant sets varied across three levels: 0.01 (top), 0.05 (middle), and 0.2 (bottom). Causal rare-variant sets were selected, and all variants within selected sets were assumed to contribute to the burden. Data were generated using unrelated individuals from UK Biobank whole-exome sequencing data (WES), with simulation based on chromosome 22. For each simulation scenario, 100 simulated traits were generated and results shown are the mean across the 100 validation-set evaluations. PRS performance is reported as the “Beta of PRS per standard deviation (SD)”, derived from the regression model $Y \sim {{\rm{PRS}}}\times \beta$, with $\beta$ representing the effect of standardized PRS on the standardized outcome (Methods). For RICE, the model used was $Y \sim {{\rm{PR}}}{{{\rm{S}}}}_{{{\rm{CV}}}}\times {\beta }_{{{\rm{CV}}}}+{{\rm{PR}}}{{{\rm{S}}}}_{{{\rm{RV}}}}\times {\beta }_{{{\rm{RV}}}}$. Statistical significance of the RICE-RV component was assessed using percentile bootstrap confidence intervals (10,000 resamples), testing the two-sided alternative ${H}_{A}:$ ${\beta }_{{{\rm{RV}}}}\ne 0$; *** indicates the lower bound of the 99% bootstrap confidence interval (CI) > 0 and ** indicates the lower bound of the 95% bootstrap CI > 0. Exact bootstrap *p* values and CI bounds are provided in the Source Data file. Source data are provided as a Source Data file.

RICE-CV was constructed by applying single-ancestry common variant PRS methods: CT, LDpred2, and Lassosum2 to the EUR training data. We compared the performance of RICE-CV and RICE-RV to these methods across different ancestries. The rare variant analysis was conducted using STAARpipeline with protein-coding genes within coding regions. RICE was evaluated across various simulation settings, including different causal variant proportions (1%, 5%, and 20%), causality structures of causal rare variant sets (a proportion or all rare variants within a set are causal), training sample size (*N* = 49,173 and 98,343), and effect size distributions (strong negative selection and no negative selection, Methods). RICE-CV consistently demonstrated robust performance (Fig. 3 and Supplementary Fig. 1). By leveraging the strengths of different common variant PRS methods, RICE-CV achieved higher prediction accuracy than any single approach. On average, RICE-CV yielded the highest standardized effect size across all ancestries and traits, showing consistent gains over the best existing single-method common variant PRS.

RICE-RV also performed strongly, effectively identifying causal rare variant signals and predicting their effects across both EUR and non-EUR populations (Fig. 3 and Supplementary Fig. 1). When added to RICE-CV, RICE-RV consistently contributed to independent predictive information. The standardized effect sizes for RICE-RV were significantly greater than zero for EUR across most scenarios ($p < 0.05$), whereas in non-EUR populations, significance was generally observed only with larger sample sizes and higher proportions of causal variants. Notably, RICE-RV showed particularly strong performance in AFR individuals, as the randomly selected causal variants and rare variant sets across the genome led to higher average burden scores (combined allele frequencies) in this ancestry (Supplementary Fig. 2), driving more notable rare variant prediction performance. Overall, RICE-CV achieved the highest prediction accuracy among common variant PRSs across all populations in most scenarios, while RICE-RV correctly identified causal rare variants and accurately predicted their impact.

### UKB imputed + WES and WGS results

We applied RICE to compute PRSs for 11 complex traits, with six continuous traits: BMI, HDL, height, low-density lipoprotein (LDL), log-transformed triglycerides (log(TG)), and total cholesterol (TC), and five binary traits: asthma, breast cancer, coronary artery disease (CAD), prostate cancer, and T2D. Training data included 98,103 EUR individuals, while tuning (*N* = 20,869) and validation (*N* = 20,868) datasets included individuals from AFR, AMR, EUR, and SAS ancestries (sample size details in Supplementary Data 2, 3; variant counts in Supplementary Data 9). We conducted association analyses as follows: for common variants, we adjusted for top 10 PCs, sex, age and age squared. For rare variant association testing, we further included RICE-CV as an additional covariate to ensure independence between RICE-RV and RICE-CV (Methods). Genomic control metrics indicated well-calibrated analyses, with ${\lambda }_{1000}$ ranging between 1 to 1.018. LD score regression intercepts^57^ further confirmed minimal population stratification (intercept < 1.2) across all traits (Supplementary Data 4). Manhattan and QQ plots for common and rare variant association tests are available for both analyses: Imputed + WES (Supplementary Figs. 3, 4), and WGS (Supplementary Figs. 5, 6).

We used two analytical configurations: (1) Imputed + WES, using imputed genotypes for common variants and WES data for rare variants; and (2) WGS, using WGS for both common and rare variant analyses. Across both configurations, RICE-CV consistently outperformed existing common variant PRS methods for continuous traits, with average ${R}^{2}$ improvements of 9.4% over the best alternative method per trait-ancestry combination (Supplementary Data 6, 7). Incorporating rare variants via RICE-RV yielded further gains, particularly for lipid traits (HDL, LDL, log(TG), and TC), where improvements were driven by genes with established roles in lipid metabolism^58–60^(e.g., *APOC3* aggregated weight for HDL = 0.516, 3.8 standard deviations above the average aggregated effect size; full model weights are available on Harvard Dataverse as described in the Data Availability statement). For height, RICE-RV provided modest but statistically significant improvements in EUR and AMR, while BMI showed limited gains likely due to its polygenic architecture with smaller rare variant effects, and binary traits exhibited no improvements, probably owing to lower case numbers. Below, we detail results by each configuration.

**Fig. 4:**
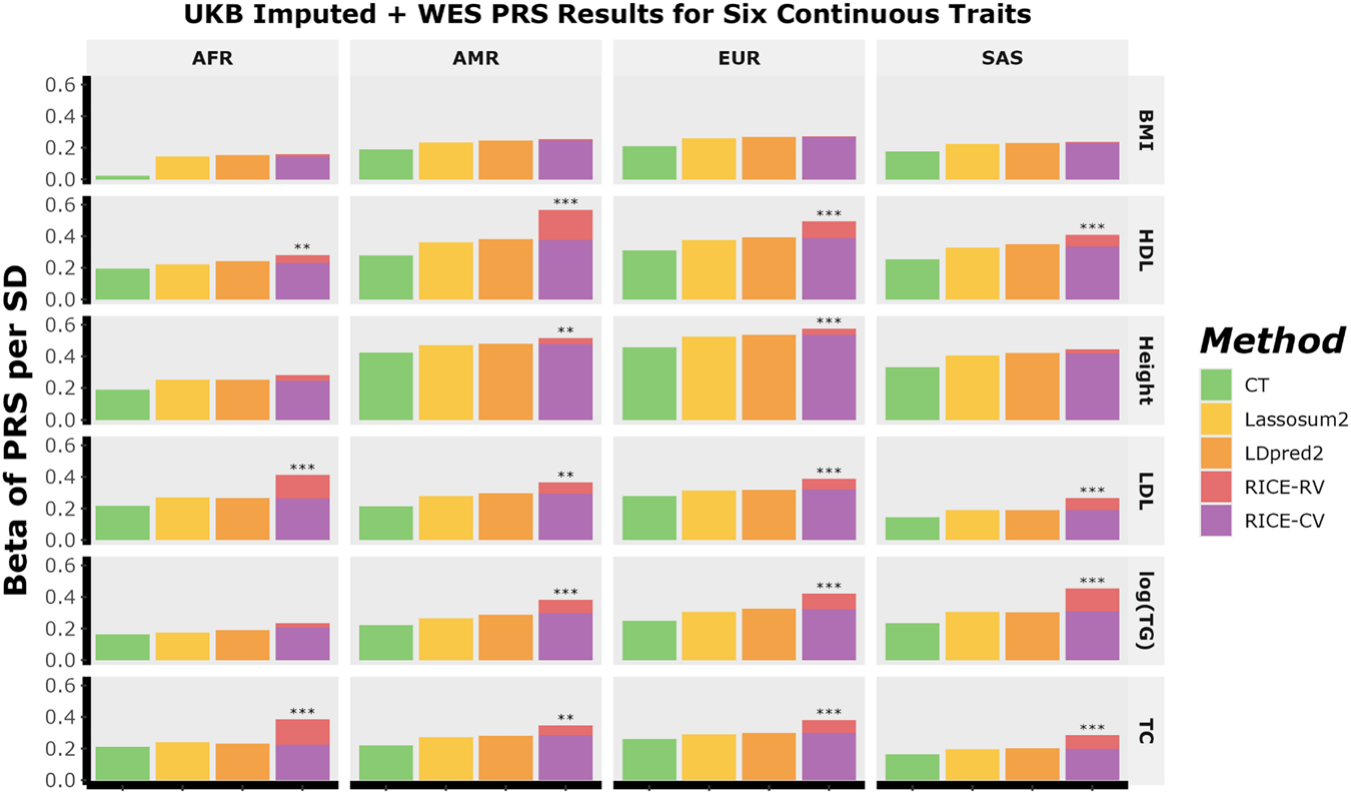
Predictive performance of ancestry-adjusted PRSs for continuous traits across four ancestral groups from UK Biobank (UKB) imputed + whole-exome sequencing (WES) data. The traits analyzed include body mass index (BMI), height, high-density lipoprotein cholesterol (HDL), low-density lipoprotein cholesterol (LDL), the natural logarithm of triglyceride cholesterol (log(TG)), and total cholesterol (TC). Results are shown for individuals of African (AFR), Admixed American or Latino (AMR), European (EUR), and South Asian (SAS) ancestries. The training data consisted solely of individuals of European ancestry, while tuning and validation sets included all four ancestries. Full sample size details for each ancestry are provided in Supplementary Data 2. Statistical significance of the RICE-RV component was assessed using percentile bootstrap confidence intervals (10,000 resamples), testing the two-sided alternative ${H}_{A}:{\beta }_{{{\rm{RV}}}}\ne 0$; *** indicates the lower bound of the 99% bootstrap confidence interval (CI) > 0 and ** indicates the lower bound of the 95% bootstrap CI > 0. Exact bootstrap *p* values and CI bounds are provided in the Source Data file. Source data are provided as a Source Data file.

In the UKB Imputed + WES analysis, we utilized imputed genotypes for common variants to ensure comprehensive genome-wide coverage, while retaining WES-based rare variants. Among common variant methods, RICE-CV generally achieved the largest standardized effect sizes across continuous traits (standardized effect sizes in Fig. 4 and Supplementary Fig. 7a; ${R}^{2}$/AUC in Supplementary Figs. 7b, c; quantile differences in Supplementary Fig. 8). Among common variant methods, RICE-CV generally achieved the largest standardized effect sizes across continuous traits (Fig. 4). For rare variants, RICE-RV showed significant standardized effect sizes ($p < 0.05$) for all lipid traits across ancestries and for height in EUR and AMR (Fig. 4). Relative to the best alternative method, RICE (CV + RV) improved ${R}^{2}$ for lipid traits in EUR by 4.9–8.0% (Supplementary Fig. 7c). Notable non-EUR gains included 29.8% for TC in AFR, 25.9% for log(TG) in AMR, and 25.5% for HDL in AMR (Supplementary Fig. 7c). For height, the rare variant component was statistically significant but small (e.g., $\beta=$0.039 in EUR and 0.038 in AMR), and corresponding ${R}^{2}$ gains were marginal, leaving RICE’s performance not significantly different from the best alternative for this trait, consistent with a highly polygenic rare variant architecture that yields small effects at the population level. Quantile stratification across ancestries showed clear gradients for lipid traits in EUR, with significant differences between extreme quantiles (Supplementary Fig. 8b, d–f).

**Fig. 5:**
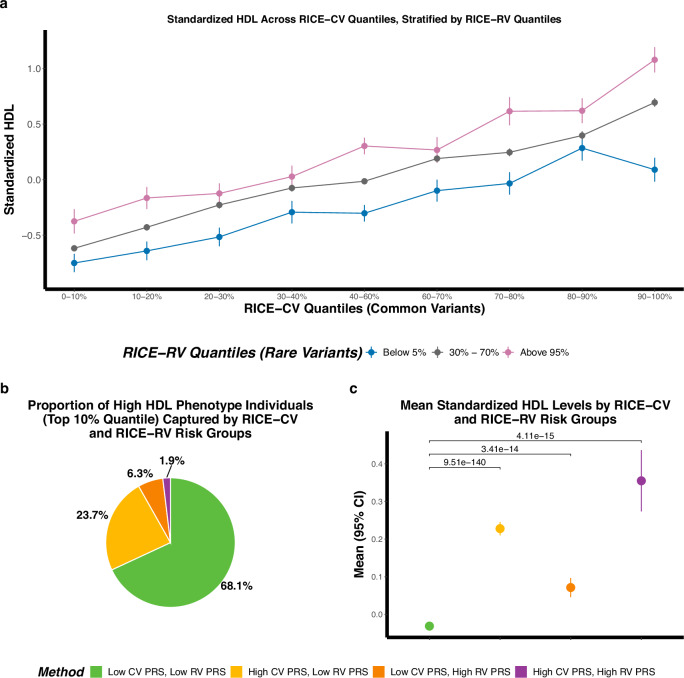
Joint stratification of common and rare variant PRS identifies discrepant high-risk individuals. Analyses were performed in the UK Biobank (UKB) imputed genotype and whole-exome sequencing (WES) validation dataset (*n* = 18,150). High-density lipoprotein cholesterol (HDL) was residualized for age, age^2^, sex, and the first 10 genetic principal components, then standardized; PRSs were ancestry-adjusted as described in Methods. **a** Mean standardized HDL levels $\pm$ $1\times$ standard error (SE) are plotted stratified by PRS quantiles for RICE-CV (common variants) on the x-axis and RICE-RV (rare variants) by color (blue: below 5%, gray: 30–70%, pink: above 95%). **b** Participants were cross-classified into four strata based on RICE-CV (high = top 10% vs low = bottom 90%) and RICE-RV (high = top 5% vs low = bottom 95%): Low CV/Low RV (*n* = 1248), High CV/Low RV (*n* = 435), Low CV/High RV (*n* = 115), and High CV/High RV (*n* = 35). Pie chart shows the proportion of individuals in each stratum among the top decile of observed HDL values (*n* = 1833). **c** Mean standardized HDL for each stratum with 95% confidence intervals (mean $\pm$ $1.96\times$SE). Pairwise *p* values were calculated using two-sided Welch’s *t*-tests comparing each stratum to the Low CV/Low RV group; exact *p* values and sample sizes are provided in the Source Data file. Source data are provided as a Source Data file.

To test whether rare variants flag individuals missed by common variant PRS, we cross-classified participants by RICE-CV (top 10% vs. bottom 90%) and RICE-RV (top 5% vs. bottom 95%), yielding four strata (Methods). For HDL, stratifying by RICE-RV quantiles shows significant differences across RICE-CV quantiles (Fig. 5a). Further, 6.3% of individuals in the top phenotype decile were captured only by high RICE-RV despite low RICE-CV (Fig. 5b). Group-level comparisons confirmed that the low-CV/high-RV stratum had significantly higher HDL than the low-CV/low-RV group (*p* = 3.41 × 10⁻^14^; Fig. 5c). Similar patterns were seen for other lipid traits but not for BMI, consistent with the standardized effect-size results (Supplementary Fig. 9). These findings show that the rare variant component of RICE can identify individuals with extreme lipid profiles who would be missed by common variant PRS alone.

To quantify the benefit of genome-wide rare variant signal beyond established high-penetrance genes, we compared RICE-RV to a gene-restricted baseline that used only burden scores from *LDLR*, *APOB*, and *PCSK9* (with identical ensemble learning) for HDL, LDL, log(TG), and TC. Across ancestries, RICE-RV consistently outperformed this baseline, yielding 196% higher standardized effect size for Europeans ($\beta$ per SD) (Supplementary Fig. 10), indicating contributions from additional modest-effect rare variant sets. We assessed sensitivity to the STAAR-Burden inclusion threshold by varying the *p* value cut-off from 1 × 10⁻^5^ to 1 × 10⁻^2^; performance was stable with no monotonic improvement at more liberal thresholds (Supplementary Fig. 11). Balancing computational cost and signal capture, we retained 1 × 10⁻^3^ for the main analyses.

We also assessed the computational efficiency of the RICE framework (Supplementary Data 10). Ensemble learning for RICE-CV and RICE-RV required on average 1.73 compute hours per trait (~1.1% of total compute time). Rare variant association testing accounted for the largest compute share (106.7 h, 381 parallel jobs, 68.6%), followed by LDpred2/Lassosum2 modeling (28.4 h, 18.9%).

Using the UKB WGS (with comprehensive variant coverage) reproduced the overall patterns seen in Imputed + WES (standardized effect sizes in Supplementary Fig. 12a, c; ${R}^{2}$/AUC in Supplementary Fig. 12b, d; quantile stratification in Supplementary Fig. 13). Among common variant methods, RICE-CV generally achieved the largest standardized effect sizes across continuous traits. For rare variants, RICE-RV showed significant effects ($p < 0.05$) for height and all lipid traits across ancestries, with BMI only being significant for SAS (Supplementary Fig. 12c). Relative to the best alternative method, RICE (CV + RV) increased ${R}^{2}$ in EUR by 7.5% (height), 8.6% (HDL), 11.2% (LDL), 9.6% (log(TG)), and 10.6% (TC) (Supplementary Fig. 12d). Notable non-EUR gains included 60.7% for log(TG) in AFR and 29.8% for HDL in AMR. In contrast, binary traits again showed limited improvement. In EUR, stratifying individuals by RICE-RV quantiles revealed clear lipid gradients: for HDL, the lowest quantile averaged ~0.22 SD lower and the highest ~0.32 SD higher than the middle group (Supplementary Fig. 13), indicating that rare variants help identify individuals with markedly extreme lipid profiles that common variant PRS alone may miss. Due to smaller validation samples, trends were less stable in non-EUR groups.

We compared (1) regression-based adjustment for population structure (Methods; Supplementary Note) versus (2) ancestry-specific standardization using group-specific means/SDs. Performance was comparable (Supplementary Fig. 14). To avoid explicit ancestry categorization while adequately controlling structure, we adopted the regression-based approach in the main analyses.

**Fig. 6:**
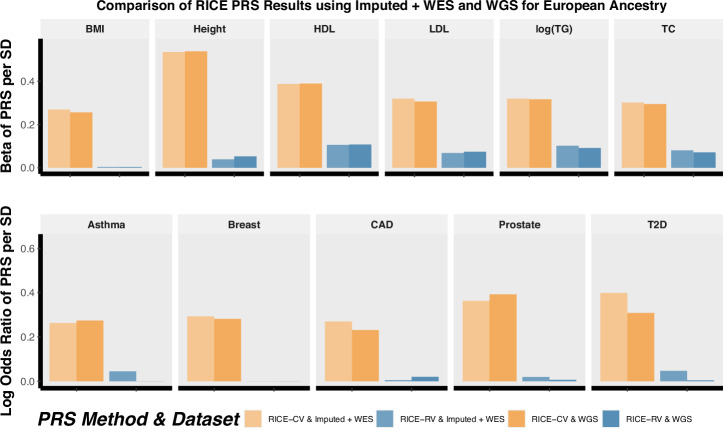
Comparison of ancestry-adjusted PRSs from RICE-CV and RICE-RV using UK Biobank (UKB) in two configurations: imputed + WES (imputed genotypes for common variants and whole exome sequencing data for rare variants) and WGS (whole-genome sequencing data for both common and rare variants). Traits include six continuous traits: body mass index (BMI), height, high-density lipoprotein cholesterol (HDL), low-density lipoprotein cholesterol (LDL), the natural logarithm of triglyceride (log(TG)), and total cholesterol (TC), and five binary traits: asthma, breast cancer, coronary artery disease (CAD), prostate cancer, and type 2 diabetes (T2D). The training data included only individuals of European ancestry, while the tuning and validation sets contained individuals from all four ancestries. This figure shows results for European ancestry individuals, and results for all other ancestries are provided in Supplementary Fig. 15. Full sample size details for each ancestry are provided in Supplementary Data 2 and 3. Source data are provided as a Source Data file.

When comparing UKB imputed data (~1.48 million common variants) to UKB WGS (~5.47 million common variants), we observed that further expansion to WGS did not yield additional improvements over imputed data for RICE-RV (Fig. 6, Supplementary Fig. 19). This indicates high accuracy of imputation for common variants in UKB. For RICE-RV, expanding rare variants from ~17 million in WES to 734 million in WGS did not result in noticeable predictive gains (Fig. 6, Supplementary Fig. 15). The lack of improvement may be due to rare variant signals being predominantly located in coding regions, with variants located in noncoding regions contributing less independent signal. A further breakdown of RICE-RV results by coding and noncoding regions revealed that variants in noncoding regions provided limited predictive signal compared to coding region variants (Supplementary Fig. 16).

### All of Us results

We applied RICE and compared its performance against other existing multi-ancestry PRS methods across six continuous traits in the AoU WGS dataset (Methods). Covariate adjustments followed similar procedures as UKB analyses. The training dataset (*N* = 155,611) consisted of individuals from AFR, AMR, and EUR ancestries, while the tuning (*N* = 33,342) and validation (*N* = 33,363) datasets included AFR, AMR, EAS, EUR, MID, and SAS populations (sample size details in Supplementary Data 5, variant count in Supplementary Data 9). Common variants were defined as those with MAF > 0.01 in any of the three ancestries (AFR, AMR, or EUR) in the training dataset. Rare variant analyses were constrained to the exome region for computational efficiency (Methods). The genomic inflation factor was well controlled, with ${\lambda }_{1000}$ ranging from 1 to 1.003 (Supplementary Data 4), with LD score regression intercepts^57^ confirming minimal population stratification across traits and ancestries. Manhattan and QQ plots for common and rare variant association tests are provided in Supplementary Figs. 17, 18.

**Fig. 7:**
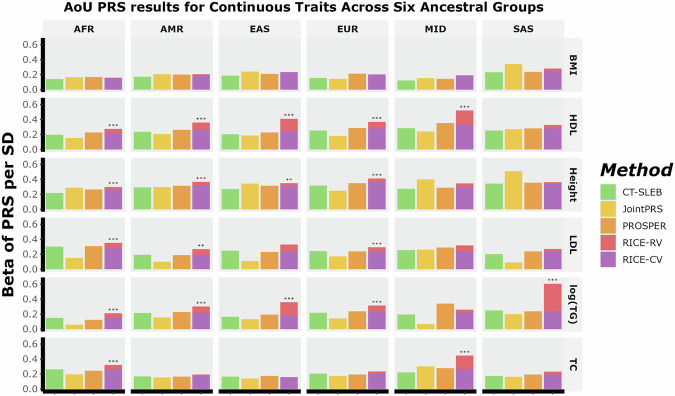
Predictive performance of ancestry-adjusted PRSs for continuous traits across six ancestral groups from the All of Us (AoU) whole-exome sequencing (WES) data. Traits include body mass index (BMI), height, high-density lipoprotein cholesterol (HDL), low-density lipoprotein cholesterol (LDL), the natural logarithm of triglyceride (log(TG)), and total cholesterol (TC). Results are shown for individuals of African (AFR), Admixed American or Latino (AMR), East Asian (EAS), European (EUR), Middle Eastern (MID), and South Asian (SAS) ancestries. Training data consisted of individuals of EUR, AFR, and AMR ancestry, while tuning and validation sets included individuals from all six ancestries. Full sample size details for each ancestry and dataset are provided in Supplementary Data 5. Statistical significance of the RICE-RV component was assessed using percentile bootstrap confidence intervals (10,000 resamples), testing the two -sided alternative ${H}_{A}:{\beta }_{{{\rm{RV}}}}\ne 0$; *** indicates the lower bound of the 99% bootstrap confidence interval (CI) > 0 and ** indicates the lower bound of the 95% bootstrap CI > 0. Exact bootstrap *p* values and CI bounds are provided in the Source Data file. Source data are provided as a Source Data file.

Performance patterns largely mirrored UKB (standardized effect sizes in Fig. 7; ${R}^{2}$ in Supplementary Fig. 19; quantile differences in Supplementary Fig. 20). Among common variant methods, RICE-CV showed robust performance across most trait–ancestry pairs, typically matching or modestly exceeding the best alternatives (Fig. 7). Two exceptions were HDL in MID and BMI in SAS, where JointPRS performed better, likely reflecting smaller validation sample sizes in these populations. The rare variant component RICE-RV contributed significantly ($p < 0.05$) for several traits, with the clearest signals in lipids and height, though patterns varied by ancestry. Significance was observed in: EUR (HDL, height, LDL, log(TG)); AFR (HDL, height, log(TG), TC); AMR (HDL, height, LDL, log(TG)); EAS (HDL, height, log(TG)); MID (HDL, TC); and SAS (log(TG) only). No ancestry showed a significant RV effect for BMI. Consistent with this, the RV contribution to standardized effects was substantial for lipids, for example, for HDL, the RICE-RV coefficient was ~26–31% of the RICE-CV coefficient in EUR and ~24–40% across non-EUR ancestries; for log(TG), the corresponding ratios were ~22–36.5% (EUR) and ~32–70% (non-EUR). For explained variance, the full RICE model (CV + RV) improved *R*^*2*^ by an average of 1.7% over the best existing method across trait-ancestry pairs (Supplementary Fig. 19). Notable gains included 14.2% for height in EUR, 43.2% for HDL in EAS, and 18.5% for TC in AFR. In contrast to UKB, we did not observe significant ${R}^{2}$ gains for lipid traits in EUR within AoU, likely reflecting smaller training sample sizes (average *N* = 65,549 per lipid trait in AoU vs. *N* = 91,356 in UKB).

Larger AFR and AMR sample sizes in AoU enabled clearer phenotypic gradients when stratifying by RICE-RV quantiles (below 5%, 30–70%, above 95%) for height and lipid traits (Supplementary Fig. 20b–f). For example, AFR individuals in the top 95% quantile of height were on average 0.07 units higher than those in the middle group and 0.41 units higher than those in the bottom 5% quantile. However, separations in EAS, SAS, and MID individuals were less pronounced due to smaller sample sizes (Supplementary Fig. 20b–f).

### Evaluation of AoU PRS on UKB

**Fig. 8:**
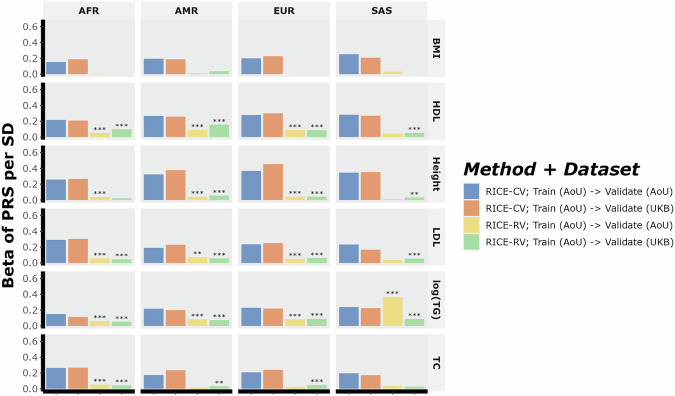
Predictive performance of RICE trained on All of Us (AoU) data and evaluated on both AoU and UK Biobank (UKB) validation datasets. Analyzed traits include body mass index (BMI), height, high-density lipoprotein cholesterol (HDL), low-density lipoprotein cholesterol (LDL), log-transformed triglycerides (log(TG)), and total cholesterol (TC). Results are shown for African (AFR), Admixed American/Latino (AMR), European (EUR), and South Asian (SAS) ancestries. Training used EUR, AFR, and AMR individuals from AoU (sample sizes in Supplementary Data 5), with validation on AFR, AMR, EUR, and SAS individuals from AoU and all UKB individuals (Supplementary Data 2). Statistical significance of the RICE-RV component was assessed using percentile bootstrap confidence intervals (10,000 resamples), testing the two -sided alternative ${H}_{A}:{\beta }_{{{\rm{RV}}}}\ne 0$; *** indicates the lower bound of the 99% bootstrap confidence interval (CI) > 0 and ** indicates the lower bound of the 95% bootstrap CI > 0. Exact bootstrap *p* values and CI bounds are provided in the Source Data file. Source data are provided as a Source Data file.

To assess the cross-dataset portability of RICE PRSs, we applied models trained on AoU data (using AFR, AMR, and EUR individuals for training; Methods) to the UKB validation dataset and compared performance against validation within AoU (standardized effect sizes in Fig. 8; ${R}^{2}$ in Supplementary Fig. 21). Analyses focused on six continuous traits across AFR, AMR, EUR, and SAS ancestries (full sample sizes in Supplementary Data 2 and 5). RICE PRSs demonstrated strong portability, with effect sizes from AoU-trained models remaining robust when evaluated in UKB (Fig. 8). Average standardized effect sizes for RICE-CV were 0.241 in AoU and 0.247 in UKB across traits and ancestries, while those for RICE-RV were 0.059 in AoU and 0.053 in UKB. RICE-RV retained significant associations ($p < 0.05$) in UKB for many of the same traits as in AoU, including HDL, height, LDL, and log(TG) in EUR; HDL, height, log(TG), and TC in AFR; HDL, height, LDL, and log(TG) in AMR; and log(TG) in SAS (Fig. 8). No significance was observed for BMI in either dataset. Notably, ${R}^{2}$ values were often higher in UKB than in AoU, with an average relative increase of 45.0% for the full RICE model (Supplementary Fig. 21). This enhanced performance in UKB may reflect superior phenotype quality or measurement consistency in that cohort, highlighting RICE’s generalizability across diverse datasets.

## Discussion

Understanding the role of rare genetic variants is crucial for unraveling the genetic architecture of complex traits. We introduced RICE, a PRS framework that integrates both common and rare genetic variants to enhance genetic risk prediction across diverse ancestries. Using large-scale sequencing data from UKB and AoU studies, RICE significantly improves predictive accuracy compared to leading common variant PRS methods, particularly for traits with distinct rare variant architectures. For lipid traits, RICE achieved substantial gains (e.g., up to ~8% increase in *R*^2^), enabling more precise risk stratification across multiple populations.

Our findings showed that RICE-CV, the common variant component, consistently delivered robust predictive accuracy, often slightly exceeding or matching existing common variant PRS methods across ancestries and traits (Figs. 4, 7, Supplementary Figs. 7, 12, and 19). By combining multiple PRS approaches, RICE-CV captured a broader spectrum of polygenic signals. This ensemble framework highlights that while no single method is best for all scenarios^22,25,55^, integrating diverse approaches can optimize predictions across populations.

Importantly, RICE-RV enhanced predictive power by incorporating rare variants, particularly improving risk prediction for lipid-related traits, while showing limited gains for BMI (Figs. 4, 7, Supplementary Figs. 7, 12, and 19). These results highlight that the predictive value of rare variants is not universal but strongly dependent on both the underlying genetic architecture and available statistical power. For lipid traits, our findings align with previous heritability studies indicating that rare variants contribute more significantly to certain traits^36^. For instance, Weiner et al.^36^ estimated that the ratio between exome burden heritability and common variant heritability was approximately 0.231 for LDL, but only 0.048 for BMI. In our models, this substantial improvement reflected RICE’s ability to capture distinct rare variant architectures, aggregating signals from both established high-impact genes (e.g., *APOB*, *LDLR*) and broader polygenic rare variation (Supplementary Fig. 10). In contrast, the negligible improvements for BMI suggest that its genetic architecture is likely dominated by infinitesimal common variant effects, or that rare variant effects are too diffuse to be effectively captured by gene-centric burden testing. Furthermore, for binary traits or diseases, the limited gains should be interpreted in the context of statistical power. Unlike quantitative traits, where the effective sample size includes the entire cohort, analyses in population-based cohorts like the UK Biobank are restricted to the subset of cases. The resulting lower case counts substantially reduce the power to accurately estimate rare variant weights, limiting the utility of RICE-RV in this specific study design.

Traditional evaluation metrics like ${R}^{2}$ may underestimate the impact of rare variants because they focus on average effects across the population. The regression coefficient of the standardized PRS on the standardized trait provides a direct measure of the PRS effect size (equivalent to correlation and subject to attenuation bias due to PRS measurement error^61^), offering an interpretable assessment of predictive performance (e.g., per-SD effect). Our analyses demonstrated that, despite affecting fewer individuals, rare variants can have significant effects on trait values, reinforcing the importance of including them in PRS models.

We emphasize that the reporting of standardized effect sizes is intended to provide a measure of the expected phenotypic shift for individual carriers, an interpretation that is often more intuitive in clinical settings than variance explained. However, it is important to acknowledge the mathematical coupling between these metrics; for a standardized PRS, the Beta per SD is equivalent to the square root of the heritability explained by the score. Consequently, for rare variants, these effect sizes should be interpreted with caution regarding population-level impact. A substantial per-SD effect can be observed even when the overall *R*^2^ is modest, as the low frequency nature of rare variants inherently limits the total variance they can explain across the entire population.

One contribution of our study is the comprehensive assessment of rare variants’ contributions across large and diverse datasets. Rare variants are often overlooked in risk prediction models due to complexity and challenges in modeling them^1,2^. Existing efforts have focused on combining high-penetrance genes with common variant PRSs^62–64^, as seen in breast cancer risk prediction models that include genes such as *BRCA1*, *BRCA2*, *PALB2*, *CHEK2*, and *ATM* ^62^. While these approaches effectively integrate high-effect genes, they may miss modest-risk rare variant sets. By incorporating functional annotations using the STAARpipeline^48^, RICE-RV can identify rare variant sets beyond those described in existing literature, leveraging larger sample sizes and diverse ancestries to capture effects of rare variants across functionally distinct regions (e.g., predicted loss-of-function (pLoF) or deleterious missense variants). This enables us to capture the varying effect sizes of rare variants across functional regions, as shown in previous studies where loss-of-function variants often exhibit large effects^65–68^.

Our results further indicated that rare variant signals contributing to PRS were largely captured in coding regions, while the contribution of rare variants in noncoding regions was comparatively weaker (Fig. 6). This observation aligns with findings from recent association testing studies^69,70^. For example, Gaynor et al.^70^ reported that the gene-based association signals from WGS data differed by only 1% from those derived from WES plus imputation, suggesting that the most actionable signals for prediction are heavily concentrated in coding regions. Similarly, Karczewski et al.^41,69^ demonstrated that rare coding variants, particularly pLoF variants, are significantly enriched for disease associations compared to synonymous or non-coding variants. However, this concentration of predictive signal contrasts with recent heritability estimates. Wainschtein et al.^71^ recently estimated that non-coding regions account for the majority (~79%) of total rare variant heritability due to their vast size. This apparent discrepancy likely reflects the structural limitations of burden-based aggregation for capturing diffuse signals. RICE relies on aggregating variants to amplify signal. In coding regions, the high per-variant heritability enrichment allows burden tests to effectively distinguish risk carriers. In contrast, the heritability in non-coding regions is likely more diffuse and intermixed with a vast number of neutral variants; aggregating these sparser signals over broad genomic windows likely dilutes the true effects with noise, rendering the resulting burden scores non-predictive. Thus, while non-coding regions harbor substantial latent heritability, coding regions currently offer the most accessible signal architecture for effective polygenic risk modeling.

An important feature of RICE to provide a single PRS for common variants applicable across ancestries. Existing methods like CT-SLEB^22^ and PROSPER^25^ are often optimized for specific ancestries, requiring ancestry-specific tuning. While these methods may achieve high performance in a target ancestry, they complicate clinical translation by necessitating prior knowledge of a patient’s genetic ancestry. In contrast, RICE-CV uses a mixed-ancestry tuning dataset that combines results from multiple methods, yielding a single PRS applicable across ancestries. Our results showed that this approach achieved higher or comparable performance to the best alternative methods in nearly all traits and ancestries (Fig. 7), simplifying its potential clinical utility.

Our cross-dataset evaluation further demonstrated RICE’s robustness, with AoU-trained PRSs performing comparably or better when applied to UKB, despite differences in sequencing technologies (WGS in AoU vs. imputed + WES in UKB) and reduced variant overlap. Effect sizes remained stable, and ${R}^{2}$ often increased in UKB (Fig. 8; Supplementary Fig. 21), potentially due to phenotype quality or measurement consistency in UKB, while rare variant signals showed consistent results with only minor attenuation, highlighting RICE’s consistent predictive performance across real-world datasets. This portability supports RICE’s utility in federated or multi-biobank settings, where training and application cohorts differ.

Our analyses demonstrated that incorporating rare variants in the coding region enhances the prediction of lipid traits, with genes like *APOC3, APOB*, and *LDLR* emerging as key contributors in our models, consistent with existing findings in lipid metabolism^72–74^. For example, rare loss-of-function variants in *APOC3* have been associated with lower triglyceride levels and reduced coronary artery disease risk^74^. This alignment with known biological mechanisms reinforces the validity of our results, highlighting the relevance of these genes in clinical risk assessment and therapeutic strategies, while also offering new opportunities for biological insights. A comparison to high-penetrance genes alone (*LDLR, APOB, PCSK9*) showed that RICE-RV’s genome-wide approach adds substantial value for lipids (Supplementary Fig. 10).

Despite the promising results, our study has several limitations. First, regarding modeling simplifications: RICE aggregates rare variant into burden scores. While efficient, this approach assumes equal weighting of all variants, potentially overlooking differences in pathogenicity^46–48,75–78^. Furthermore, while the STAAR framework can detect non-linear signals (e.g., via SKAT^47^), RICE prioritizes STAAR-Burden (STAAR-B) to align with its additive prediction model; this ensures consistency but may fail to capture complex epistatic interactions, suggesting a need for future non-linear integration methods. Second, regarding data constraints: computational limits restricted rare variant inclusion to sets with $p < {1\times 10}^{-3}$, though sensitivity analyses showed stable performance across thresholds (Supplementary Fig. 11). Additionally, limited case counts for binary outcomes currently restrict utility, necessitating larger disease-specific consortia. Future applications in these cohorts could employ metrics like the net reclassification index to better assess improvements in high-risk individual identification and clinical decision-making. Finally, regarding generalizability: while RICE leverages individual-level data for optimal conditioning, it can adapt to external summary statistics (e.g., large-scale GWAS for CV and STAARpipeline results for RV). However, challenges may arise if these summary statistics are from disparate samples, potentially compromising independence between components. Future extensions could incorporate orthogonalization techniques to maintain component independence when using disparate summary statistics, or adopt summary statistics-based fine-tuning approaches^79–82^ to eliminate the requirement for an independent tuning data.

In conclusion, we developed RICE, a PRS framework that integrates both common and rare variants within a single model to improve genetic risk prediction across diverse ancestries. Our study demonstrates that incorporating rare coding variants enhances the predictive power of PRSs for lipid-related traits, particularly for individuals carrying these variants who may experience significant impacts on trait values. By providing open-source software for RICE, we offer a practical tool to advance polygenic risk prediction and contribute to precision medicine.

## Methods

We developed RICE, a framework that integrates both common and rare genetic variants to enhance polygenic risk prediction across diverse populations. RICE has three primary components: (1) RICE-CV, which constructs a robust PRS for common variants using ensemble regression; (2) RICE-RV, which identifies and incorporates rare variant signals into the PRS; and (3) a final evaluation step that combines the PRSs from RICE-CV and RICE-RV within a regression model to produce an integrated risk prediction (Fig. 1).

### Ethics

This study used de-identified data from the UK Biobank under approved application 52008 and from the All of Us Research Program under controlled-tier access via the Researcher Workbench. UK Biobank has research ethics approval from the North West Centre for Research Ethics Committees (REC reference 11/NW/0382). All of Us participants are consented under the All of Us research protocol, approved by the All of Us Institutional Review Board. Additional information on All of Us IRB oversight is available at https://allofus.nih.gov/about/who-we-are/institutional-review-board-irb-of-all-of-us. Analyses were conducted using de-identified data within the respective secure analysis environments.

### Data processing and standardization

All analyses employ a three-way data split into independent training, tuning, and validation datasets. The training dataset is used to compute GWAS summary statistics, train common variant PRS models, perform rare variant association testing, compute burden scores, and train rare variant PRS models using penalized regression (LASSO and ridge regression). The tuning dataset is used to train the ensemble learning model, combining PRSs generated by different methods and tuning parameters, using LASSO and ridge regression as base learners. The final validation dataset is a fully independent, held-out test set used only to evaluate the performance of the final PRSs.

To ensure consistency and comparability across ancestries during the tuning and validation stages, we implemented specific standardization procedures for phenotypes and PRSs. For the initial association testing of common variants in GWAS and rare variants in STAARpipeline within the training dataset, we used the original phenotype values. These analyses adjusted for covariates including the top 10 PCs, sex, age, and age squared. Association testing of common variants was performed with REGENIE^83^. Rare variant association testing using STAARpipeline^48^ was performed with linear regression for continuous traits and logistic regression for binary traits, aligning with standard association testing practices.

During the ensemble learning for RICE-CV and RICE-RV in the tuning dataset and fitting LASSO and ridge regression models for burden scores in the training dataset (detailed in the later sections), continuous phenotypes were adjusted by regressing out the same set of covariates to obtain residuals. These residuals were used as inputted outcomes in the mentioned procedures. Binary traits remained on their original scale throughout all stages.

In the validation stage, we standardized the residualized continuous phenotypes within each ancestry group to have a mean of 0 and a variance of 1. PRSs generated from existing methods, RICE-CV, and RICE-RV are also standardized within each ancestry to have a mean of 0 and a variance of 1, following methods outlined in prior works^4,10^ and described in the Supplementary Note. This standardization ensured that both phenotypes and PRSs were on a common scale across ancestries, facilitating fair comparisons during performance evaluation. All PRS methods evaluated in the manuscript followed identical evaluation procedure.

### RICE-CV: common variant PRS modeling

The RICE-CV component has two main steps: (1) PRS training and (2) ensemble learning.

#### PRS training

In RICE-CV, common variants are defined as variants with MAF $$ > 0.01$$ in any of the genetically inferred ancestry groups. Let $${\hat{u}}_{{kl}}$$ denote the estimated effect size of the $$k$$-th genetic variant in the $$l$$-th ancestry group, with $${s}_{{kl}}$$ as its standard error. We construct the common variant PRS using several established PRS methods.

For single-ancestry analyses (e.g., in a primarily European population), we employ methods such as CT^11–13^, LDpred2^54^, and Lassosum2^15^, which represent clumping-based, Bayesian, and penalization-based approaches, respectively. For multi-ancestry analyses, we apply CT-SLEB^22^, JointPRS^55^, and PROSPER^25^, covering the same methodological categories. Each method generates multiple PRSs for each individual in the tuning dataset by varying its tuning parameters. Detailed implementations of each method are provided in the “Existing PRS Methods” section.

#### Ensemble learning

The ensemble learning step combines the candidate PRSs to optimize predictive performance. Specifically, we use LASSO and ridge regression as base learners within a generalized linear regression model^54,83^.

For continuous traits, the ensemble model is trained to minimize the cross-validated mean squared error (MSE). The process is as follows: for LASSO regression, we train a model on the tuning dataset using the PRS features as predictors. The model minimizes the objective function:

1 $${\min }_{{w}^{(1)}}\frac{1}{n}\mathop{\sum }\limits_{i=1}^{n}{\left({Y}_{i}-\mathop{\sum }\limits_{j=1}^{J}{w}_{j}^{(1)}{PR}{S}_{{ij}}\right)}^{2}+{\lambda }_{1}\mathop{\sum }\limits_{j=1}^{J}{{|w}}_{j}^{(1)}|$$

where ${Y}_{i}$ is the residualized phenotypes for individual $i$ regressing out for covariates, ${{\rm{PR}}}{{{\rm{S}}}}_{{ij}}$ is the $j$-th candidate PRS for individual $i$, and ${w}^{(1)}={({w}_{1}^{(1)},\ldots,{w}_{J}^{(1)})}^{T}$ is the vector of weights estimated for each PRS. Here, $n$ denotes the tuning data sample size, $J$ represents the total number of candidate PRSs, and ${\lambda }_{1}$ is the LASSO regularization parameter.

For ridge regression, we minimize the objective function:

2 $${\min }_{{w}^{\left(2\right)}}\frac{1}{n}\mathop{\sum }\limits_{i=1}^{n}{\left({Y}_{i}-\mathop{\sum }\limits_{j=1}^{J}{w}_{j}^{\left(2\right)}{{\rm{PR}}}{S}_{{ij}}\right)}^{2}+{\lambda }_{2}\mathop{\sum }\limits_{j=1}^{J}{({w}_{j}^{\left(2\right)})}^{2},$$

where ${w}^{(2)}={({w}_{1}^{(2)},\ldots,{w}_{J}^{(2)})}^{T}$ are the weights estimated for each PRS and ${\lambda }_{2}$ is the ridge regularization parameter.

The optimal regularization parameters ${\lambda }_{1}$ and ${\lambda }_{2}$ are selected by minimizing the cross-validated MSE (default fold = 10) over a default grid of regularization parameters provided in glmnet^84^. With optimized weights for each base learner, we generate predictions for each individual in the tuning dataset as: ${\hat{Y}}_{i}^{(1)}={\sum }_{j=1}^{J}{w}_{j}^{(1)}{{\rm{PR}}}{{{\rm{S}}}}_{{ij}}$ for LASSO and ${\hat{Y}}_{i}^{(2)}={\sum }_{j=1}^{J}{w}_{j}^{(2)}{{\rm{PR}}}{{{\rm{S}}}}_{{ij}}$ for ridge. The ensemble learning algorithm then estimates the optimal weights $\alpha={\left({\alpha }_{1},{\alpha }_{2}\right)}^{T}$ to combine these predictions from the base learners using linear regression. This is done by solving:

3 $${\min }_{\alpha }\frac{1}{n}\mathop{\sum }\limits_{i=1}^{n}{\left({Y}_{i}-\mathop{\sum }\limits_{m=1}^{2}{\alpha }_{m}{\hat{Y}}_{i}^{(m)}\right)}^{2}$$

The final ensemble prediction for individual $i$ is a weighted combination of base learner predictions: ${\hat{Y}}_{i}={\alpha }_{1}{\hat{Y}}_{i}^{(1)}+{\alpha }_{2}{\hat{Y}}_{i}^{(2)}={\sum }_{j=1}^{J}({\alpha }_{1}{w}_{j}^{\left(1\right)}+{\alpha }_{2}{w}_{j}^{\left(2\right)}){{\rm{PR}}}{{{\rm{S}}}}_{{ij}}$.

Similarly for binary traits, the ensemble model combines predictions from LASSO and ridge regression using a generalized linear regression model. For LASSO logistic regression, we minimize the objective function:

4 $$\mathop{\min }_{{w}^{(1)}}-\frac{1}{n}\mathop{\sum }\limits_{i=1}^{n}{\left[{Y}_{i}\log \left({p}_{i}\right)+\left(1-{Y}_{i}\right)\log \left(1-{p}_{i}\right)\right]}^{2}+{\lambda }_{1}\mathop{\sum }\limits_{j=1}^{J}|{w}_{j}^{(1)}|$$

where ${p}_{i}=\sigma ({\sum }_{j=1}^{J}{w}_{j}^{(1)}{{\rm{PR}}}{{{\rm{S}}}}_{{ij}})$ is the predicted probability for individual $i$, with $\sigma \left(x\right)=exp (x)/\{1+exp \left(x\right)\}$ being the logistic function and ${Y}_{i}$ is the binary phenotype (0 or 1) for individual $i$.

For ridge logistic regression, we minimize the objective function:

5 $${\min }_{{w}^{(2)}}-\frac{1}{n}\mathop{\sum }\limits_{i=1}^{n}{\left[{Y}_{i}\log \left({p}_{i}\right)+\left(1-{Y}_{i}\right)\log \left(1-{p}_{i}\right)\right]}^{2}+{\lambda }_{2}\mathop{\sum }\limits_{j=1}^{J}{({w}_{j}^{(2)})}^{2}$$

where ${p}_{i}=\sigma ({\sum }_{j=1}^{J}{w}_{j}^{(2)}{{\rm{PR}}}{{{\rm{S}}}}_{{ij}})$. Optimal regularization parameters ${\lambda }_{1}$ and ${\lambda }_{2}$ are selected by minimizing the cross-validated AUC (default fold = 10). Using the optimal weights for each base learner, the predicted log odds are generated for LASSO and ridge (${\hat{Y}}^{(1)}$ and ${\hat{Y}}^{(2)}$ respectively). The ensemble learning algorithm estimates the optimal weights of the predictions, $\alpha={({\alpha }_{1},{\alpha }_{2})}^{T}$, by minimizing the objective function:

6 $${\min }_{\alpha }-\frac{1}{n}\mathop{\sum }\limits_{i=1}^{n}{\left[{Y}_{i}\log \left({p}_{i}\right)+\left(1-{Y}_{i}\right)\log \left(1-{p}_{i}\right)\right]}^{2}$$

where ${p}_{i}=\sigma ({\sum }_{j=1}^{2}{\alpha }_{j}{\hat{Y}}_{i}^{(j)}).$ The final prediction of the ensemble learning algorithm is then: ${\hat{Y}}_{{{\rm{CV}}}}={\alpha }_{1}{\hat{Y}}^{(1)}+{\alpha }_{2}{\hat{Y}}^{(2)}={\alpha }_{1}{w}^{(1)}\,{{{\rm{PRS}}}}_{{{\rm{CV}}}}+{\alpha }_{2}{w}^{(2)}\,{{{\rm{PRS}}}}_{{{\rm{CV}}}}$, where ${{{\rm{PRS}}}}_{{{\rm{CV}}}}=[{{{\rm{PRS}}}}_{1},{{{\rm{PRS}}}}_{2},\ldots,{{{\rm{PRS}}}}_{J}]$.

### RICE-RV: rare variant PRS modeling

The RICE-RV component focuses on identifying and integrating signals from rare variants into the PRS to enhance genetic risk prediction. It has four main steps: (1) fitting the null model and performing rare variant association testing, (2) modeling significant rare variant sets using penalized regression, and (4) combining PRSs through ensemble learning. Steps 1–2 are based on STAARpipeline focusing on association testing. Steps 3–4 focuses on building the PRS model using the select rare variants sets.

#### Null model and association testing

We first fit a null model using STAARpipeline to adjust for covariates and the predicted common variant PRS from RICE-CV. This step ensures that rare variant signals are independent of common variant effects. For each individual $i$, let ${Y}_{i}$ denote the phenotype of interest, which can be continuous (e.g., height) or binary (e.g., disease status). We model the conditional mean of ${Y}_{i}$ using:

7 $$g\left({\mu }_{i}\right)={\alpha }_{0}+{{Q}_{i}}^{T}\alpha$$

where $g\left({\mu }_{i}\right)$ is the link function (identify for continuous traits, logit for binary traits). ${\alpha }_{0}$ is the intercept term, ${Q}_{i}={({Q}_{i1},\ldots,{Q}_{{iq}})}^{T}$ represent $q$ covariates, such as age, sex, PCs and the common variant PRS from RICE-CV (${\hat{Y}}_{{CV},i}$).

After fitting the null model, we perform rare variant association testing using STAARpipeline, which is specifically designed for large-scale sequencing studies. STAARpipeline adjusts for population structure, corrects for case-control imbalances, and incorporates functional annotations to increase the power of rare variant tests.

Rare variants are grouped into biologically meaningful sets based on functional categories, increasing the power to detect associations by aggregating variants likely to affect gene function. To capture orthogonal signals, RICE-RV uses STAARpipeline to perform association tests on rare variant sets, conditional on the RICE-CV PRS. STAARpipeline defines gene-centric rare variant sets by functional categories. For variants in coding regions, it analyzes five categories per gene: (1) putative loss of function (stop gain, stop loss and splice); (2) missense, (3) disruptive missense, (4) putative loss of function and disruptive missense; and (5) synonymous. For variants in noncoding regions, it tests eight categories: (1) promoter overlaid with CAGE sites, (2) promoter overlaid with DHS sites, (3) enhancer overlaid with CAGE sites, (4) enhancer overlaid with DHS sites, (5) UTR, (6) upstream region, (7) downstream region, and (8) ncRNA. In our study, we use variants in coding regions for WES data and variants in coding and noncoding regions for WGS data.

Within each set, STAARpipeline performs annotation-weighted tests, aggregating information across variants and weighting each variant based on its functional annotation. Multiple test statistics, including burden, SKAT, and ACAT-V, are computed, and an omnibus *p* value is generated using ACAT to combine correlated *p* values. Within RICE-RV, we specifically restrict our identification to the *p* values derived from the annotation-weighted burden tests, referred to as STAAR-B(1,1) *p* values in STAARpipeline to ensure that the identified rare variant signals align with the additive burden-based architecture used in the downstream RICE prediction model. We identify significant rare variant sets as those with STAAR-B (1,1) *p* values less than $p < 1\times {10}^{-3}$.

#### Burden score construction and penalization regression

For each significant rare variant set, we compute a burden score for each individual to summarize the collective effect of the rare variants within that set. Let $K$ denote the total number of significant rare variant sets identified. For the $k$-th rare variant set $(k={\mathrm{1,2}},\ldots,K)$, let ${m}_{k}$ be the number of rare variants in the *k-*th set. For individual $i$, let ${G}_{{ik}}={({G}_{i1k},{G}_{i2k},\ldots,{G}_{i{m}_{k}k})}^{T}$ denote the vector of genotypes for the rare variants within the $k$-th set. Here, ${G}_{{ijk}}$ represents the genotype of individual $i$ for the $j$-th rare variant in set $k$, coded as the number of minor alleles (e.g., 0, 1, or 2). The burden score for individual $i$ for the *k-*th rare variant set is defined as:

9 $${b}_{{ik}}=\mathop{\sum }\limits_{j=1}^{{m}_{k}}{G}_{{ijk}}={G}_{{ik}}^{T}{1}_{{m}_{k}}$$

where ${1}_{{m}_{k}}$ is a vector of ones of length ${m}_{k}$. This burden score ${b}_{{ik}}$ represents the total number of minor alleles carried by individual $i$ across all rare variants in the $k$-th rare variant set.

We then model the relationship between phenotype ${Y}_{i}$ and burden scores using penalized regression methods, specifically LASSO and ridge regression. For continuous traits, we estimate the coefficients $\gamma={\left({\gamma }_{1},{\gamma }_{2},\ldots,{\gamma }_{K}\right)}^{T}$ by minimizing the following objective function:

10 $${\min }_{\gamma }\frac{1}{{n}_{{{\rm{tr}}}}}\mathop{\sum }\limits_{i=1}^{{n}_{{\rm{tr}}}}{\left({Y}_{i}-\mathop{\sum }\limits_{k=1}^{K}{\gamma }_{k}{b}_{{ik}}\right)}^{2}+\lambda P\left(\gamma \right)$$

where ${Y}_{i}$ is the residualized phenotypes regressing out for covariates, ${n}_{{{\rm{tr}}}}$ is the total of number individuals in the training dataset, and $\lambda$ is the regularization parameter controlling the penalty strength. $P\left(\gamma \right)$ is the penalty function (${\sum }_{k=1}^{K}|{\gamma }_{k}|$ for LASSO or ${\sum }_{k=1}^{K}{\gamma }_{k}^{2}$ for ridge regression).

For binary traits, we use a logistic regression framework with penalization, estimating $\gamma$ by minimizing:

11 $${\min }_{\gamma }-\frac{1}{{n}_{{{\rm{tr}}}}}\mathop{\sum }\limits_{i=1}^{{n}_{{{\rm{tr}}}}}{\left[{Y}_{i}\log \left({p}_{i}\right)+\left(1-{Y}_{i}\right)\log \left(1-{p}_{i}\right)\right]}^{2}+\lambda P\left(\gamma \right)$$

where ${p}_{i}=\sigma ({\sum }_{k=1}^{K}{\gamma }_{k}{b}_{{ik}})$ is the predicted probability for individual $i$, with $\sigma \left(x\right)=exp (x)/\{1+exp \left(x\right)\}$ being the logistic function. For fitting the penalized regression models on burden scores, we used the glmnet^84^ package in R (version 4.1.8). The PRS for specific individual $i$ given the estimated $\hat{\gamma }$ under a specific penalization $\lambda$, can be calculated as ${{\rm{PR}}}{{{\rm{S}}}}_{i}={\sum }_{k=1}^{K}{\hat{\gamma }}_{k}{b}_{{ik}}$.

#### Ensemble learning to combine PRSs for RICE-RV

After computing the burden score PRSs, we follow a similar ensemble learning step on the tuning dataset as in RICE-CV to compute the final PRS for RICE-RV. Using a generalized linear regression model, we combine predictions from LASSO and ridge regression to optimizing predictive performance. For rare variants, we select regularization parameters ${\lambda }_{1}$ (for Lasso) and ${\lambda }_{2}$ (for ridge) via a grid search on the full tuning dataset. We use default grid values provided by glmnet^84^ and choose the optimal regularization parameter based on ${R}^{2}$ or AUC. The final PRS for RICE-RV is then denoted as: ${\hat{Y}}_{{{\rm{RV}}}}={{{\rm{PRS}}}}_{{{\rm{RV}}}}$ ${w}_{{{\rm{RV}}}}$, where ${\hat{Y}}_{{RV}}$ is the predicted response from the rare variants. ${{{\rm{PRS}}}}_{{{\rm{RV}}}}=[{{\rm{PR}}}{{{\rm{S}}}}_{1},{{\rm{PR}}}{{{\rm{S}}}}_{2},\ldots,{{\rm{PR}}}{{{\rm{S}}}}_{J}]$ represents the matrix of PRSs generated by based on the burden score, where the $j$-th PRS, ${{\rm{PR}}}{{{\rm{S}}}}_{j}={({{\rm{PR}}}{{{\rm{S}}}}_{1j},..,{{\rm{PR}}}{{{\rm{S}}}}_{{nj}})}^{T}$, corresponds to a PRS derived from a specific penalized regression model for $n$ individuals in the tuning dataset. ${w}_{{{\rm{RV}}}}$ is the vector of optimal weights derived from the ensemble learning model.

### Evaluation of RICE

We evaluated RICE using standardized effect sizes and ${R}^{2}$/AUC. First, to establish the combined RICE score, we model the joint prediction on the tuning dataset. For continuous traits, we fit: $Y={\hat{\theta }}_{1}{\hat{Y}}_{{{\rm{CV}}}}+{\hat{\theta }}_{2}{\hat{Y}}_{{{\rm{RV}}}}$, and for binary traits: ${{\rm{logit}}}(P(Y=1))={\alpha }_{0}+{\hat{\theta }}_{1}{\hat{Y}}_{{{\rm{CV}}}}+{\hat{\theta }}_{2}{\hat{Y}}_{{{\rm{RV}}}}.$ Here ${\hat{Y}}_{{{\rm{CV}}}}$ and ${\hat{Y}}_{{{\rm{RV}}}}$ are the standardized RICE-CV and RICE-RV PRSs, respectively. Crucially, the weights ${\hat{\theta }}_{1}$ and ${\hat{\theta }}_{2}$ are estimated exclusively in the tuning dataset and fixed. These fixed weights are then applied to the validation dataset to compute the final score. ${R}^{2}$ (for continuous traits) and AUC (for binary traits) are calculated in the validation dataset, adjusting for covariates.

Second, to report standardized effect sizes of RICE-CV and RICE-RV, we fit regression models directly in the validation dataset. This step is intended solely to quantify component effect sizes in an independent sample. For continuous traits, we fit linear regression: $Y={\hat{\beta }}_{1}{\hat{Y}}_{{{\rm{CV}}}}+{\hat{\beta }}_{2}{\hat{Y}}_{{{\rm{RV}}}}$. For binary traits, we fit logistic regression ${{\rm{logit}}}(P(Y=1))={\alpha }_{0}+{\hat{\beta }}_{1}{\hat{Y}}_{{{\rm{CV}}}}+{\hat{\beta }}_{2}{\hat{Y}}_{{{\rm{RV}}}}+{Q}_{i}^{T}{\alpha }$, where ${Q}_{i}$ represent other covariates, such as age, sex, PCs. These estimated coefficients ${\hat{\beta }}_{1}$ and ${\hat{\beta }}_{2}$ are the reported standardized effect sizes.

We assessed the significance of the standardized effect size, ${R}^{2}$, and AUC using bootstrap confidence intervals. For the standardized effect-size of RICE-RV, we performed 10,000 bootstrap resamples, and tested whether the coefficient differed significantly from zero at the 95% and 99% confidence levels. For ${R}^{2}$ and AUC, we conducted pairwise comparisons with RICE and the best alternative method for each trait-ancestry combination. Using 10,000 bootstrap resamples, we tested whether the pairwise difference in ${R}^{2}$ or AUC were significantly different from zero at the 95% and 99% confidence levels.

### Existing PRS methods

CT^11–13^ is a method that first removes variants in high LD with an index variant that has the lowest *p* value within a specified base-pair window. From the remaining set of index SNPs, multiple *p* value thresholds are applied to generate PRSs. The optimal PRS and corresponding *p* value threshold are then chosen based on performance in the tuning dataset. We implemented CT using plink1.9^85^ with an LD threshold ${r}^{2}$ of 0.5 (--clump-r2), a window size of 500 kb, and nine *p* value thresholds ranging from $5\times {10}^{-8}$ to 1.

Lassosum2^15^ implements LASSO regression using GWAS summary statistics and LD information. Lassosum2 penalizes effect sizes according to LD by minimizing the objective function: ${\beta }^{T}\left(\left(1-s\right)R+{sI}\right)\beta -{\beta }^{T}r+{\lambda }_{1}{||}\beta |{|}_{1}$, where $\beta$ is the vector of effect sizes, $R$ is the LD matrix, $I$ is the identity matrix, $r$ is the vector of marginal correlations from summary statistics, and $s$ is a tuning parameter to ensure convexity. The parameter ${\lambda }_{1}$ controls ${L}_{1}$ penalty, with$|\left|\beta \right|{|}_{1}$ denote the ${L}_{1}$ norm of $\beta$. We implemented Lassosum2 with 10 different values of $s$ (ranging from 0.5 to 1000) and 30 values of ${\lambda }_{1}$. As in CT, the optimal combination of *s* and *λ*_*1*_ was chosen based on predictive performance in the tuning dataset.

LDpred2^54^ utilizes a Bayesian framework with a spike-and-slab prior for variant effects ${\beta }_{j}$. The prior density is given by ${p}({\beta }_{j}|\pi )=(1-\pi )\delta ({\beta }_{j}=0)+\pi \times N(0,\frac{{h}^{2}}{M\pi })$, where $\delta$ is the Dirac delta function, $\pi$ is the proportion of casual variants, ${h}^{2}$ is the total heritability, $M$ is the total number of variants, and $\pi$ is the proportion of nonzero variants. Posterior effect sizes are estimated using Markov Chain Monte Carlo (MCMC) sampling. We conducted a grid search over 15 values of ${h}^{2}$ (from 0.1 to 1.5) times the heritability estimated by LD score regression, 17 values of $\pi$ ranging from $1\times {10}^{-4}$ to 1, and set the sparse setting to “False”. Both LDpred2 and Lassosum2 were implemented in R using the bigsnpr package (version 1.12.6). The optimal values of ${h}^{2}$ and $\pi$ were selected using the tuning dataset.

CT-SLEB^22^ is a recent multi-ancestry extension of CT method that consists of three steps: two-dimensional CT, calibration of regression coefficients using Empirical Bayes, and ensemble learning. Two-dimensional CT and Empirical Bayes are implemented between pairs of ancestries, designating one as the target population and the other as the reference population. For our analysis of the AoU data, which included three ancestries in the training dataset (AFR, AMR, and EUR), CT-SLEB was implemented twice, treating AFR and AMR as target populations with EUR as the reference population. We further implemented the ensemble learning step on the tuning data to include both sets of PRSs, producing a single prediction for all ancestries. The hyperparameters required for CT-SLEB are identical to those used in CT. We implemented CT-SLEB with eight different *p* value thresholds ranging from $5\times {10}^{-8}$ to 1, two window sizes (50 kb and 100 kb), and six LD threshold (${r}^{2}$ = 0.01, 0.05, 0.1, 0.2, 0.5, 0.8).

JointPRS^55^ is a multi-ancestry PRS method that extends the PRS-CSx^24^ method. JointPRS assumes variant effect size ${\beta }_{j}$ across multiple populations using a correlated Gaussian prior: ${\beta }_{j} \sim N(0,{\psi }_{j}M\Sigma M)$, where $M\Sigma M$ accounts for correlations across ancestries and differences in the sample size, and ${\psi }_{j}$ is a variant-specific hyper-parameter with a hyper-prior ${\psi }_{j} \sim {{\rm{Gamma}}}(1,{\delta }_{j})$ and ${\delta }_{j} \sim {{\rm{Gamma}}}(\frac{1}{2},\phi )$. We implemented JointPRS-auto version, which assumes $\phi \sim {{\rm{Cauchy}}}({\mathrm{0,1}})$. JointPRS-auto requires only GWAS summary statistics from the training data and does not need tuning data for parameter estimation. LD information was provided using the 1000 G reference panel included with the PRS-CSx^24^ software.

PROSPER^25^ is an ensemble penalized regression multi-ancestry PRS method. It involves three steps: (1) performing single-ancestry Lassosum2 to estimate optimal parameters, second, (2) constructing a joint analysis across ancestries using penalized regression, and (3) combining all PRSs from the second step using ensemble learning with SuperLearner. We implemented single ancestry Lassosum2 with the default five values for $s$ and five values for ${\lambda }_{1}$, as described in the Lassosum2 section. The joint multi-ancestry analysis was performed similarly, using default values for the penalty parameters for $\lambda$ and $c$ following PROSPER GitHub guidance. Ensemble regression was performed on the tuning data using SuperLearner.

### Simulation study

We conducted large-scale simulations using simulated traits generated from chromosome 22 of the UKB WES dataset. The training dataset included only individuals with EUR ancestry, with sample sizes of 49,173 or 98,343. The remaining individuals, comprising those with EUR, AFR, AMR, and SAS ancestry, were evenly divided into tuning and validation sets.

Causality was assigned to both randomly selected common variants (MAF > 0.01 in the UKB WES dataset) and to rare variant sets. The proportion of causality varied among three levels: 1%, 5%, and 20%. We explored two different strategies of assigning causality to rare variants within a causal rare variant set. First, all rare variants within a rare variant set are causal and used in the construction of the causal burden score. Second, a proportion (${\pi }_{i} \sim {{\rm{Uniform}}}\left(\right.{\mathrm{0.1,0.9}}$)) of rare variants within causal rare variant set $i$ are used in construction of the causal burden score. The heritability was kept constant across simulations, with the common variant heritability (${h}_{{CV}}^{2}$) to 5% to ensure sufficient statistical power to evaluate model mechanics within a single-chromosomal simulation framework. To reflect realistic genetic architectures, we calibrated the rare variant heritability based on recent empirical estimates of the ratio between burden and common variant heritability (median around 1:12^36^). Accordingly, we set rare variant heritability ${h}_{{RV}}^{2}=0.42\%$. Additionally, we considered two scenarios of negative selection: strong negative selection and no negative selection.

Let ${u}_{{CV},q}$ denote the standardized effect size of the *q*th causal common variant, and ${u}_{{RV},k}$ denote the standardized effect size for the *k-*th causal rare variant burden. Under strong negative selection scenario, the standardized effect sizes were drawn from normal distributions: ${u}_{{{\rm{CV}}},q}\approx N\left(\right.0,\frac{{{h}^{2}}_{{{\rm{CV}}}}}{{C}_{{{\rm{CV}}}}},{u}_{{{\rm{RV}}},k}\approx N(0,\frac{{{h}^{2}}_{{{\rm{RV}}}}}{{C}_{{{\rm{RV}}}}})$, where ${C}_{{{\rm{CV}}}}$ and ${C}_{{{\rm{RV}}}}$ are the number of causal common variants and rare variants, respectively.

Phenotypes were simulated using the following linear model:

12 $${Y}_{i}=\mathop{\sum }_{q=1}^{{C}_{{{\rm{CV}}}}}\frac{{G}_{{iq}}}{\sqrt{{{\hat{\sigma }}^{2}}_{q}}}{u}_{{{\rm{CV}}},q}+\mathop{\sum }\limits_{k=1}^{{C}_{{{\rm{RV}}}}}\frac{{b}_{{ik}}}{\sqrt{{{\hat{\sigma }}^{2}}_{k}}}{u}_{{{\rm{RV}}},k}+{\epsilon }_{i}$$

where ${Y}_{i}$ is the phenotype for individual $i$, ${G}_{{iq}}$ is the genotype of individual $i$ at the *q-*th causal common variant, ${b}_{{ik}}$ is the burden score of individual $i$ for the *k-*th causal rare variant set, and ${\epsilon }_{i}$ is the residual error term. The variance ${{\hat{\sigma }}^{2}}_{q}$ and ${{\hat{\sigma }}^{2}}_{k}$ are defined as follows: ${{\hat{\sigma }}^{2}}_{q}=2{f}_{q}(1-{f}_{q})$, where ${f}_{q}$ is the effect allele frequency for the *q-*th causal common variant, and ${{\hat{\sigma }}^{2}}_{k}$ is calculated similarly for the *k-*th rare variant sets. Under the no negative selection scenario, we set ${{\hat{\sigma }}^{2}}_{q}={{\hat{\sigma }}^{2}}_{k}=1$. Each simulation scenario had 100 simulated traits, and the final presented results is the average of the 100 validation dataset results.

### UKB imputed + WES and WGS analysis

We analyzed the UKB imputed (Field #22828), WES (Field #23156), and WGS (Fields #24304 and #24305) datasets, focusing on participants from four ancestries: AFR, AMR, EUR, and SAS. EAS ancestry was excluded due to limited sample size in the UKB data, which led to unstable performance in the evaluation. Our analyses included six continuous traits: BMI, height, HDL, LDL, log(TG), and TC, and five binary traits: asthma, breast cancer, CAD, prostate cancer, and T2D.

Genetically inferred ancestry groups were defined using the 1000 Genomes Project Phase 3 (1000G) dataset^56^, which includes 2,504 individuals across five ancestry groups: 661 AFR, 347 AMR, 504 EAS, 503 EUR, and 489 SAS. We first performed principal component analysis on all 2,504 samples using plink v2.0^85^, using a set of selected variants provided from gnomAD to capture population structure. Using the first five PCs, we trained a random forest classifier to accurately assign each individual in the 1000 G dataset to their respective ancestry group. Next, we projected the UKB WES, Imputed, and WGS datasets onto this PC space and applied the pre-trained random forest classifier to assign genetically-inferred ancestry groups to each UKB sample based on maximum probability.

Participants were randomly divided into independent training, tuning, and validation sets using stratified sampling within each ancestry, allocating 70%, 15%, and 15% of the total sample size, respectively. Since EUR is the majority ancestry in UKB, we restricted the training dataset to EUR participants, while both EUR and non-EUR participants were included in the tuning and validation sets. Detailed sample sizes for each group are provided in Supplementary Data 2 and 3. Standard quality control procedures were applied to both common and rare variants following previous studies^38,76,86–88^ (Supplementary Note). All analyses, including GWAS, rare variant association testing, and ensemble learning within RICE-CV and RICE-RV, were adjusted for control covariates: the first 10 PCs, sex, age, and age squared. PCs used were the pre-computed ones provided by UKB (Field #22009).

We evaluated PRS prediction performance on the validation set across five methods: CT, LDpred2, Lassosum2, RICE-CV, and RICE-RV. Optimal hyperparameters for CT, LDpred2, and Lassosum2 were chosen based on performance in the tuning dataset. Both phenotypes and PRSs were standardized to have mean 0 and variance 1 within each ancestry, as described in the Phenotype and PRS Standardization section. Performance of the standardized PRSs was assessed by fitting a linear regression model between the standardized phenotype and standardized PRS. For binary traits, PRS standardization followed the same procedure as for continuous traits, and prediction performance was evaluated using logistic regression with control covariates.

For the CT, LDpred2, and Lassosum2 methods for common variant PRS construction, LD reference panels were estimated from 3,000 individuals randomly sampled from the training dataset for both Imputed and WGS analyses. For LDpred2 and Lassosum2 in the imputed data analysis, LD was estimated using variants included in the HapMap3 (HM3) and Multi-Ethnic Genotyping Array (MEGA). In contrast, for the WGS analysis, computational constraints restricted LDpred2 and Lassosum2 to the subset of variants included in the HM3 array. CT was implemented using all variants available in the reference panel without restriction. For rare variant PRS construction, the STAARpipeline differed based on sequencing data: for the WES data (used in the Imputed + WES configuration), we analyzed rare variants located in the coding region of protein-coding genes. For the WGS data, we included rare variants located in both coding and noncoding regions of protein-coding genes.

### AoU analysis

We analyzed the AoU dataset using WGS data version 7.1. For common variants, we used the Allele Count/Allele Frequency (ACAF) Threshold callset provided in the AoU Researcher Workbench, which include variants with MAF > 0.01 or allele count > 100 in any of the computed ancestry groups. Given that the training dataset included only AFR, AMR, and EUR ancestries, we further restricted common variants to those with MAF > 0.01 in any of these three ancestries. For rare variants, we focused on exonic regions using the “Exome” callset from the AoU Researcher Workbench. We employed the genetically inferred ancestry groups provided by AoU, based on variants from gnomAD^69^, the Human Genome Diversity Project^89^, and 1000G^56^. The ancestries included in our analysis were AFR, AMR, EAS, MID, EUR, and SAS. We examined six continuous traits: BMI, height, HDL, LDL, log(TG), and TC. Genetically inferred ancestry groups (EUR, EAS, SAS, AMR, MID, AFR) were directly provided by the All of Us Research Program^40^. For AoU, we computed PCs based on pre-computed PCA loadings calculated from the samples of the 1000G reference panel.

Data were randomly split using stratified sampling within ancestries into training, tuning, and validation, comprising 70%, 15%, and 15% of total sample size, respectively. Since AFR, AMR, and EUR are the majority ancestries in the AoU database, we included only these three ancestries in the training dataset. For the tuning and validation datasets, we included all available ancestries in AoU. The detailed sample size breakdown is provided in Supplementary Data 5. Both common and rare variants underwent standard quality control procedures (Supplementary Note). All analyses, including GWAS, rare variant association testing, and ensemble learning within RICE-CV and RICE-RV, were adjusted for control covariates: the first 10 PCs, sex, age, and age squared.

We compared PRS prediction performance on the validation set across five methods: CT-SLEB, JointPRS, PROSPER, RICE-CV, and RICE-RV. Following a similar procedure as in the UKB analyses, we standardized the phenotypes and PRSs to have a mean of 0 and variance of 1 within each ancestry. The detailed standardization procedure is provided in the Phenotype and PRS Standardization section. The performance of the standardized PRSs was assessed by fitting a linear regression model between the standardized phenotype and the standardized PRS.

LD reference data for JointPRS and PROSPER was publicly provided and built using the 1000G reference data. Specifically, the provided LD for PROSPER was constrained to the HM3 + MEGA array, while the provided LD for JointPRS was constrained to the HM3 array. Consequently, our analyses using these methods were limited to these specific variant subsets. In contrast, for CT-SLEB, the LD reference was derived directly from a random subset of 3000 individuals from each ancestry (AFR, AMR and EUR) in the training dataset. This approach allowed us to implement CT-SLEB on the full set of available common variants. RICE-RV was implemented using rare variants located in coding regions of protein-coding genes utilizing STAARpipeline.

### Cross-dataset portability analysis

To evaluate PRS portability, we applied AoU-trained models (using AFR, AMR, and EUR ancestries for training, as described above) to the UKB validation dataset. Common variants were scored using UKB imputed genotypes, and rare variants using UKB WES data, with the same quality control and covariate adjustments (top 10 PCs, sex, age, age squared). RICE models were trained using all available variants in the AoU dataset. When applying these models to the UKB validation dataset, we utilized the subset of variants present in both datasets (details on variant counts in Supplementary Data 9). Performance was assessed on the six shared continuous traits (BMI, height, HDL, LDL, log(TG), TC) across AFR, AMR, EUR, and SAS ancestries, using the standardized effect size, *R*^*2*^, and bootstrap significance (10,000 resamples) as in other evaluations.

### Reporting summary

Further information on research design is available in the Nature Portfolio Reporting Summary linked to this article.

## Supplementary information

Supplementary Figures and Supplementary Notes

Description of Additional Supplementary Files

Supplementary Data

Reporting Summary

Transparent Peer Review file

## Source data

Source Data

## Data Availability

UK Biobank phenotype data, WES data (Field #23156), imputed data (Field #22828), and WGS data (Fields #24304 and #24305) can be accessed through the UK Biobank research analysis platform [https://ukbiobank.dnanexus.com/landing]. All data used in this research are publicly available to registered researchers through the UKB data-access protocol and who are listed as collaborators on UKB-approved access applications. All of Us phenotype data, WES data, and WGS data can be accessed through the All of Us research workbench [https://workbench.researchallofus.org/login] (version 7.1). All data used in this research are publicly available to registered researchers with controlled tier access through the All of Us data-access protocol. Data generated in this study are largely available in the Supplementary Data or Source Data files. Large results generated in this study, including common-variant GWAS summary statistics, rare-variant association test summary statistics, and the RICE-CV and RICE-RV model weight files, are available through a Harvard Dataverse dataset^90^. Source data are provided with this paper.

## Code availability

Simulation and data analyses code are archived on Zenodo (v1.0.0^91^). The corresponding GitHub repository is https://github.com/jwilliams10/RareVariantPRS. Software and tutorial to implement RICE are archived on Zenodo (v1.0.0^92^) and available on GitHub [https://github.com/jwilliams10/RICE].
