# Supplementary material for: Integrating common and rare variants improves polygenic risk prediction across diverse populations
Source: Nat Commun. 2026 Apr 24;17:5772. doi: 10.1038/s41467-026-72185-2 (PMC13323733; doi:10.1038/s41467-026-72185-2)
Supplement: Supplementary file 1 — Supplementary Figures and Supplementary Notes [file 41467_2026_72185_MOESM1_ESM.pdf]

Supplementary Figures and Supplementary Note

Table of Contents

Supplementary Figures 3

Supplementary Figure 1. Simulation results comparing the predictive performance of PRSs for four ancestral groups from the UK Biobank (UKB) 3

Supplementary Figure 2. Average heritability of rare variant burden scores for each ancestry by simulation design 12

Supplementary Figure 3. Manhattan and QQ plots for UKB Imputed + WES 14

Supplementary Figure 4. Rare variant QQ plots for UKB Imputed + WES 18

Supplementary Figure 5. Manhattan and QQ plots for UKB WGS 20

Supplementary Figure 6. Rare variant QQ plots for UKB WGS 24

Supplementary Figure 7. Predictive performance of ancestry-adjusted PRSs for 11 traits across four ancestral groups from UK Biobank (UKB) imputed + whole exome sequencing data (WES) data 27

Supplementary Figure 8. Relationship between common and rare variant PRSs and standardized traits across four ancestral groups from UK Biobank (UKB) imputed + whole-exome sequencing (WES) data 29

Supplementary Figure 9. Joint stratification of common and rare variant PRS for standardized traits across four ancestral groups from UK Biobank (UKB) imputed + whole-exome sequencing (WES) data 35

Supplementary Figure 10. Predictive performance of RICE-RV constructed using high-penetrance genes 41

Supplementary Figure 11. Predictive performance of RICE-RV constructed using different p-value thresholds 42

Supplementary Figure 12. Predictive performance of ancestry-adjusted PRSs for 11 traits across four ancestral groups from UK Biobank (UKB) whole genome sequencing data (WGS) data 43

Supplementary Figure 13. Relationship between common and rare variant PRSs and standardized traits across four ancestral groups from UK Biobank (UKB) whole-genome sequencing (WGS) data 46

Supplementary Figure 14. Predictive performance of PRSs standardized within genetically-inferred ancestries or using the first five principal components (Methods) for

six continuous traits and five binary traits across four ancestral groups from UK Biobank (UKB) whole-genome sequencing (WGS) data 52

Supplementary Figure 15. Comparison of ancestry-adjusted PRSs from RICE-CV and RICE-RV using UK Biobank (UKB)) imputed genotype and WES, and whole-genome sequencing (WGS) data for individuals of either African, Admixed American, or South Asian ancestry 56

Supplementary Figure 16. Comparison of ancestry-adjusted PRSs from RICE-CV, RICE-RV, RICE-RV constructed with only coding genes (Coding), and RICE-RV constructed using only noncoding genes (Noncoding) using UK Biobank (UKB) whole-genome sequencing (WGS) data for individuals of either African, Admixed American, or South Asian ancestry 58

Supplementary Figure 17. Manhattan and QQ plots for All of Us 60

Supplementary Figure 18. Rare variant QQ plots for All of Us 67

Supplementary Figure 19. Predictive performance of ancestry-adjusted PRSs for six continuous traits across six ancestral groups from All of Us (AoU) data 68

Supplementary Figure 20. Relationship between common and rare variant PRSs and standardized traits across six ancestral groups from All of Us (AoU) data 69

Supplementary Figure 21. Assessing the prediction performance of RICE trained on All of Us (AoU) and validated on UK Biobank Imputed + whole-exome sequencing (WES) data 75

Supplementary Note 76

Supplementary Figures

Supplementary Figure 1. Simulation results comparing the predictive performance of PRSs for four ancestral groups from the UK Biobank (UKB). The training data had a sample size of N = 49,173 (Supp. Fig. 1a and 1c) or N = 98,343 (Supp. Fig. 1b) with only individuals of European ancestry (EUR), while the tuning (N = 20,869) and validation datasets (N = 20,868) contained individuals of African (AFR), Admixed American or Latino (AMR), European (EUR), and South Asian (SAS) ancestries (**Supplementary Data 1**). Simulations assumed a common variant heritability of 0.05 and a rare variant set heritability of 4.17×10^{-3}, under the assumption of either strong negative selection (Supp. Fig. 1a and 1b) or no negative selection effect size distribution (Supp. Fig. 1c) (**Methods**). Causal proportions for both common variants and rare variant sets varied across three levels: 0.01 (top), 0.05 (middle), and 0.2 (bottom). Lastly, rare variants within causal rare variant sets were either all assigned as causal (Supp. Fig. 1a. – d.) or a proportion of them were assigned as causal (proportion $\sim Uniform(0.2, 0.9)$, Supp. Fig. 1e. – h.). Data were generated using unrelated individuals from UK Biobank whole-exome sequencing data (WES), with simulation based on chromosome 22. For each simulation scenario, 100 simulated traits were generated and results shown are the mean across the 100 validation-set evaluations. In the first figure, PRS performance is reported as the "Beta of PRS per standard deviation (SD)", derived from the regression model $Y \sim PRS \times \beta$, with β representing the effect of standardized PRS on the standardized outcome (**Methods**). For RICE, the model used was $Y \sim PRS_{CV} \times \beta_{CV} + PRS_{RV} \times \beta_{RV}$. Beta values can be interpreted as the square root of the heritability ($\sqrt{h^2}$) of the outcome explained by the PRS (**Supplementary Note**). Significance of β_{RV} is assessed using percentile bootstrap confidence intervals (10,000 resamples), testing the two-sided alternative $H_A : \beta_{RV} \neq 0$, *** indicates the lower bound of the 99% bootstrap CI > 0 and ** indicates the lower bound of the 95% bootstrap CI > 0. Exact bootstrap p-values and CI bounds are provided in the Source Data file. Source data are provided as a Source Data file. Source data are provided as a Source Data file. In the second figure, PRS performance is evaluated with R^2 derived from the regression model $Y \sim PRS \times \beta$, with β representing the effect of standardized PRS on the standardized outcome (**Methods**). For RICE, R^2 is derived using a predicted PRS from a linear model containing both RICE-CV and RICE-RV trained on the tuning dataset. Significance of R^2 was assessed using 10,000 bootstrap resamples of the validation set, testing whether the pairwise difference ${R^2}_{RICE} - {R^2}_{Best\ Alternative}$ (per trait–ancestry pair) differs from 0 ($p < 0.05$, **; $p < 0.01$, ***; exact p-values in Source Data). Source data are provided as a Source Data file.

a) Training dataset consisting of 49,173 individuals of European Ancestry, data simulated under a strong negative selection model, and all rare variants within a causal rare variant set are assigned to be causal.

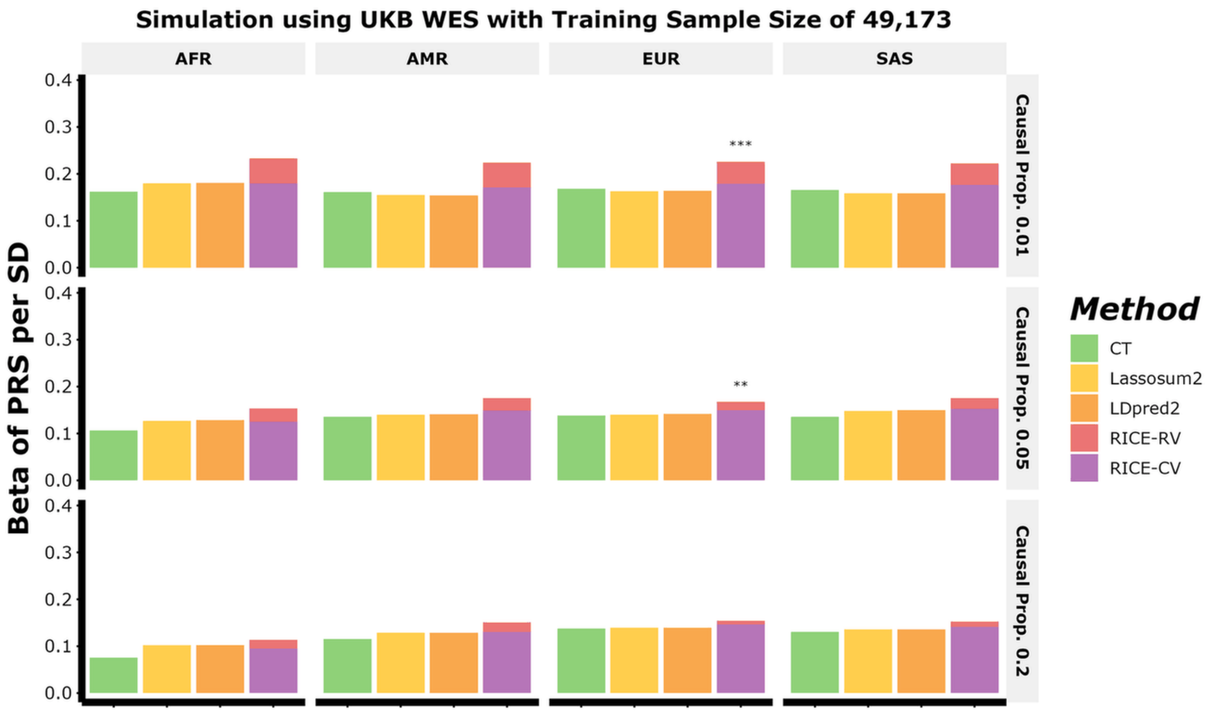

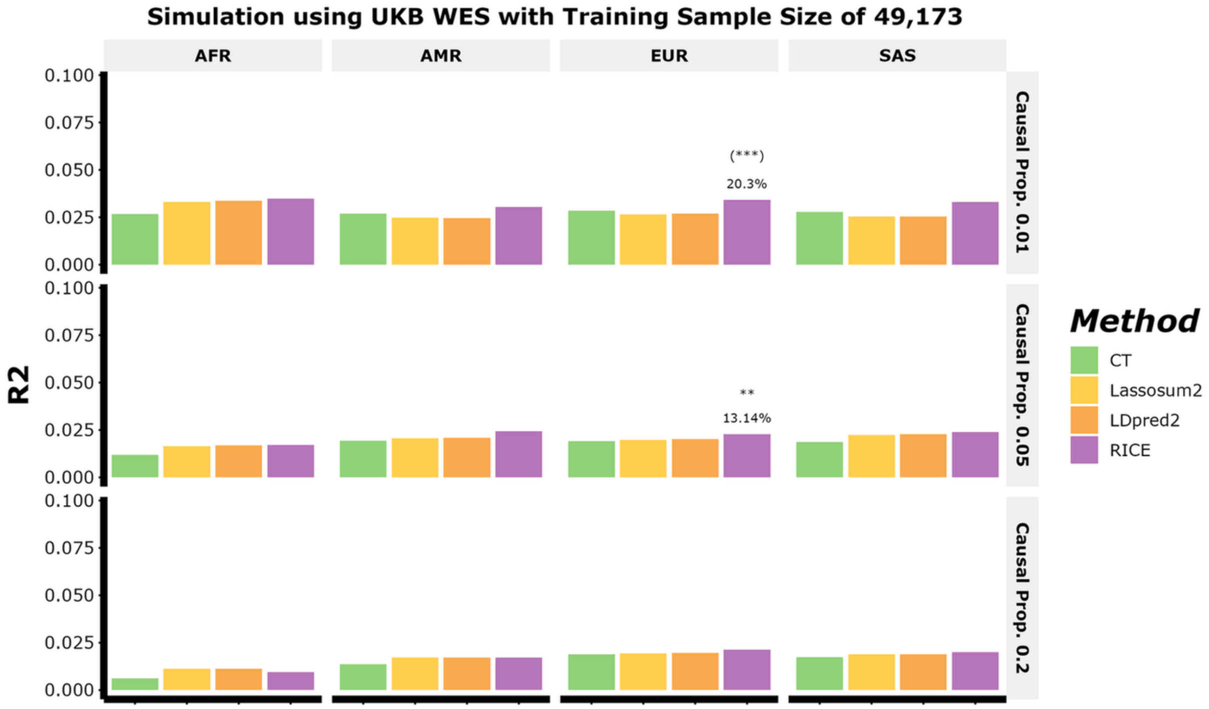

Supplementary Figure 1 continued: b) Training dataset consisting of 98,343 individuals of European Ancestry, data simulated under a strong negative selection model, and all rare variants within a causal rare variant set are assigned to be causal.

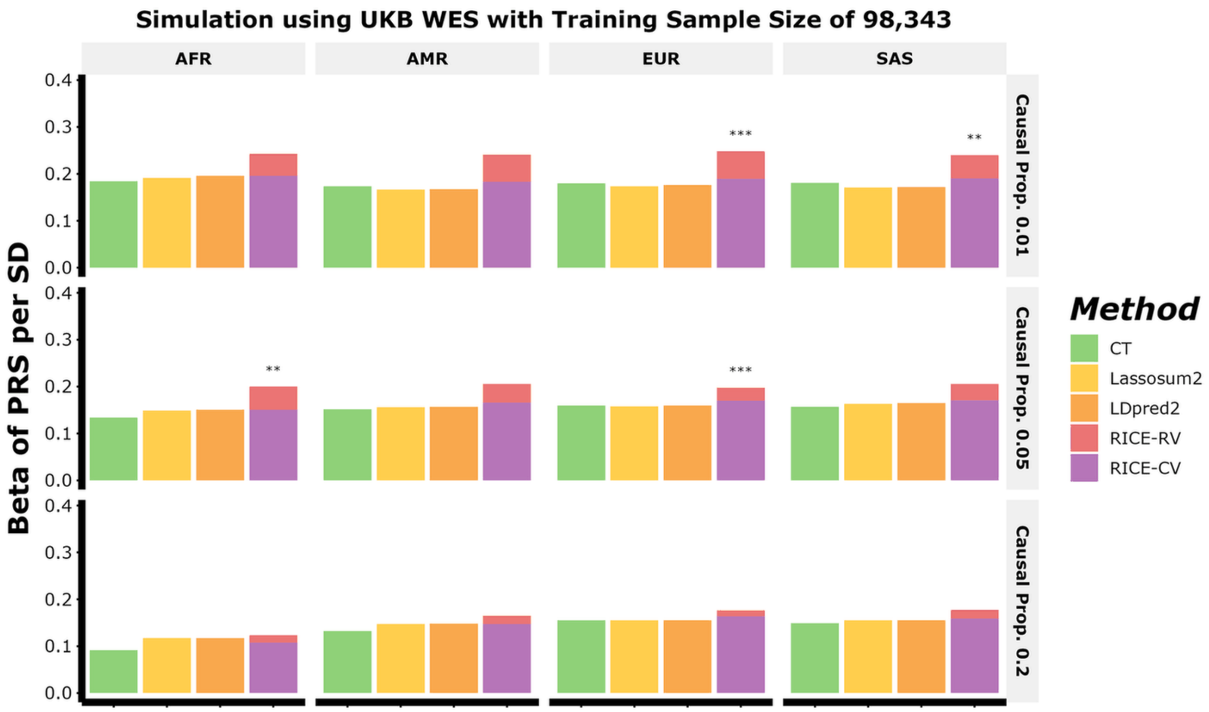

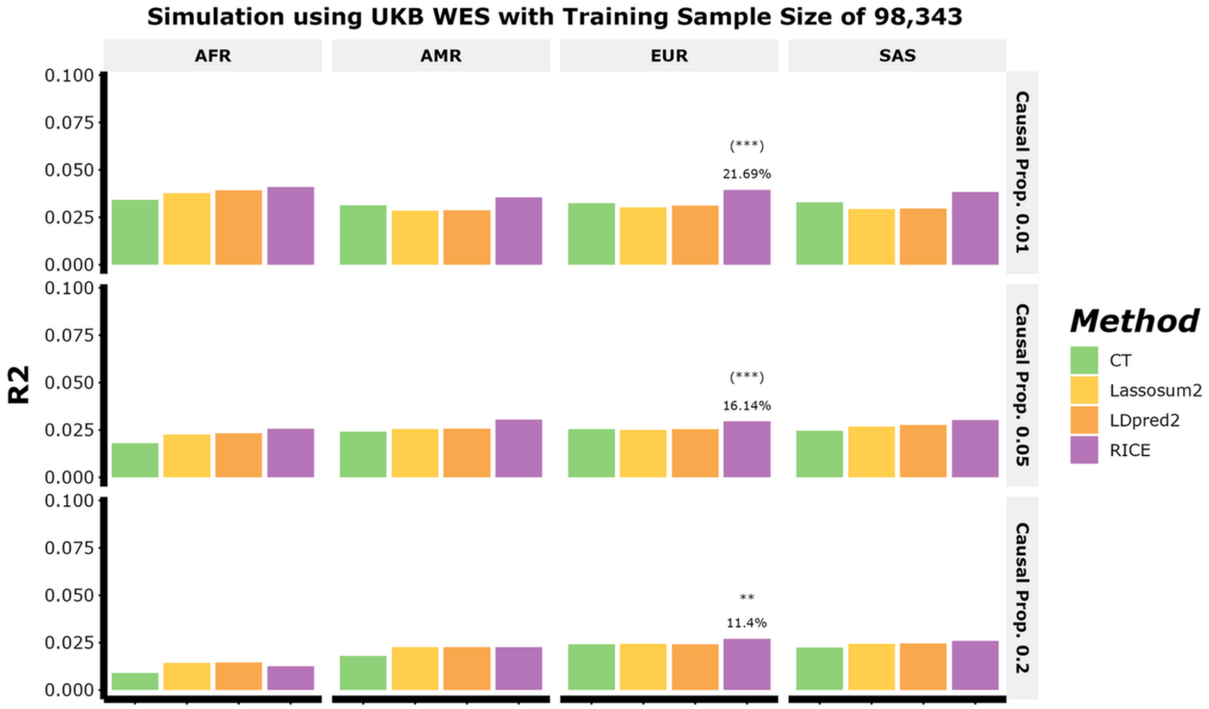

Supplementary Figure 1 continued: c) Training dataset consisting of 49,173 individuals of European Ancestry, data simulated under no negative selection, and all rare variants within a causal rare variant set are assigned to be causal.

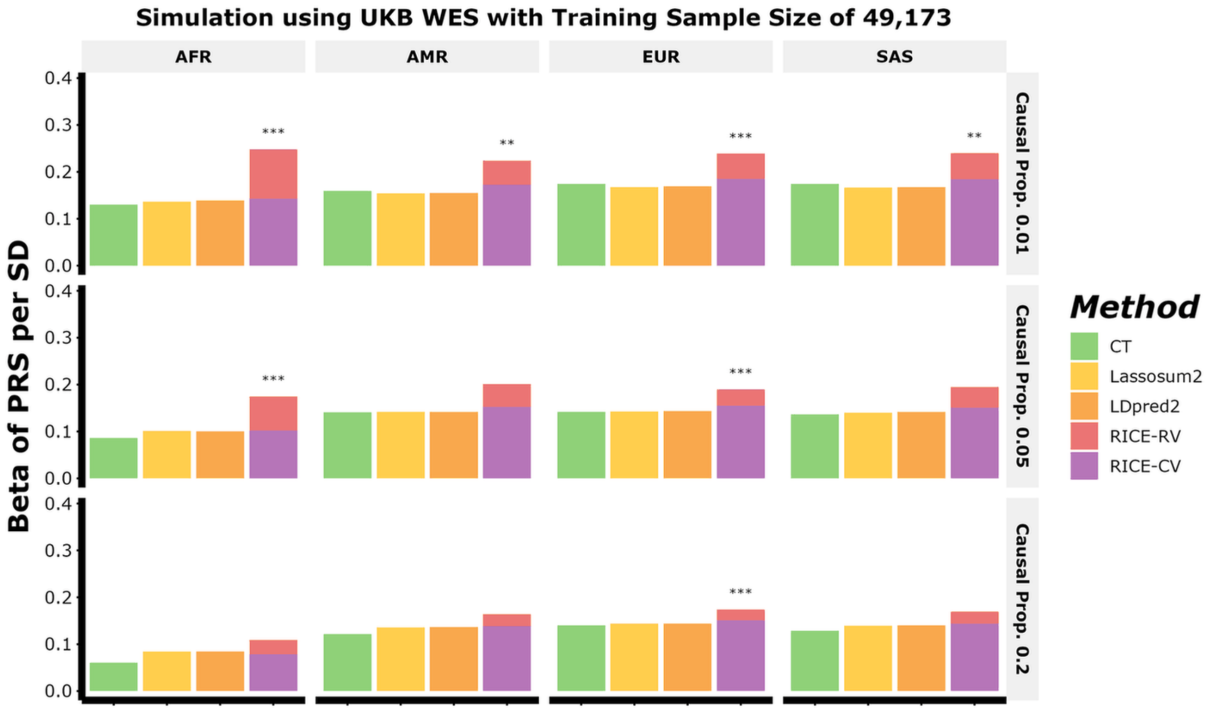

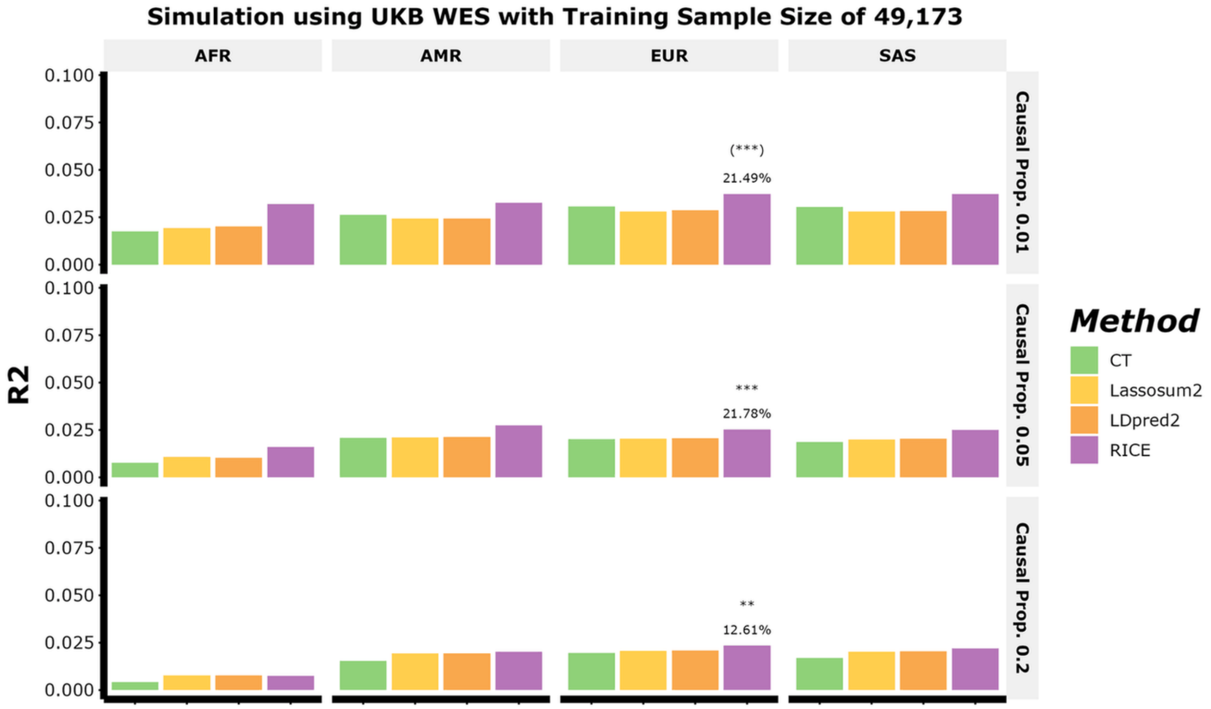

Supplementary Figure 1 continued: d) Training dataset consisting of 98,343 individuals of European Ancestry and data simulated under no negative selection, and all rare variants within a causal rare variant set are assigned to be causal.

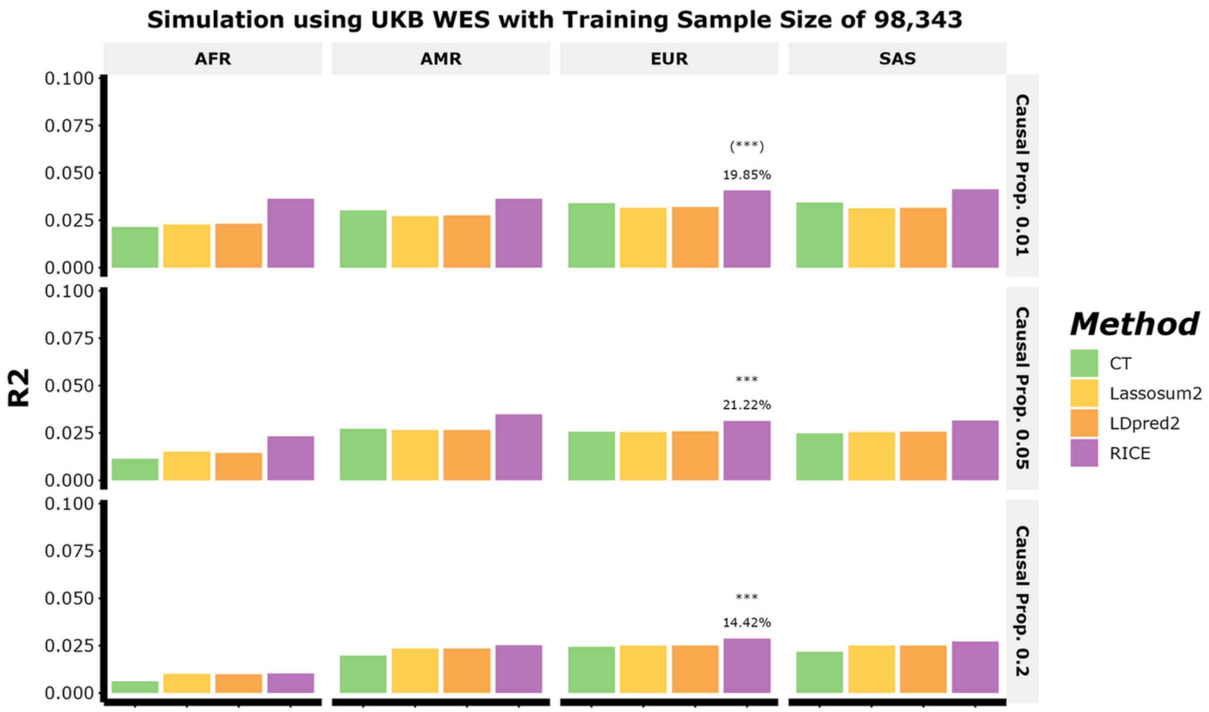

Supplementary Figure 1 continued: e) Training dataset consisting of 49,173 individuals of European Ancestry, data simulated under a strong negative selection model, and a proportion of rare variants within a causal rare variant set are assigned to be causal.

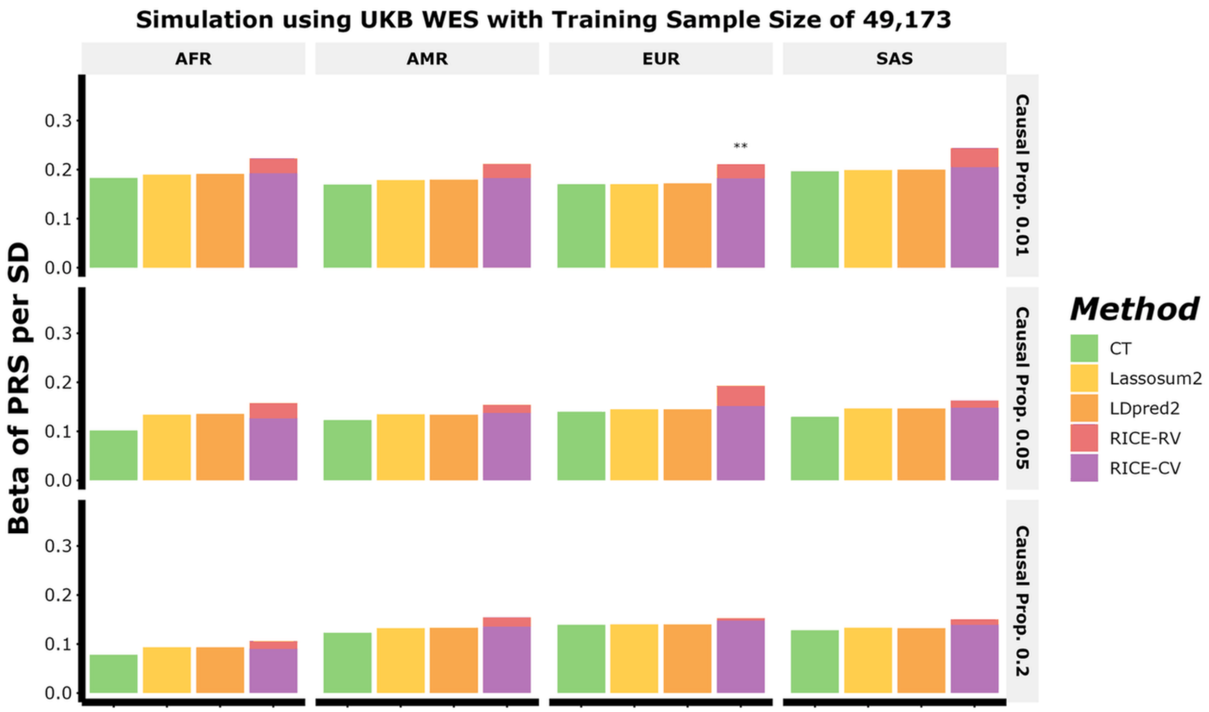

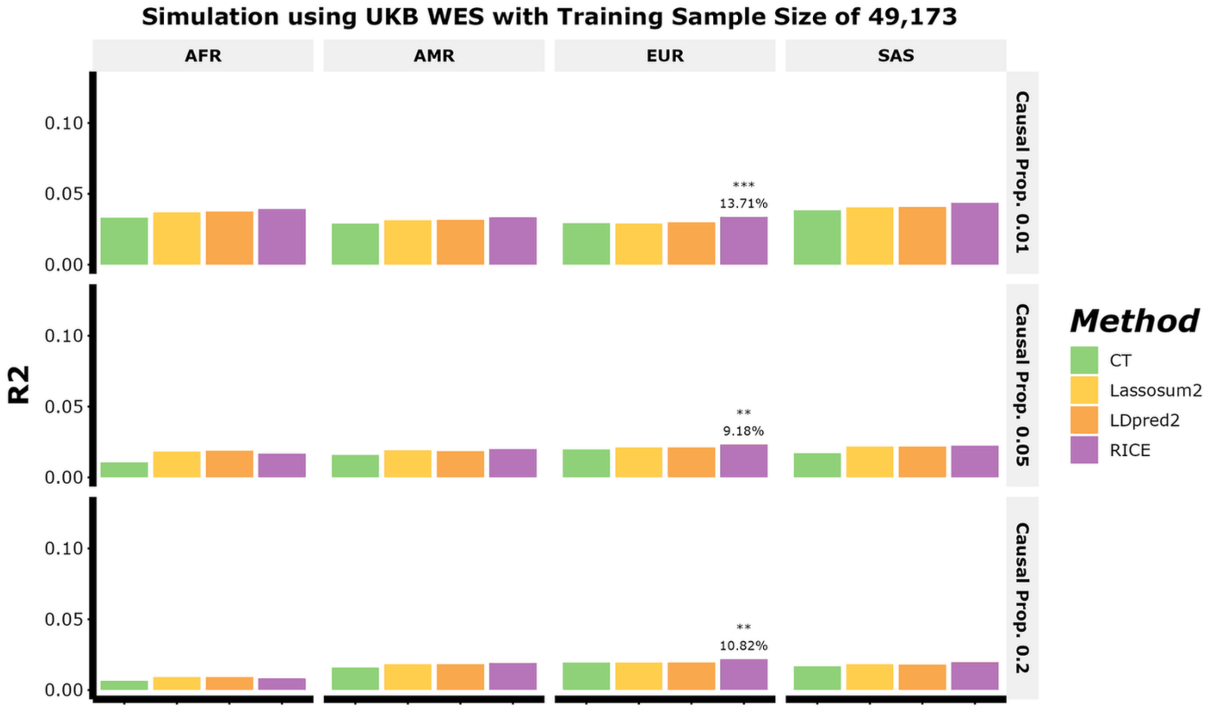

Supplementary Figure 1 continued: f) Training dataset consisting of 98,343 individuals of European Ancestry, data simulated under a strong negative selection model, and a proportion of rare variants within a causal rare variant set are assigned to be causal.

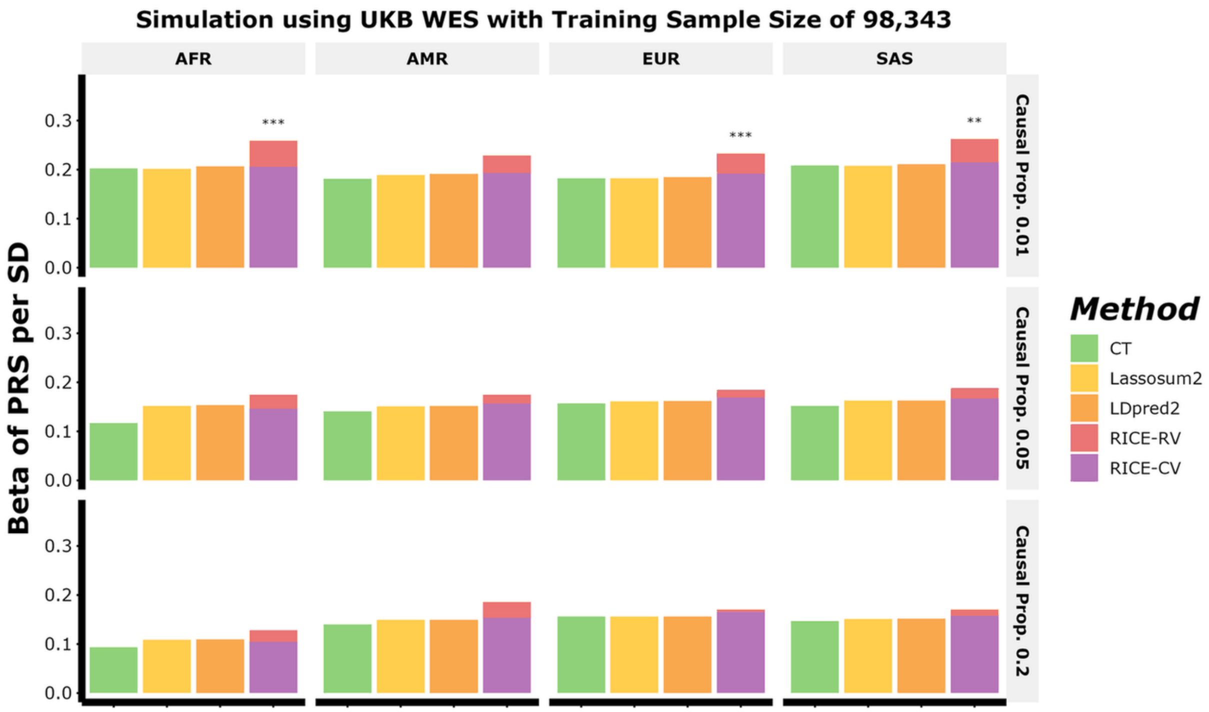

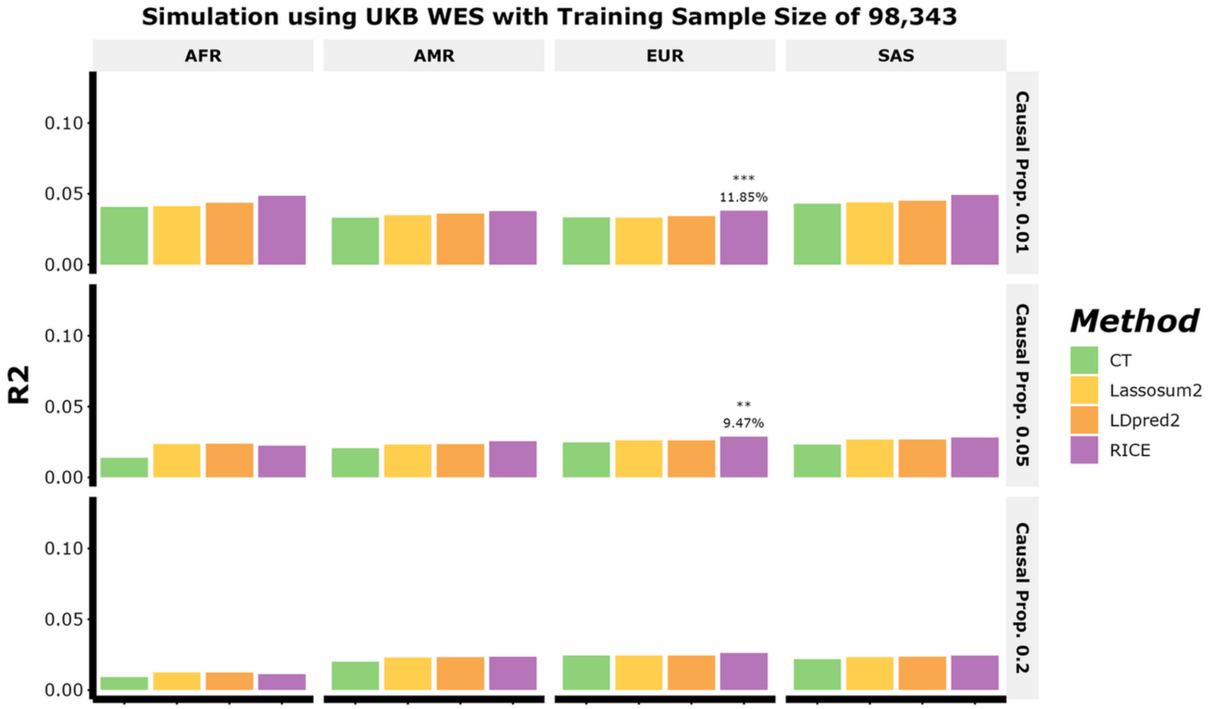

Supplementary Figure 1 continued: g) Training dataset consisting of 49,173 individuals of European Ancestry, data simulated under no negative selection, and a proportion of rare variants within a causal rare variant set are assigned to be causal.

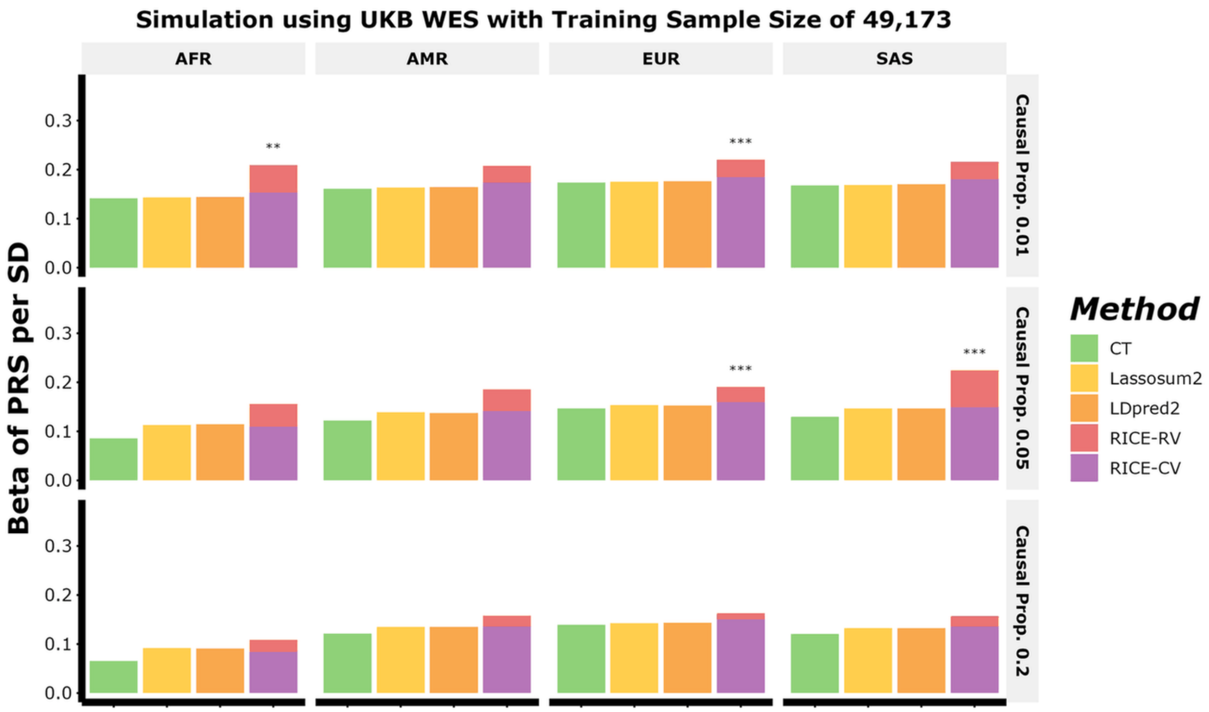

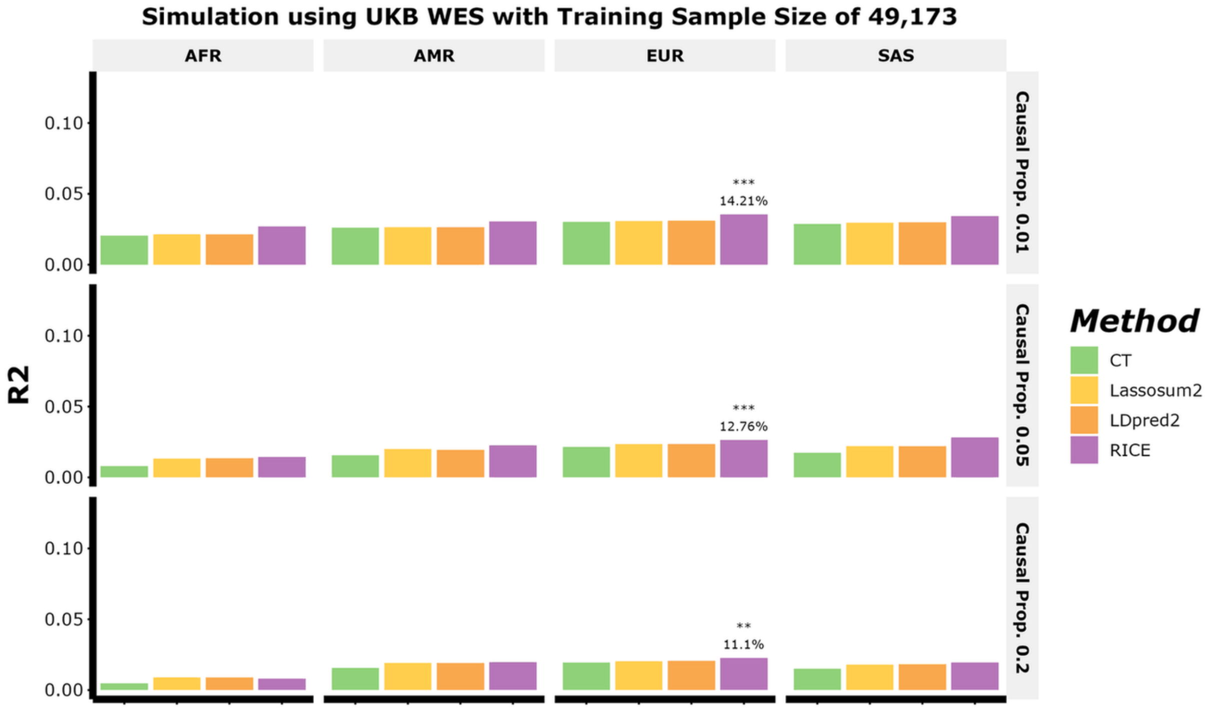

Supplementary Figure 1 continued: h) Training dataset consisting of 98,343 individuals of European Ancestry, data simulated under no negative selection, and a proportion of rare variants within a causal rare variant set are assigned to be causal.

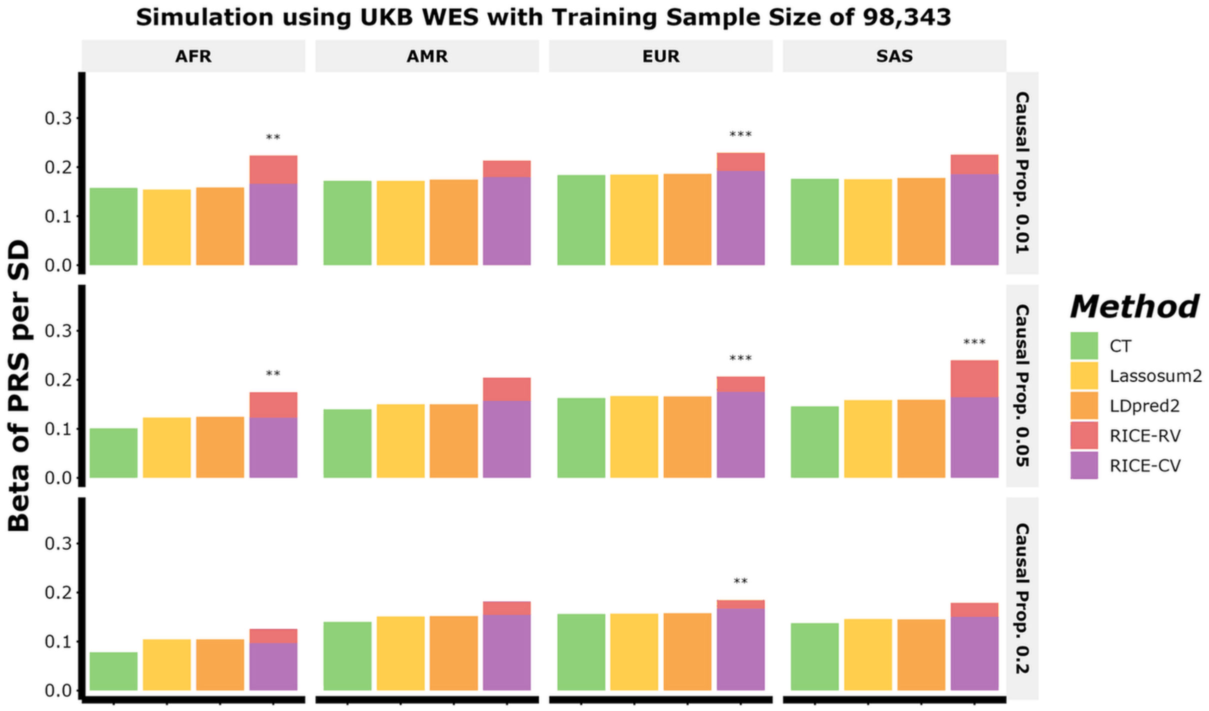

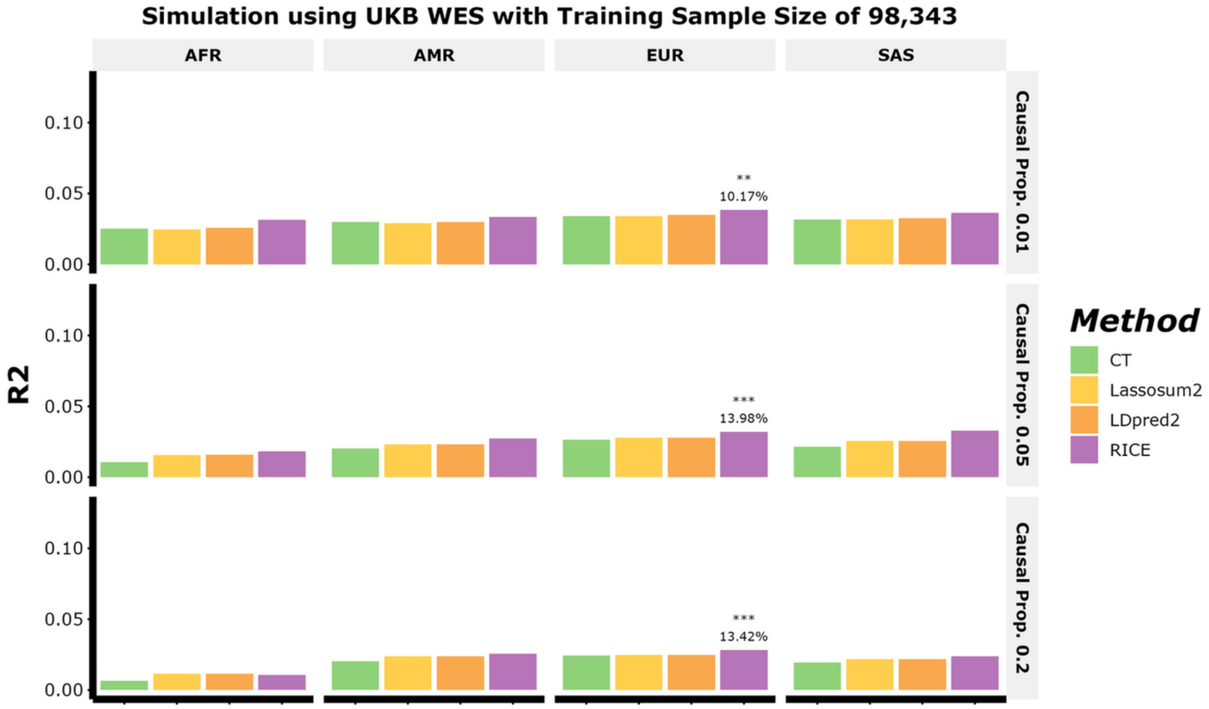

Supplementary Figure 2. Average heritability of rare variant burden scores for each ancestry by simulation design. Simulations assumed a common variant heritability of 0.05 and a rare variant set heritability of 4.17×10^{-3}. Causal proportions for both common variants and rare variant sets varied across three levels: 0.01 (top), 0.05 (middle), and 0.2 (bottom). Data was simulated under both strong negative selection (Scaled: Yes) and no negative selection (Scaled: No). Rare variants within causal rare variant sets were either all assigned as causal (Supp. Fig. 2a.) or a proportion of them were assigned as causal (proportion $\sim \text{Uniform}(0.2{,}0.9)$, Supp. Fig. 2b.). Data were generated using unrelated individuals from UK Biobank whole-exome sequencing data (WES), with simulation based on chromosome 22. For each simulation scenario, 100 simulated traits were generated and results shown are the mean across the 100 simulated outcomes. Data is plotted by ancestry; African (AFR), Admixed American or Latino (AMR), European (EUR), and South Asian (SAS). Source data are provided as a Source Data file.

a) Average heritability of rare variant burden scores for multiple simulation designs assuming all rare variants within causal rare variant sets are causal.

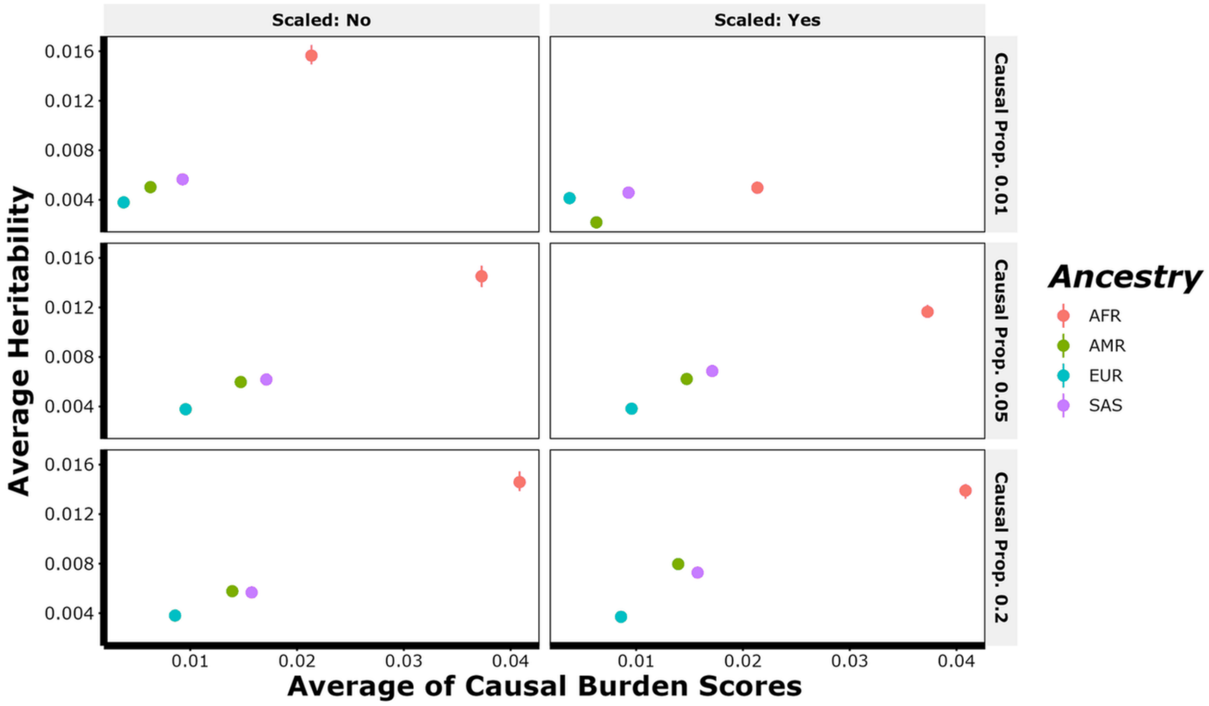

Supplementary Figure 2 continued: b) Average heritability of rare variant burden scores for multiple simulation designs assuming a proportion of rare variants within causal rare variant sets are causal.

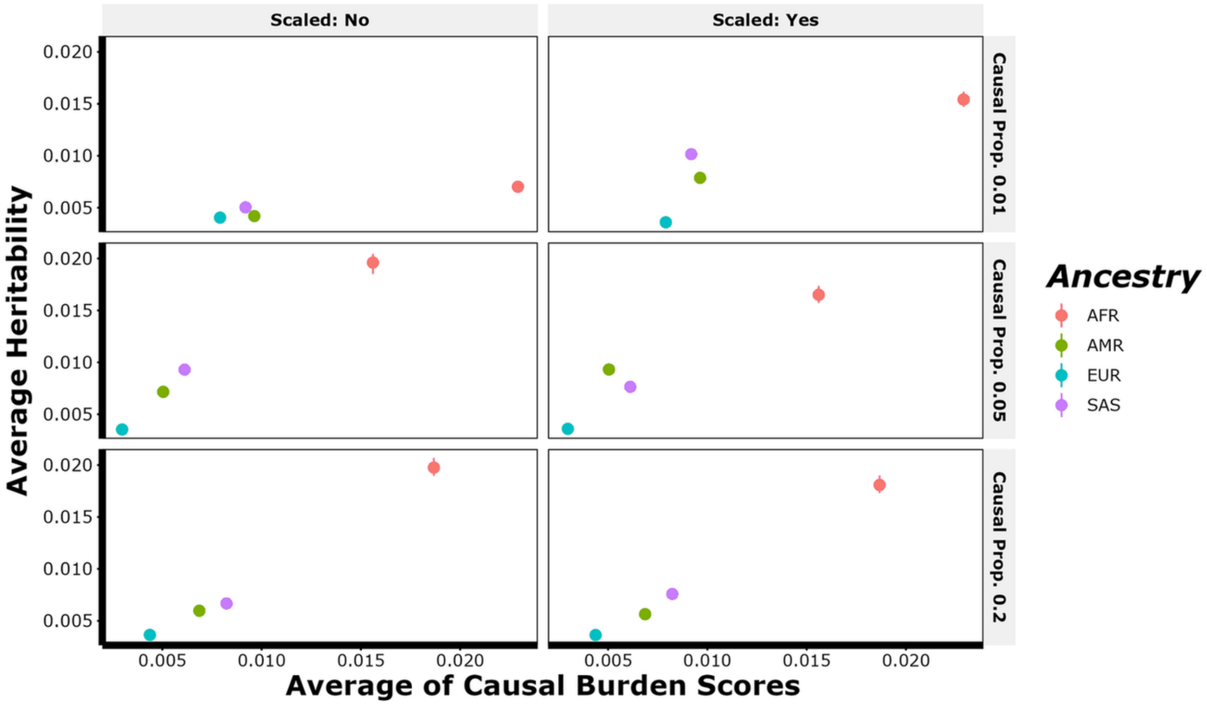

Supplementary Figure 3. Manhattan plot and QQ plots based on the UK Biobank imputed + whole exome sequencing (WES) GWAS summary statistics computed using the training set consisting of only individuals of European ancestry (EUR) for five binary traits: asthma, breast cancer, coronary artery disease (CAD), prostate cancer, and type 2 diabetes (T2D) and six continuous traits: body mass index (BMI), high-density lipoprotein cholesterol (HDL), height, low-density lipoprotein cholesterol (LDL), natural logarithm of triglycerides (log(TG)), and total cholesterol (TC). The red and blue shaded regions around the diagonal line in the QQ plots indicate the 95% confidence intervals expected under the null hypothesis of no association between genetic variants and the trait of interest, for minor allele frequencies (MAF) within the ranges (0.05, 0.5] and [0.01, 0.05], respectively. Under the null hypothesis, the p-value follows a $\mathrm{Uniform}(0,1)$ distribution. The jth order statistic follows a $\mathrm{Beta}(j, N-j+1)$ distribution, where N is the total number of variants given a specific MAF cutoff. For binary traits, λ_{1000} scales λ to a study with 1000 cases and 1000 controls using $\lambda_{1000} = 1 + 1000 \times (\lambda - 1) \times \left(\frac{1}{N_{\mathrm{case}}} + \frac{1}{N_{\mathrm{control}}}\right)$. Genomic control factors are shown in **Supplementary Data 4**. Source data are provided as a Source Data file.

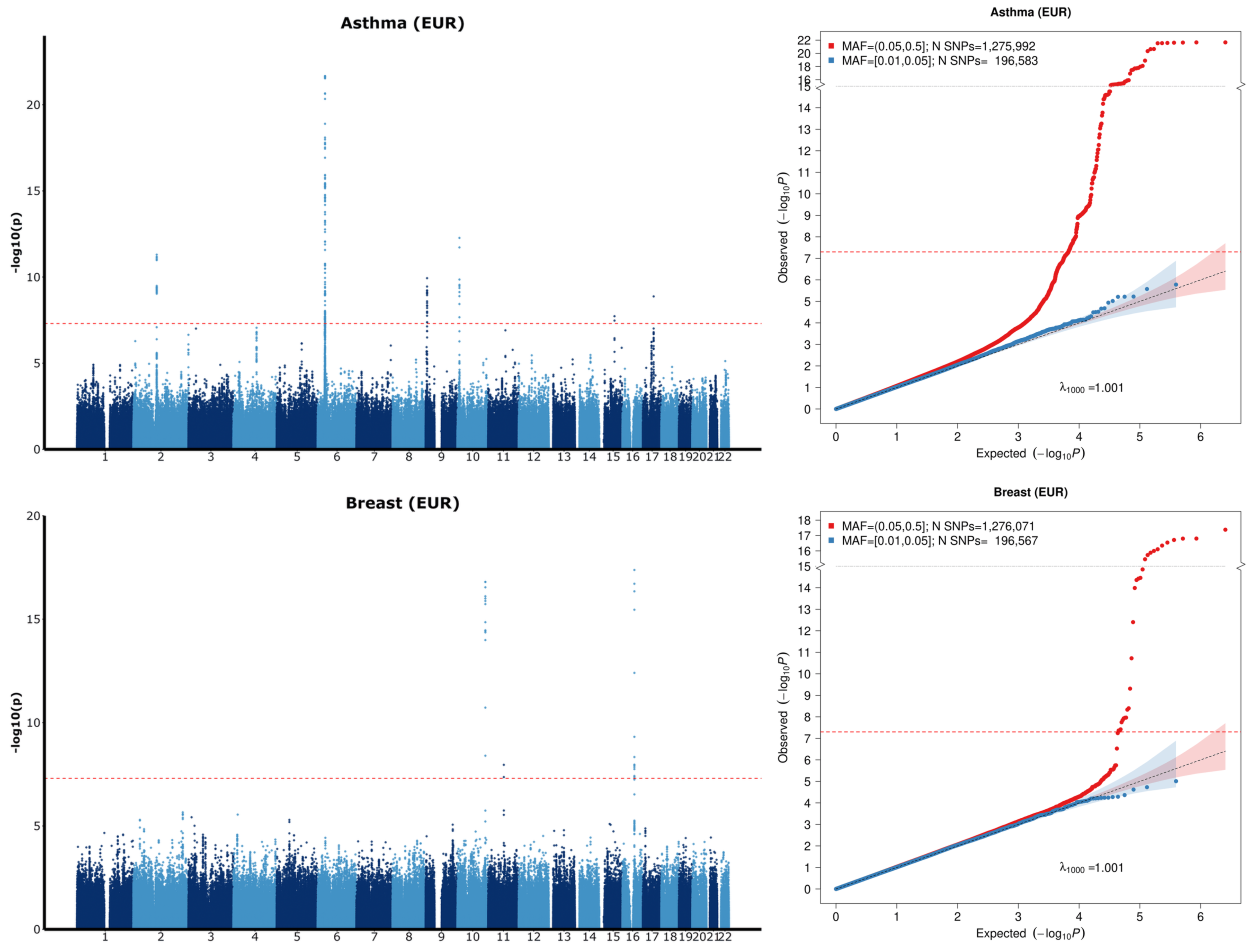

Supplementary Figure 3 continued: Manhattan and QQ Plots for CAD, prostate cancer, and T2D based on UK Biobank Imputed + WES GWAS summary statistics in European populations.

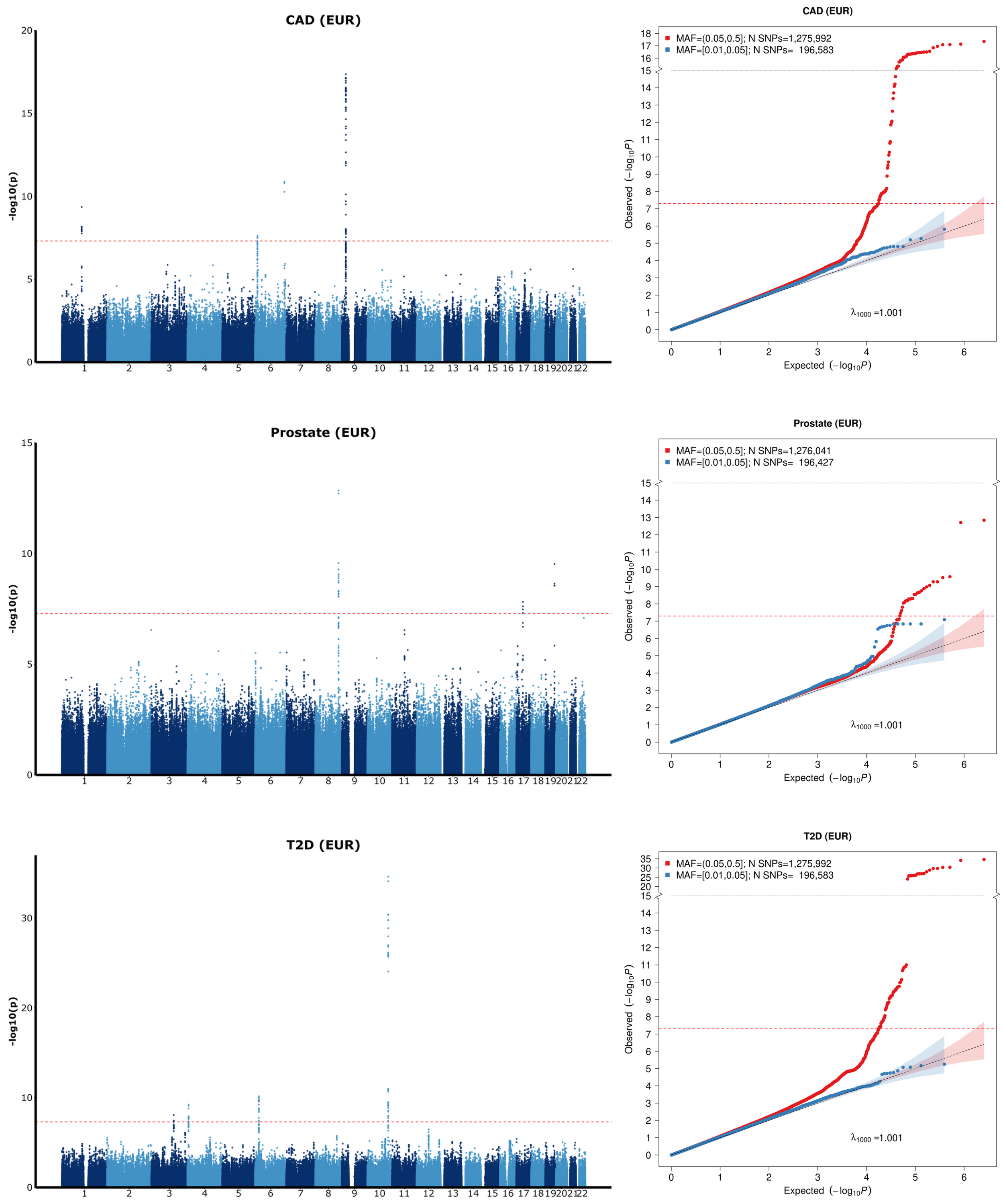

Supplementary Figure 3 continued: Manhattan and QQ Plots for BMI, HDL, and Height based on UK Biobank Imputed + WES GWAS summary statistics in European populations.

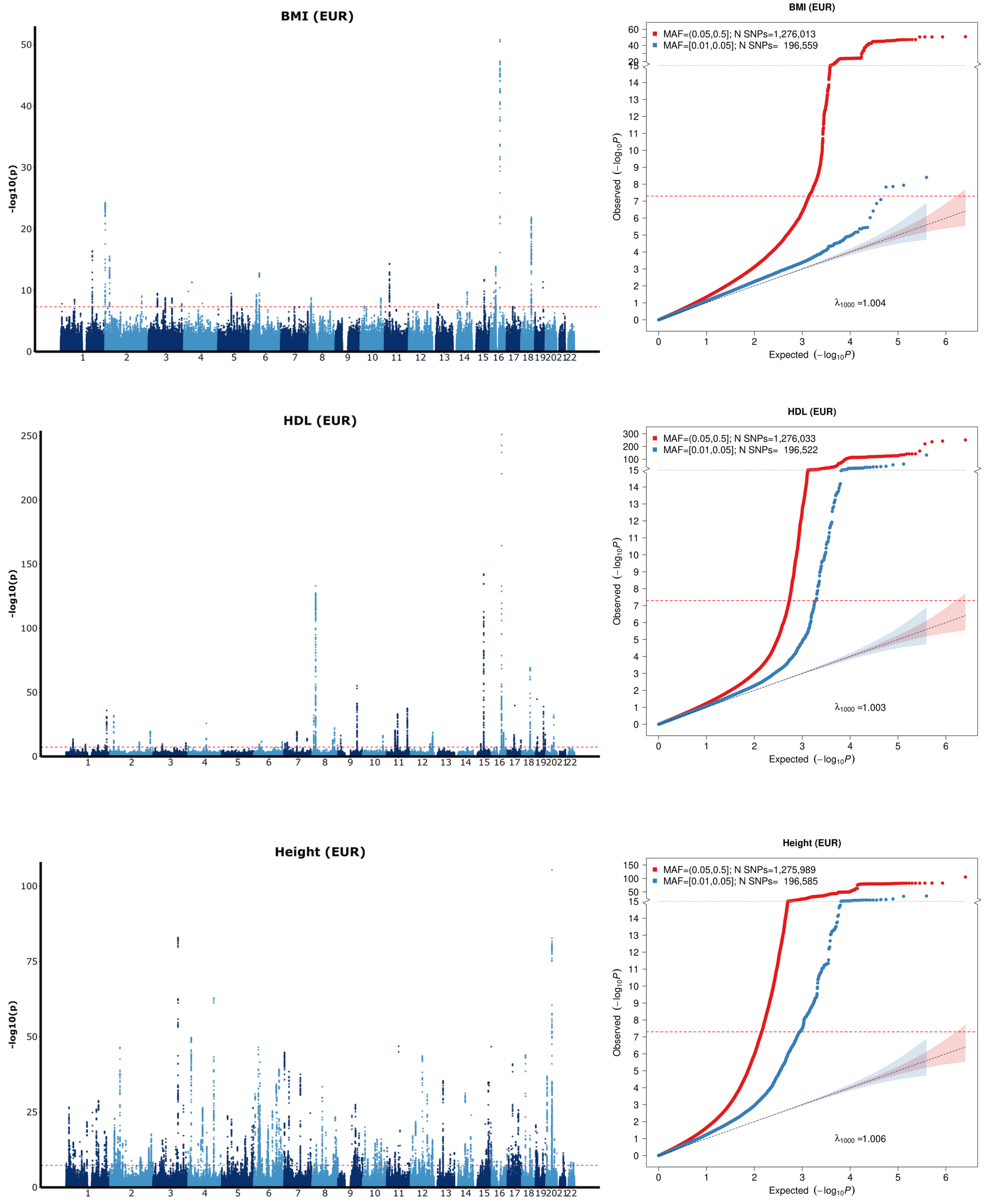

Supplementary Figure 3 continued: Manhattan and QQ Plots for LDL, log(TG), and TC based on UK Biobank Imputed + WES GWAS summary statistics in European populations.

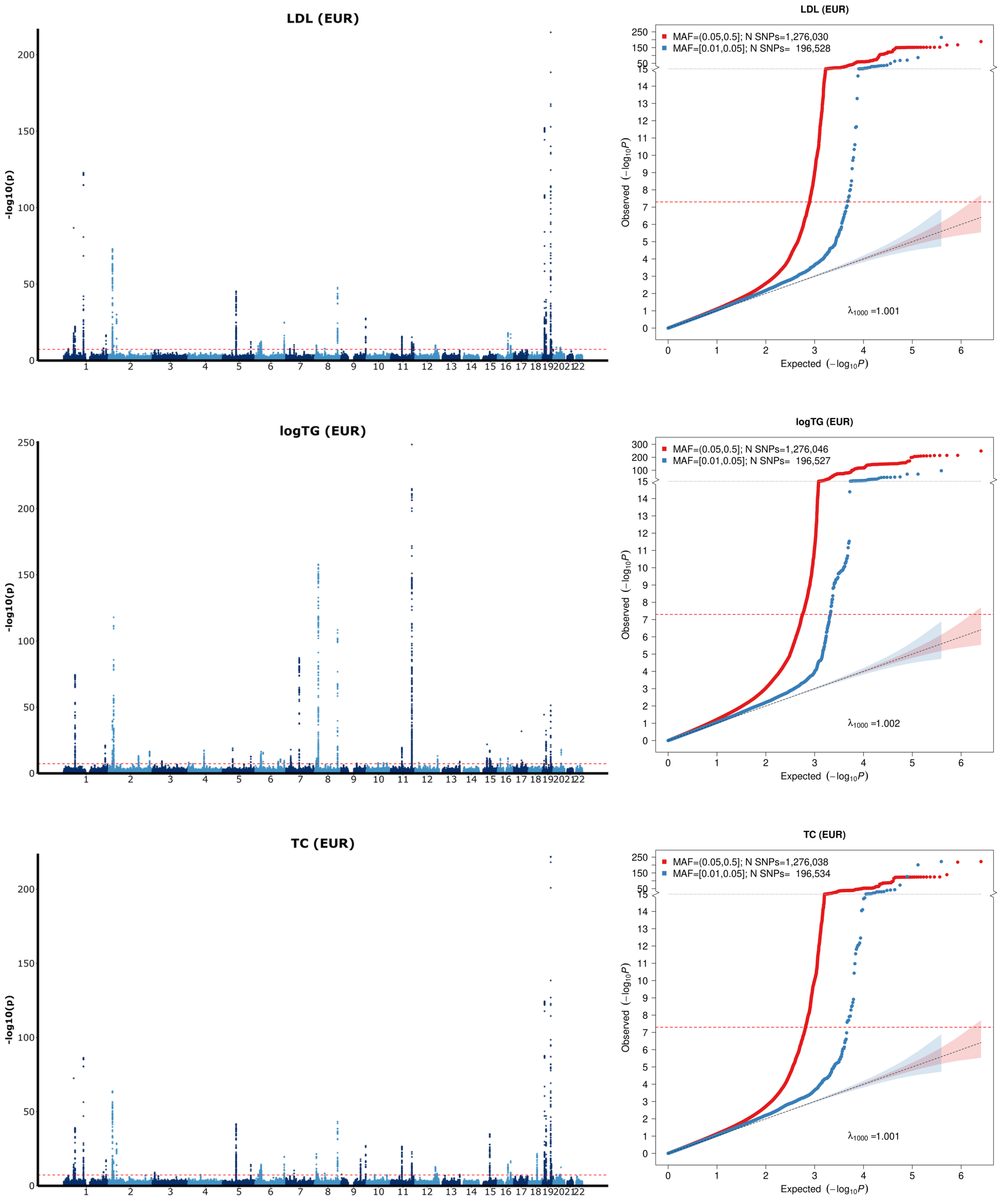

Supplementary Figure 4. QQ plots based on the UK Biobank imputed + whole exome sequencing rare variant association analysis for coding genes conducted with STAARpipeline using the training set consisting of only individuals of European ancestry (EUR) for 11 traits (sample sizes provided in **Supplementary Data 2**). The five binary traits analyzed and displayed in Supp. Fig. 4a: asthma, breast cancer, coronary artery disease (CAD), prostate cancer, and type 2 diabetes (T2D) and the six continuous traits analyzed and displayed in Supp. Fig. 4b: body mass index (BMI), high-density lipoprotein cholesterol (HDL), height, low-density lipoprotein cholesterol (LDL), natural logarithm of triglycerides (log(TG)), and total cholesterol (TC). P-values are split into the five functional categories investigated in the gene-centric coding analysis in the STAARpipeline: putative loss of function (pLoF), putative loss of function and disruptive (pLoF+D), missense, disruptive missense, and synonymous. Under the null hypothesis, the p-value follows a $\text{Uniform}(0,1)$ distribution. Source data are provided as a Source Data file.

a) QQ plots for five binary traits from the UKB Imputed + WES rare variant association analysis using coding genes with STAARpipeline.

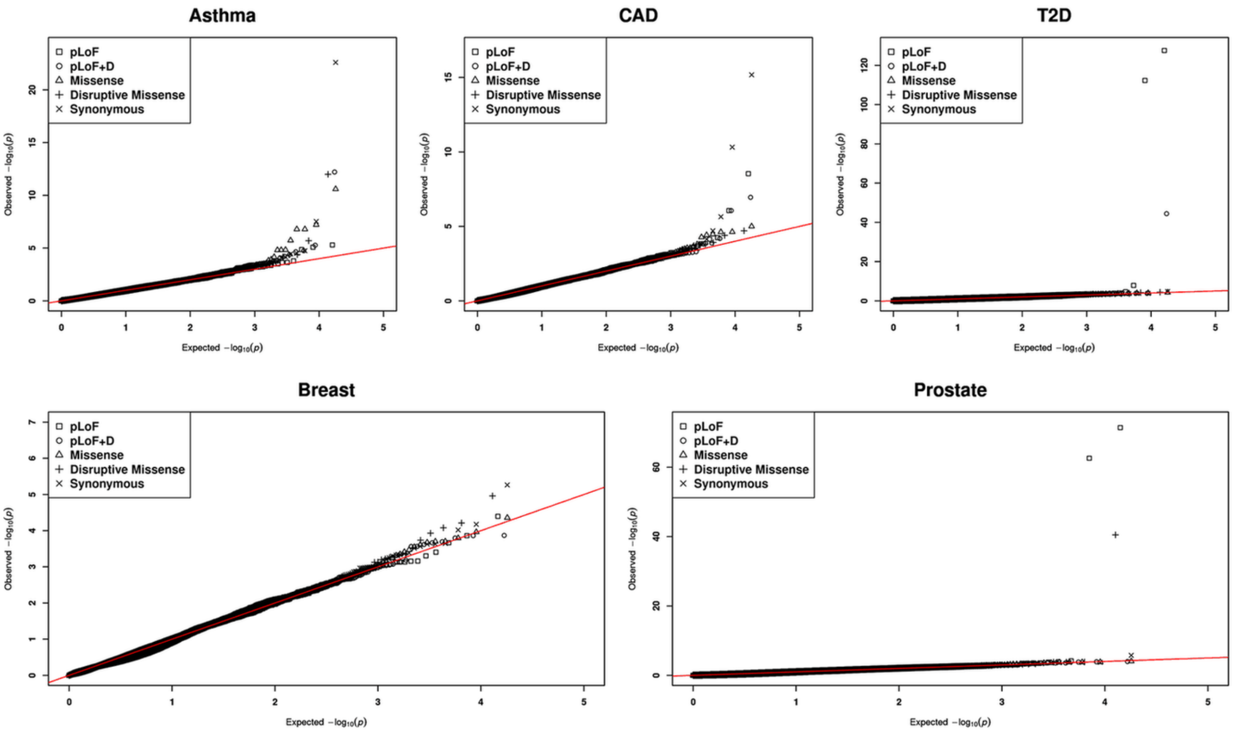

Supplementary Figure 4 continued: b) QQ plots for six continuous traits from the UKB Imputed + WES rare variant association analysis using coding genes with STAARpipeline.

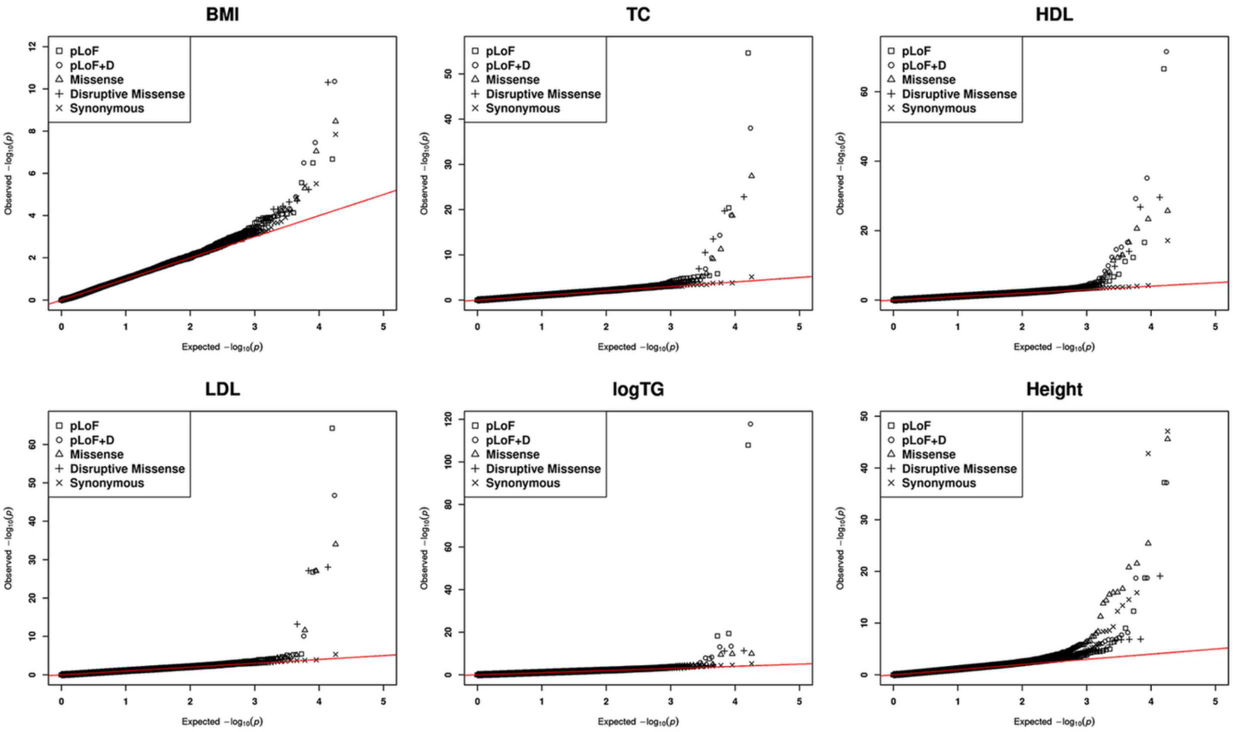

Supplementary Figure 5. Manhattan plot and QQ plots based on the UK Biobank whole genome sequencing (WGS) GWAS summary statistics computed using the training set consisting of only individuals of European ancestry (EUR) for five binary traits: asthma, breast cancer, coronary artery disease (CAD), prostate cancer, and type 2 diabetes (T2D) and six continuous traits: body mass index (BMI), high-density lipoprotein cholesterol (HDL), height, low-density lipoprotein cholesterol (LDL), natural logarithm of triglycerides (log(TG)), and total cholesterol (TC). The red and blue shaded regions around the diagonal line in the QQ plots indicate the 95% confidence intervals expected under the null hypothesis of no association between genetic variants and the trait of interest, for minor allele frequencies (MAF) within the ranges (0.05, 0.5] and [0.01, 0.05], respectively. The jth order statistic follows a $\text{Beta}(j, N-j+1)$ distribution, where N is the total number of variants given a specific MAF cutoff. For binary traits, λ_{1000} scales λ to a study with 1000 cases and 1000 controls using $\lambda_{1000} = 1 + 1000 \times (\lambda - 1) \times \left(\frac{1}{N_{\text{case}}} + \frac{1}{N_{\text{control}}}\right)$. Genomic control factors are shown in **Supplementary Data 4**. Source data are provided as a Source Data file.

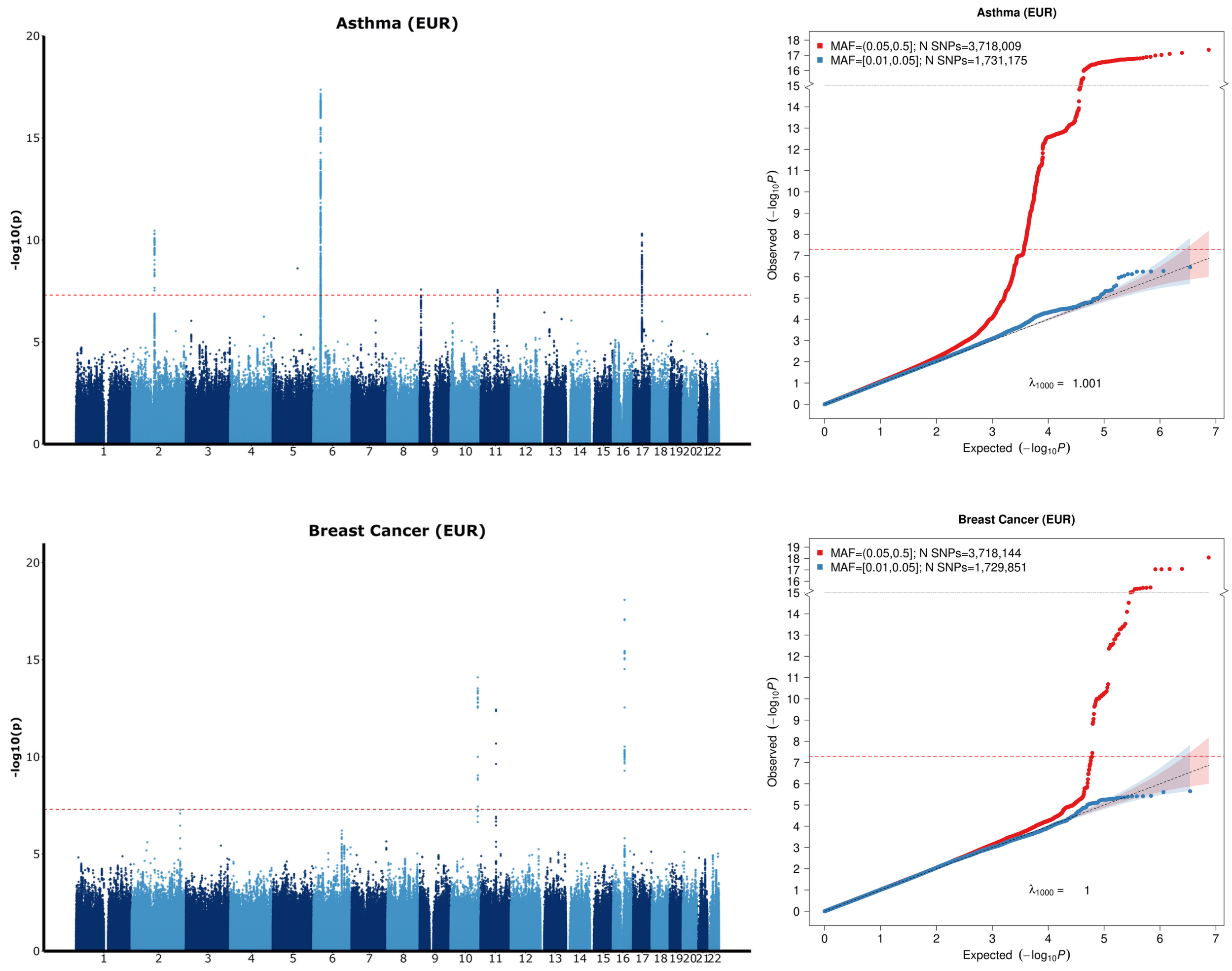

Supplementary Figure 5 continued. Manhattan and QQ Plots for CAD, prostate cancer and T2D based on UK Biobank WGS GWAS summary statistics in European populations.

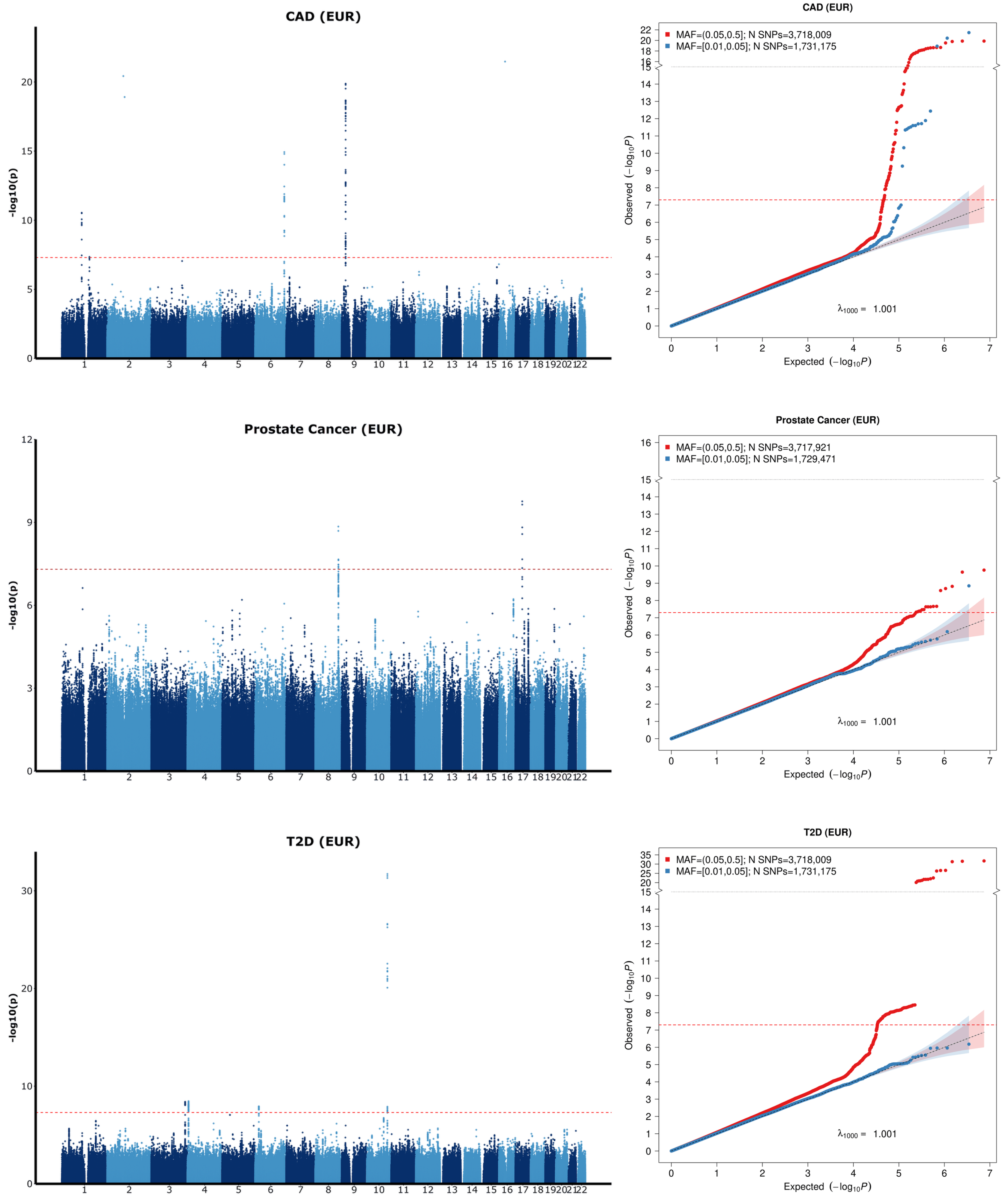

Supplementary Figure 5 continued. Manhattan and QQ Plots for BMI, HDL, and Height based on UK Biobank WGS GWAS summary statistics in European populations.

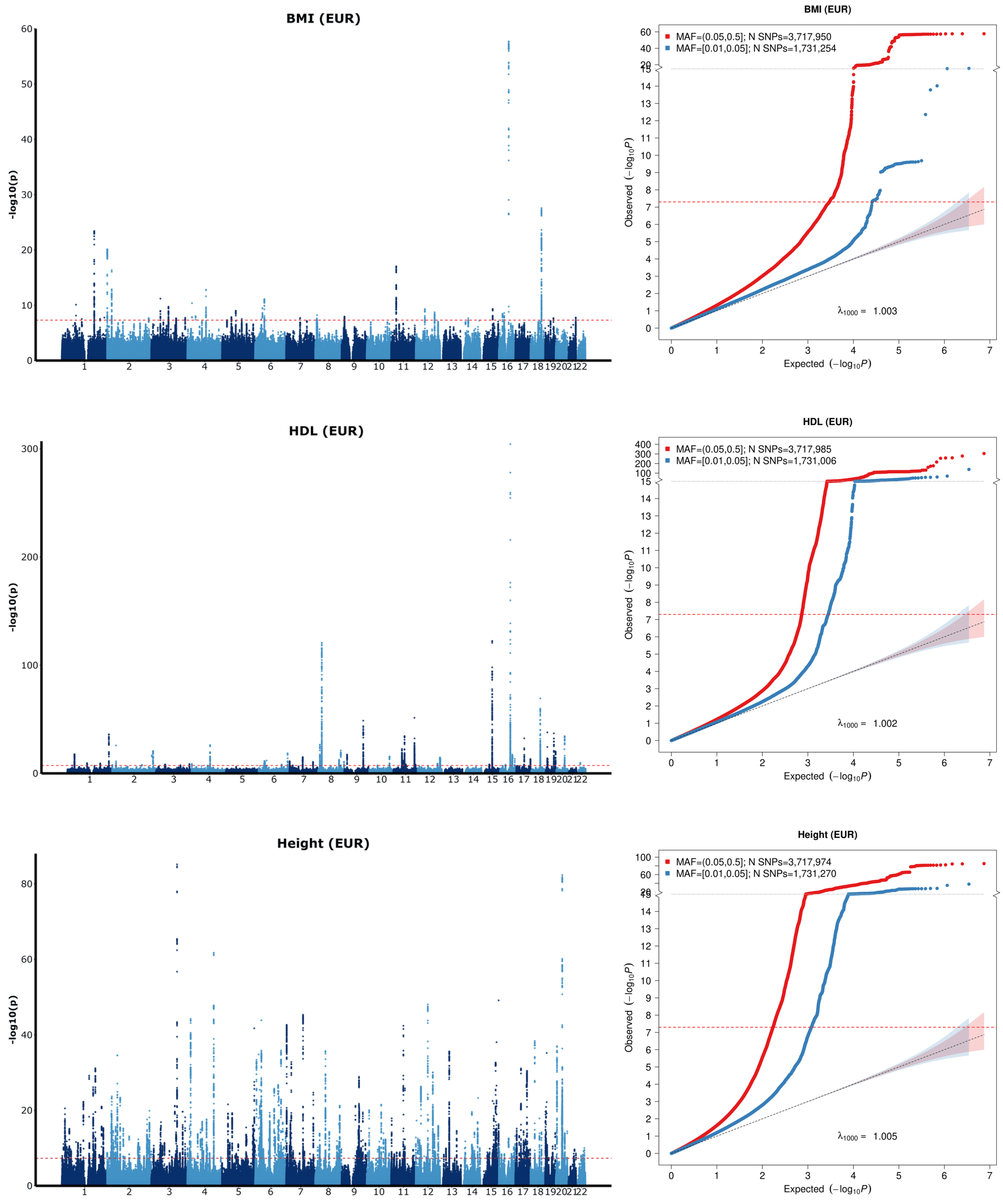

Supplementary Figure 5 continued. Manhattan and QQ Plots for LDL, log(TG), and TC based on UK Biobank WGS GWAS summary statistics in European populations.

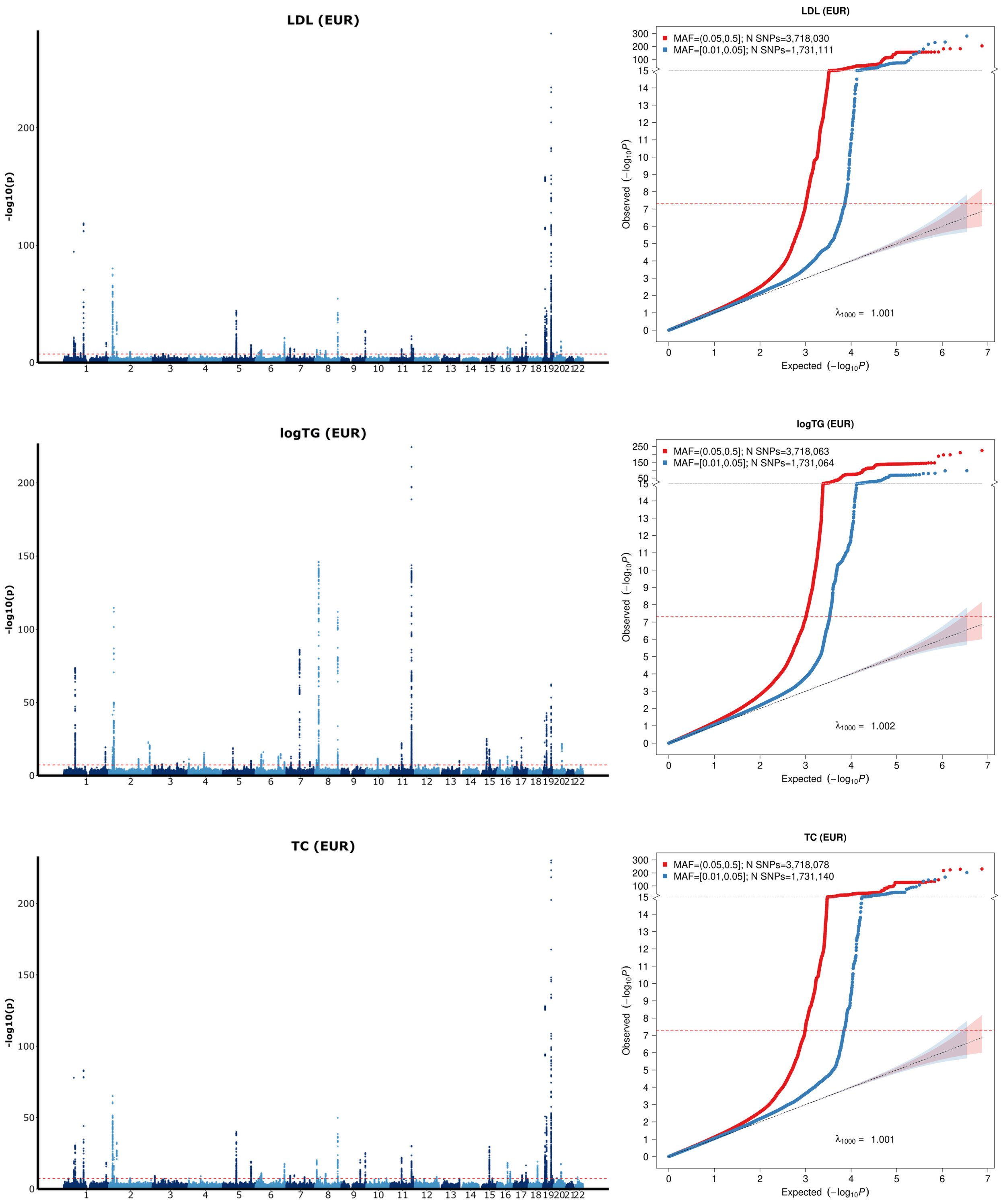

Supplementary Figure 6. QQ plots based on the UK Biobank whole genome sequencing rare variant association analysis for coding and noncoding genes conducted with STAARpipeline using the training set consisting of only individuals of European ancestry (EUR) for 11 traits (sample sizes provided in **Supplementary Data 3**). The five binary traits analyzed and displayed in Supp. Fig. 6a and 6b: asthma, breast cancer, coronary artery disease (CAD), prostate cancer, and type 2 diabetes (T2D) and the six continuous traits analyzed and displayed in Supp. Fig. 6c and 6d: body mass index (BMI), high-density lipoprotein cholesterol (HDL), height, low-density lipoprotein cholesterol (LDL), natural logarithm of triglycerides (log(TG)), and total cholesterol (TC). P-values using coding genes (Supp. Fig. 6a and 6c) are split into the five functional categories investigated in the gene-centric coding analysis in the STAARpipeline: putative loss of function (pLoF), putative loss of function and disruptive (pLoF+D), missense, disruptive missense, and synonymous. P-values using noncoding genes (Supp. Fig. 6b and 6d) are split into the eight functional categories investigated in the gene-centric noncoding analysis in the STAARpipeline: upstream, downstream, noncoding RNA (ncRNA), untranslated regions (UTR), promoters within cap-analysis gene expression (promoter-CAGE), promoters within DNase 1 hypersensitive regions (promoter-DHS), enhancers within cap-analysis gene expression (enhancer-CAGE), and enhancers within DNase 1 hypersensitive regions (enhancer-DHS). Under the null hypothesis, the p-value follows a $\mathrm{Uniform}(0,1)$ distribution. Source data are provided as a Source Data file.

a) QQ plots for five binary traits from the UKB WGS rare variant association analysis using coding genes with STAARpipeline.

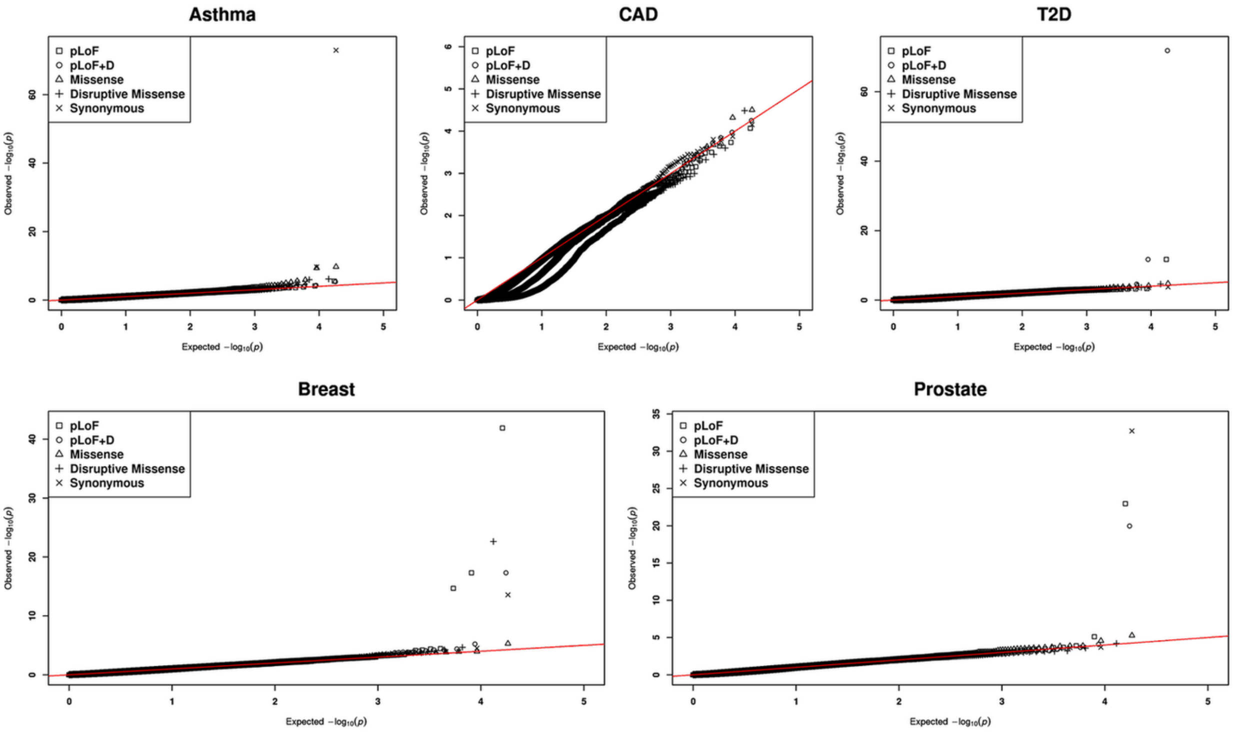

Supplementary Figure 6 continued: b) QQ plots for five binary traits from the UKB WGS rare variant association analysis using noncoding genes with STAARpipeline.

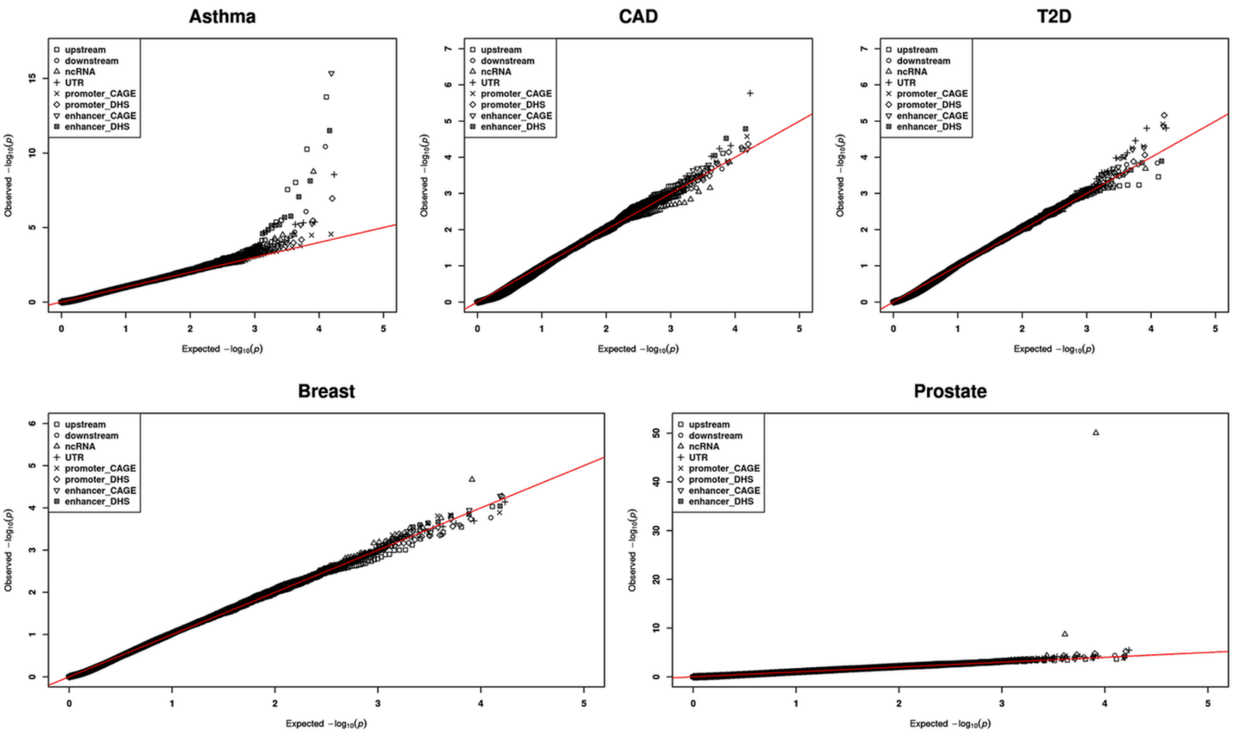

c) QQ plots for six continuous traits from the UKB WGS rare variant association analysis using coding genes with STAARpipeline.

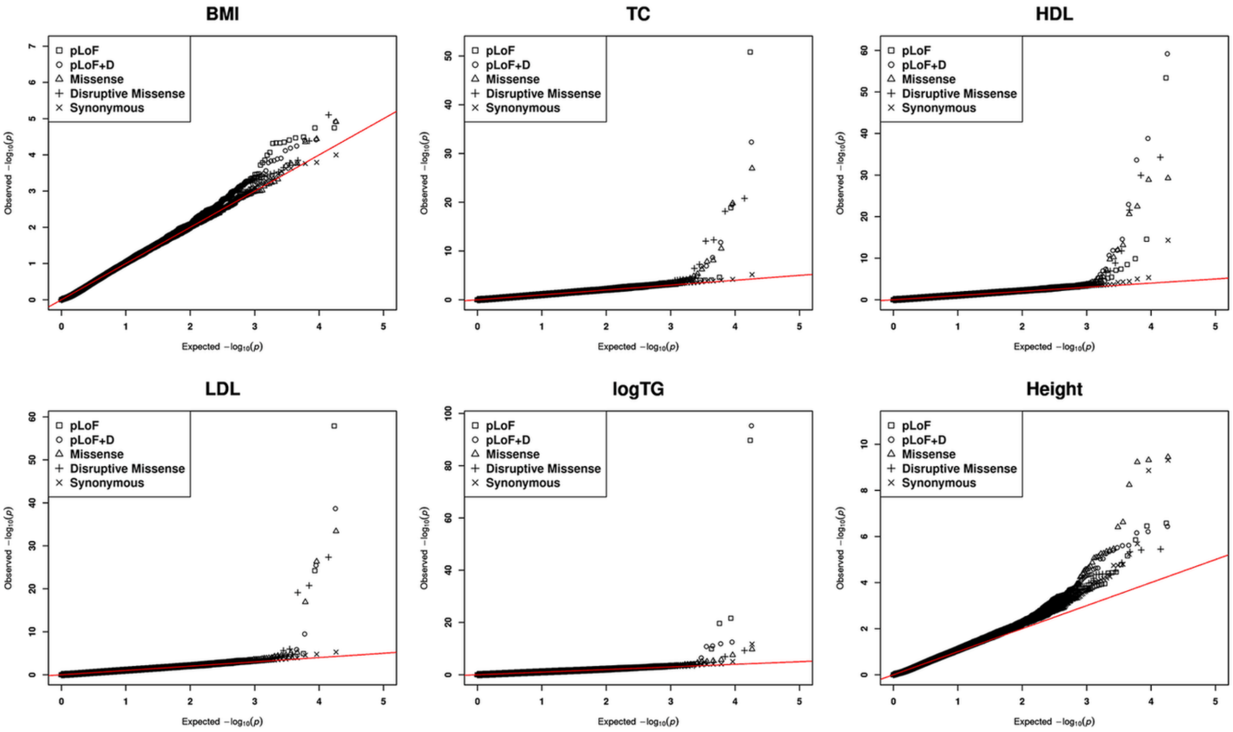

Supplementary Figure 6 continued: d) QQ plots for six continuous traits from the UKB WGS rare variant association analysis using noncoding genes with STAARpipeline.

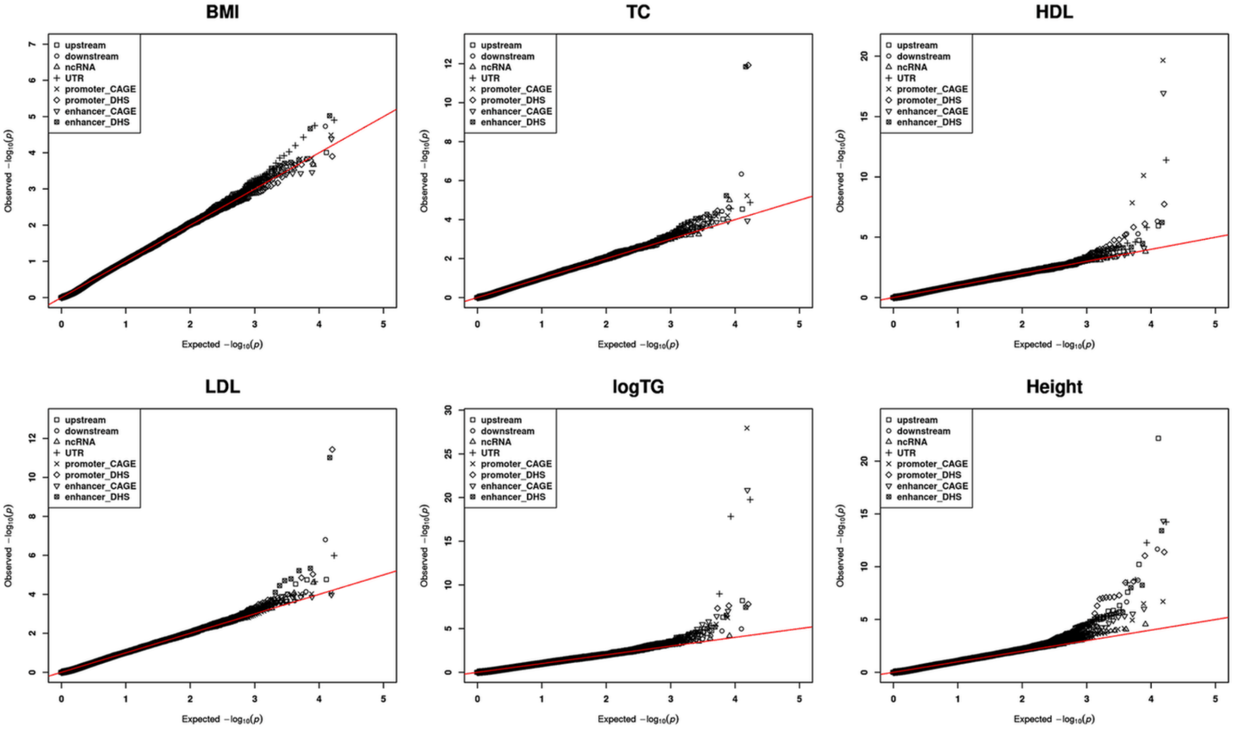

Supplementary Figure 7. Predictive performance of ancestry-adjusted PRSs for 11 traits across four ancestral groups from UK Biobank (UKB) imputed + whole exome sequencing data (WES) data. The five binary traits analyzed and displayed in Supp. Fig. 7a and 7b: asthma, breast cancer, coronary artery disease (CAD), prostate cancer, and type 2 diabetes (T2D). The six continuous traits analyzed and displayed in Supp. Fig. 7c include body mass index (BMI), height, high-density lipoprotein cholesterol (HDL), low-density lipoprotein cholesterol (LDL), the natural logarithm of triglyceride cholesterol (log(TG)), and total cholesterol (TC). Results are shown for individuals of African (AFR), Admixed American or Latino (AMR), European (EUR), and South Asian (SAS) ancestries. The training data consisted solely of individuals of European ancestry, while tuning and validation sets included all four ancestries. Full sample sizes details for each ancestry are provided in **Supplementary Data 2**. Statistical significance of the RICE-RV component was assessed using percentile bootstrap confidence intervals (10,000 resamples), testing the two-sided alternative $H_A: \beta_{\mathrm{RV}} \neq 0$; *** indicates the lower bound of the 99% bootstrap CI > 0 and ** indicates the lower bound of the 95% bootstrap CI > 0. In the second figure, PRS performance is evaluated with R^2 derived from the regression model $Y \sim \mathrm{PRS} \times \beta$, with β representing the effect of standardized PRS on the standardized outcome (**Methods**). For RICE, R^2 or AUC is derived using a predicted PRS from a linear model containing both RICE-CV and RICE-RV trained on the tuning dataset. Significance of R^2 or AUC was assessed using 10,000 bootstrap resamples of the validation set, testing whether the pairwise difference, ${R^2}_{\mathrm{RICE}} - {R^2}_{\mathrm{Best\ Alternative}}$ or $\mathrm{AUC}_{\mathrm{RICE}} - \mathrm{AUC}_{\mathrm{Best\ Alternative}}$, (per trait–ancestry pair) differs from 0 ($p < 0.05$, **; $p < 0.01$, ***; exact p-values in Source Data). Exact bootstrap p-values and CI bounds are provided in the Source Data file. Source data are provided as a Source Data file.

a) Beta of PRS per SD of ancestry-adjusted PRSs for five binary traits across four ancestral groups from UKB Imputed + WES data.

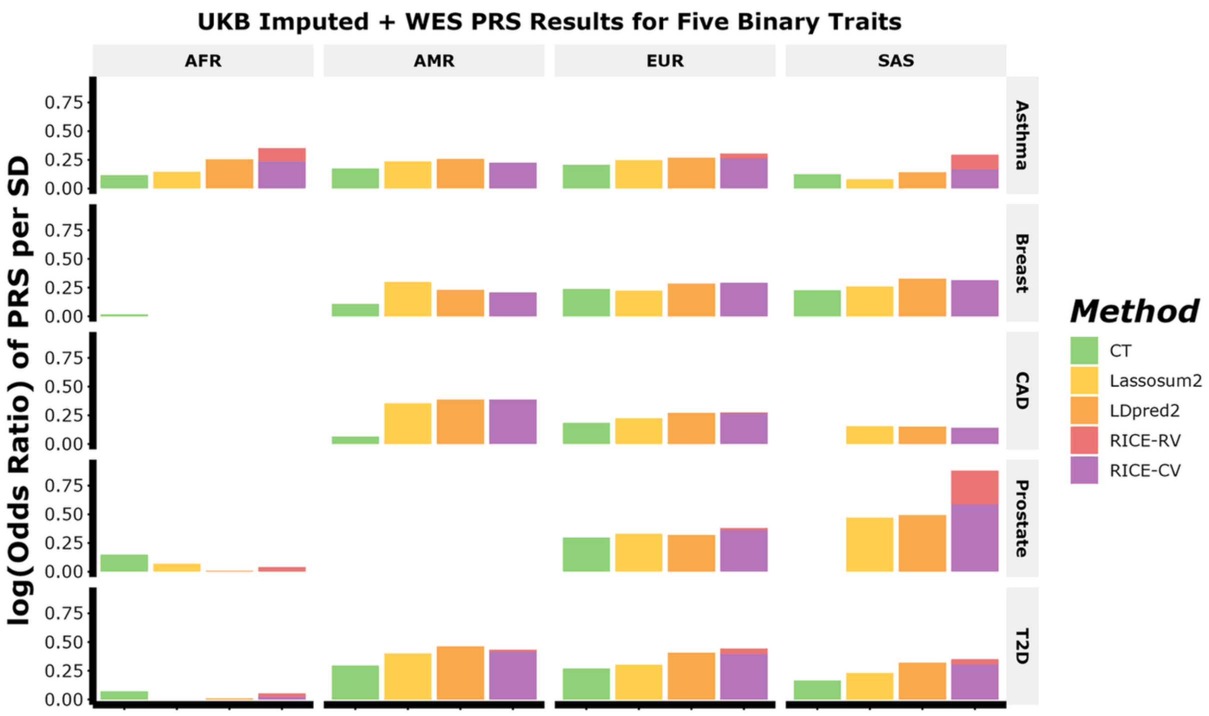

Supplementary Figure 7 continued: b) AUC of ancestry-adjusted PRSs for five binary traits across four ancestral groups from UKB Imputed + WES data.

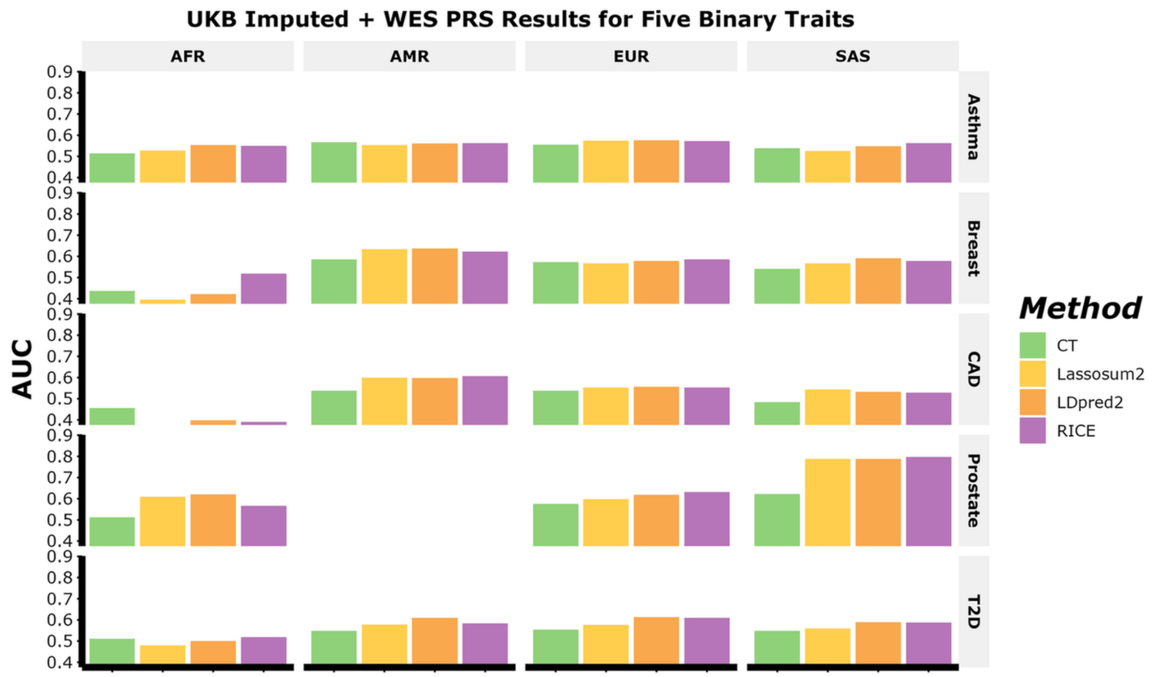

c) R^2 of ancestry-adjusted PRSs for six continuous traits across four ancestral groups from UKB Imputed + WES data. Percentages above the RICE bars indicate the relative improvement in R^2 achieved by RICE compared to the best alternative method for each ancestry–trait combination.

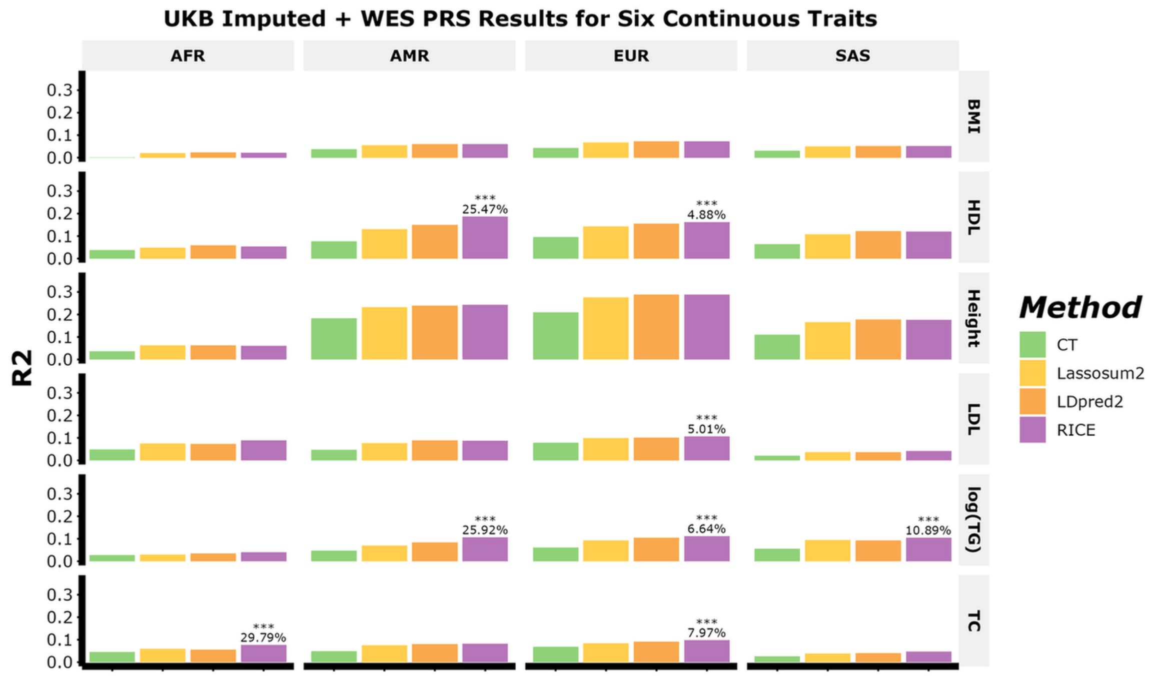

Supplementary Figure 8. Relationship between common and rare variant PRSs and standardized traits across four ancestral groups from UK Biobank (UKB) imputed + whole-exome sequencing (WES) data. The six continuous traits analyzed and shown: body mass index (BMI) (Supp. Fig. 8a), high-density lipoprotein cholesterol (HDL) (Supp. Fig. 8b), height (Supp. Fig. 8c), low-density lipoprotein cholesterol (LDL) (Supp. Fig. 8d), natural logarithm of triglyceride cholesterol (log(TG)) (Supp. Fig. 8e), and total cholesterol (TC) (Supp. Fig. 8f). PRS quantiles for RICE-CV (common variants) are plotted on the x-axis, and standardized trait on the y-axis. Data are stratified by rare variant PRS quantiles from RICE-RV (blue: below 5%, grey: 20–70%, pink: above 95%). Mean $\pm 1 \times$ SE are shown for individuals of African (AFR), Admixed American/Latino (AMR), European (EUR), and South Asian (SAS) ancestries. The training data consisted solely of individuals of European ancestry, while the tuning and validation sets included all four ancestries. Full sample sizes details for each ancestry are provided in **Supplementary Data 2**. Exact values to reconstruct this plot can be found in the Source Data file. Source data are provided as a Source Data file.

a) Relationship between ancestry-adjusted common and rare variant PRSs and standardized body mass index (BMI) levels across four ancestral groups from UKB Imputed + WES data.

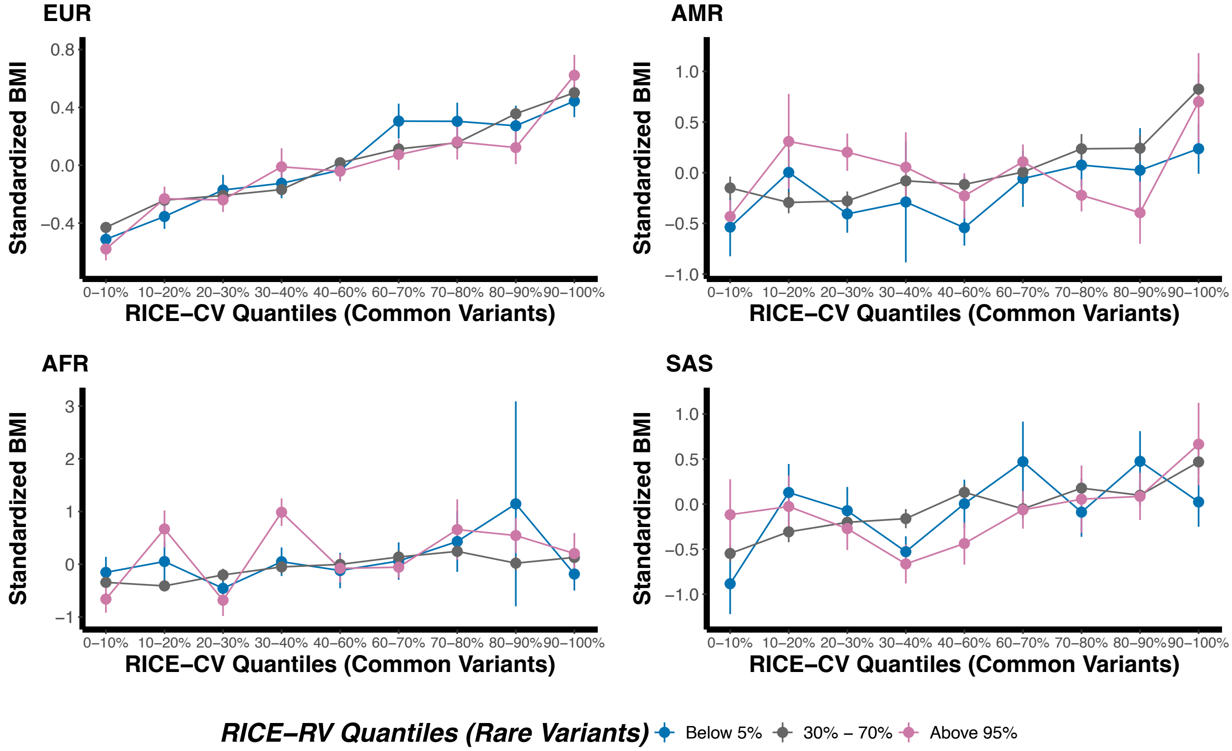

Supplementary Figure 8 continued: b) Relationship between ancestry-adjusted common and rare variant PRSs and standardized high-density lipoprotein cholesterol (HDL) across four ancestral groups from UKB Imputed + WES data.

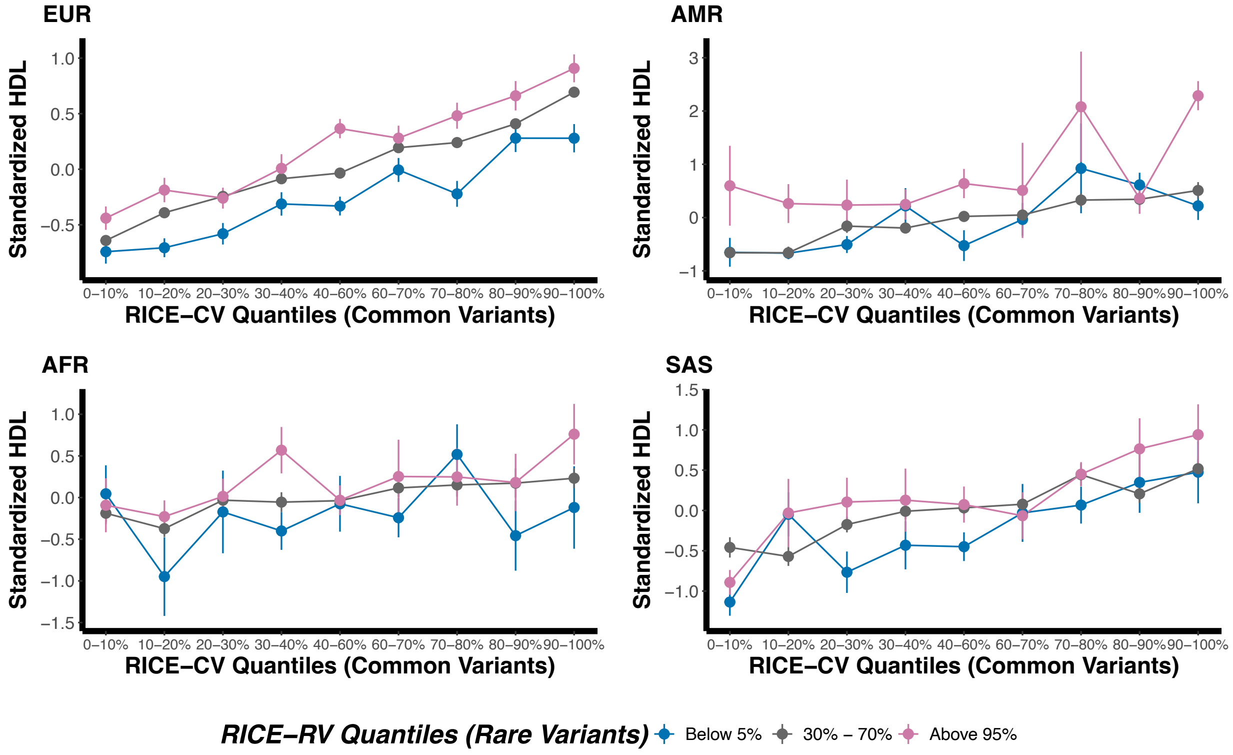

Supplementary Figure 8 continued: c) Relationship between ancestry-adjusted common and rare variant PRSs and standardized height levels across four ancestral groups from UKB Imputed + WES data.

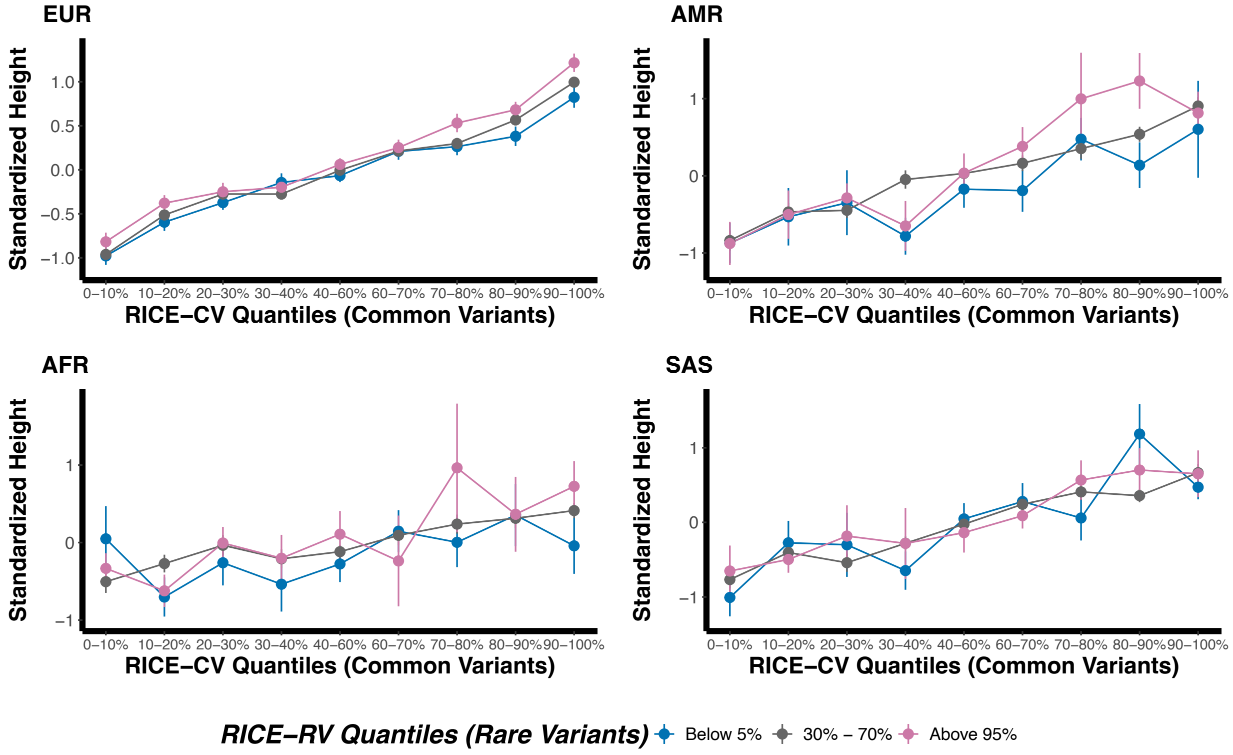

Supplementary Figure 8 continued: d) Relationship between ancestry-adjusted common and rare variant PRSs and standardized low-density lipoprotein cholesterol (LDL) levels across four ancestral groups from UKB Imputed + WES data.

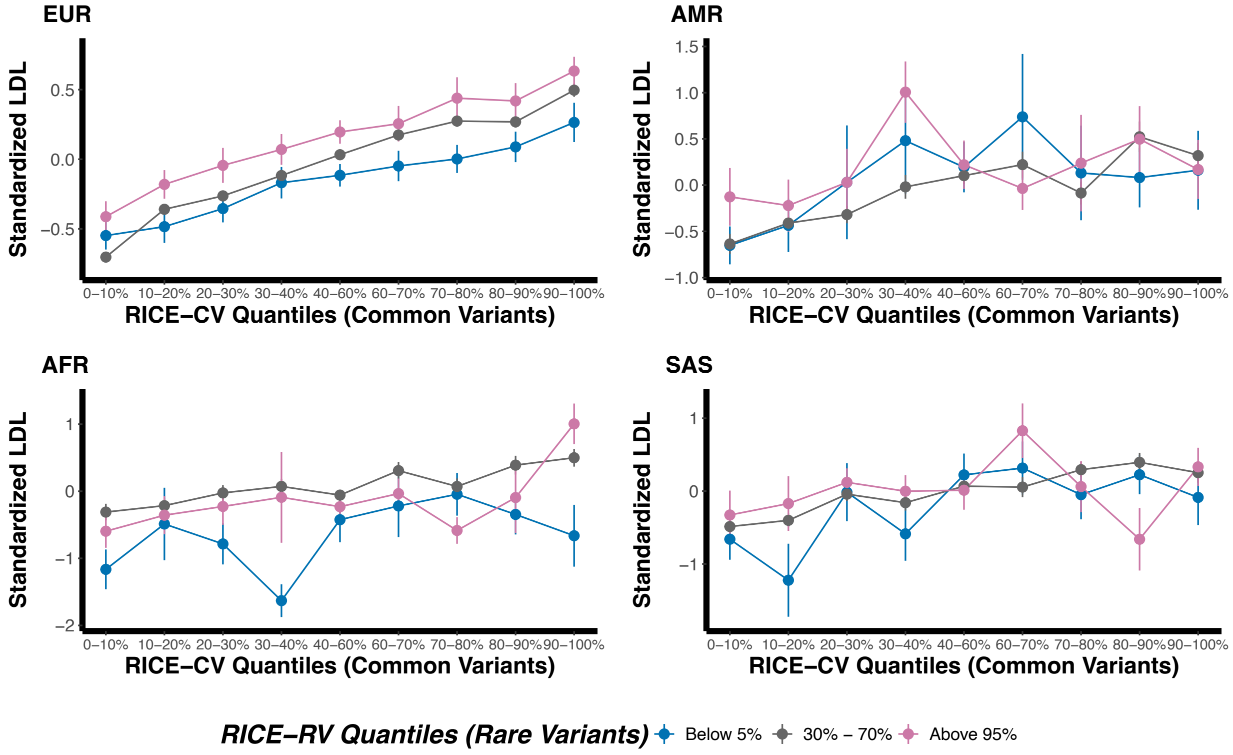

Supplementary Figure 8 continued: e) Relationship between ancestry-adjusted common and rare variant PRSs and standardized natural logarithm of triglycerides (log(TG)) levels across four ancestral groups from UKB Imputed + WES data.

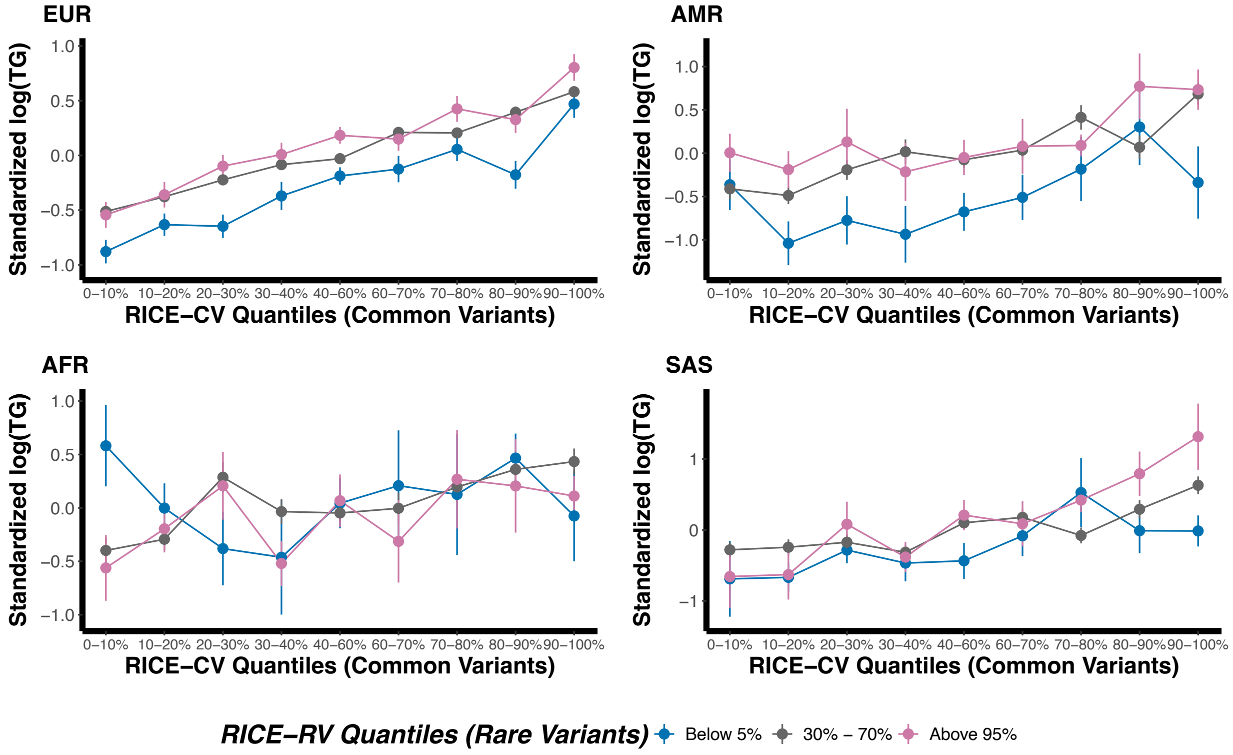

Supplementary Figure 8 continued: f) Relationship between ancestry-adjusted common and rare variant PRSs and standardized total cholesterol (TC) levels across four ancestral groups from UKB Imputed + WES data.

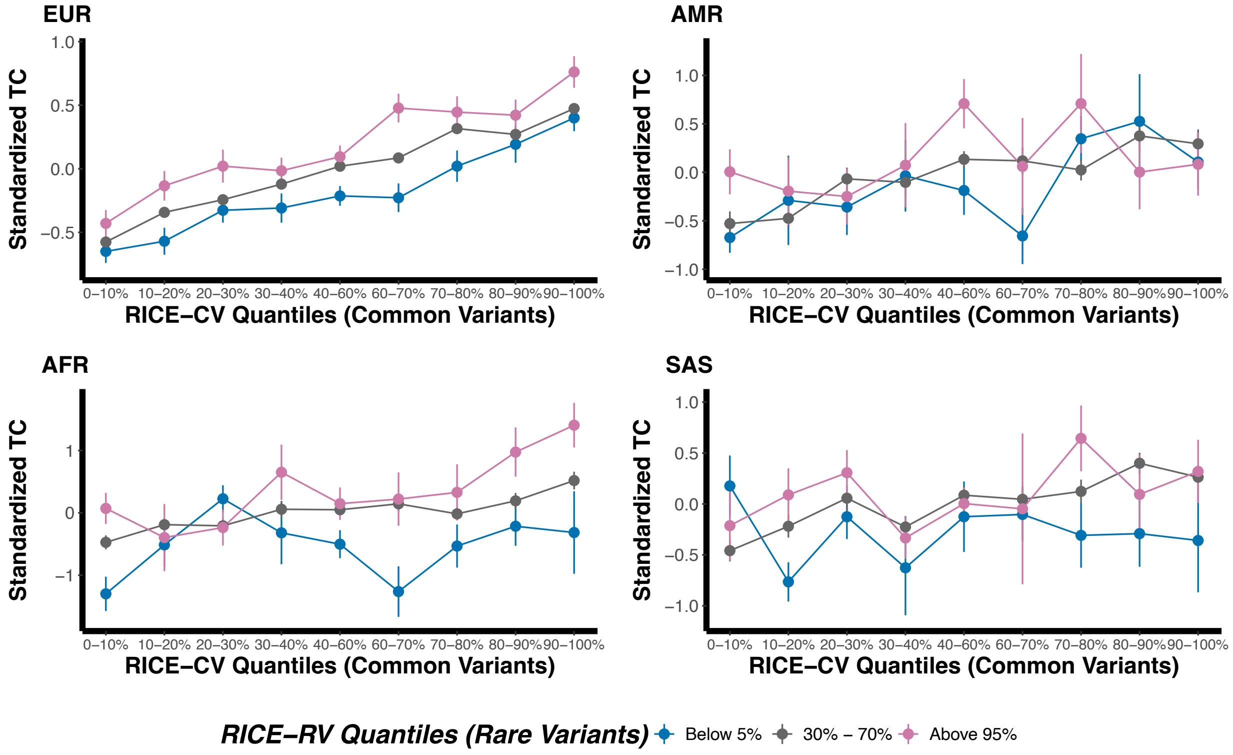

Supplementary Figure 9. Joint stratification of common and rare variant PRS for standardized traits across four ancestral groups from UK Biobank (UKB) imputed + whole-exome sequencing (WES) data. The five continuous traits analyzed and shown: body mass index (BMI) (Supp. Fig. 9a), height (Supp. Fig. 9b), low-density lipoprotein cholesterol (LDL) (Supp. Fig. 9c), natural logarithm of triglyceride cholesterol (log(TG)) (Supp. Fig. 9d), and total cholesterol (TC) (Supp. Fig. 9f). Individuals in the UKB imputed + WES validation dataset were stratified into based on rare variant PRS (RV; high risk = top 5%). (a) Mean standardized trait levels $\pm 1 \times$ standard error (SE) are plotted stratified by PRS quantiles for RICE-CV (common variants) on the x-axis and RICE-RV (rare variants) by color (blue: below 5%, grey: 30–70%, pink: above 95%). (b) Participants were cross-classified into four strata based on RICE-CV (high = top 10% vs low = bottom 90%) and RICE-RV (high = top 5% vs low = bottom 95%): Low CV/Low RV, High CV/Low RV, Low CV/High RV, and High CV/High RV. Pie chart shows the proportion of individuals in each stratum among the top decile of observed trait values (n = 1,815). (c) Mean standardized trait values for each stratum with 95% confidence intervals (mean $\pm 1.96 \times$ SE). Pairwise p-values were calculated using two-sided Welch's t-tests comparing each stratum to the Low CV/Low RV group; exact p-values and sample size are provided in the Source Data file. Source data are provided as a Source Data file.

Supplementary Figure 9 continued a) Joint stratification of common and rare variant PRS for standardized body mass index (BMI) levels across four ancestral groups from UK Biobank (UKB) imputed + whole-exome sequencing (WES) data.

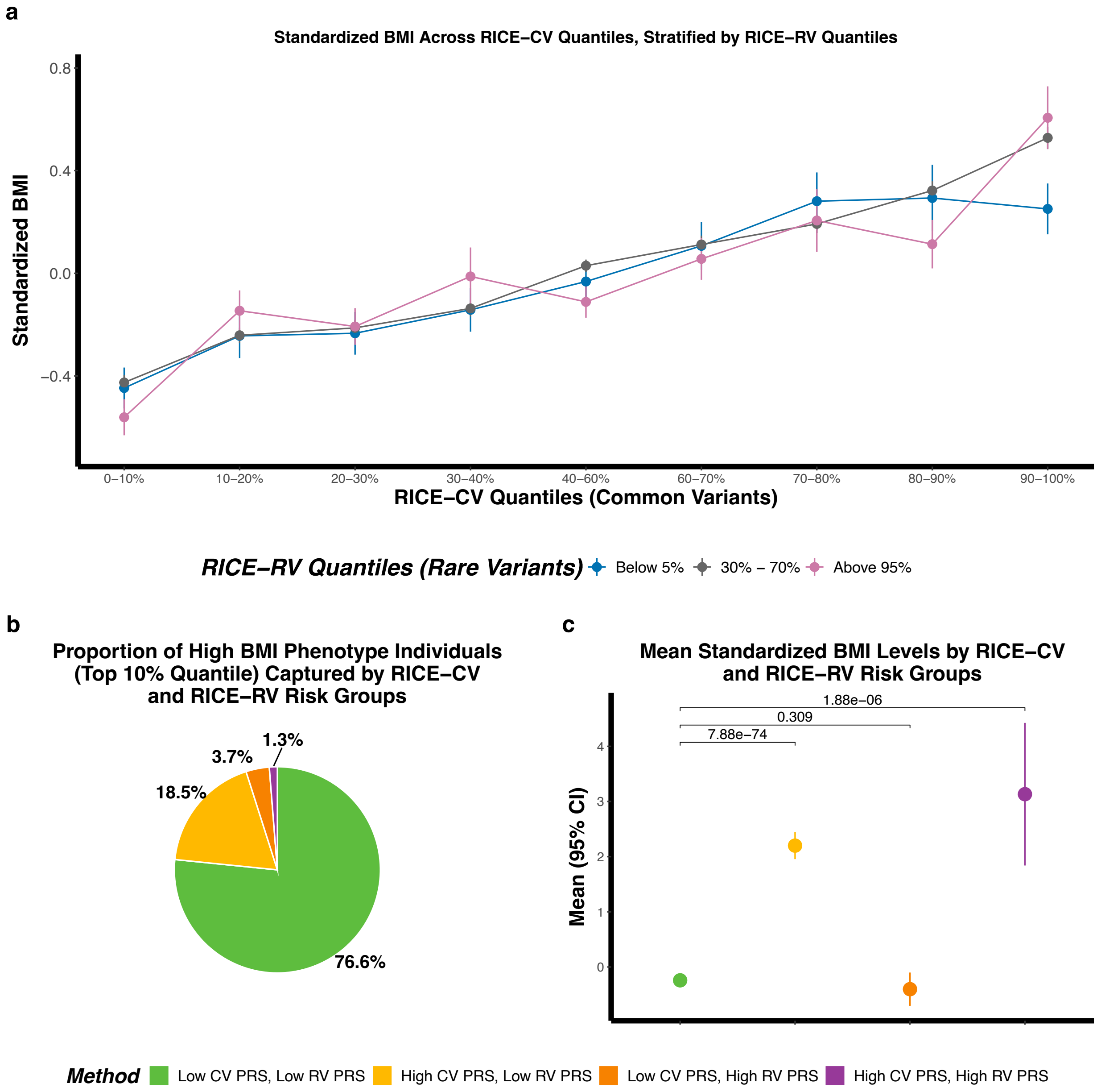

Supplementary Figure 9 continued b) Joint stratification of common and rare variant PRS for standardized height levels across four ancestral groups from UK Biobank (UKB) imputed + whole-exome sequencing (WES) data.

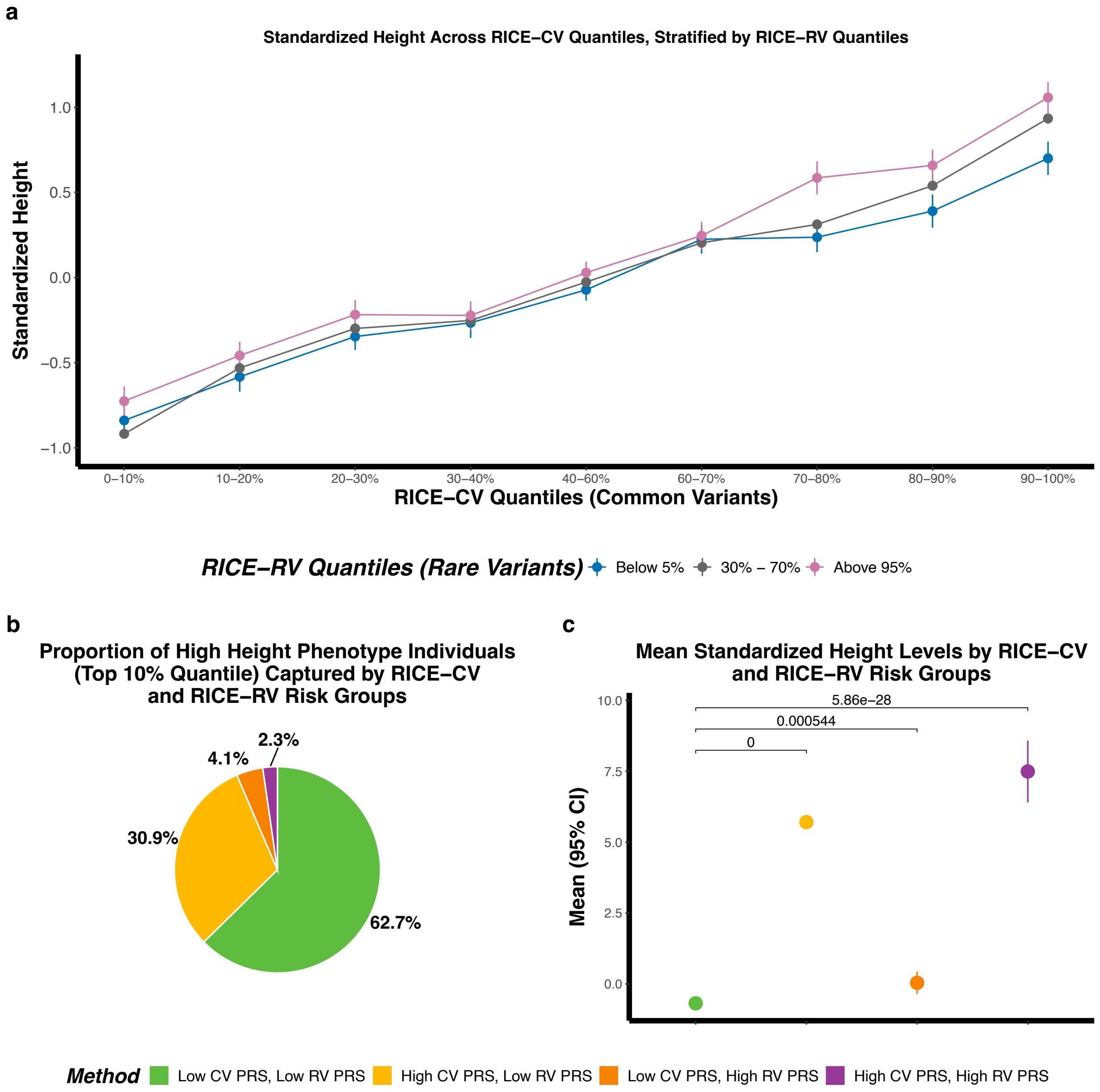

Supplementary Figure 9 continued c) Joint stratification of common and rare variant PRS for standardized low-density lipoprotein cholesterol (LDL) levels across four ancestral groups from UK Biobank (UKB) imputed + whole-exome sequencing (WES) data.

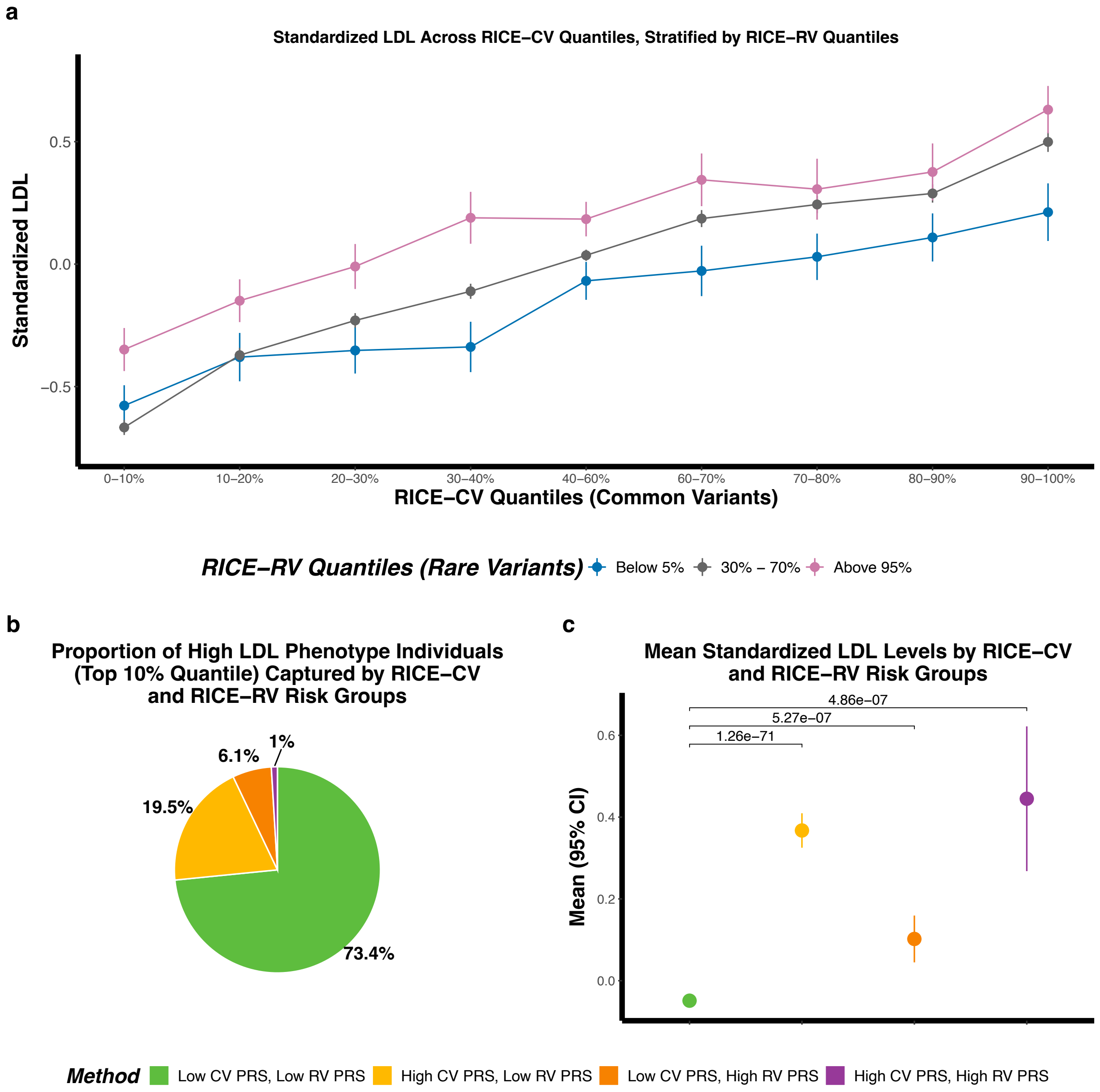

Supplementary Figure 9 continued d) Joint stratification of common and rare variant PRS for standardized natural logarithm of triglycerides (log(TG)) levels across four ancestral groups from UK Biobank (UKB) imputed + whole-exome sequencing (WES) data.

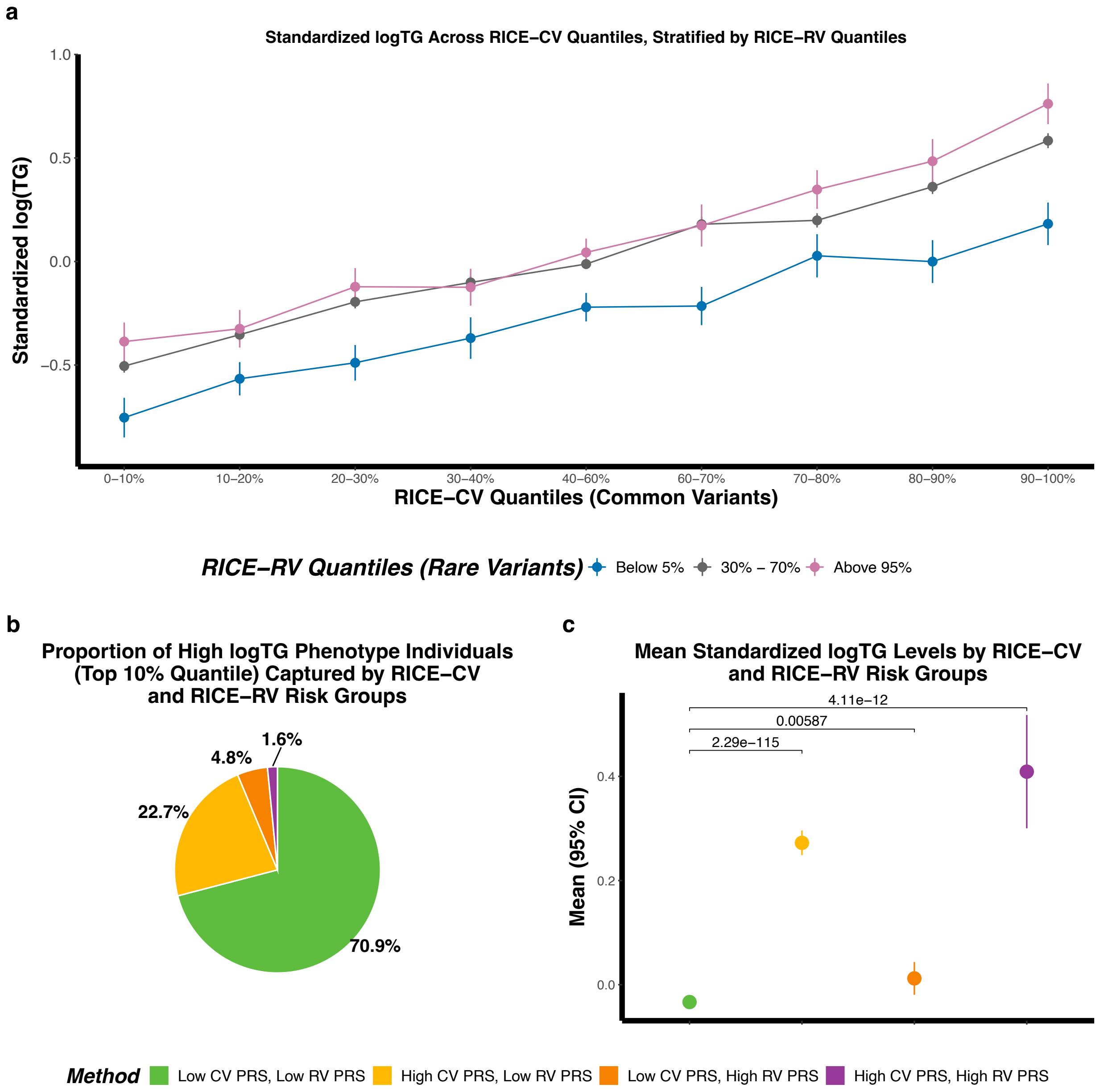

Supplementary Figure 9 continued e) Joint stratification of common and rare variant PRS for standardized total cholesterol (TC) levels across four ancestral groups from UK Biobank (UKB) imputed + whole-exome sequencing (WES) data.

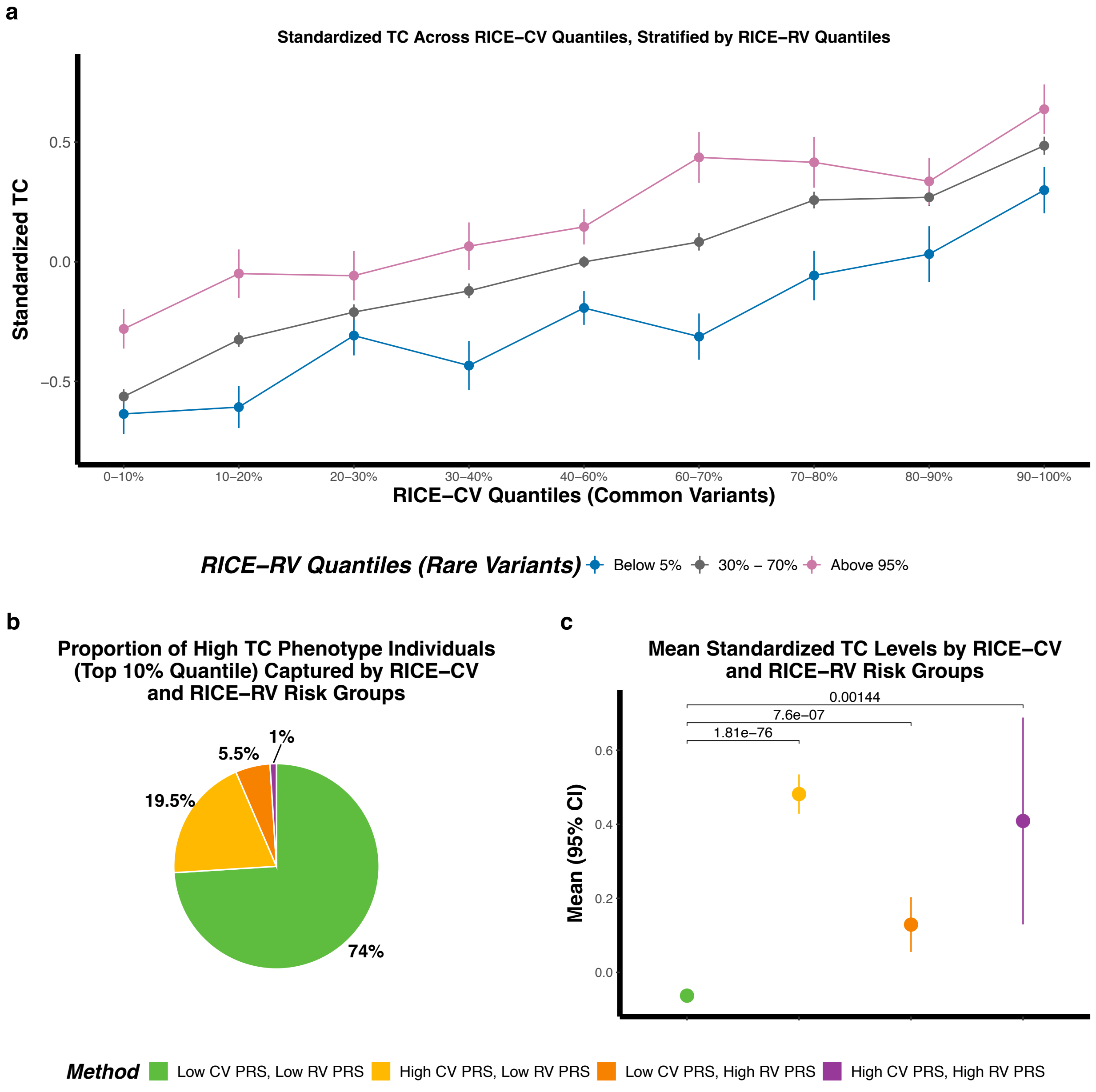

Supplementary Figure 10. Predictive performance of RICE-RV constructed using high-penetrance genes. Using burden scores from high-penetrance genes (*LDLR*, *APOB*, and *PCSK9*), a rare variant PRS was constructed using ensemble regression. The comparison of this PRS to the original RICE-RV PRS is shown for four continuous lipid traits. The training data consisted solely of individuals of European ancestry, while the tuning and validation sets included all four ancestries. Full sample sizes details for each ancestry are provided in **Supplementary Data 2**. Data is plotted by ancestry; African (AFR), Admixed American or Latino (AMR), European (EUR), and South Asian (SAS). Source data are provided as a Source Data file.

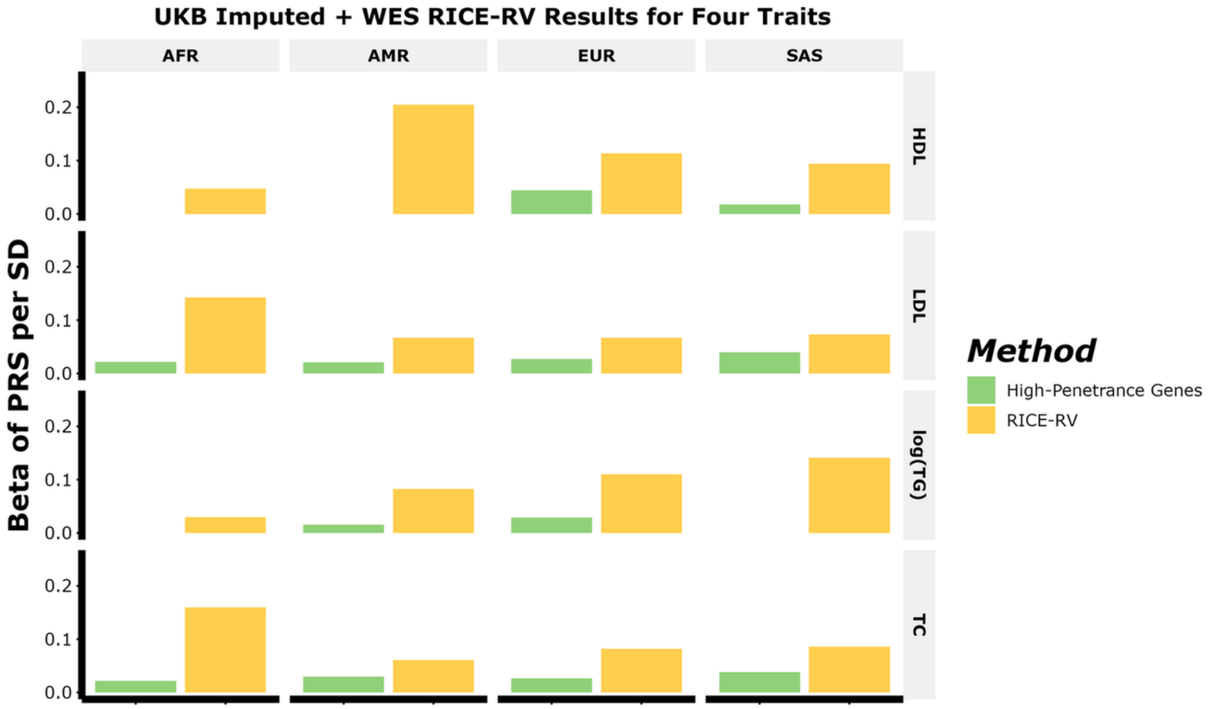

Supplementary Figure 11. Predictive performance of RICE-RV constructed using different p-value thresholds. PRS from RICE-RV was constructed using significant rare variant sets under varying thresholds (1×10^{-5} to 1×10^{-2}). Predictive performance was assessed for six continuous traits. The training data consisted solely of individuals of European ancestry, while the tuning and validation sets included all four ancestries. Data is plotted by ancestry; African (AFR), Admixed American or Latino (AMR), European (EUR), and South Asian (SAS). Full sample sizes details for each ancestry are provided in **Supplementary Data 2**. Source data are provided as a Source Data file.

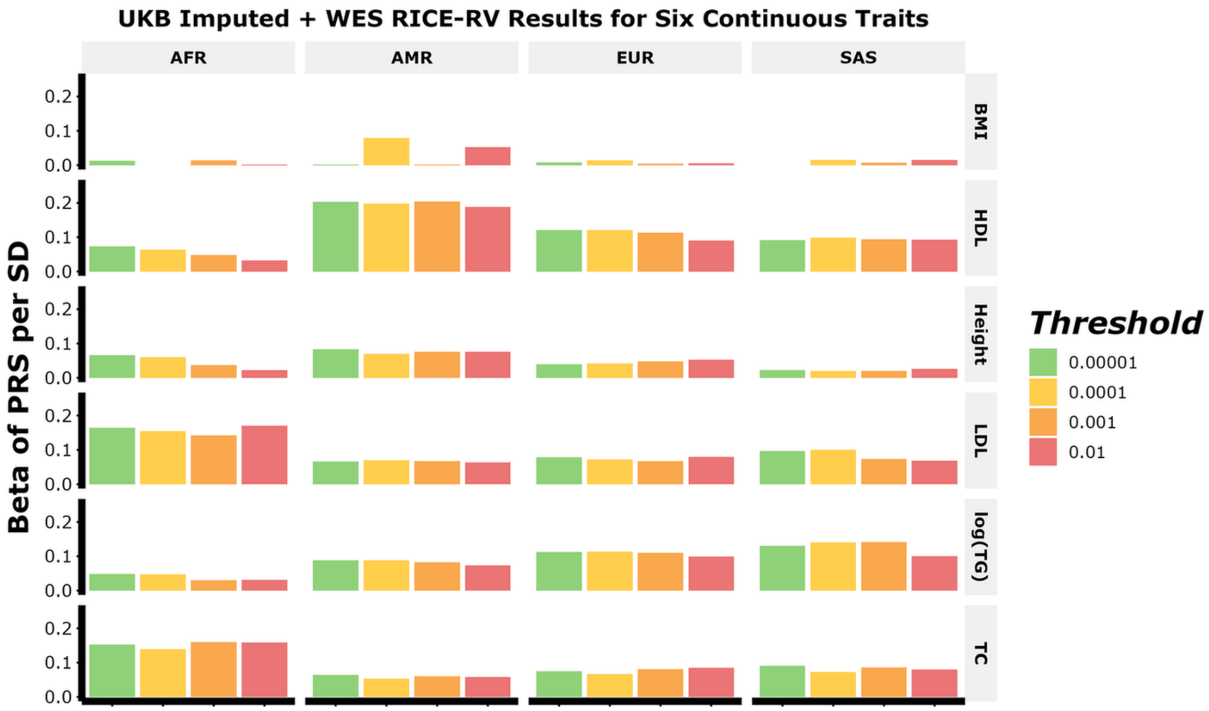

Supplementary Figure 12. Predictive performance of ancestry-adjusted PRSs for 11 traits across four ancestral groups from UK Biobank (UKB) whole genome sequencing data (WGS) data. The five binary traits analyzed and displayed in Supp. Fig. 12a and 12b: asthma, breast cancer, coronary artery disease (CAD), prostate cancer, and type 2 diabetes (T2D). The six continuous traits analyzed and displayed in Supp. Fig. 12c and 12d include body mass index (BMI), height, high-density lipoprotein cholesterol (HDL), low-density lipoprotein cholesterol (LDL), the natural logarithm of triglyceride cholesterol (log(TG)), and total cholesterol (TC). Results are shown for individuals of African (AFR), Admixed American or Latino (AMR), European (EUR), and South Asian (SAS) ancestries. The training data consisted solely of individuals of European ancestry, while tuning and validation sets included all four ancestries. Full sample sizes details for each ancestry are provided in **Supplementary Data 3**. Statistical significance of the RICE-RV component was assessed using percentile bootstrap confidence intervals (10,000 resamples), testing the two -sided alternative $H_A : \beta_{RV} \neq 0$; *** indicates the lower bound of the 99% bootstrap CI > 0 and ** indicates the lower bound of the 95% bootstrap CI > 0. In the second figure, PRS performance is evaluated with R^2 derived from the regression model $Y \sim \mathrm{PRS} \times \beta$, with β representing the effect of standardized PRS on the standardized outcome (**Methods**). For RICE, R^2 or AUC is derived using a predicted PRS from a linear model containing both RICE-CV and RICE-RV trained on the tuning dataset. Significance of R^2 or AUC was assessed using 10,000 bootstrap resamples of the validation set, testing whether the pairwise difference, ${R^2}_{\mathrm{RICE}} - {R^2}_{\mathrm{Best\ Alternative}}$ or $\mathrm{AUC}_{\mathrm{RICE}} - \mathrm{AUC}_{\mathrm{Best\ Alternative}}$, (per trait–ancestry pair) differs from 0 ($p < 0.05$, **; $p < 0.01$, ***; exact p-values in Source Data). Exact bootstrap p-values and CI bounds are provided in the Source Data file. Source data are provided as a Source Data file.

a) Beta of PRS per SD of ancestry-adjusted PRSs for five binary traits across four ancestral groups from UKB WGS data.

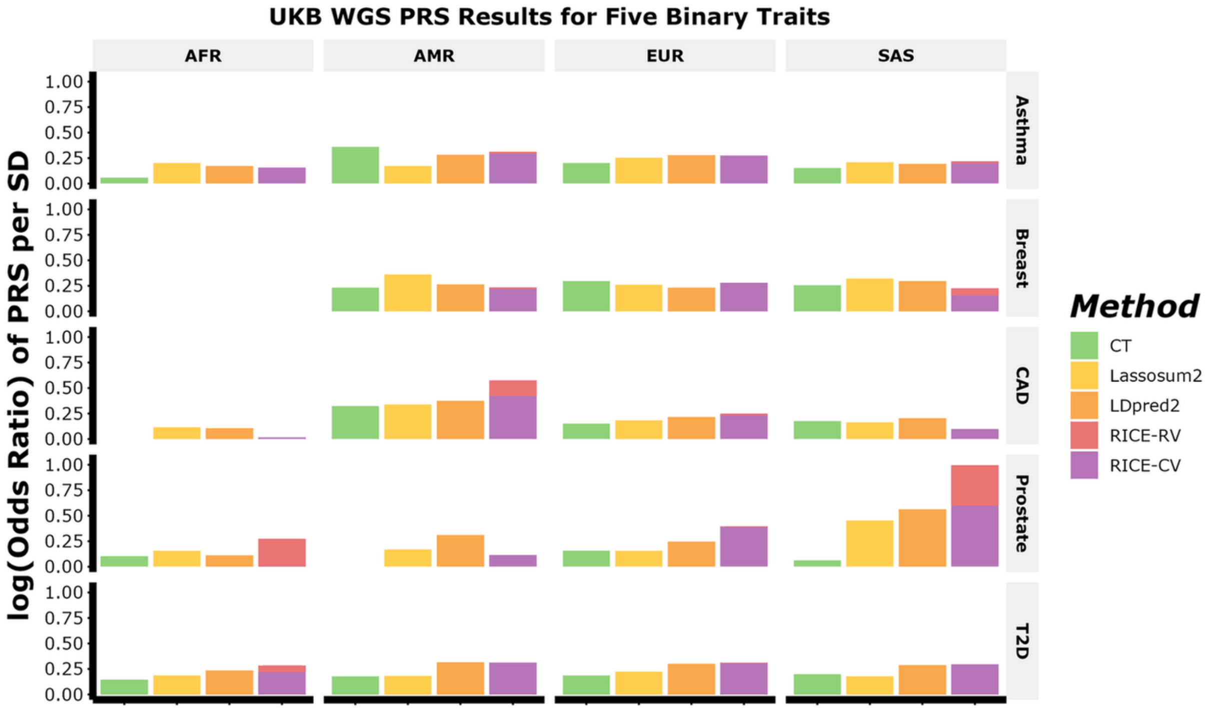

Supplementary Figure 12 continued: b) AUC of ancestry-adjusted PRSs for five binary traits across four ancestral groups from UKB WGS data.

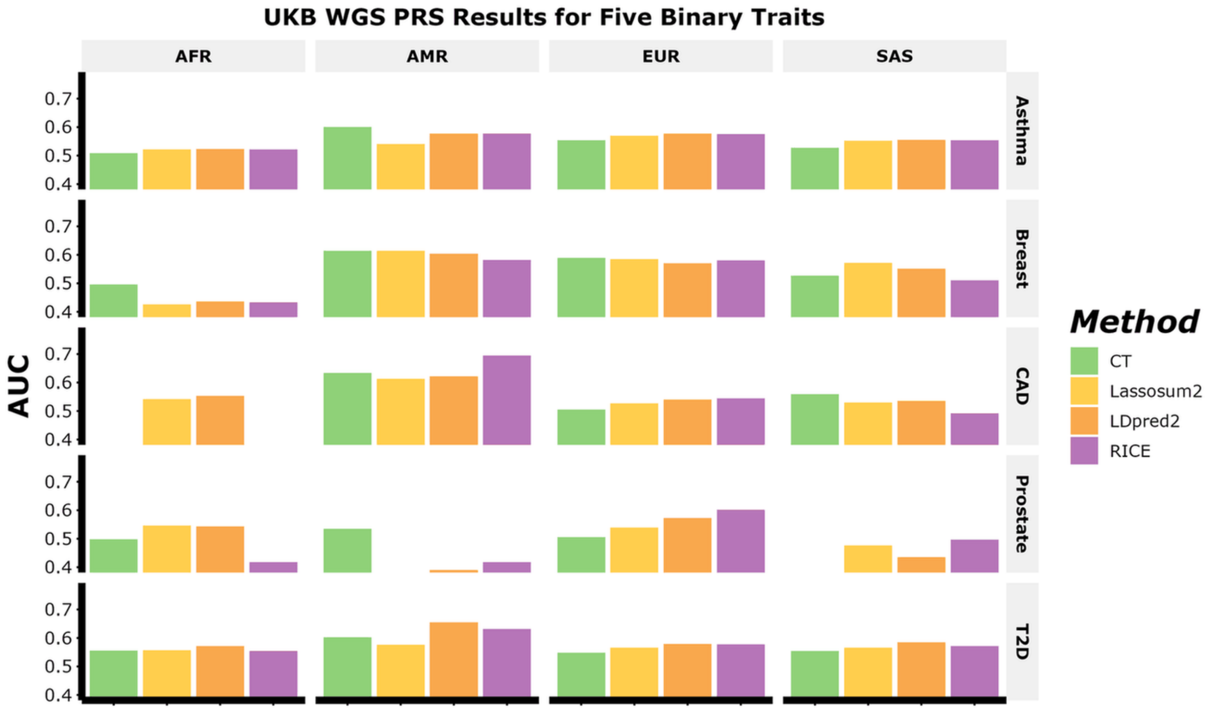

c) Beta of PRS per SD of ancestry-adjusted PRSs for six continuous traits across four ancestral groups from UKB WGS data.

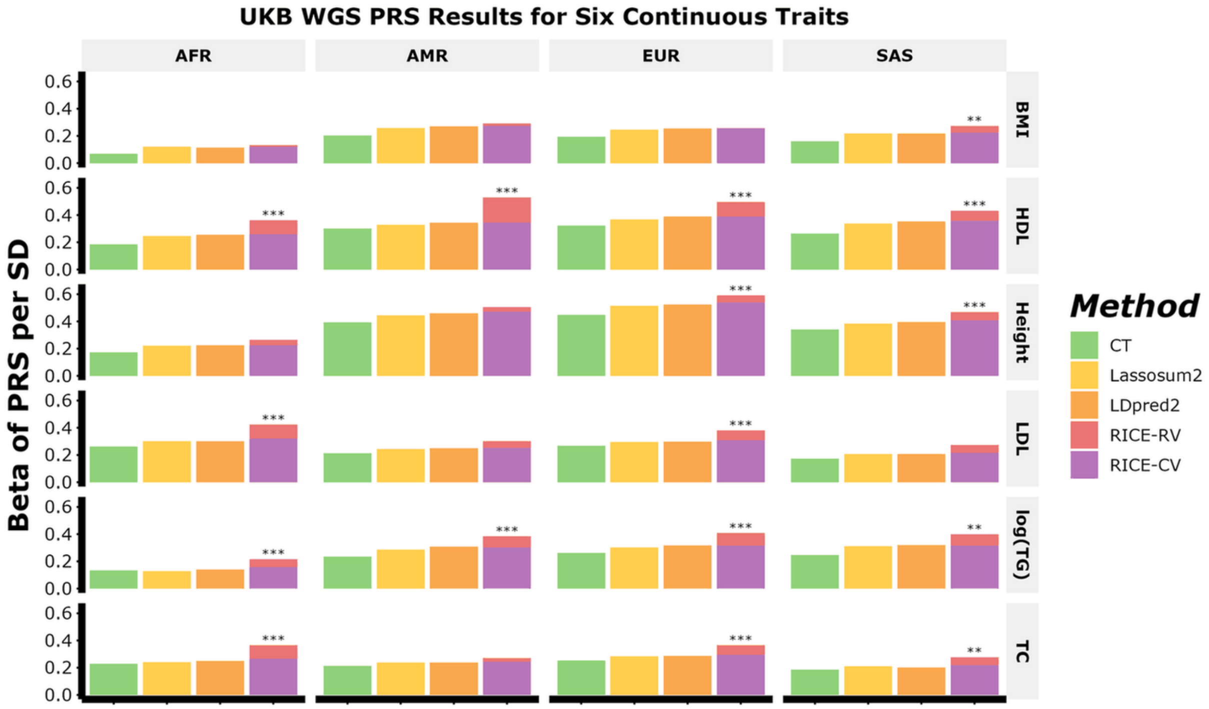

Supplementary Figure 12 continued: d) R^2 of ancestry-adjusted PRSs for six continuous traits across four ancestral groups from UKB WGS data. Percentages above the RICE bars indicate the relative improvement in R^2 achieved by RICE compared to the best alternative method for each ancestry–trait combination.

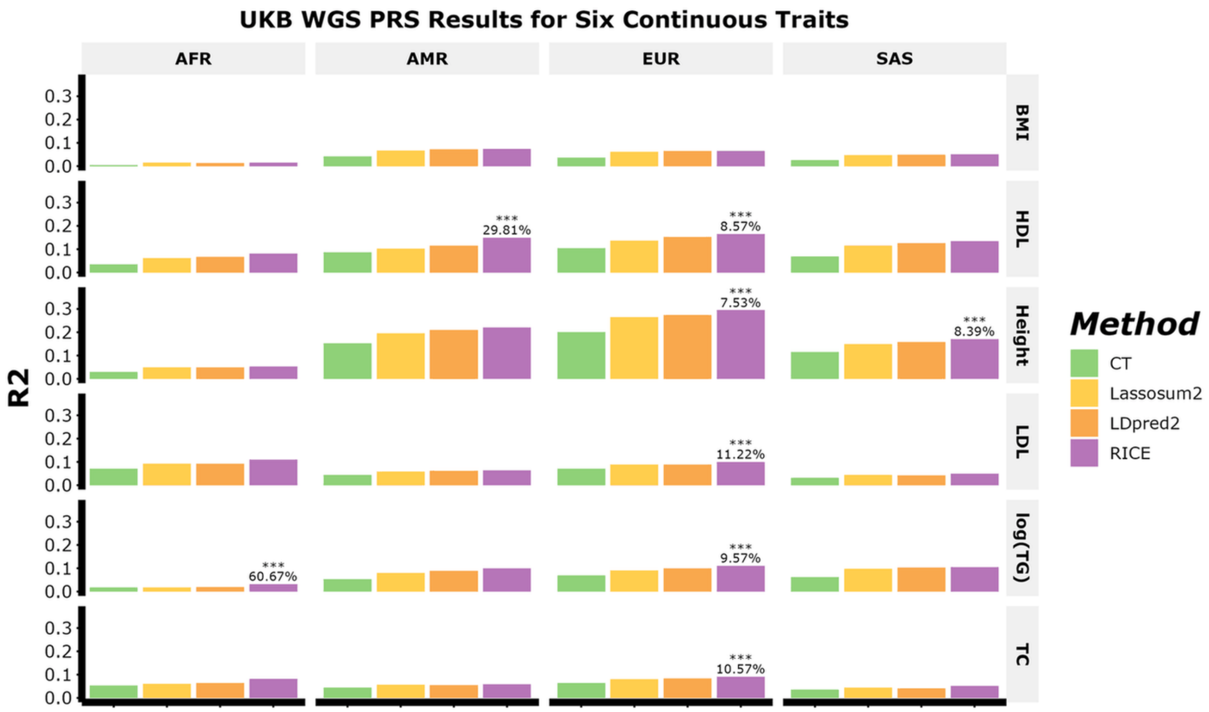

Supplementary Figure 13. Relationship between common and rare variant PRSs and standardized traits across four ancestral groups from UK Biobank (UKB) whole-genome sequencing (WGS) data. The six continuous traits analyzed and shown: body mass index (BMI) (Supp. Fig. 13a), high-density lipoprotein cholesterol (HDL) (Supp. Fig. 13b), height (Supp. Fig. 13c), low-density lipoprotein cholesterol (LDL) (Supp. Fig. 13d), natural logarithm of triglyceride cholesterol (log(TG)) (Supp. Fig. 13e), and total cholesterol (TC) (Supp. Fig. 13f). PRS quantiles for RICE-CV (common variants) are plotted on the x-axis, and standardized trait on the y-axis. Data are stratified by rare variant PRS quantiles from RICE-RV (blue: below 5%, grey: 20–70%, pink: above 95%). Mean $\pm 1 \times$ SE are shown for individuals of African (AFR), Admixed American/Latino (AMR), European (EUR), and South Asian (SAS) ancestries. The training data consisted solely of individuals of European ancestry, while the tuning and validation sets included all four ancestries. Full sample sizes details for each ancestry are provided in **Supplementary Data 3**. Source data are provided as a Source Data file.

a) Relationship between ancestry-adjusted common and rare variant PRSs and standardized body mass index (BMI) levels across four ancestral groups from UKB WGS data.

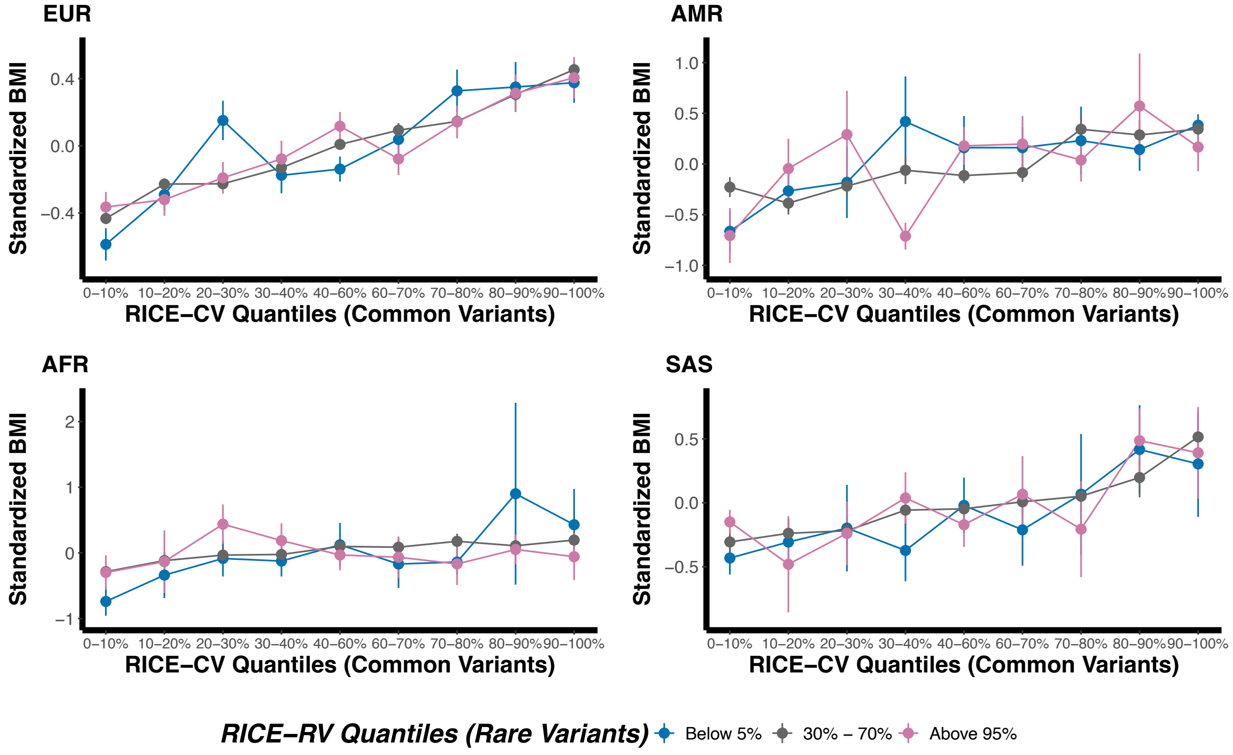

Supplementary Figure 13 continued: b) Relationship between ancestry-adjusted common and rare variant PRSs and standardized high-density lipoprotein cholesterol (HDL) across four ancestral groups from UKB WGS data.

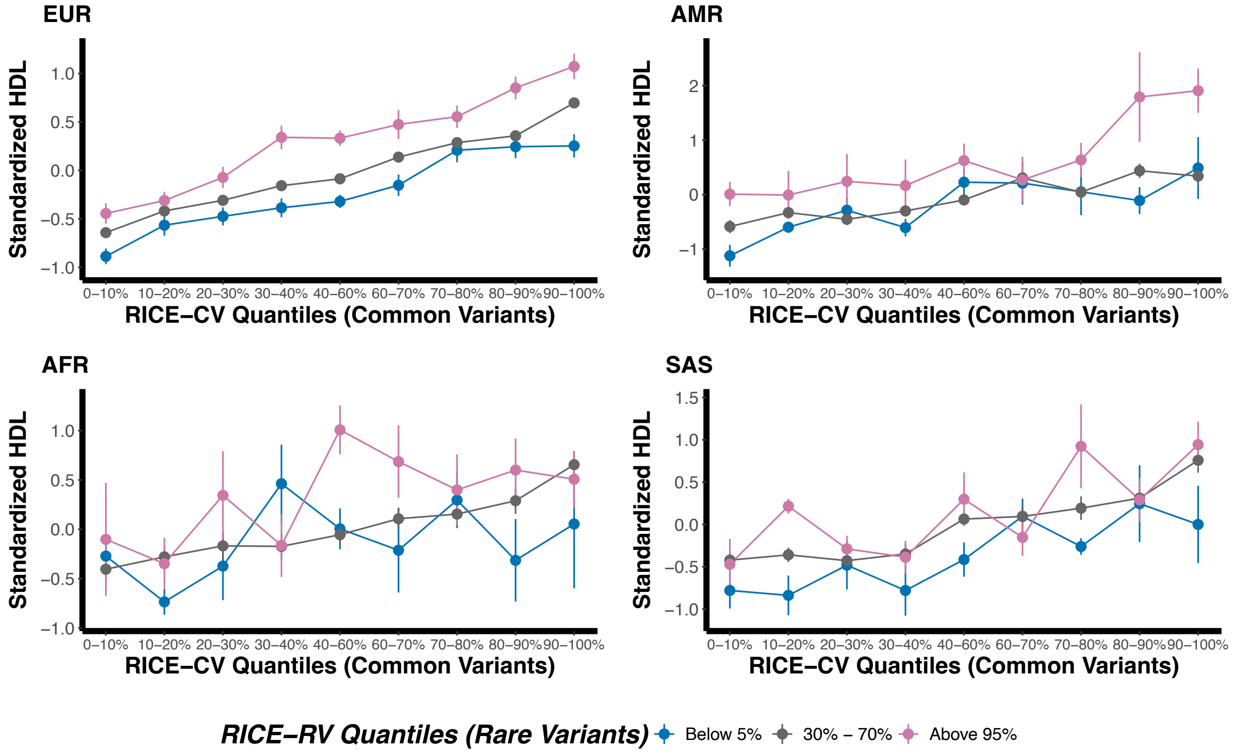

Supplementary Figure 13 continued: c) Relationship between ancestry-adjusted common and rare variant PRSs and standardized height across four ancestral groups from UKB WGS data.

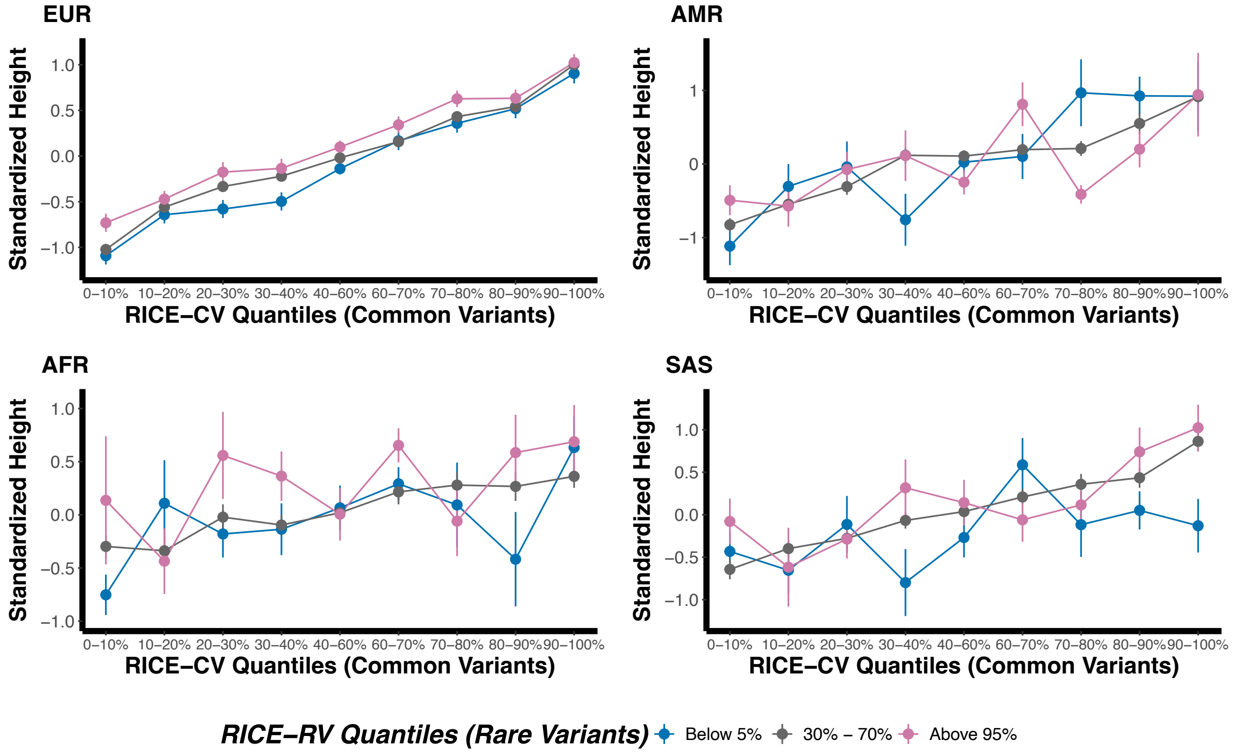

Supplementary Figure 13 continued: d) Relationship between ancestry-adjusted common and rare variant PRSs and standardized low-density lipoprotein cholesterol (LDL) levels across four ancestral groups from UKB WGS data.

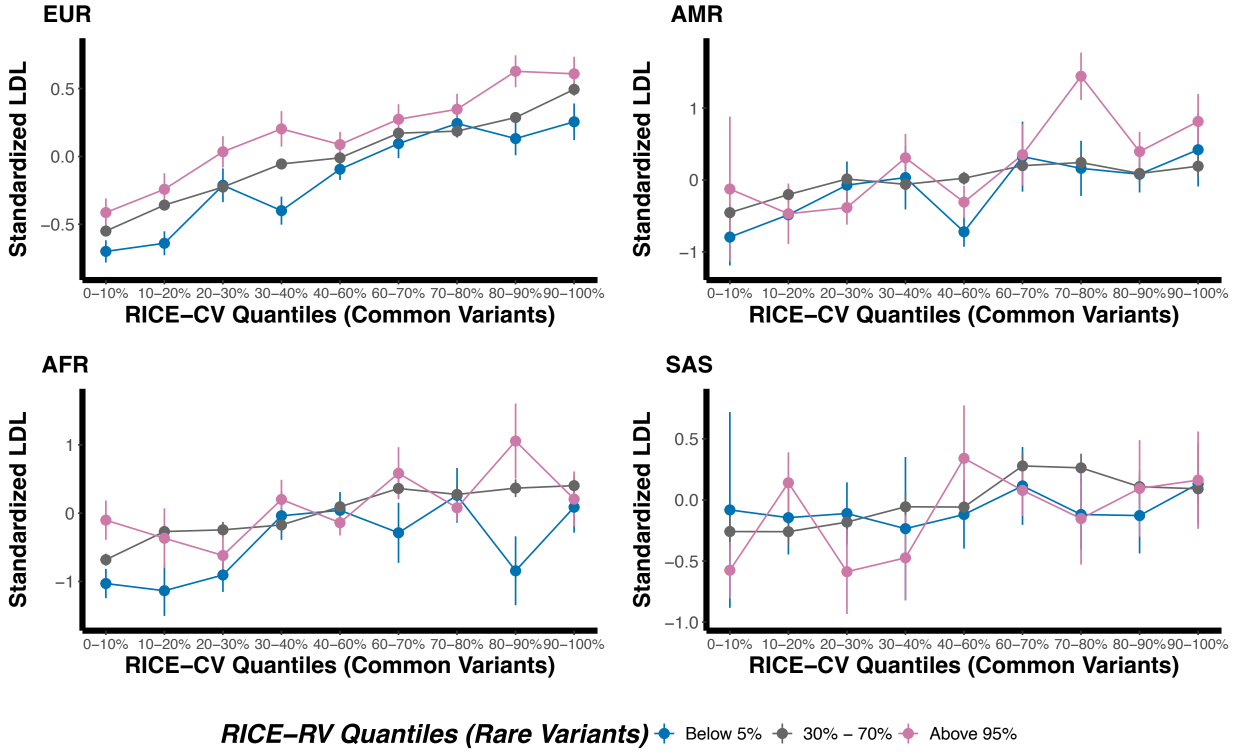

Supplementary Figure 13 continued: e) Relationship between ancestry-adjusted common and rare variant PRSs and standardized natural logarithm of triglyceride cholesterol (log(TG)) levels across four ancestral groups from UKB WGS data.

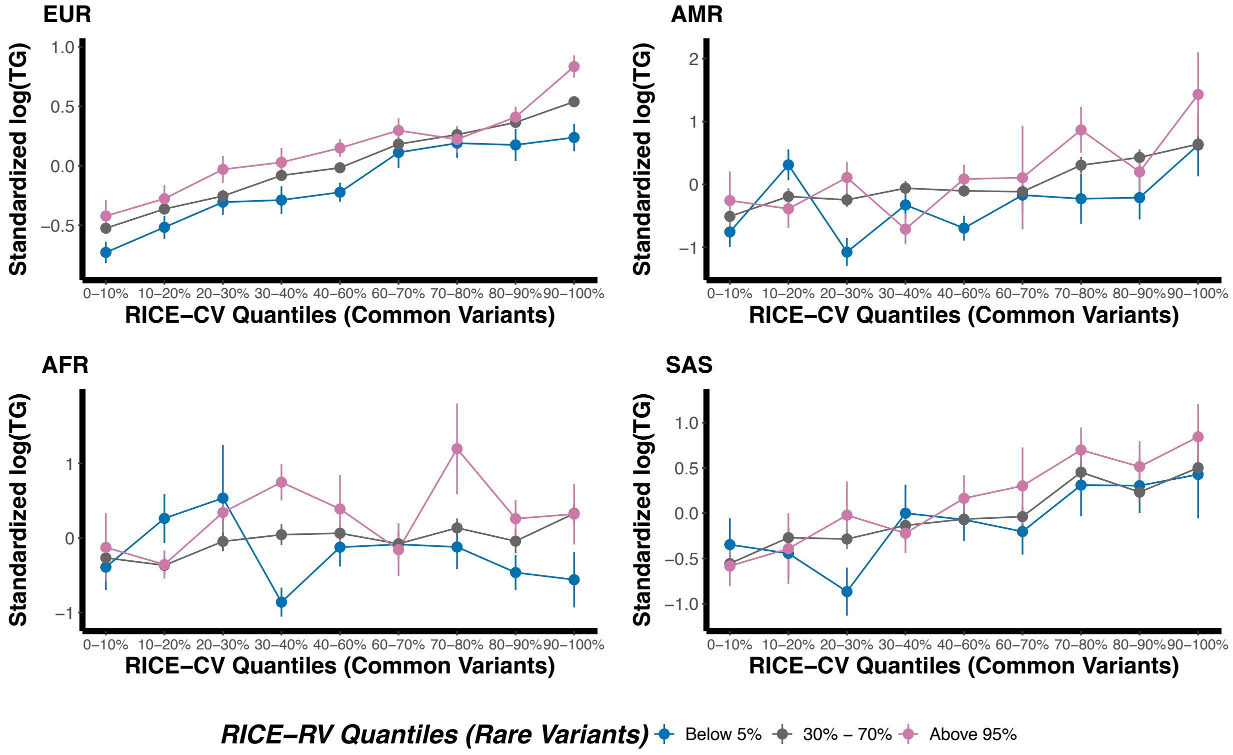

Supplementary Figure 13 continued: f) Relationship between ancestry-adjusted common and rare variant PRSs and standardized total cholesterol (TC) levels across four ancestral groups from UKB WGS data.

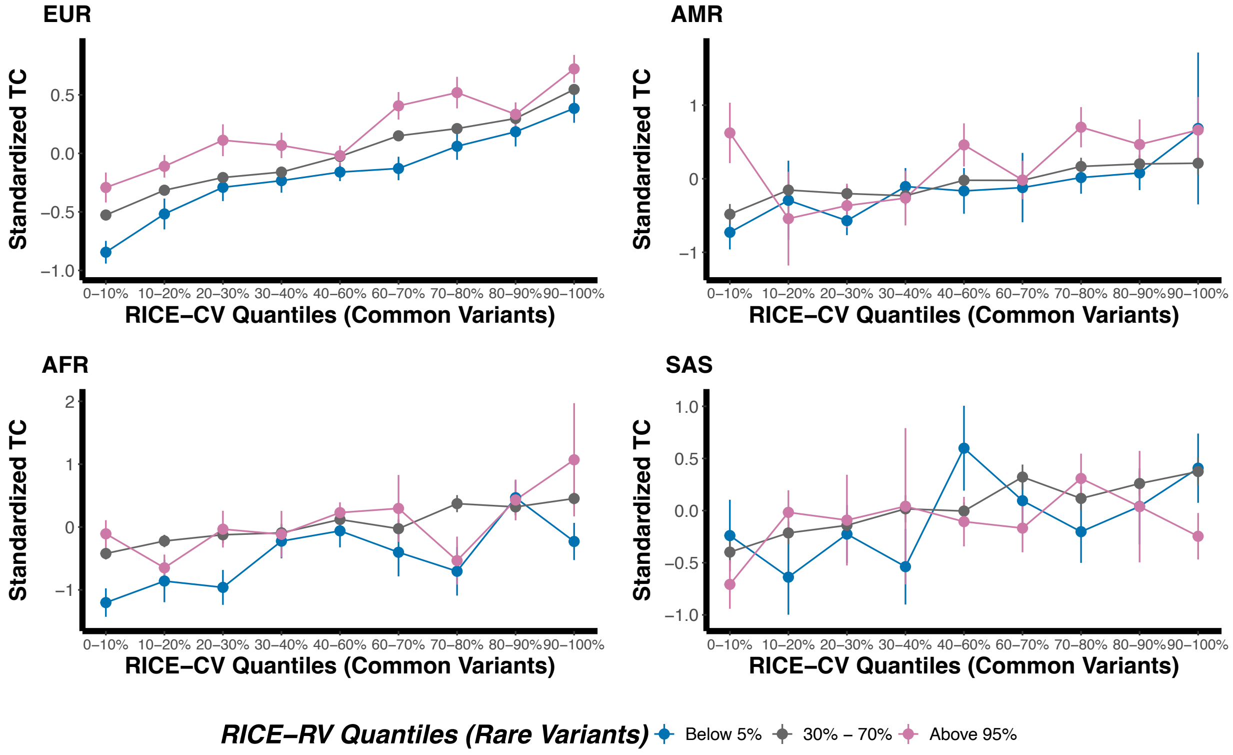

Supplementary Figure 14. Predictive performance of PRSs standardized within genetically-inferred ancestries or using the first five principal components (Methods) for six continuous traits and five binary traits across four ancestral groups from UK Biobank (UKB) whole-genome sequencing (WGS) data. The continuous traits analyzed include body mass index (BMI), high-density lipoprotein cholesterol (HDL), height, low-density lipoprotein cholesterol (LDL), natural logarithm of triglyceride cholesterol (log(TG)), and total cholesterol (TC). The binary traits analyzed include asthma, breast cancer, coronary artery disease (CAD), prostate cancer and type 2 diabetes (T2D). Mean and 95% bootstrap confidence intervals are shown for individuals of African (AFR), Admixed American or Latino (AMR), European (EUR), and South Asian (SAS) ancestries. Source data are provided as a Source Data file.

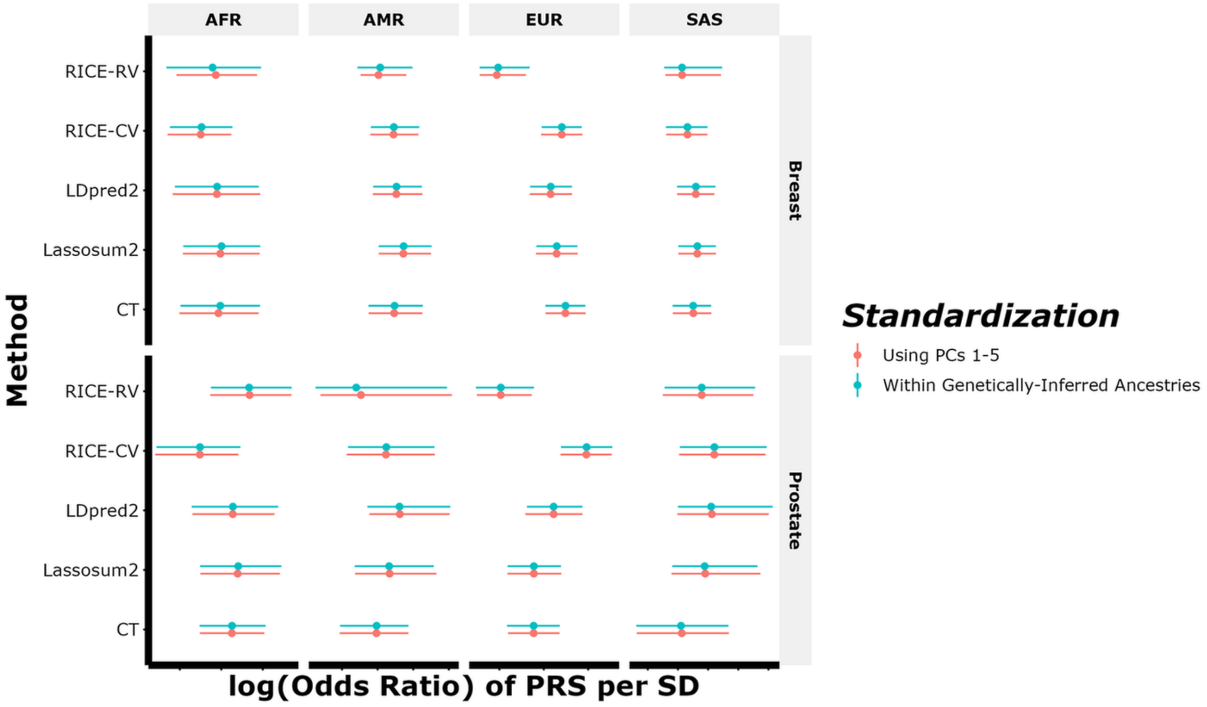

Supplementary Figure 14 continued. Figures for CAD, T2D, and Asthma are included in this page.

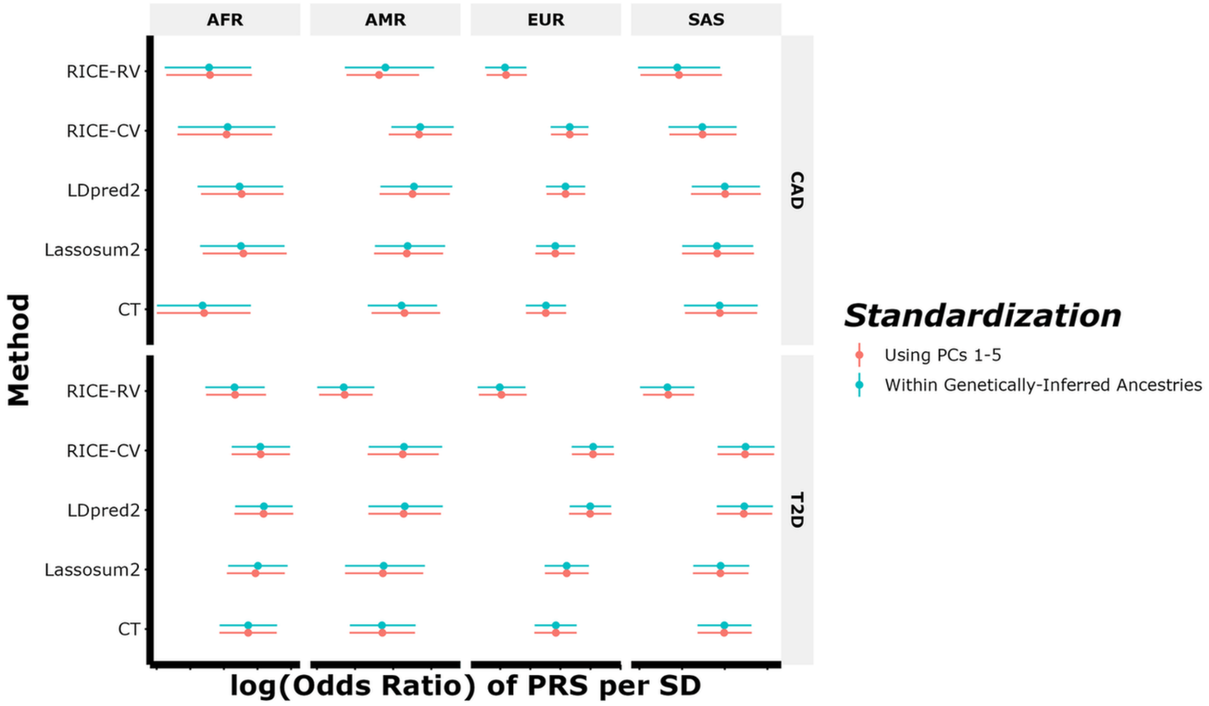

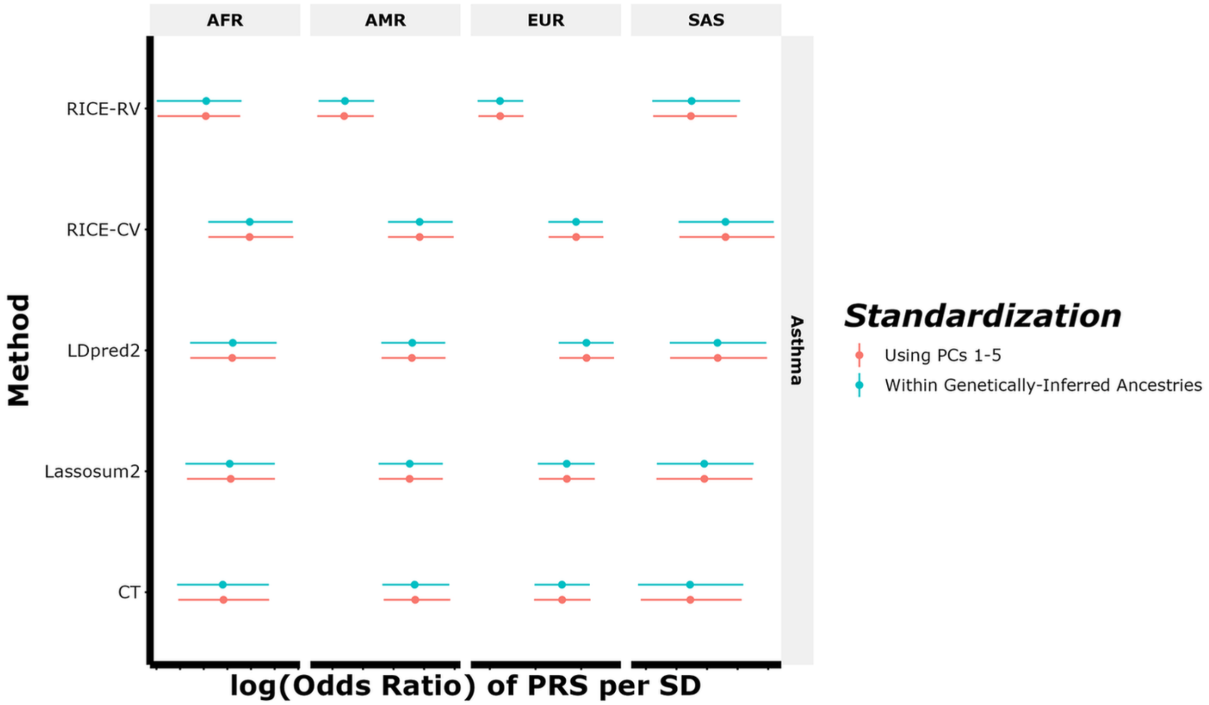

Supplementary Figure 14 continued. Figures for BMI, Height, HDL, and LDL are included in this page.

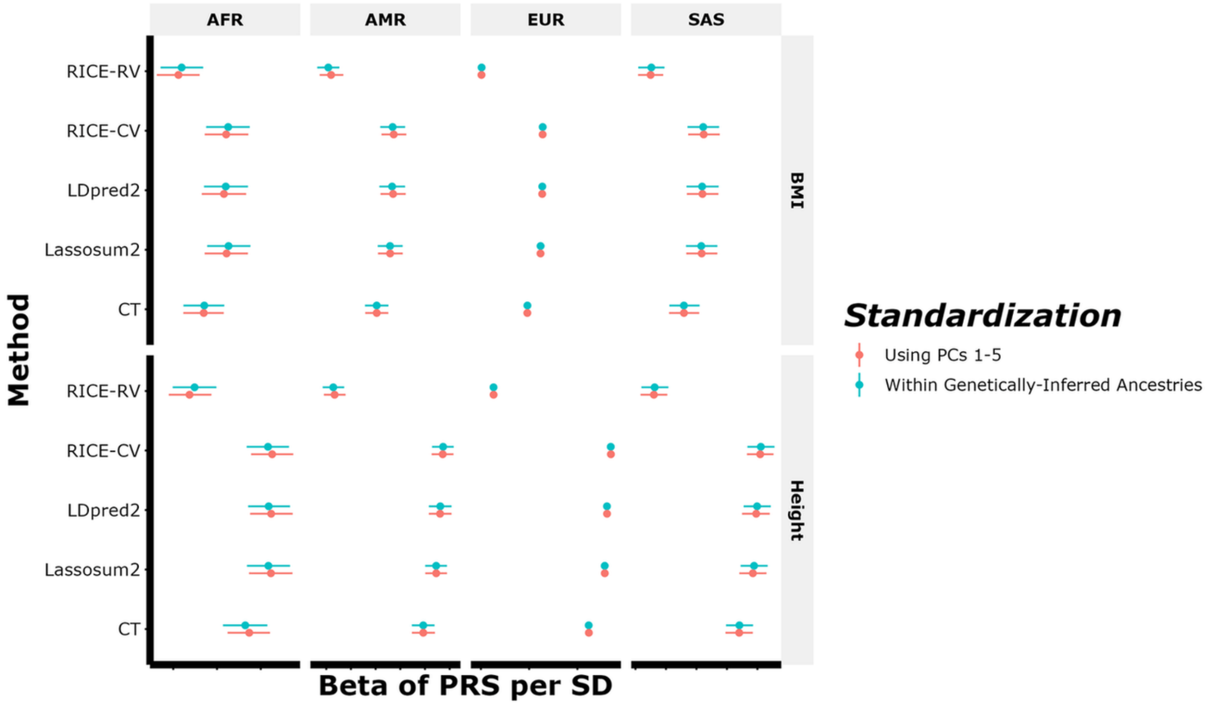

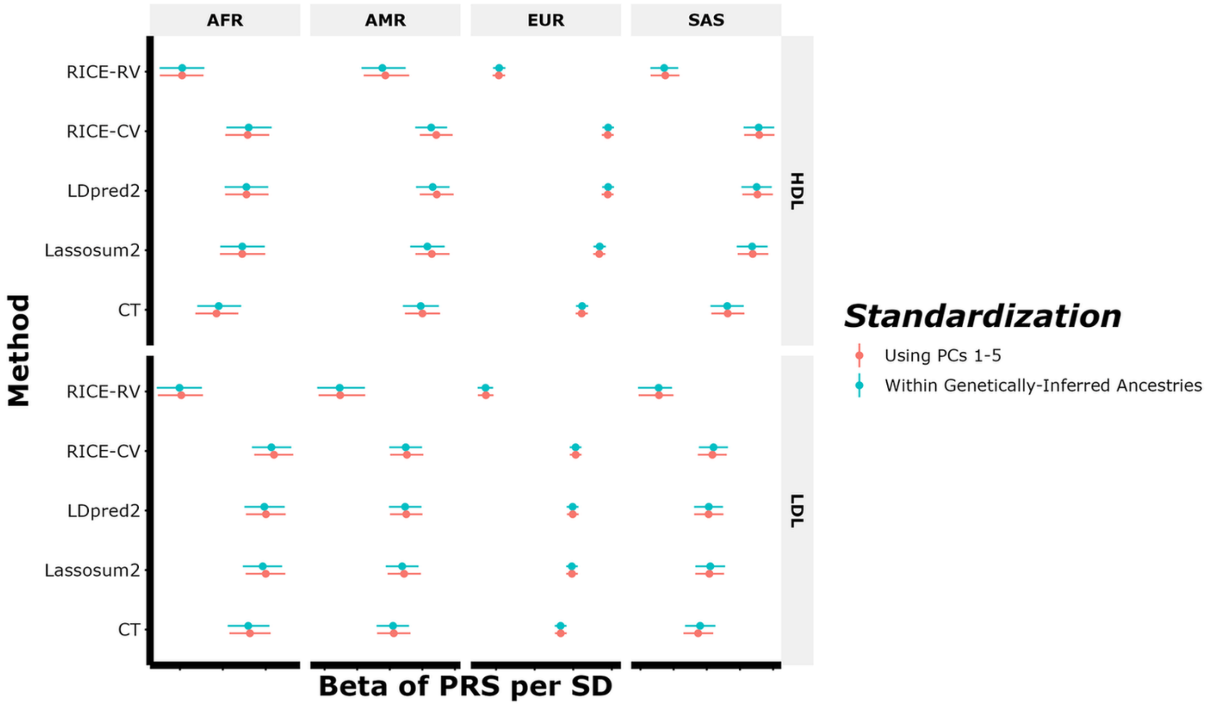

Supplementary Figure 14 continued. Figures for log(TG) and TC are included in this page.

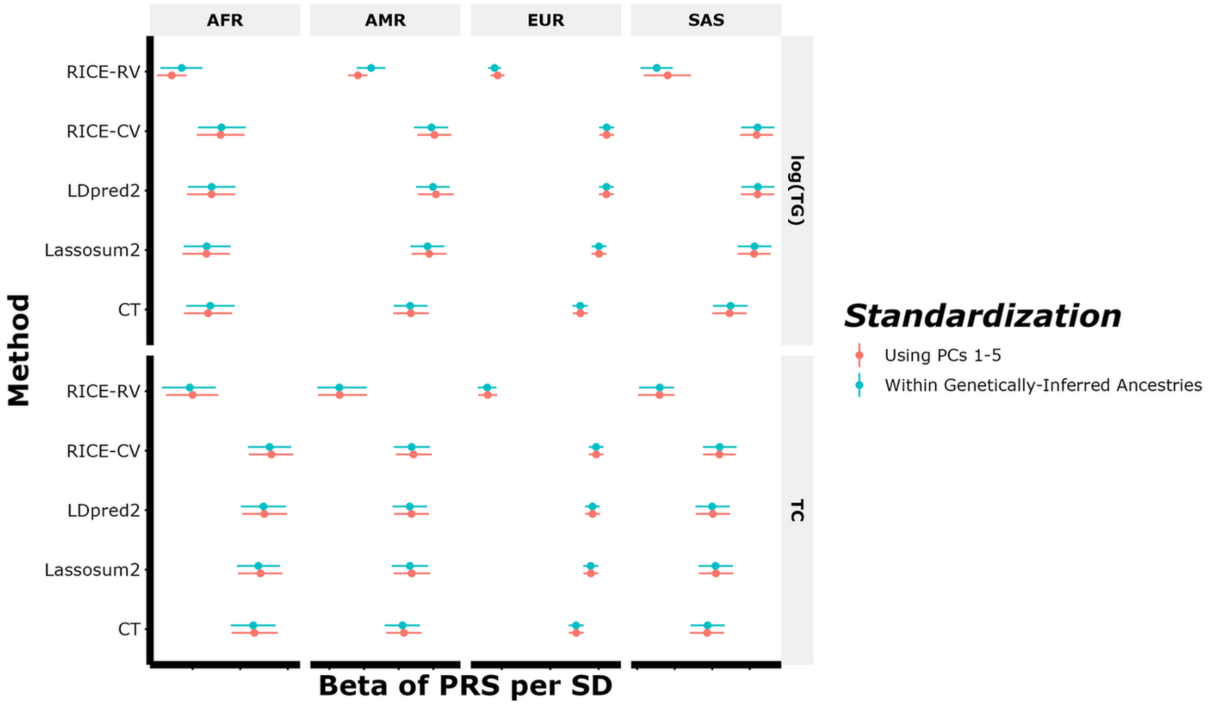

Supplementary Figure 15. Comparison of ancestry-adjusted PRSs from RICE-CV and RICE-RV using UK Biobank (UKB) imputed genotype and WES and whole-genome sequencing (WGS) data for individuals of either African, Admixed American, or South Asian ancestry. Results are shown for six continuous traits: body mass index (BMI), height, high-density lipoprotein cholesterol (HDL), low-density lipoprotein cholesterol (LDL), the natural logarithm of triglyceride (log(TG)), total cholesterol (TC), and five binary traits: asthma, breast cancer, coronary artery disease (CAD), prostate cancer, and type 2 diabetes (T2D). The training data included only individuals of European ancestry, while the tuning and validation sets contained individuals from all four ancestries. Full sample size details for each ancestry are provided in **Supplementary Data 2 and 3**. Separate figures for individuals of African (Supp. Fig. 15a), Admixed American (Supp. Fig. 15b), and South Asian (Supp. Fig. 15c) ancestry are shown below. Source data are provided as a Source Data file.

a) Comparison of ancestry-adjusted PRSs from RICE-CV and RICE-RV using UKB Imputed + WES and WGS data for individuals of African ancestry.

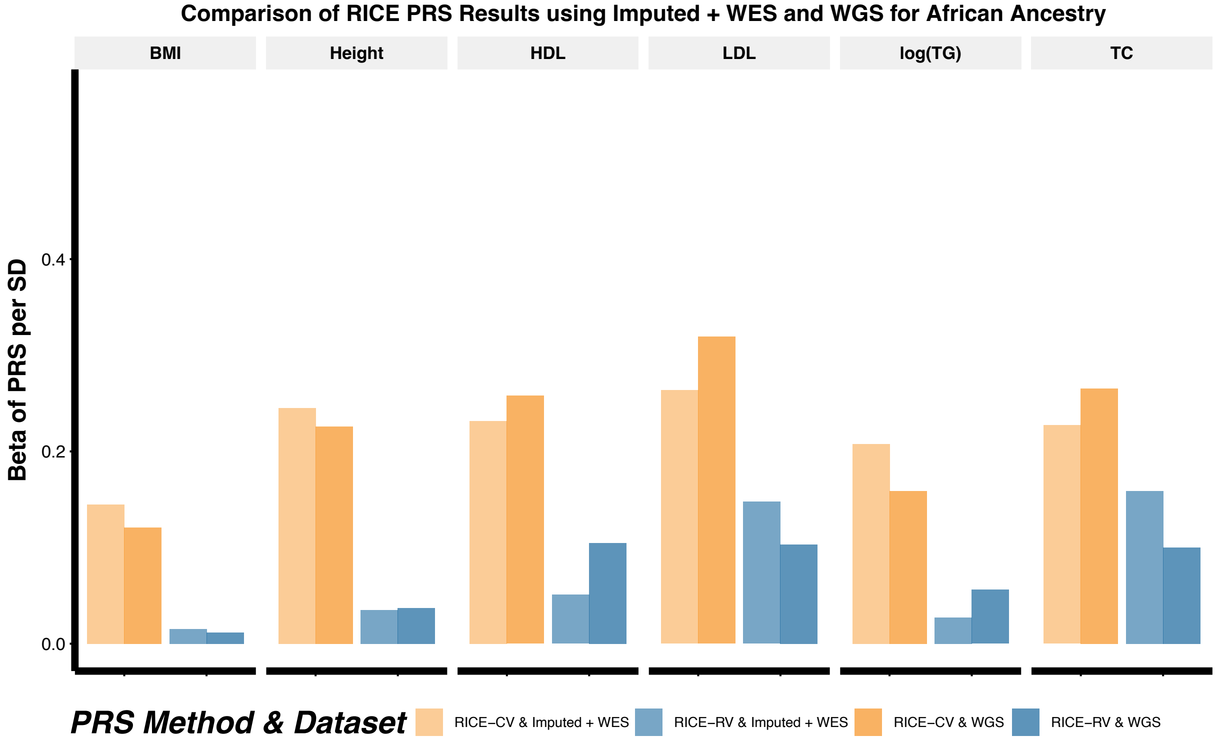

Supplementary Figure 15 continued: b) Comparison of ancestry-adjusted PRSs from RICE-CV and RICE-RV using UKB Imputed + WES and WGS data for individuals of Admixed American ancestry.

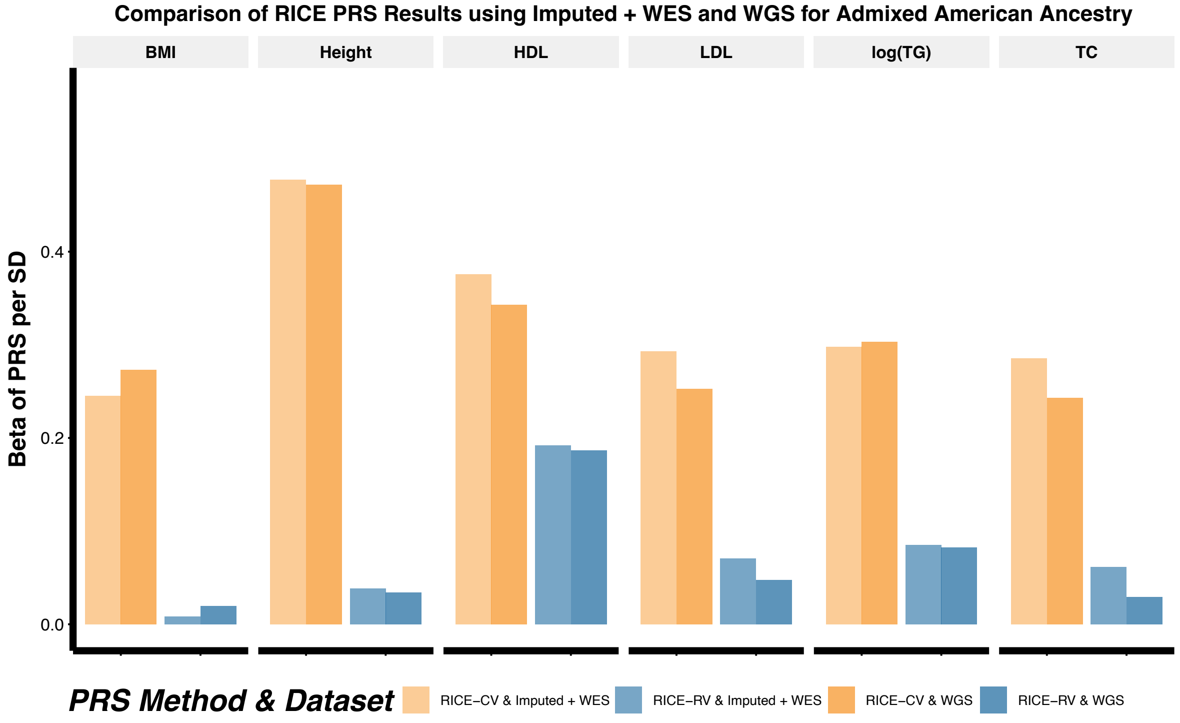

c) Comparison of ancestry-adjusted PRSs from RICE-CV and RICE-RV using UKB Imputed + WES and WGS data for individuals of South Asian ancestry.

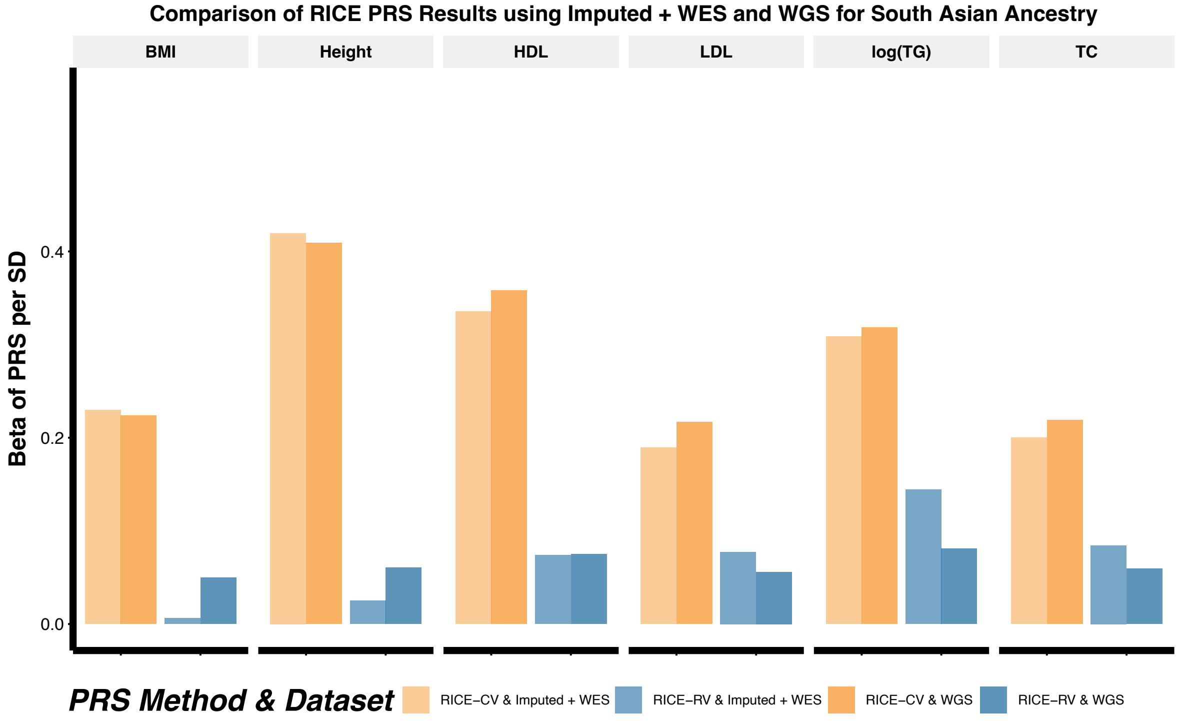

Supplementary Figure 16. Comparison of ancestry-adjusted PRSs from RICE-CV, RICE-RV, RICE-RV constructed with only coding genes (Coding), and RICE-RV constructed using only noncoding genes (Noncoding) using UK Biobank (UKB) whole-genome sequencing (WGS) data for individuals of either African, Admixed American, or South Asian ancestry. Results are shown for five binary traits (Supp. Fig. 16a): asthma, breast cancer, coronary artery disease (CAD), prostate cancer, and type 2 diabetes (T2D) and six continuous traits (Supp. Fig. 16b): body mass index (BMI), height, high-density lipoprotein cholesterol (HDL), low-density lipoprotein cholesterol (LDL), the natural logarithm of triglyceride (log(TG)), total cholesterol (TC). The training data included only individuals of European ancestry, while the tuning and validation sets contained individuals from all four ancestries. Data is plotted by ancestry; African (AFR), Admixed American or Latino (AMR), European (EUR), and South Asian (SAS). Full sample size details for each ancestry are provided in **Supplementary Data 3.** Source data are provided as a Source Data file.

a) Comparison of ancestry-adjusted PRSs from RICE-CV, RICE-RV, RICE-RV constructed with only coding genes (Coding), and RICE-RV constructed using only noncoding genes (Noncoding) using UKB WGS data for five binary traits.

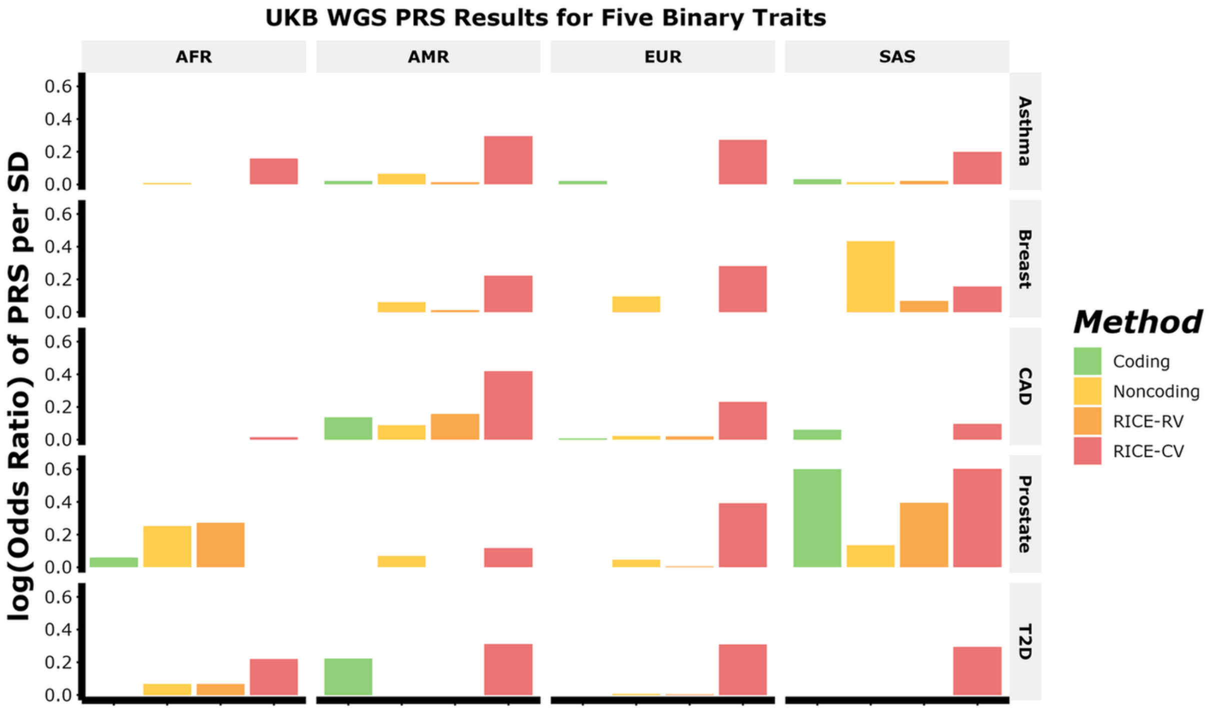

Supplementary Figure 16 continued: b) Comparison of ancestry-adjusted PRSs from RICE-CV, RICE-RV, RICE-RV constructed with only coding genes (Coding), and RICE-RV constructed using only noncoding genes (Noncoding) using UKB WGS data for six continuous traits.

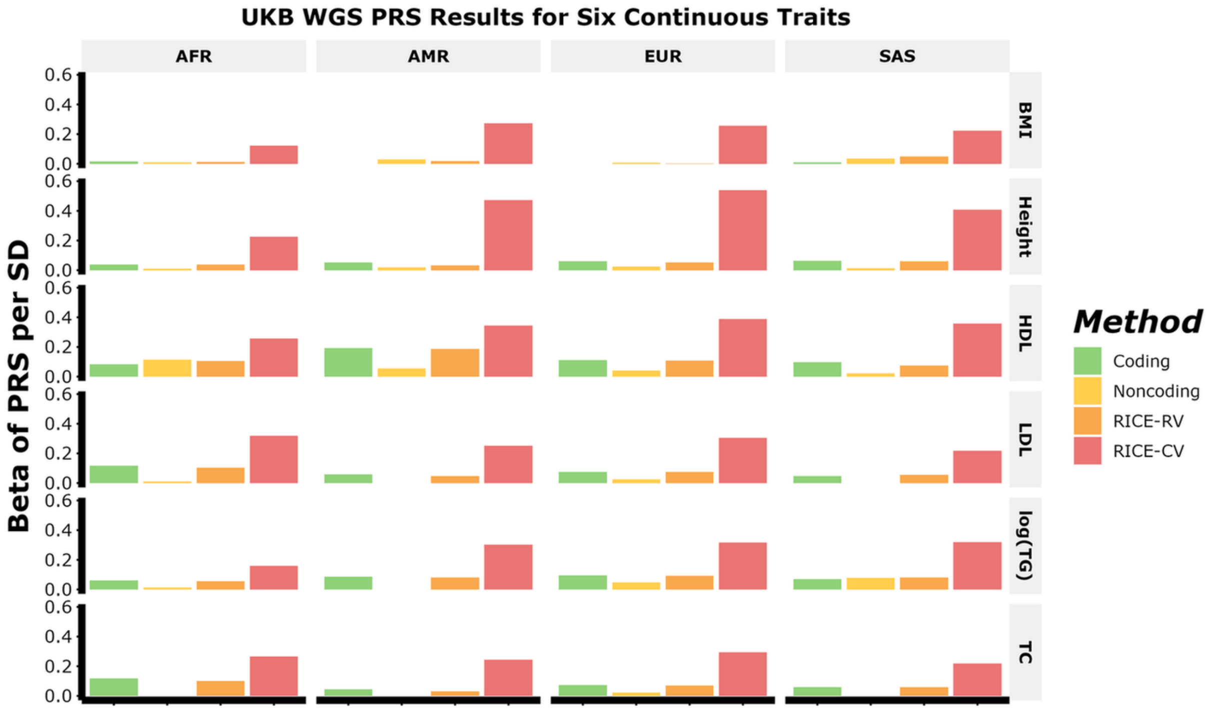

Supplementary Figure 17. Manhattan plot and QQ plots based on the All of Us (AoU) GWAS summary statistics computed using the training set consisting of individuals of either African (AFR), Admixed American (AMR), or European (EUR) ancestries for six continuous traits: body mass index (BMI), high-density lipoprotein cholesterol (HDL), height, low-density lipoprotein cholesterol (LDL), natural logarithm of triglycerides (log(TG)), and total cholesterol (TC). The red and blue shaded regions around the diagonal line in the QQ plots indicate the 95% confidence intervals expected under the null hypothesis of no association between genetic markers and the trait of interest, for minor allele frequencies (MAF) within the ranges (0.05, 0.5] and [0.01, 0.05], respectively. The jth order statistic follows a $\mathrm{Beta}(j, N-j+1)$ distribution, where N is the total number of variants given a specific MAF cutoff. For binary traits, λ_{1000} scales λ to a study with 1000 cases and 1000 controls using $\lambda_{1000} = 1 + 1000 \times (\lambda - 1) \times \left(\frac{1}{N_{\mathrm{case}}} + \frac{1}{N_{\mathrm{control}}}\right)$. Genomic control factors are shown in **Supplementary Data 4**. Source data are provided as a Source Data file.

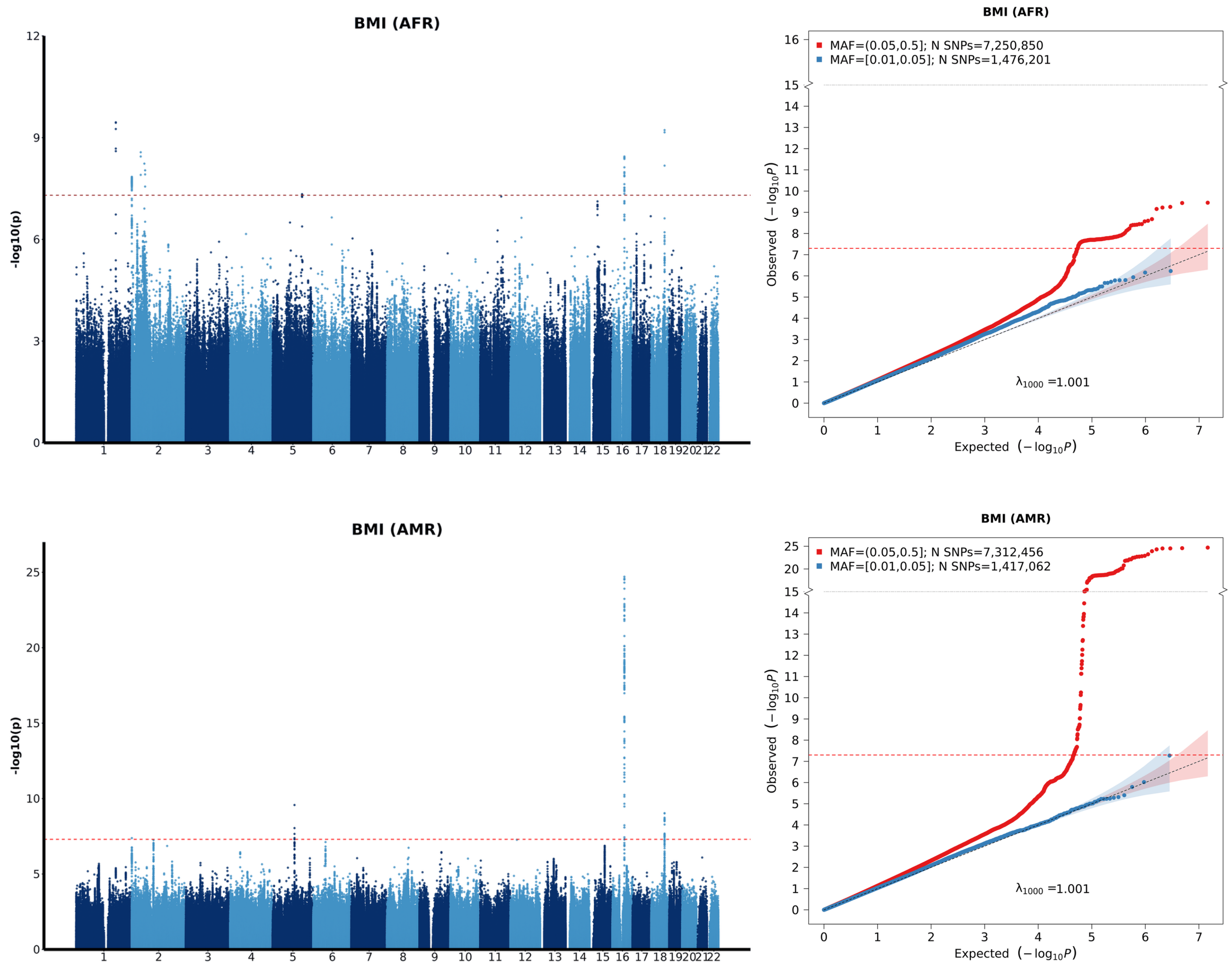

Supplementary Figure 17 continued. Manhattan and QQ Plots for BMI (EUR), HDL (AFR, AMR) based on All of Us GWAS summary statistics.

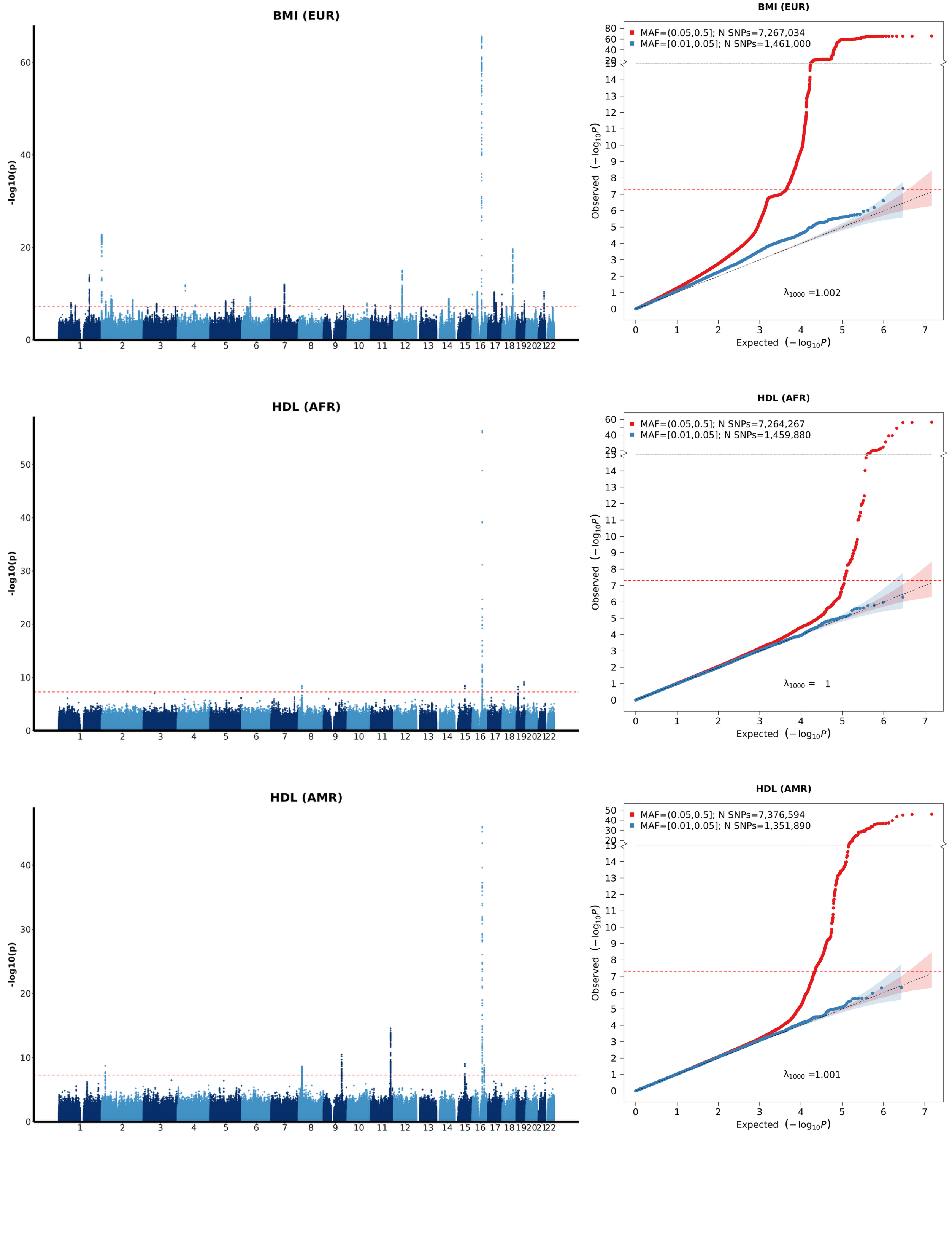

Supplementary Figure 17 continued. Manhattan and QQ Plots for HDL (EUR), height (AFR, AMR) based on All of Us GWAS summary statistics.

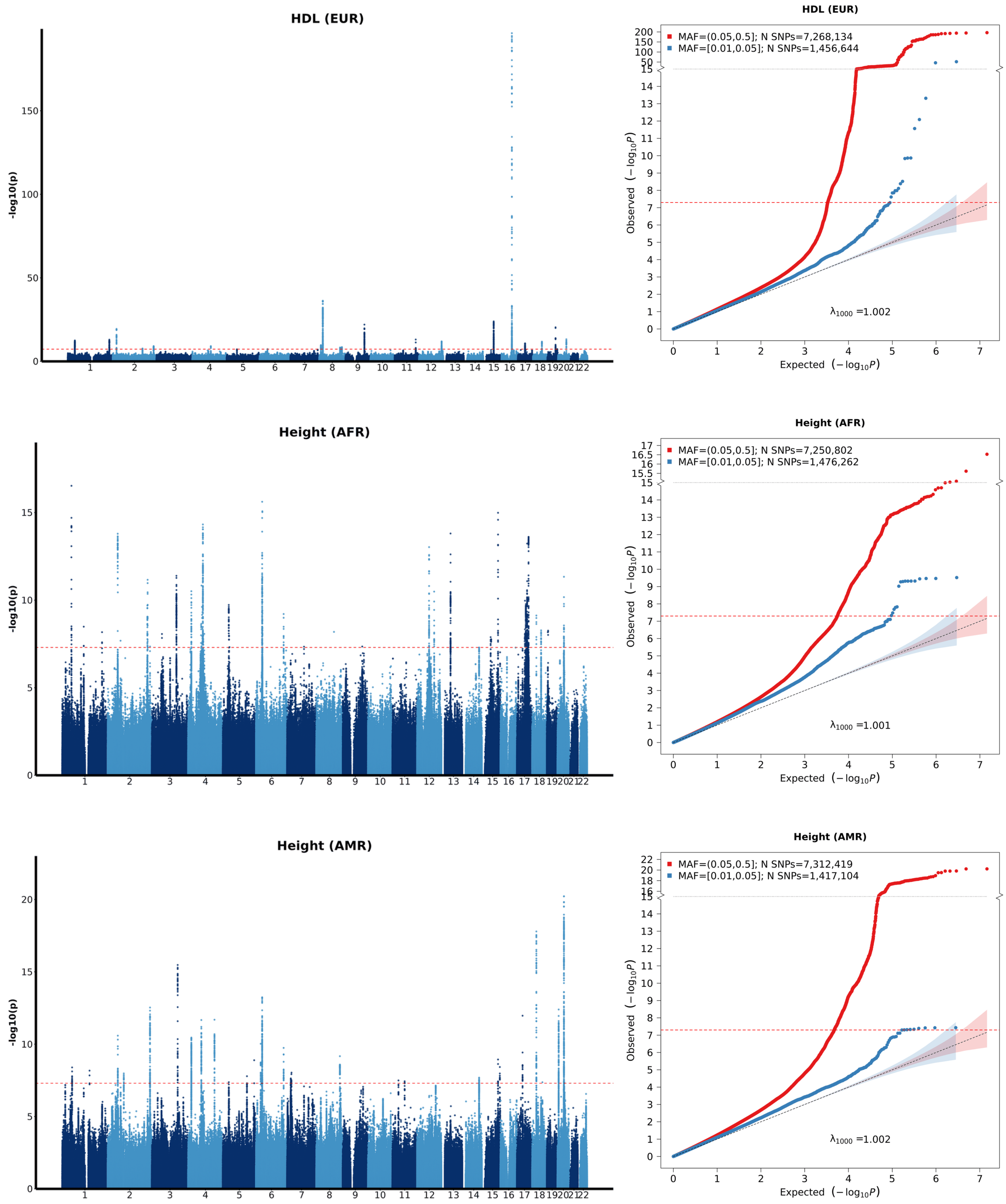

Supplementary Figure 17 continued. Manhattan and QQ Plots for height (EUR), LDL (AFR, AMR) based on All of Us GWAS summary statistics.

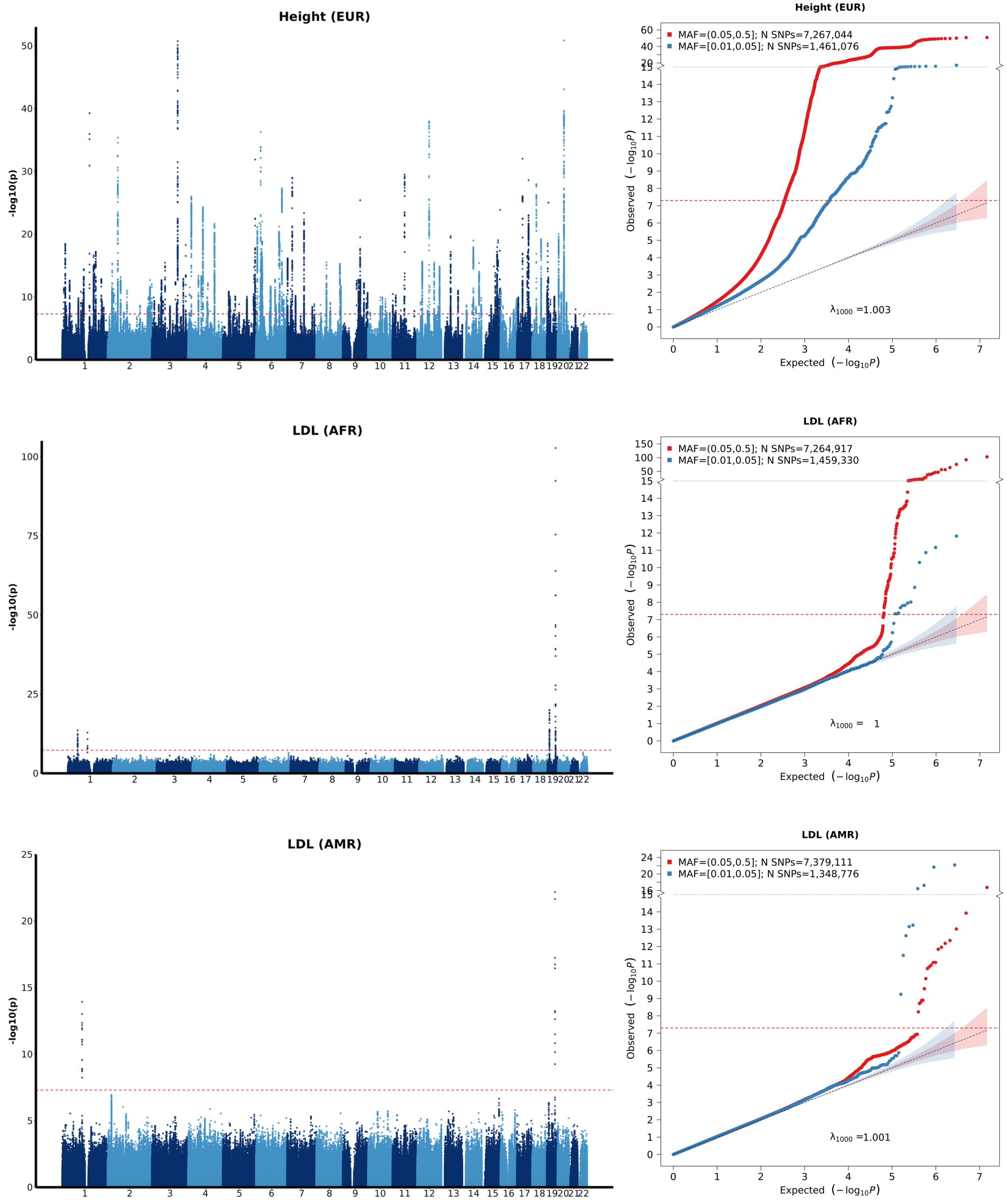

Supplementary Figure 17 continued. Manhattan and QQ Plots for LDL (EUR), log(TG) (AFR, AMR) based on All of Us GWAS summary statistics.

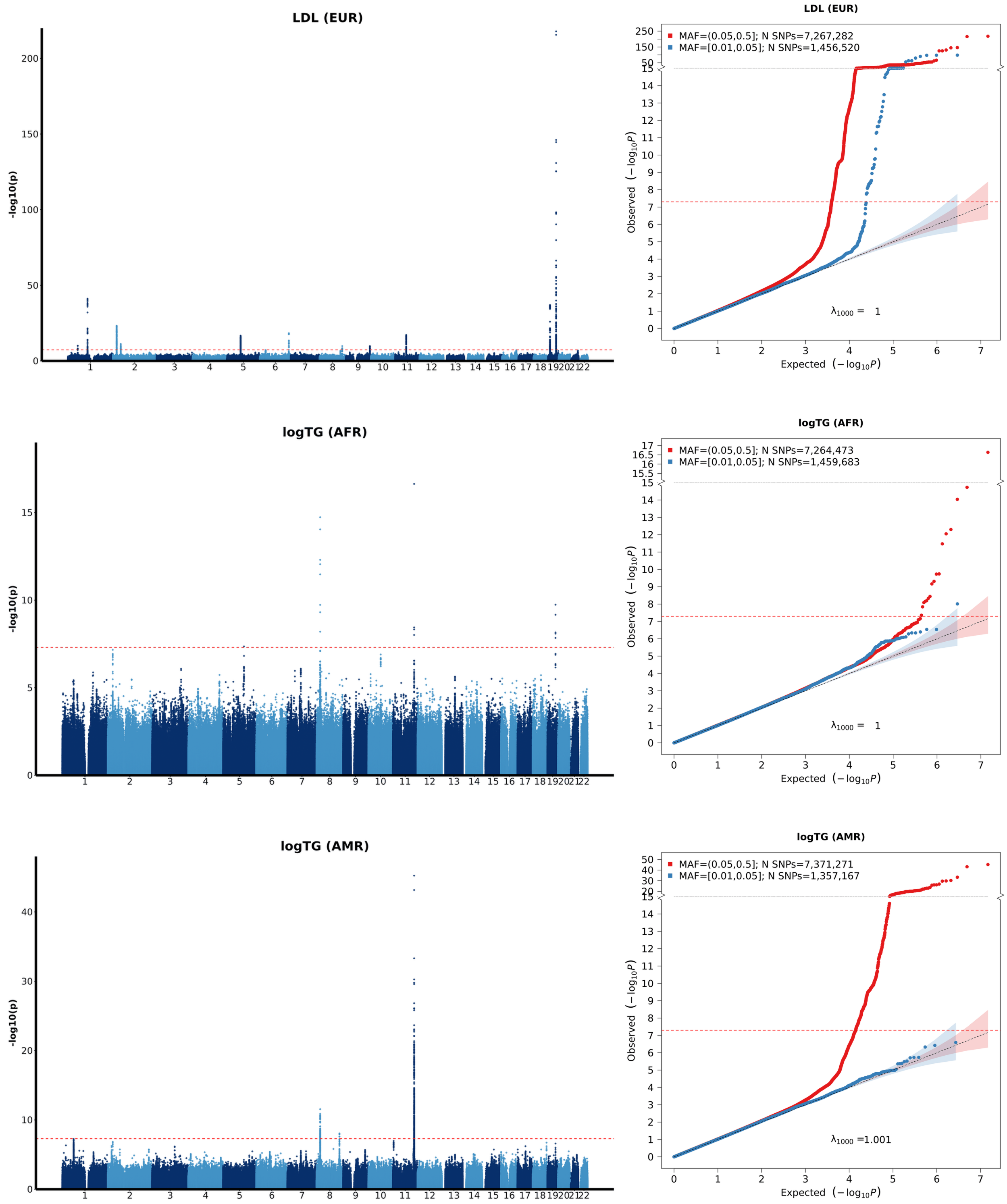

Supplementary Figure 17 continued. Manhattan and QQ Plots for log(TG) (EUR), TC (AFR, AMR) based on All of Us GWAS summary statistics.

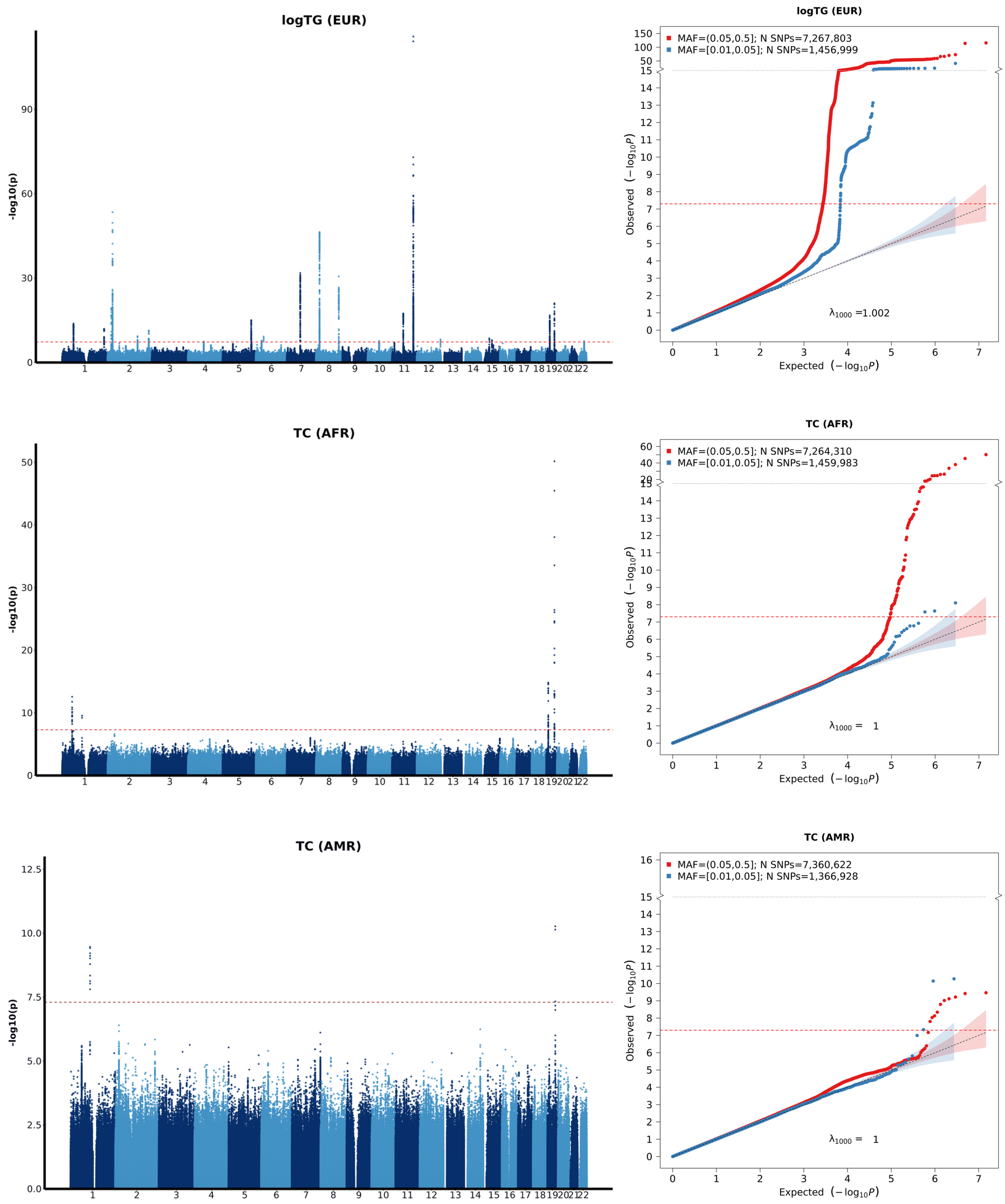

Supplementary Figure 17 continued. Manhattan and QQ Plots for TC (EUR) based on All of Us GWAS summary statistics.

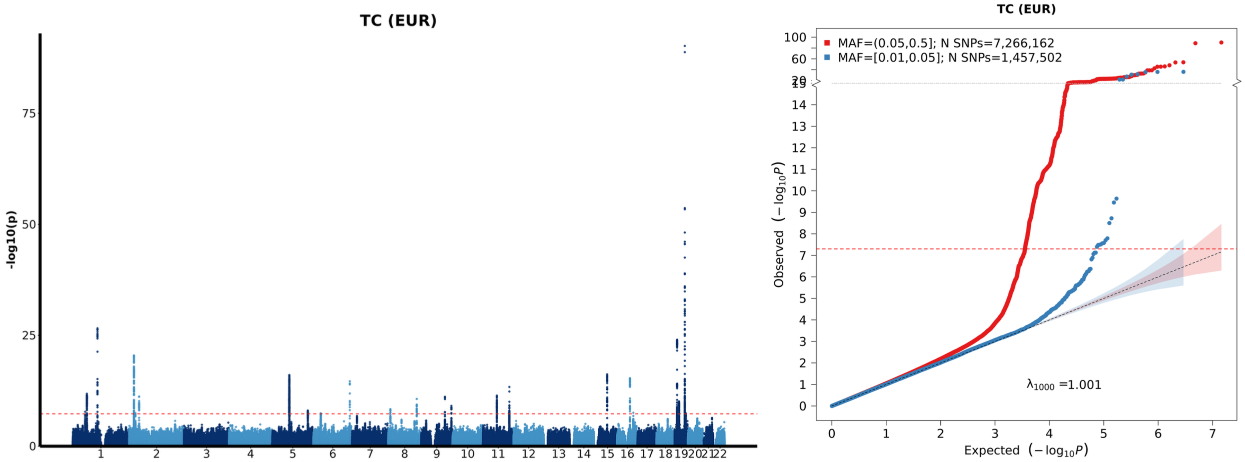

Supplementary Figure 18. QQ plots based on the All of Us rare variant association analysis for coding genes conducted with STAARpipeline using the training set consisting of only individuals of European ancestry (EUR) for training set consisting of individuals of either African (AFR), Admixed American (AMR), or European (EUR) ancestry for six continuous traits: body mass index (BMI), high-density lipoprotein cholesterol (HDL), height, low-density lipoprotein cholesterol (LDL), natural logarithm of triglycerides (log(TG)), and total cholesterol (TC) (sample sizes provided in **Supplementary Data 5**). P-values are split into the five functional categories investigated in the gene-centric coding analysis in the STAARpipeline: putative loss of function (pLoF), putative loss of function and disruptive (pLoF+D), missense, disruptive missense, and synonymous. Under the null hypothesis, the p-value follows a $\mathrm{Uniform}(0,1)$ distribution. Source data are provided as a Source Data file.

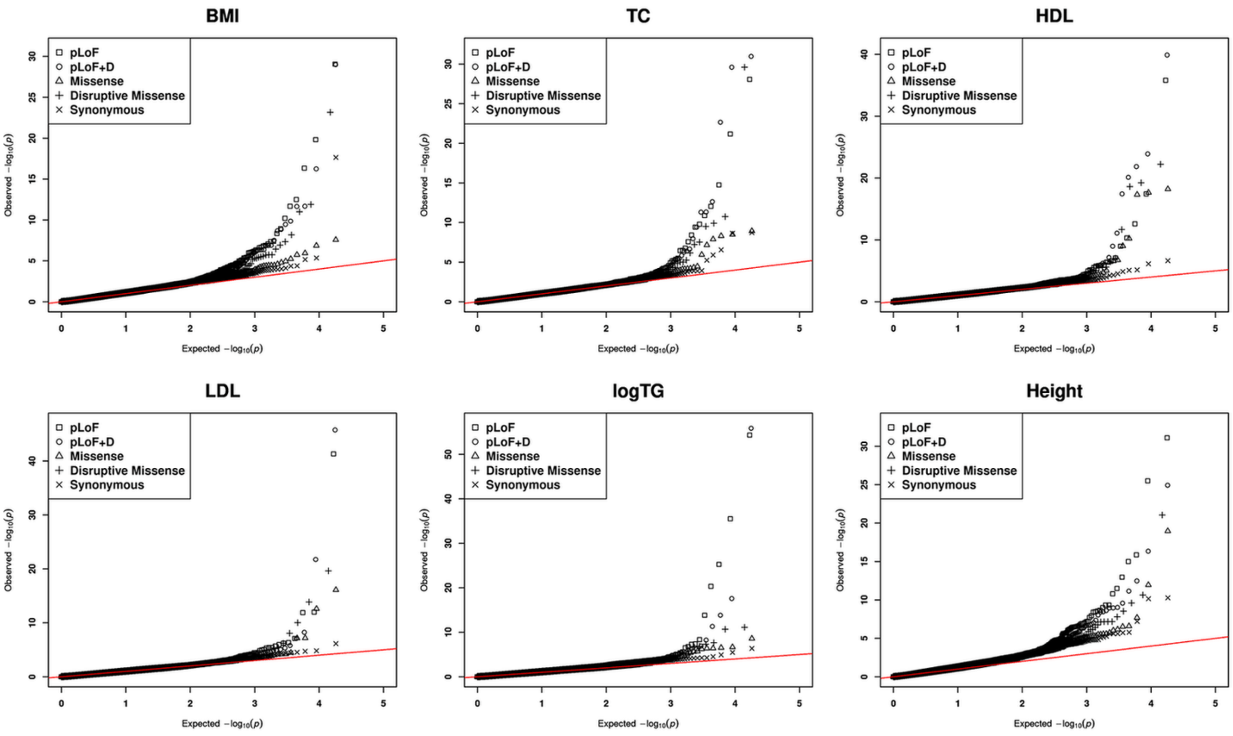

Supplementary Figure 19. Predictive performance of ancestry-adjusted PRSs for six continuous traits across six ancestral groups from All of Us (AoU). The six continuous traits analyzed include body mass index (BMI), height, high-density lipoprotein cholesterol (HDL), low-density lipoprotein cholesterol (LDL), the natural logarithm of triglyceride cholesterol (log(TG)), and total cholesterol (TC). Results are shown for individuals of African (AFR), Admixed American/Latino (AMR), East Asian (EAS), European (EUR), Middle Eastern (MID), and South Asian (SAS) ancestries. The training data consisted of individuals of African, Admixed American, and European ancestries, while the tuning and validation sets included all six ancestries. Full sample sizes details for each ancestry are provided in **Supplementary Data 5**. PRS performance is evaluated with R^2 derived from the regression model $Y \sim \mathrm{PRS} \times \beta$, with β representing the effect of standardized PRS on the standardized outcome (**Methods**). For RICE, R^2 is derived using a predicted PRS from a linear model containing both RICE-CV and RICE-RV trained on the tuning dataset. Significance of R^2 was assessed using 10,000 bootstrap resamples of the validation set, testing whether the pairwise difference, ${R^2}_{\mathrm{RICE}} - {R^2}_{\mathrm{Best\ Alternative}}$(per trait–ancestry pair) differs from 0 ($p < 0.05$, **; $p < 0.01$, ***; exact p-values in Source Data). Exact bootstrap p-values and CI bounds are provided in the Source Data file. Source data are provided as a Source Data file.

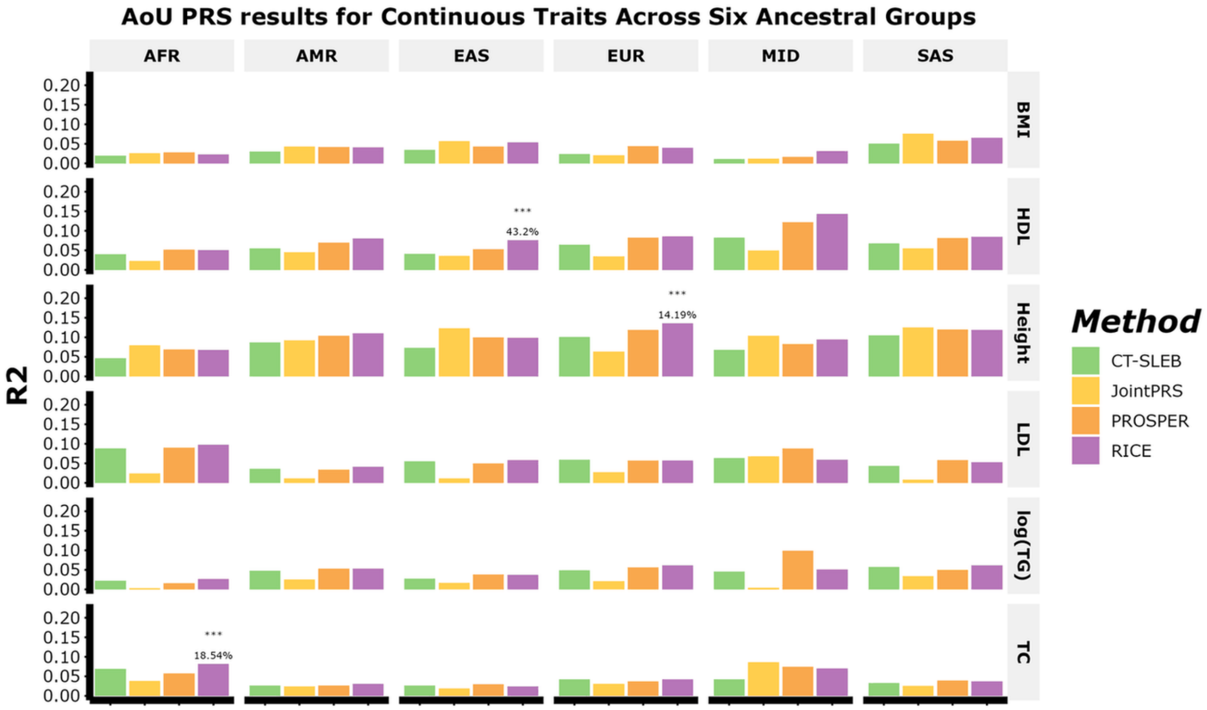

Supplementary Figure 20. Relationship between common and rare variant PRSs and standardized traits across six ancestral groups from All of Us (AoU) data. The six continuous traits analyzed and shown: body mass index (BMI) (Supp. Fig. 20a), high-density lipoprotein cholesterol (HDL) (Supp. Fig. 20b), height (Supp. Fig. 20c), low-density lipoprotein cholesterol (LDL) (Supp. Fig. 20d), natural logarithm of triglyceride cholesterol (log(TG)) (Supp. Fig. 20e), and total cholesterol (TC) (Supp. Fig. 20f). PRS quantiles for RICE-CV (common variants) are plotted on the x-axis, and standardized trait on the y-axis. Data are stratified by rare variant PRS quantiles from RICE-RV (blue: below 5%, grey: 20–70%, pink: above 95%). Results are shown for individuals of African (AFR), Admixed American/Latino (AMR), East Asian (EAS), European (EUR), Middle Eastern (MID), and South Asian (SAS) ancestries. The training data consisted of individuals of African, Admixed American, and European ancestries, while the tuning and validation sets included all six ancestries. Full sample sizes details for each ancestry are provided in **Supplementary Data 5.** Source data are provided as a Source Data file.

a) Relationship between ancestry-adjusted common and rare variant PRSs and standardized body mass index (BMI) levels across six ancestral groups from AoU data.

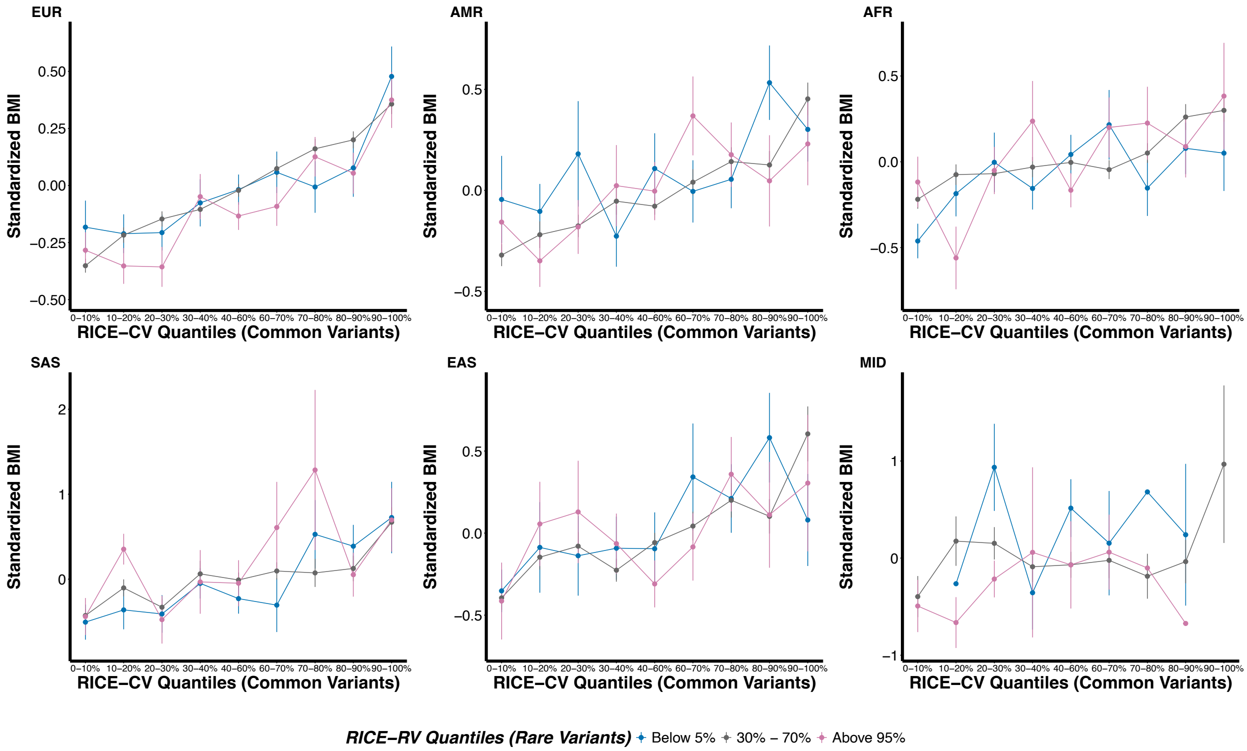

Supplementary Figure 20 continued: b) Relationship between ancestry-adjusted common and rare variant PRSs and standardized high-density lipoprotein cholesterol (HDL) levels across six ancestral groups from AoU data.

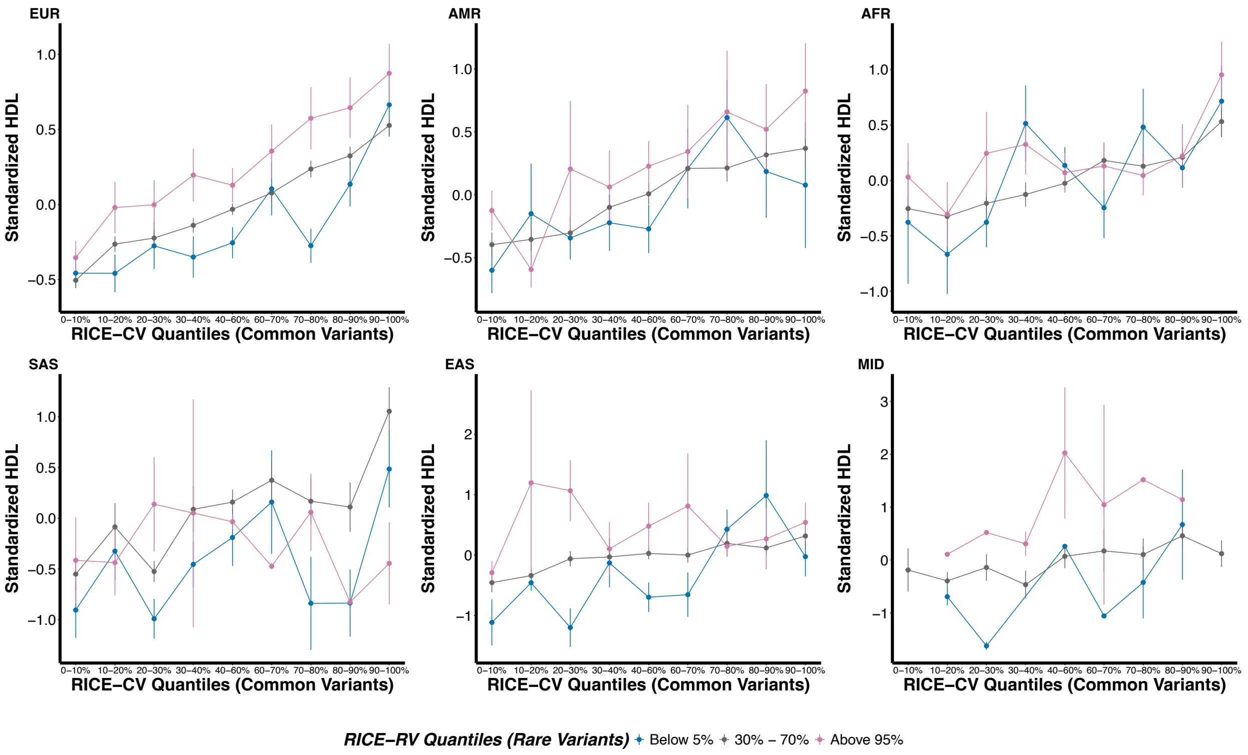

Supplementary Figure 20 continued: c) Relationship between ancestry-adjusted common and rare variant PRSs and standardized height across six ancestral groups from AoU data.

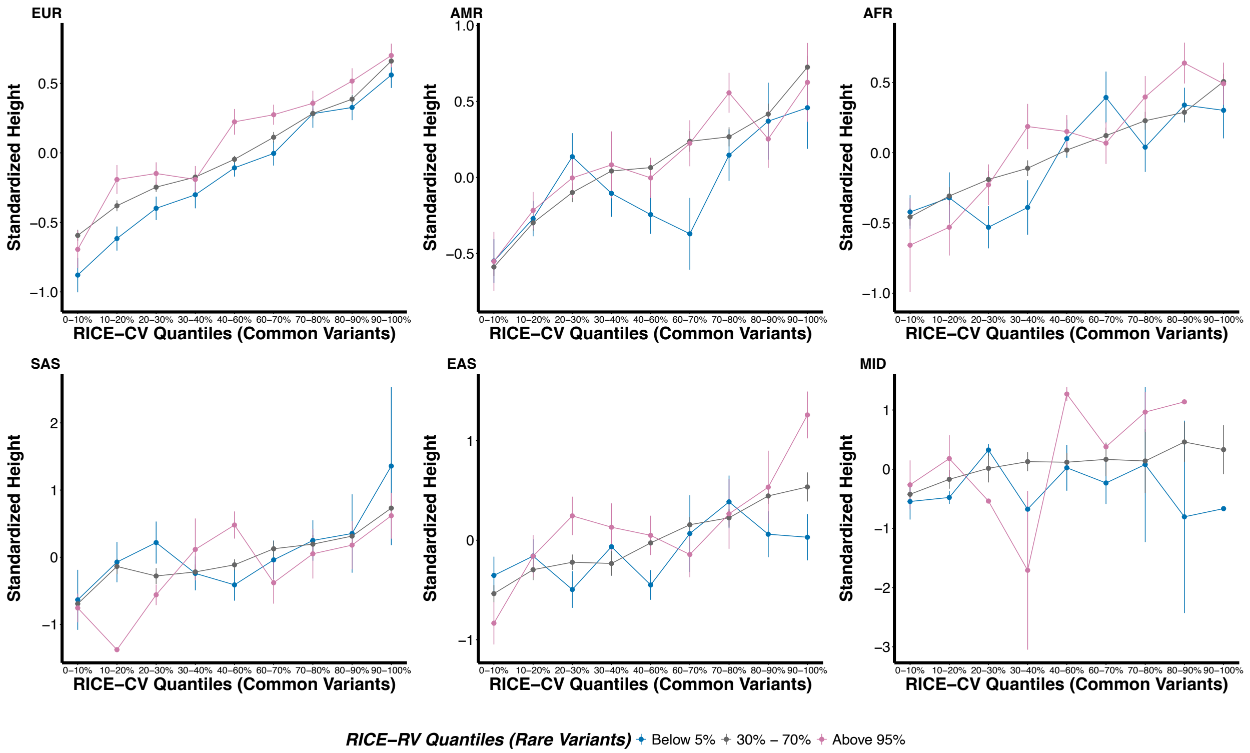

Supplementary Figure 20 continued: d) Relationship between ancestry-adjusted common and rare variant PRSs and standardized low-density lipoprotein cholesterol (LDL) levels across six ancestral groups from AoU data.

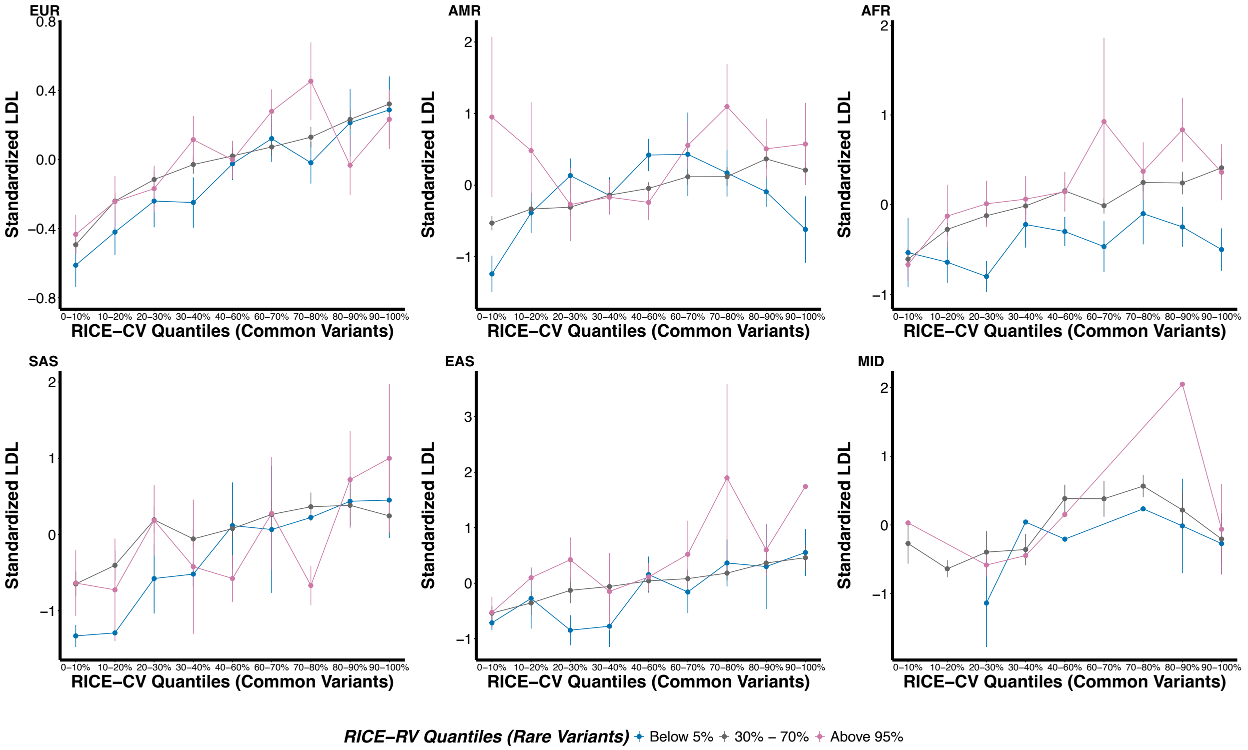

Supplementary Figure 20 continued: e) Relationship between ancestry-adjusted common and rare variant PRSs and standardized natural logarithm of triglycerides (log(TG)) levels across six ancestral groups from AoU data.

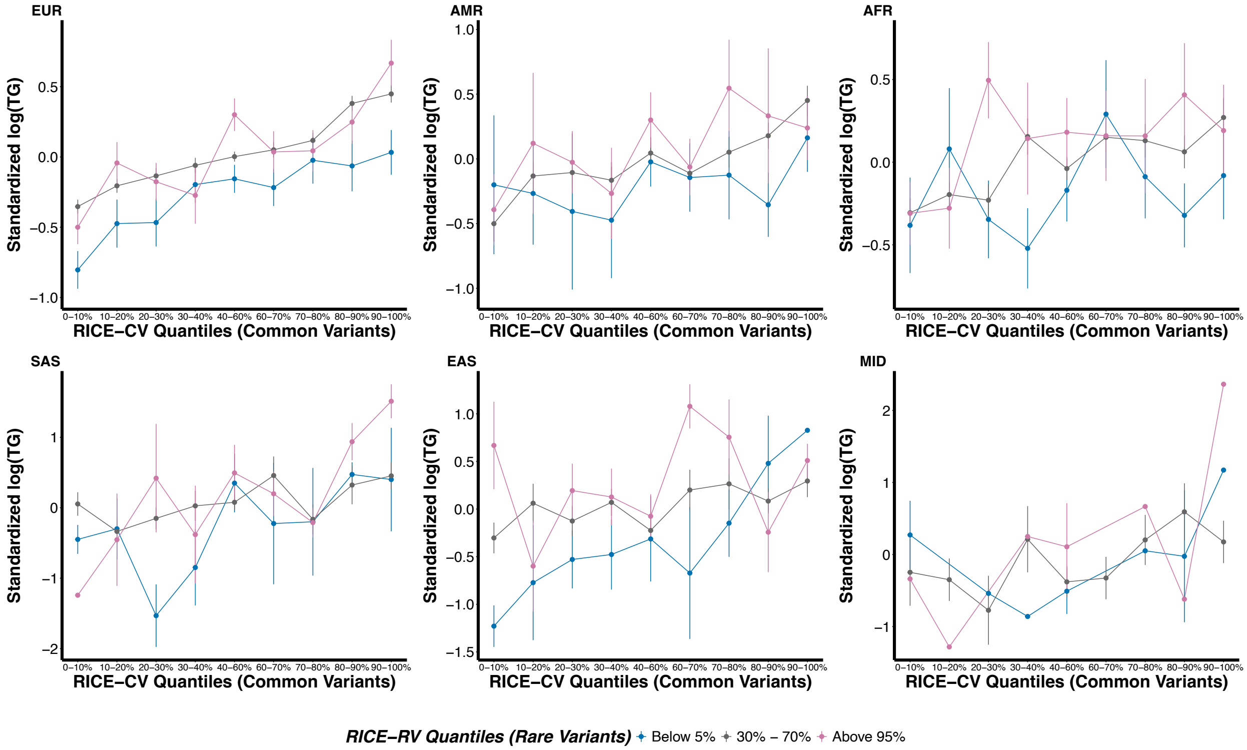

Supplementary Figure 20 continued: f) Relationship between ancestry-adjusted common and rare variant PRSs and standardized total cholesterol (TC) levels across six ancestral groups from AoU data.

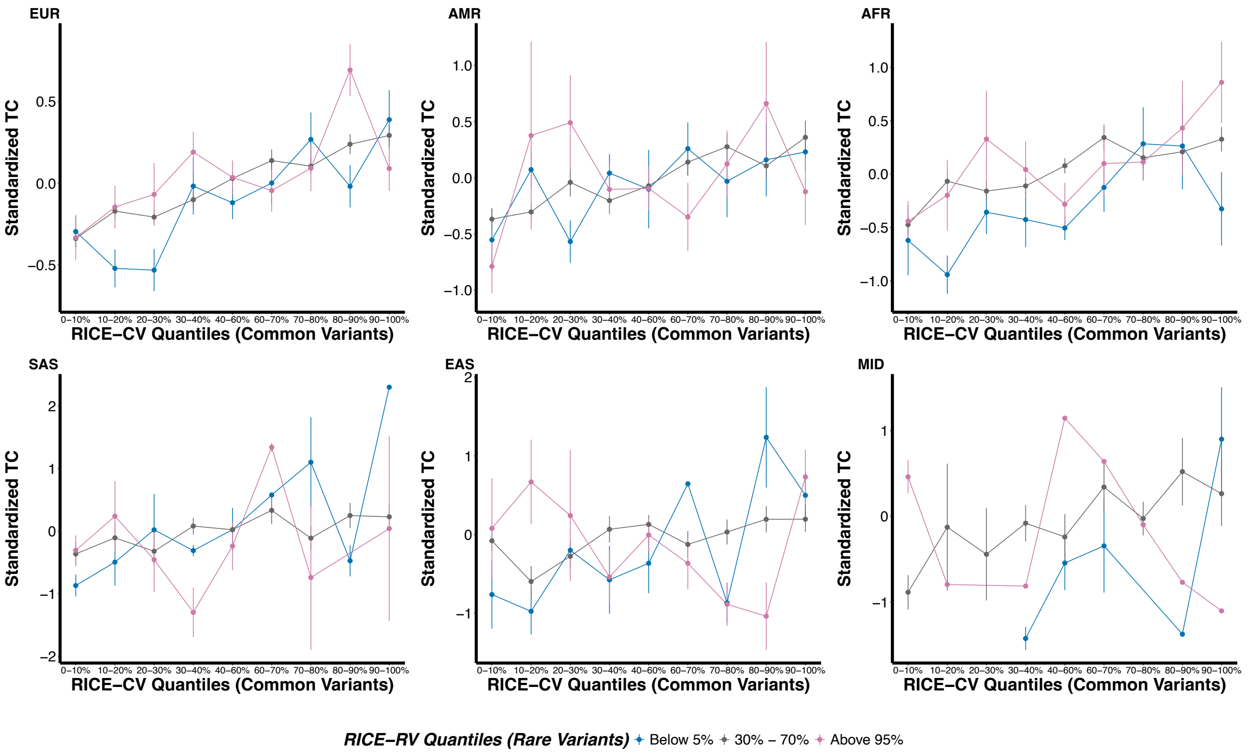

Supplementary Figure 21. Assessing the prediction performance of RICE trained on All of Us (AoU) and validated on UK Biobank data. The six continuous traits analyzed include body mass index (BMI), height, high-density lipoprotein cholesterol (HDL), low-density lipoprotein cholesterol (LDL), the natural logarithm of triglyceride cholesterol (log(TG)), and total cholesterol (TC). Results are shown for individuals of African (AFR), Admixed American/Latino (AMR), European (EUR), and South Asian (SAS) ancestries. The training data from AoU consisted of individuals of African, Admixed American, European, the tuning data from AoU consisted of African, Admixed American, East Asian, European, and South Asian ancestries, and the validation data from UKB Imputed + WES included African, Admixed American/Latino, European, and South Asian. Source data are provided as a Source Data file.

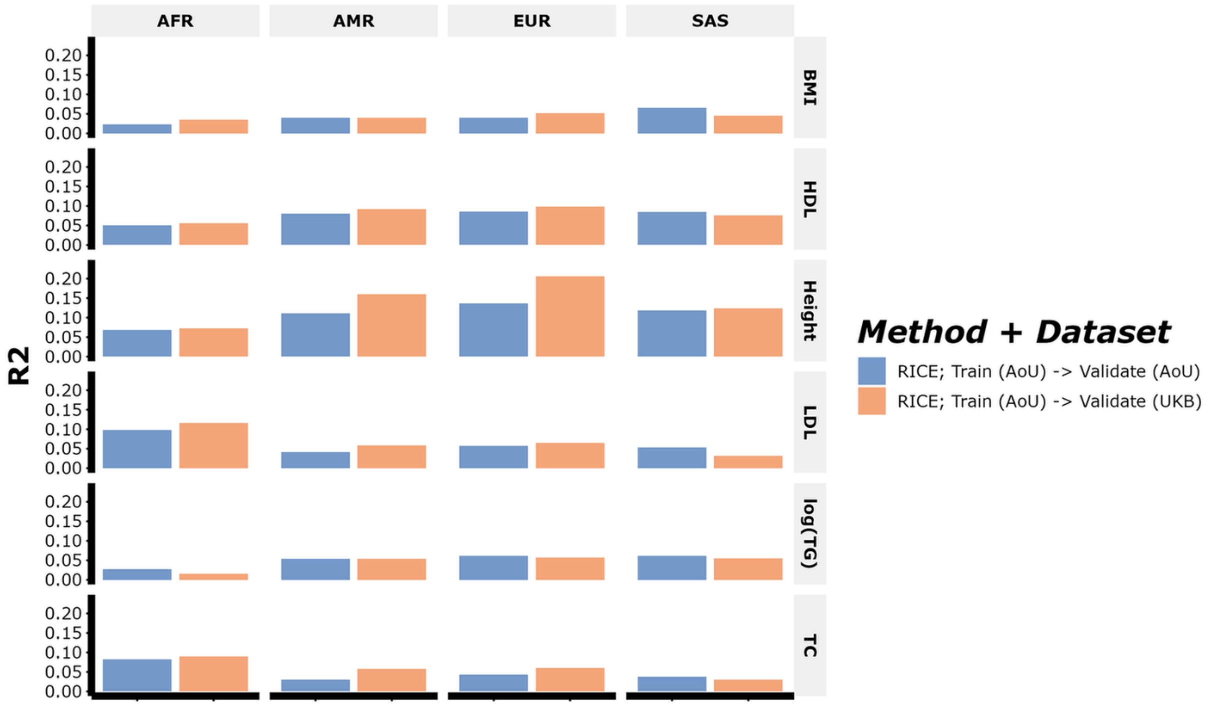

Supplementary Note

Genotype quality control of the UK Biobank whole exome sequencing
UK Biobank (UKB) whole exome sequencing (WES) data were preprocessed following steps out lined in a prior manuscript[1]. VCF files for UK Biobank WES data for 200,643 participants were downloaded[2]. Quality control measures were performed in the following steps[3]. First, variants with Hardy–Weinberg equilibrium $P < 1 \times 10^{-15}$ were removed. Second, any SNV genotype with read depth less than seven reads (DP < 7) and indel genotype with DP < 15 was changed to a no-call. Third, any heterozygous genotype was changed to a no-call if any of the conditions are satisfied as follows: (1) genotype quality < 20, (2) allele balance < 0.15 for SNV and allele balance < 0.20 for indel, and (3) binomial test on allelic balance using allelic depth $P < 1 \times 10^{-3}$. Lastly, variants with more than 10% missing genotypes were excluded.

Rare variant association testing was implemented with the processed genotypes. Common variant association testing was performed with the processed genotype data with an additional minor allele frequency filter (MAF > 1%).

Genotype quality control of the UK Biobank whole genome sequencing
The analysis of rare variants used pVCF format files for whole genome sequencing (WGS) data of 200,004 UK Biobank participants (UK Biobank Field #24304) were used following the same quality control procedure in a previous study of UK Biobank WGS data[4]. All variants were kept that had pass indicated by QC label and AAScore greater than 0.5, where AAScore was generated by GraphTyper, the software used by the UK Biobank to perform genotype calling. vcf2agds was used to preprocess the UK Biobank WGS data[5].

Common variant association testing was used with the Plink formatted genotype data derived from the pVCF format files for WGS data (UK Biobank Field #24305). Quality control measures were applied to the genotype data using four criteria; (1) variants with minor allele frequency greater than 1% were kept, (2) variants with Hardy–Weinberg equilibrium $P < 1 \times 10^{-6}$ were removed, (3) variants with missing call rates exceeding 0.02 were removed, and (4) samples with missing call rate exceeding 0.05 were removed.

Genotype quality control of the All of Us
The analysis of rare variants used version 7.1 Plink formatted short read whole genome sequencing data that includes SNPs and indel variants that are within the exon regions provided by All of Us (srWGS: Exome)[6].

The analysis of common variants used version 7.1 Plink formatted short read whole genome sequencing data including SNPs and indel variants that are frequent in the All of Us computed subpopulations provided by All of Us (srWGS: ACAF Threshold)[6]. Genotype data was further constrained to only include variants that had minor allele frequency greater than 0.01 in the Admixed American, African, and European computed subpopulations.

Proof of Beta of PRS per SD
Assuming a model

$$\boldsymbol{Y} = \boldsymbol{G\beta} + \boldsymbol{\epsilon},$$

where Y is a vector of observed phenotype values for individuals of a single ancestry and G is a standardized genotype matrix. Under this equation, the true heritability explained by G is defined as

$$h^2 = \frac{\mathrm{var}(\boldsymbol{G\beta})}{\mathrm{var}(\boldsymbol{Y})}.$$

Defining an estimate PRS as $\mathbf{PRS} = \boldsymbol{G\hat{\beta}}$ where $\boldsymbol{\hat{\beta}} = (\boldsymbol{G^T G})^{-\mathbf{1}}\boldsymbol{G^T Y}$. Then the standardized PRS can be defined as

$$\mathrm{PRS_{Stand}} = \frac{\boldsymbol{G\hat{\beta}}}{(\mathrm{var}(\boldsymbol{G\hat{\beta}}))^{1/2}},$$

and the standardized response as

$$\boldsymbol{Y}_{\mathrm{Stand}} = \frac{\boldsymbol{Y}}{(\mathrm{var}(\boldsymbol{Y}))^{1/2}}.$$

The estimated coefficient of the standardized PRS from the linear model $\boldsymbol{Y}_{\mathbf{Stand}} = \boldsymbol{PRS}_{\mathbf{Stand}}\,\boldsymbol{b} + \boldsymbol{\epsilon}$ is given as

$$\begin{aligned}
\boldsymbol{\hat{b}} &= (\boldsymbol{PRS}_{\mathbf{Stand}}{}^{\boldsymbol{T}}\boldsymbol{PRS}_{\mathbf{Stand}})^{-\mathbf{1}}\boldsymbol{PRS}_{\mathbf{Stand}}{}^{\boldsymbol{T}}\boldsymbol{Y}_{\mathbf{Stand}} \\
&= \left(\frac{\boldsymbol{\hat{\beta}^T G^T G\hat{\beta}}}{\mathrm{var}(\boldsymbol{G\hat{\beta}})}\right)^{-1} \frac{\boldsymbol{\hat{\beta}^T G^T}}{(\mathrm{var}(\boldsymbol{G\hat{\beta}}))^{\frac{1}{2}}} \frac{\boldsymbol{Y}}{(\mathrm{var}(\boldsymbol{Y}))^{\frac{1}{2}}} \\
&= \left(\frac{\mathrm{var}(\boldsymbol{G\hat{\beta}})}{\mathrm{var}(\boldsymbol{Y})}\right)^{\frac{1}{2}} \left(\boldsymbol{Y^T G}(\boldsymbol{G^T G})^{-\mathbf{1}}\boldsymbol{G^T G}(\boldsymbol{G^T G})^{-\mathbf{1}}\boldsymbol{G^T Y}\right)^{-\mathbf{1}}\boldsymbol{Y^T G}(\boldsymbol{G^T G})^{-\mathbf{1}}\boldsymbol{G^T Y} \\
&= \left(\frac{\mathrm{var}(\boldsymbol{G\hat{\beta}})}{\mathrm{var}(\boldsymbol{Y})}\right)^{\frac{1}{2}} \\
&= (h^2)^{\frac{1}{2}}
\end{aligned}$$

Ancestry Adjusted PRS

We standardize the PRS distributions for each PRS constructed, RICE-CV, RICE-RV, RICE, or conventional common variant methods, using a regression-based method to adjust for differences in distributions across ancestries[7]. The standardization involves two steps: mean adjustment and variance adjustment.

1. Mean adjustment

Linear regression is conducted of the raw PRS against the to five principal components (PC):

$$\mathrm{PRS_i} = \alpha_0 + \alpha_1 \mathrm{PC}_{i1} + \alpha_2 \mathrm{PC}_{i2} + \cdots + \alpha_5 \mathrm{PC}_{i5} + \epsilon_i{}^{\mathrm{mean}}.$$

The residuals $\mathrm{r_i}$ of the raw PRS accounting for mean differences in the PRS distributions across ancestry are computed as:

$$r_i = \mathrm{PRS}_i - \hat{\alpha}_0 - \hat{\alpha}_1 \mathrm{PC}_{i1} - \hat{\alpha}_2 \mathrm{PC}_{i2} - \cdots - \hat{\alpha}_5 \mathrm{PC}_{i5}.$$

2. Variance adjustment

Using the square residuals $r_i{}^2$ as a proxy for the variance of the PRS distribution, a second linear regression is ran:

$$r_i{}^2 = \gamma_0 + \gamma_1 \mathrm{PC}_{i1} + \gamma_2 \mathrm{PC}_{i2} + \cdots + \gamma_5 \mathrm{PC}_{i5} + \epsilon_i{}^{\mathrm{var}}.$$

Then the final ancestry-adjusted PRS for each individual i was computed as:

$$\mathrm{PRS}_i{}^{\mathrm{adj}} = \frac{r_i}{\sqrt{\hat{\gamma}_0 + \hat{\gamma}_1 \mathrm{PC}_{i1} + \hat{\gamma}_2 \mathrm{PC}_{i2} + \cdots + \hat{\gamma}_5 \mathrm{PC}_{i5}}}.$$

The resulting distribution of the standardized PRS has mean 0 and variance 1 within each ancestry, ensuring that PRS is accurately modeled regardless of ancestry.

Supplementary References

1. Li, X. *et al.* Powerful, scalable and resource-efficient meta-analysis of rare variant associations in large whole genome sequencing studies. *Nat. Genet.* **55**, 154–164 (2022).
2. Szustakowski, J. D. *et al.* Advancing human genetics research and drug discovery through exome sequencing of the UK Biobank. *Nat. Genet.* **53**, 942–948 (2021).
3. Van Hout, C. V. *et al.* Exome sequencing and characterization of 49,960 individuals in the UK Biobank. *Nature* **586**, 749–756 (2020).
4. Halldorsson, B. V. *et al.* The sequences of 150,119 genomes in the UK Biobank. *Nature* **607**, 732–740 (2022).
5. Li, X. *et al.* Streamlining large-scale genomic data management: Insights from the UK Biobank whole-genome sequencing data. *Cell Genomics* **5**, 101009 (2025).
6. Bick, A. G. *et al.* Genomic data in the All of Us Research Program. *Nature* **627**, 340–346 (2024).
7. Chen, T. *et al.* Genomic insights for personalised care in lung cancer and smoking cessation: motivating at-risk individuals toward evidence-based health practices. *EBioMedicine* **110**, 105441 (2024).
