# Supplementary material for: Integrating common and rare variants improves polygenic risk prediction across diverse populations
Source: Nat Commun. 2026 Apr 24;17:5772. doi: 10.1038/s41467-026-72185-2 (PMC13323733; doi:10.1038/s41467-026-72185-2)
Supplement: Supplementary file 2 — Description of Additional Supplementary Files [file 41467_2026_72185_MOESM2_ESM.pdf]

File Name: Supplementary Data 1

Description: Sample size for training, tuning, and validation for the simulation study using chromosome 22 of UK Biobank whole exome sequencing data

File Name: Supplementary Data 2

Description: Sample size for training, tuning, and validation in the UK Biobank imputed + whole exome sequencing analysis

File Name: Supplementary Data 3

Description: Sample size for training, tuning, and validation in the UK Biobank whole genome sequencing analysis

File Name: Supplementary Data 4

Description: Genomic control factor and LDSC intercept for eleven traits

File Name: Supplementary Data 5

Description: Sample size for training, tuning, and validation in the All of Us analysis

File Name: Supplementary Data 6

Description: Estimated coefficient and AUC/R2 of the Standardized PRS and their 95% confidence intervals for the UK Biobank imputed + whole exome sequencing analysis

File Name: Supplementary Data 7

Description: Estimated coefficient and AUC/R2 of the Standardized PRS and their 95% confidence intervals for the UK Biobank whole genome sequencing analysis

File Name: Supplementary Data 8

Description: Estimated coefficient and AUC/R2 of the Standardized PRS and their 95% confidence intervals for the All of Us analysis

File Name: Supplementary Data 9

Description: Total number of common variants and rare variants in each analysis

File Name: Supplementary Data 10

Description: Timing of each step of RICE for the UKB Imputed + WES analysis in compute hours
