# Supplementary material for: Integrating common and rare variants improves polygenic risk prediction across diverse populations
Source: Nat Commun. 2026 Apr 24;17:5772. doi: 10.1038/s41467-026-72185-2 (PMC13323733; doi:10.1038/s41467-026-72185-2)
Supplement: Supplementary file 4 — Reporting Summary [file 41467_2026_72185_MOESM4_ESM.pdf]

nature portfolio

Corresponding author(s): Jacob Williams, Xihao Li, and Haoyu Zhang

Last updated by author(s): Mar 2, 2026

Reporting Summary

Nature Portfolio wishes to improve the reproducibility of the work that we publish. This form provides structure for consistency and transparency in reporting. For further information on Nature Portfolio policies, see our Editorial Policies and the Editorial Policy Checklist.

Statistics

For all statistical analyses, confirm that the following items are present in the figure legend, table legend, main text, or Methods section.

n/a	Confirmed	
☐	☒	The exact sample size (n) for each experimental group/condition, given as a discrete number and unit of measurement
☐	☒	A statement on whether measurements were taken from distinct samples or whether the same sample was measured repeatedly
☐	☒	The statistical test(s) used AND whether they are one- or two-sided<br>*Only common tests should be described solely by name; describe more complex techniques in the Methods section.*
☐	☒	A description of all covariates tested
☐	☒	A description of any assumptions or corrections, such as tests of normality and adjustment for multiple comparisons
☐	☒	A full description of the statistical parameters including central tendency (e.g. means) or other basic estimates (e.g. regression coefficient) AND variation (e.g. standard deviation) or associated estimates of uncertainty (e.g. confidence intervals)
☐	☒	For null hypothesis testing, the test statistic (e.g. F, t, r) with confidence intervals, effect sizes, degrees of freedom and P value noted<br>*Give P values as exact values whenever suitable.*
☐	☒	For Bayesian analysis, information on the choice of priors and Markov chain Monte Carlo settings
☒	☐	For hierarchical and complex designs, identification of the appropriate level for tests and full reporting of outcomes
☐	☒	Estimates of effect sizes (e.g. Cohen's d, Pearson's r), indicating how they were calculated

Our web collection on statistics for biologists contains articles on many of the points above.

Software and code

Policy information about availability of computer code

Data collection	No software was used for data collection.
Data analysis	Simulation and data analyses code are archived on Zenodo (v1.0.092). The corresponding GitHub repository is https://github.com/jwilliams10/RareVariantPRS.<br><br>Tutorial to implement RICE is available at: GitHub (https://github.com/jwilliams10/RICE). Software implementing CT-SLEB is available at: GitHub (https://github.com/andrewhaoyu/CTSLEB). Software implementing PROSPER is available at: GitHub (https://github.com/Jingning-Zhang/PROSPER). Software implementing JointPRS is available at: GitHub (https://github.com/LeqiXu/JointPRS). Software implementing Lassosum2 and LDpred2: https://github.com/privefl/bigsnpr. Software implementing PLINK: https://www.cog-genomics.org/plink/1.9/. Software implementing PLINK2: https://www.cog-genomics.org/plink/2.0/. vcf2agds is implemented as a collection of applets in the UK Biobank Research Analysis Platform (RAP) available at https://github.com/drarwood/vcf2agds_overview. vcf2agds was used to preprocess the UK Biobank WGS data. Most of our statistical analyses were performed using the following R packages: ggplot2 version 3.5.1, dplyr version 1.1.4, bigsnpr version 1.12.15, SuperLearner version 2.0.29, caret version 6.0.94, glmnet version 4.1.8, STAARpipeline version 0.9.7, TxDb.Hsapiens.UCSC.hg38.knownGene version 3.18.0, SeqVarTools version 1.42.0, and SeqArray version 1.44.0.

For manuscripts utilizing custom algorithms or software that are central to the research but not yet described in published literature, software must be made available to editors and reviewers. We strongly encourage code deposition in a community repository (e.g. GitHub). See the Nature Portfolio guidelines for submitting code & software for further information.

Data

Policy information about availability of data

All manuscripts must include a data availability statement. This statement should provide the following information, where applicable:

- Accession codes, unique identifiers, or web links for publicly available datasets
- A description of any restrictions on data availability
- For clinical datasets or third party data, please ensure that the statement adheres to our policy

UK Biobank phenotype data, WES data, and WGS data can be accessed through the UK Biobank research analysis platform (https://ukbiobank.dnanexus.com/landing). All data used in this research are publicly available to registered researchers through the UKB data-access protocol and who are listed as collaborators on UKB-approved access applications. All of Us phenotype data, WES data, and WGS data can be accessed through the All of Us research workbench (https://workbench.researchallofus.org/login version 7.1). All data used in this research are publicly available to registered researchers with controlled tier access through the All of Us data-access protocol. Data generated in this study are largely available in the Supplementary Data or Source Data files. Large results generated in this study, including common-variant GWAS summary statistics, rare-variant association test summary statistics, and the RICE-CV and RICE-RV model weight files, are available through a Harvard Dataverse dataset.

Research involving human participants, their data, or biological material

Policy information about studies with human participants or human data. See also policy information about sex, gender (identity/presentation), and sexual orientation and race, ethnicity and racism.

Reporting on sex and gender

The real data analyses of UK Biobank and All of Us, data of all sexes were used for all traits except prostate and breast cancer which were limited to sex defined as males and females, respectively. Sex, age, and age^2 were used as control covariates in all real data analyses.

Reporting on race, ethnicity, or other socially relevant groupings

For the analysis of UK Biobank (UKB) whole exome sequencing (WES), simulation data, and whole genome sequencing (WGS) we used genetically-inferred ancestry labels created using the 1000 Genomes Project Phase 3 (1000G) data. The 1000G dataset includes 633 Europeans (EUR), 893 Africans (AFR), 585 East Asians (EAS), 601 South Asians (SAS), and 490 Admixed Americans or Latino (AMR), totaling 3,202 individuals. Principal component analysis was conducted on all 3,202 samples using plink v2.0. A random forest classifier was run to map every individual in 1000G to their respective populations using the first 5 principal components. The UKB WES and WGS datasets were then projected to this PC space and applied the same random forest classifier to produce genetically-inferred ancestry labels.

For the analysis of All of Us WES data, genetically-inferred ancestry labels (EUR, EAS, SAS, AMR, MID, AFR) were provided by All of Us.

Population characteristics

The detailed sample size for each ancestry in UK Biobank is provided in Supplementary Data 1, 2, and 3. We use the individuals with available quality control and sample relateness information in All of Us (AoU) The detailed sample size for each ancestry can be found in Supplementary Data 4.

For the UK Biobank data, the average participant age was 56.26 (SD = 8.11). The average participant age for each genetically-inferred ancestry are as follows: African - 51.67 (SD = 8.00), Admixed American or Latino - 53.54 (SD = 8.31), European - 56.54 (SD = 8.03), and South Asian - 53.30 (SD = 8.60). Female participants made up 55% of the UK Biobank data with ancestry specific percentages: African - 60%, Admixed American or Latino - 54%, European - 55%, and South Asian - 47%. Details about UK Biobank's genotyping can be found at https://www.ukbiobank.ac.uk/enable-your-research/about-our-data/genetic-data.

For the All of Us data, the average participant age was 52.12 (SD = 17.11). The average participant age for each genetically-inferred ancestry are as follows: African-American - 49.55 (SD = 14.88), Admixed American or Latino - 44.89 (SD = 15.81), East Asian- 44.50 (SD = 17.40), European - 56.09 (SD = 17.16), Middle Eastern - 44.72 (SD = 17.46), and South Asian - 42.08 (SD = 16.51). Female participants made up 60% of the All of Us data with ancestry specific percentages: African-American - 57%, Admixed American or Latino - 66%, East Asian- 63%, European - 59%, Middle Eastern - 53%, and South Asian - 53%. Details about All of Us genotyping can be found at https://support.researchallofus.org/hc/en-us/articles/27633757470228-All-of-Us-Genomic-Quality-Report.

Recruitment

All of Us data set is accessed through All of Us Research Program (https://workbench.researchallofus.org/). The UK Biobank data is obtained under the UK Biobank resource application 52008.

Ethics oversight

This study used de-identified data from the UK Biobank under approved application 52008 and from the All of Us Research Program under controlled-tier access via the Researcher Workbench. UK Biobank has research ethics approval from the North West Centre for Research Ethics Committees (REC reference 11/NW/0382). All of Us participants are consented under the All of Us research protocol, approved by the All of Us Institutional Review Board. Additional information on All of Us IRB oversight is available at https://allofus.nih.gov/about/who-we-are/institutional-review-board-irb-of-all-of-us. Analyses were conducted using de-identified data within the respective secure analysis environments.

Note that full information on the approval of the study protocol must also be provided in the manuscript.

Field-specific reporting

[illegible]

For a reference copy of the document with all sections, see nature.com/documents/nr-reporting-summary-flat.pdf

Life sciences study design

All studies must disclose on these points even when the disclosure is negative.

Sample size	Using UK Biobank whole exome and whole genome sequencing data, we analyzed 11 traits for four different ancestries. Detailed sample sizes are provided in Supplementary Data 1, 2, and 3. Using All of Us data, we analyzed six traits for six different ancestries. Detailed sample size information is provided in Supplementary Data 4.<br><br>No a priori sample-size (power) calculations were performed because this study is a secondary analysis of existing population cohorts and the number of participants is constrained by the availability of sequencing and phenotype data. For each analysis (each trait × ancestry group × cohort), we included all eligible participants with available whole-exome/whole-genome sequencing and the relevant phenotype after applying the quality-control and inclusion/exclusion criteria described in Methods, to maximize statistical power and generalizability across ancestries. Final analytic sample sizes therefore varied by trait and ancestry due to differences in phenotype availability and QC filtering and are reported in Supplementary Data 1-3 and 5.
Data exclusions	Real data analyses of UK Biobank and All of Us data were restricted to unrelated individuals as defined by UK Biobank and All of Us respectively. Exclusion of variants due to quality control is described in the Supplementary Material. Detailed information about genotyping, ancestry determination, quality control, removing related individuals are listed in All of Us Research Program Genomic Research Data Quality Report (https://support.researchallofus.org/hc/en-us/articles/27633757470228-All-of-Us-Genomic-Quality-Report).
Replication	The simulation study was performed with 100 replications of each simulation setting, and performance metrics reported in the manuscript are summarized by averaging across these 100 independent validation datasets. In the real data analyses of the UK Biobank and All of Us data, data was randomly split into training (70%), tuning (15%), and validation (15%). The model is trained using the GWAS summary level statistics from training dataset, tuning dataset is used to determine the model parameters, and all final performance estimates and reported results were computed on the independent held-out validation split. The same pre-specified analysis pipeline was applied independently within each cohort, providing an external check of consistency across datasets.
Randomization	Not applicable. This is an observational study.
Blinding	Not applicable. This is an observational study.

Reporting for specific materials, systems and methods

We require information from authors about some types of materials, experimental systems and methods used in many studies. Here, indicate whether each material, system or method listed is relevant to your study. If you are not sure if a list item applies to your research, read the appropriate section before selecting a response.

Materials & experimental systems

n/a	Involved in the study
☒	☐ Antibodies
☒	☐ Eukaryotic cell lines
☒	☐ Palaeontology and archaeology
☒	☐ Animals and other organisms
☒	☐ Clinical data
☒	☐ Dual use research of concern
☒	☐ Plants

Methods

n/a	Involved in the study
☒	☐ ChIP-seq
☒	☐ Flow cytometry
☒	☐ MRI-based neuroimaging

Plants

Seed stocks	*Report on the source of all seed stocks or other plant material used. If applicable, state the seed stock centre and catalogue number. If plant specimens were collected from the field, describe the collection location, date and sampling procedures.*
Novel plant genotypes	*Describe the methods by which all novel plant genotypes were produced. This includes those generated by transgenic approaches, gene editing, chemical/radiation-based mutagenesis and hybridization. For transgenic lines, describe the transformation method, the number of independent lines analyzed and the generation upon which experiments were performed. For gene-edited lines, describe the editor used, the endogenous sequence targeted for editing, the targeting guide RNA sequence (if applicable) and how the editor was applied.*
Authentication	*Describe any authentication procedures for each seed stock used or novel genotype generated. Describe any experiments used to assess the effect of a mutation and, where applicable, how potential secondary effects (e.g. second site T-DNA insertions, mosiacism, off-target gene editing) were examined.*
