# Supplementary material for: Integrating common and rare variants improves polygenic risk prediction across diverse populations
Source: Nat Commun. 2026 Apr 24;17:5772. doi: 10.1038/s41467-026-72185-2 (PMC13323733; doi:10.1038/s41467-026-72185-2)
Supplement: Supplementary file 5 — Transparent Peer Review file [file 41467_2026_72185_MOESM5_ESM.pdf]

nature portfolio

Peer Review File

Integrating Common and Rare Variants Improves Polygenic Risk Prediction Across Diverse Populations

Corresponding Author: Dr Haoyu Zhang

This file contains all reviewer reports in order by version, followed by all author rebuttals in order by version.

Version 0:

Reviewer comments:

Reviewer #1

(Remarks to the Author)

In this manuscript, Williams and colleagues developed RICE, a PRS framework designed to integrate both common and rare variant PRS generated by various existing tools. They evaluated RICE using both simulated and real whole-exome and whole-genome sequencing data from the UK Biobank and the All of Us Research Program, and showed that incorporating rare variant PRS alongside common variant PRS can improve prediction accuracy. While there are existing studies exploring the relationship between common and rare PRS for certain conditions, a comprehensive investigation is lacking. This work is thus timely, both in establishing a pipeline for constructing rare variant PRS, and in assessing its performance across several complex traits and diseases in biobanks. The manuscript is generally well written. However, I have a few major concerns which I outline below.

- There are major issues with the performance metric used throughout the manuscript (i.e., the regression coefficient of the standardized PRS on the standardized trait). First, this metric is not easily interpretable particularly for binary phenotypes. A similar metric -- odds ratio per standard deviation change in PRS -- is widely used and may be more appropriate for binary outcomes. Interpreting a XX% increase in the beta coefficient as the PRS being XX% more accurate doesn't seem appropriate to me. Second, this metric does not account for the low frequency of rare variants and thus will likely lead to an overestimation of the impact of RV PRS in the population. The authors mentioned that metrics like R2 and AUC are average-based and do not fully capture the potential of RV PRS, which I totally agree with; however those metrics are still useful to report as they provide a reference for understanding the relative and joint contributions of CV and RV PRS in explaining population-level variation in the trait.

More importantly, the metric used in the manuscript does not provide any insights into the practical or clinical utility of RV PRS. For example, the effects of CV PRS and RV PRS are assessed and reported separately, but what if the goal is to identify high-risk individuals in the population? If the authors propose to use a score that integrates CV and RV PRS, I would expect to see the effect of the integrated PRS, in comparison with the CV PRS alone. Additionally, what is the utility of RV PRS for patient stratification or identifying high-risk individuals? I would expect to see metrics such as odds ratios comparing individuals in top percentiles of the PRS with population average, both with and without incorporating RV PRS. Other useful metrics include how many additional high-risk individuals can be identified by incorporating RV PRS, or the net reclassification index.

In summary, the metric proposed by the authors is neither interpretable nor clinically informative. While it's still a valid metric to report, additional metrics that are practically and clinically informative are needed to fully characterize the importance of RV PRS in comparison to CV PRS.

- It appears that in the UK Biobank and All of Us analyses, RICE-CV was applied to WES/WGS data rather than imputed genotype data. I think this substantially limited the performance of CV PRS, especially for WES, due to the relatively small number of common variants captured. As a result, this may have biased the comparison of prediction accuracy between CV and RV PRS. Given that imputed data from UK Biobank are readily available, I see no reason not to use them and adhere to the best practices for CV PRS construction. In All of Us, WGS data can be filtered to common variants and used for CV PRS

construction. It would also be interesting to evaluate the performance of RICE on external large-scale GWAS for selected traits and diseases.

- The manuscript focused on a scenario where individual-level training data are available, with the RV PRS being trained conditioned on the CV PRS constructed on the same sample. In practice, however, most users would not run their own GWAS and RV association tests, but calculate CV and RV PRS using existing summary statistics, such as large-scale CV GWAS and RV association results generated by the STAARpipeline. These CV and RV summary statistics may not be derived from the same set of individuals. I was wondering whether the RICE pipeline is still applicable in such cases, and what the potential challenges might arise (e.g., CV and RV PRS may not be independent in this scenario).

- All evaluations in this work were conducted by splitting a single dataset (UK Biobank or All of Us) into training, tuning and validation sets. It would be interesting to assess the performance of RICE across different datasets, e.g., training in UK Biobank and testing in All of Us, and vice versa. In practice, training sets often differ from testing cohorts, so it's important to evaluate the robustness of RICE in this context. This scenario may pose additional challenges, particularly for RV PRS as the observed set of rare variants could differ between datasets due to variations in sequencing technologies and sample characteristics.

- RICE requires a relatively large tuning dataset to fit penalized regressions and perform ensemble learning. Is the method still applicable if an independent tuning sample is difficult to acquire especially for non-European populations?

- As the authors presented and discussed, a major contribution to the RV PRS came from established high-penetrance genes such as LDLR, APOB, and PCSK9 for lipids and coronary artery disease. Existing studies already examined the interactions between these genes and CV PRS (see e.g., https://www.nature.com/articles/s41467-020-17374-3). It would be interesting to examine and quantify the benefit of including additional genome-wide RV signals above and beyond known monogenic risk variants.

- The causal variants proportions in the simulations (0.01, 0.05, and 0.2) focused on relatively sparse genetic architectures. What about a more polygenic model with 1% or more causal variants?

- Would that be possible to add confidence intervals to all reported CV and RV PRS associations, e.g., those in figures 3, 4, 6 and 7? The authors claimed that RV PRS significantly improve prediction but no statistical test was performed to assess the 'significance'.

- Computational cost of each component of RICE should be described and reported.

- I may have missed but the authors discussed two approaches for standardizing PRS in the main text -- (1) a PC-based regression approach and (2) standardizing within each ancestry. The authors stated that performance of the two approaches was consistent and the regression-based approach was presented to avoid categorizing individuals into discrete ancestry groups. However, I can't find a description of the regression-based approach in the methods section, and it seems that in many analyses PRS were still standardized separately within each ancestry. Please clarify. In additional, a mixed-ancestry tuning dataset seemed to be used in this work. Does the performance of the final PRS depend on the ancestry composition of the tuning set?

- Is there any justification for the 1x10^(-3) p-value threshold used for STAAR-Burden?

- Line 269: "This suggests that rare variants can have both protective and deleterious effects, influencing an individual's risk status." I'm not sure this statement is supported by the data presented. Even if all rare variants are deleterious, similar effects can still be observed when stratifying individuals based on RV PRS quantiles, right?

(Remarks on code availability)

Reviewer #2

(Remarks to the Author)
This paper introduces a new PRS framework (RICE) to integrate both common and rare variants, demonstrating the benefits

of including rare variants in complex trait prediction using WES and WGS datasets in UK Biobank and All of Us. With the increasing availability of WGS data, PRS methods that fully leverage WGS will become essential. Their approach of incorporating rare variants through burden scores is a reasonable attempt, and it's encouraging to see it works for some traits. However, some results may be misleading without further clarification.

Major comments:
1. The use of rare variant burden scores for trait prediction is not novel. The authors should provide a brief background in Introduction and cite relevant prior work, such as PMID: 34615865; PMID: 36309498.

2. Quantifying prediction accuracy using the regression slope of standardized phenotypes on standardized PRS is also not new. The lower regression slope due to imperfect (standardized) predictor is known as attenuation bias (e.g., PMID: 37491308). Such a slope is still a "average-based metric" at the cohort level - it can be shown that in this setting the slope is the correlation between y and PRS, which is the usual way of assessing prediction accuracy. It is unclear how using the standardized PRS unveil insights beyond the standard correlation, as claimed in "providing a more nuanced assessment of predictive performance".

3. A key advantage of including rare variants is the potential to identify individuals with low common variant PRS but high disease risk due to rare variants (e.g., PMID: 33110269). The authors should examine whether such individuals exist for the traits analyzed. For example, Figure 2 shows individuals with low RICE-CV PRS but high HDL phenotype. The question is whether their method can effectively capture cases with low CV PRS but high RV PRS.

4. The burden scores approach implicitly assumes that causal rare alleles are proportional to the non-causal rare alleles within the region. This method relies on the assumption that regions with higher rare variant counts (high SNP burden score) also contain proportionally more causal variants (high causal burden score). They should evaluate whether high burden scores introduce systematic bias when causal burden scores do not proportionally align with SNP burden scores. They could do a simulation with varying proportions of causal rare variants across regions/SNP sets.

5. Their claim of adequate quality control is not convincingly supported by the data. Line 273 "Quality control assessments for UKB WES association analyses were performed using Manhattan and QQ plots, confirming well-calibrated p-values and controlled inflation factors (Supplementary Figures 7–9)" However, Supplementary Figure 8 shows substantial inflations in the QQ plots, as observed P-values deviate significantly from expected values at the beginning.

6. Some results are unclear and difficult to interpret:
a) In UKB WES analysis, what explains the large variation in RICE-RV between ancestries, such as AFR and SAS for BMI (Figure 4)? The discrepancies are much larger than those in the simulation.
b) Why is prediction seemingly driven entirely by RICE-RV for some trait, such as Breast cancer in AFR and Prostate cancer in AMR (Supplementary Figure 3)?
c) why does RICE-RV contribute substantially to BMI in UKB WES data (Figure 4) but not in UKB WGS data (Supplementary Figure 10), given that UKB WGS include WES?
d) RICE-RV have nearly zero effects on breast cancer in both UKB WES and WGS data of European ancestry (Supplementary Figure 3 and 10), despite the known contributions of large-effect alleles (e.g., BRACA1/2). How do the authors reconcile this?
e) For some traits, both RICE-CV and RICE-RV have nearly zero prediction accuracy is nearly zero in both, such as Breast cancer in SAS and CAD in AFR (Supplementary Figure 10). Since their RICE-CV is based on ensemble learning that combines predictors from existing methods, how/why does it perform much worse than existing methods?

7. Line 269 "This suggests that rare variants can have both protective and deleterious effects, influencing an individual's risk status." Line 286 "Incorporating RICE-RV helped to identify individuals whose trait values were significantly impacted by rare variants, which would have been missed by common variant PRSs alone." However, the figures (Fig S6 and Fig 5) do not seem to directly support these conclusions. Again, a more direct approach could be to identify cases with low RICE-CV but high RICE-RV.

8. They introduce multi-ancestry methods for RICE-CV PRS, but surprisingly, they do not include these methods in comparison. It's unclear whether RICE-CV outperforms the existing multi-ancestry methods.

Minor:
Line 105: "independent individuals". More common to say "unrelated individuals".
Line 954: "For the k-th rare variant" should be "k-th rare variant set".
Line 989-992: The notation PRS_j is unclear. It appears to correspond to a specific lambda value in line 969 and 977, but this should be clarified, along with the range of lambda tested.
The Methods section contains many subsections, which should be grouped to improve readability.

(Remarks on code availability)

Reviewer #3

(Remarks to the Author)
Williams et al. present an approach for integrating rare variants with common variants for polygenic risk score estimation in large-scale genetic datasets. They introduce a pipeline tool, RICE, which incorporates the results of multiple existing tools to learn ensemble scores leveraging common and rare variants in two steps. RICE is utilized in the WGS and WES in the UK Biobank, as well as the All-of-Us dataset, and the authors demonstrate an overall performance improvement across almost all traits based on a novel metric proposed in the manuscript. We find the manuscript interesting but have major concerns regarding choices in modeling design and statistical approach.

Major comments

1. First, a major issue is that the performance is reported on validation data and not on held-out test data. Here test data would usually be a completely unseen data set to evaluate how well the prediction models generalize and is standard for evaluation of machine learning models. Without evaluation on a held-out test set, it is not possible to compare the performance of RICE to other models.

2. Related to this, we find that the choice of terminology regarding the data subsets is rather confusing. In the manuscript, the terms training, tuning, and validation are used. The standard is to use training, validation, and test. As mentioned above, the test data would be a completely unseen data set to evaluate how well the prediction models generalize.

3. The 'validation' set used in the manuscript cannot be understood as a held-out test set as the authors are using the 'validation' set to optimize the combined RICE-CV and RICE-RV model. Therefore, it is strictly part of the training (and tuning) dataset.

4. When the ensemble model on common variants (RICE-CV) is trained (termed tuned) this represents extra training of the model. The performance of RICE-CV should therefore be compared to each of the PRS models that are trained on both training and tuning datasets. For instance, for the simulated UKB dataset, the training data was 98k individuals, and the tuning data was 20k individuals. This means that RICE-CV is trained on 20k more individuals (20% increase in training data) compared to the single models that are only trained on the 98k. RICE-CV should be compared to single models trained on 98+20k individuals.

5. The authors introduce a new evaluation metric for the predictive performance of their tools due to issues in traditional metrics, which may overlook contributions from rare variants according to the authors. The new metric (regression coefficient of the standardized PRS on the standardized trait) is shown to correspond to the square root of the heritability in a final linear model for one term. However, the authors never show that this relationship holds, when including both the PRS_cv and the PRS_rv terms? We would expect the two terms to be somewhat correlated, making the additive assumption in their predictive performance metric potentially impossible. This could be one of the reasons why the performance gains using the authors' metric are so large. The authors should show the predictive performances of the models using the traditional metrics as well. The authors also never show or argue that their performance metric would hold in the binary case, where even more terms exist in the final model with a logistic link. The binary trait is kept on the original scale, which means the assumption of a standardized phenotype used in their proof of the metric does not hold.

6. In the initial association testing for both common and rare variants, the authors used the original phenotype values, which we assume are on the original scale as well. For continuous traits, a more common approach would be to utilize rank-based inverse normal transformed phenotype values or similar. One could theorize that the rare variants are gaining predictive power by being better at capturing the extreme outliers in the original phenotype scale, which would also persist after the residual standardization in the polygenic risk score estimation. It would be great if the authors could address this and dispute that the predictive performance gain from rare variants arises from phenotype artifacts.

7. It is also common practice to utilize a linear mixed model (LMM)-based approach (e.g. BOLT-LMM) or similar (REGENIE) for performing association testing to ensure proper correction for structure. The authors utilize linear models with only 10 PCs to correct for population structure, while the WGS paper of UK Biobank uses 20-40 PCs while also using a LMM-based approach. In Supplementary Figure 7, 8, 15, 16 and 20, it is very clear in the plots that the GWAS summary statistics of the common variants are highly inflated for all traits (as well as many of the WES rare variant association testing), which is highly likely due to improper correction for structure. Despite this observation, the authors state that their Manhattan and QQ plots confirm well-calibrated p-values and controlled inflation factors, which is contradictory. Effect sizes are inflated due to excessive residual confounding, which could further inflate the predictive performance of polygenic risk scores as they may be modelling non-genetic effects as well, potentially benefiting rare variants due to cryptic structure in the dataset.

8. It is unclear how the principal components (PCs) are obtained in the different datasets and their subsets, as the authors adjust for the top 10 PCs in most steps of their analysis pipeline. We therefore assume that the authors are using the pre-computed PCs from the UK Biobank and All-of-Us. However, this means that the validation set (test set) has been used in the estimation of the PCs, such that it is not an unseen set, thus the test set evaluation will be biased. A way to avoid this would be to perform PCA in the training data to obtain their PCs and only afterward project the test individuals onto the PC space produced by the training data.

9. The genetically-inferred ancestry labels in the UK Biobank were estimated based on a random forest classifier trained on the top 5 PCs estimated from the 1000 Genomes Project (1KGP). However, here you use the 3202 individuals in 1KGP, which also includes all related individuals in 1KGP and not the 2504 "unrelated" individuals from the phase 3 release. This

would create a bias in the estimated PCs as it will somewhat capture relatedness and not population structure. The labels here are the super populations in 1KGP, but there are plenty of individuals with mixed ancestry, which will be completely disregarded using your classification approach as well. How was the ancestry labelling of individuals defined? Using the argmax or some probability threshold?

Minor comments

10. It is not clear how the other methods are evaluated, if it is the same approach as for PRS_cv, where the single standardized PRS is used in a linear model with the standardized phenotype based on their new performance metric.

11. Due to using multiple external computation tools and fitting multiple prediction models (ensemble) as well, it would be nice to have an estimate or overview of the full computational runtime for a given trait (both in the continuous and the binary case).

12. How different is your approach that performs both LASSO and Ridge regression in the ensemble step (using the “SuperLearner” package) from an elastic net that optimizes both the L1 and L2 norm jointly? Ridge logistic regression is not used in your pipeline for binary traits due to the unavailability in the “SuperLearner” package, however, that should be available (alongside elastic net) in other popular machine learning packages. It is already a part of the “glmnet” package that is a requirement for your pipeline as an example.

13. The predictive performance for binary traits is usually reported on the liability scale using some pseudo R^2-measures to account for the case-control ratio between data and population prevalence. Pseudo R^2’s are by no means a perfect metric, but how are the case-control ratio and population prevalence mitigated with your proposed metric?

(Remarks on code availability)

Reviewer #4

(Remarks to the Author)
I co-reviewed this manuscript with one of the reviewers who provided the listed reports. This is part of the Nature Communications initiative to facilitate training in peer review and to provide appropriate recognition for Early Career Researchers who co-review manuscripts.

(Remarks on code availability)

Version 1:

Reviewer comments:

Reviewer #1

(Remarks to the Author)
This revision has enhanced the manuscript, and I commend the authors for their hard work. I have a few remaining comments and suggestions as detailed below.

- I remain skeptical about using "Beta per SD of PRS" as a metric for rare variant PRS. As the authors point out, it is effectively the square root of variance explained and therefore does not really carry additional information beyond R2. In the rare variant PRS context, the metric is also not easily interpretable because the SD of PRS depends on the frequency of the variant. A more interpretable metric might be to report the effect per additional copy of the risk variant. My concern is that this choice of metric could overstate the contribution of rare variant PRS, which would look much smaller if beta is squared and expressed as variance explained. That said, I do not think this issue should prevent publication.

- From Figure 1 and the accompanying text (line 150), it appears that another linear combination between common and rare PRS is learned during evaluation, which could imply overfitting. However, the Methods section seems to suggest that the combination weights were trained using the tuning set. This should be clarified. The authors might consider adjusting Figure 1 to avoid giving the impression that additional model fitting was performed on evaluation data.

- The STAAR framework uses kernel-based methods (e.g., SKAT) to detect variant set associations, while RICE aggregates rare variants into burden scores, which may fail to capture nonlinear effects that contribute to association signals. It would be helpful to discuss this limitation explicitly.

- The simulation settings do not appear realistic. For example, assuming that common and rare variants on chromosome 22 explain 5% and 1.25% heritability, respectively, is overly optimistic. All simulation scenarios focus on unrealistically strong genetic signals and do not examine performance in settings where GWAS/EWAS power is limited. In reality, for many traits and diseases, genome-wide rare variants collectively explain <1% of heritability.

- In Figure 3, the “Beta per SD” for rare variant PRS reaches ~0.2, implying ~4% variance explained, whereas the simulated variance explained by rare variants is only 1.25%. Please clarify this discrepancy. I'm not sure if I missed anything here.

- Statements such as RICE-RV increased R^2 by XXX% compared to RICE-CV are not particularly meaningful because the relative contributions of common and rare variants were predetermined and set by authors and were completely artificial.

- I would suggest the authors take out the WES-only configuration for RICE-CV as it's not what is used in practice. This would simplify the manuscript.

- The manuscript focuses primarily on lipid traits, for which rare variant PRS is known to have large contributions to the traits. However, for most other complex traits and diseases examined, rare variant PRS seems to contribute little or no predictive value. This limitation should be discussed more clearly, and the overall conclusions tuned down accordingly. It remains unclear whether rare variant PRS improves prediction beyond common variant PRS for complex traits or diseases in general, which depends on the genetic architecture of the traits and diseases.

- The newly added Figure 5 is helpful, but I would still like to see an analysis aligned with how PRS is used in practice, e.g., quantifying how many additional cases are identified or the improvement in OR when stratifying individuals by the integrated score compared with common variant PRS alone.

- Please clarify whether the cross-dataset analysis was limited to overlapping variants between UKB and AoU, or if it was performed on all available variants in AoU and then applied to those present in UKB. The latter would be a more realistic scenario, but either way this should be clarified.

I was also asked to assess whether the authors have sufficiently addressed the concerns raised by Reviewer 2, who is no longer available.

Reviewer 2 had raised a similar concern about the metric “Beta per SD of PRS.” Since the slope does not provide additional information beyond R2, I find the statements that this metric “emphasizes individual-level effects of rare variants” and offers “an individualized perspective” unconvincing.

Reviewer 2 also raised a similar concern about whether cases with low CV PRS but high RV PRS can be effectively captured. As I mentioned, Figure 5 is informative in showing the distribution of risk profiles among individuals with extreme phenotypes, but it does not demonstrate how such individuals can be identified in practice by thresholding CV and RV PRS.

The responses to the other comments appear sufficient to me.

(Remarks on code availability)

Reviewer #3

(Remarks to the Author)
I want to thank the authors for their careful revision of the manuscript. No further comments.

(Remarks on code availability)

Version 2:

Reviewer comments:

Reviewer #1

(Remarks to the Author)
The authors have addressed my concerns. I would like to thank them for their hard work and patience.

(Remarks on code availability)

Open Access This Peer Review File is licensed under a Creative Commons Attribution 4.0 International License, which permits use, sharing, adaptation, distribution and reproduction in any medium or format, as long as you give appropriate credit to the original author(s) and the source, provide a link to the Creative Commons license, and indicate if changes were made.
In cases where reviewers are anonymous, credit should be given to 'Anonymous Referee' and the source.
The images or other third party material in this Peer Review File are included in the article's Creative Commons license, unless indicated otherwise in a credit line to the material. If material is not included in the article's Creative Commons license and your intended use is not permitted by statutory regulation or exceeds the permitted use, you will need to obtain permission directly from the copyright holder.
To view a copy of this license, visit https://creativecommons.org/licenses/by/4.0/

Author Response Letter

We are grateful for the reviewers' very helpful and thoughtful comments. We have carefully incorporated these suggestions into the revised manuscript, which we believe has significantly strengthened the work. Our detailed, point-by-point responses to each of the reviewer's comments are provided below:

Reviewer #1 (Remarks to the Author):

In this manuscript, Williams and colleagues developed RICE, a PRS framework designed to integrate both common and rare variant PRS generated by various existing tools. They evaluated RICE using both simulated and real whole-exome and whole-genome sequencing data from the UK Biobank and the All of Us Research Program, and showed that incorporating rare variant PRS alongside common variant PRS can improve prediction accuracy. While there are existing studies exploring the relationship between common and rare PRS for certain conditions, a comprehensive investigation is lacking. This work is thus timely, both in establishing a pipeline for constructing rare variant PRS, and in assessing its performance across several complex traits and diseases in biobanks. The manuscript is generally well written. However, I have a few major concerns which I outline below.

We thank you for the positive assessment of our manuscript, recognizing the timeliness of RICE as a comprehensive framework for integrating common and rare variant PRSs across traits and biobanks. We appreciate the constructive feedback and have addressed the major concerns below, incorporating new analyses and revisions to strengthen the work.

Q1: There are major issues with the performance metric used throughout the manuscript (i.e., the regression coefficient of the standardized PRS on the standardized trait). First, this metric is not easily interpretable particularly for binary phenotypes. A similar metric -- odds ratio per standard deviation change in PRS -- is widely used and may be more appropriate for binary outcomes. Interpreting a XX% increase in the beta coefficient as the PRS being XX% more accurate doesn't seem appropriate to me. Second, this metric does not account for the low frequency of rare variants and thus will likely lead to an overestimation of the impact of RV PRS in the population. The authors mentioned that metrics like R2 and AUC are average-based and do not fully capture the potential of RV PRS, which I totally agree with; however those metrics are still useful to report as they provide a reference for understanding the relative and joint contributions of CV and RV PRS in explaining population-level variation in the trait.

More importantly, the metric used in the manuscript does not provide any insights into the practical or clinical utility of RV PRS. For example, the effects of CV PRS and RV PRS are assessed and reported separately, but what if the goal is to identify high-risk individuals in the population? If the authors propose to use a score that integrates CV and RV PRS, I would expect to see the effect of the integrated PRS, in comparison with the CV PRS alone. Additionally, what is the utility of RV PRS for patient stratification or identifying high-risk individuals? I would expect to see metrics such as odds ratios comparing individuals in top percentiles of the PRS with population average, both with and without incorporating RV PRS. Other useful metrics include how many additional high-risk individuals can be identified by incorporating RV PRS, or the net reclassification index.

In summary, the metric proposed by the authors is neither interpretable nor clinically informative. While it's still a valid metric to report, additional metrics that are practically and clinically informative are needed to fully characterize the importance of RV PRS in comparison to CV PRS.

We sincerely thank the reviewer for this thoughtful and constructive feedback. We agree that incorporating more interpretable and clinically relevant metrics is important for a comprehensive evaluation of PRSs, particularly those including rare variants. Below, we address each aspect of the comment and note the corresponding manuscript revisions.

1. Clarifying the regression coefficient of the standardized PRS

We apologize for any confusion in our presentation. The regression coefficient of the standardized PRS has a clear interpretation:

- For continuous traits, it represents the change in standardized phenotype per SD increase in PRS (equivalent to the correlation and approximately the square root of the variance explained).
- For binary traits, it corresponds to the log odds ratio (log OR) per SD increase in PRS, a standard measure in clinical genetics and genetic epidemiology.

We have revised all relevant figures and legends to label this explicitly as “Beta per SD of PRS” (continuous traits) and “log OR per SD of PRS” (binary traits). We also revised the text to describe “effect size per SD” rather than “accuracy,” to improve interpretability. We have updated the **Results** section as follows:

Results (Line 156, Evaluation of Prediction Performance):

“To better capture the contribution of rare variants, we report an additional, complementary metric: the estimated regression coefficient of the standardized PRS on the standardized trait (**Methods**). For continuous traits, this coefficient represents the expected change in standardized phenotype per standard deviation (SD) increase in PRS. For binary traits, it corresponds to the log odds ratio (log OR) per SD increase in PRS, a widely used and interpretable measure in clinical genetics and genetic epidemiology. Throughout this manuscript, we refer to this as the standardized effect size of PRS, denoted as “Beta per SD of PRS” for continuous traits and “log OR per SD of PRS” for binary traits. As detailed in the **Supplementary Note**, this coefficient can also be expressed as the square root of the heritability explained by the PRS. We use this metric to quantify the effect sizes of both common and rare variant PRSs, particularly when rare variants may have limited influence on average-based metrics, offering an interpretable per-SD effect that complements standard PRS measures like R^2 or AUC.”

2. Including traditional performance metrics (R^2 and AUC)

We fully agree that R^2 (continuous traits) and AUC (binary traits) are valuable population-level metrics for benchmarking. We have now included these metrics for all real and simulated analyses, derived from a joint model combining RICE-CV and RICE-RV PRSs (trained on the tuning set, evaluated on the validation set). Additional results are provided in the manuscript (detailed below). To assess statistical significance, we used 10,000 bootstrap resamples for confidence intervals on RICE-RV's effect size (testing $\neq 0$) and pairwise R^2/AUC differences (RICE vs. best common variant method). Significance is denoted as ** (95%) or *** (99%) in figures.

We have updated the **Results** and **Methods** Section as follows:

Results (Line 179, Evaluation of Prediction Performance):

“RICE's predictive performance was evaluated using three complementary metrics: (1) standardized effect size, for assessing statistical significance and relative improvements; (2) R^2 (continuous traits) or AUC (binary traits), for absolute predictive accuracy; and (3) trait differences across PRS quantiles, to illustrate potential clinical relevance in the UKB and AoU datasets.”

Results (Line 219, Simulation Study Results):

“RICE-RV also performed strongly, effectively identifying causal rare variant signals and predicting their effects across both EUR and non-EUR populations (**Figure 3** and **Supplementary Figure 1**). When added to RICE-CV, RICE-RV significantly improved overall predictive performance. Across ancestries, it increased R^2 by an average of 35.9% compared to using RICE-CV alone.”

Results (Line 274, UKB WES Results):

“UKB WES Results. In the WES-only configuration, RICE (combining common and rare variants) achieved higher standardized effect sizes and R^2 compared to alternatives for continuous traits (**Supplementary Figure 9d**, **Supplementary Table 7**). On average, RICE-CV improved R^2 by 6.7% over the best alternative method for each trait-ancestry combination. The largest gains were observed for lipid traits. In EUR individuals, RICE increased R^2 by 10.1% for HDL, 3.2% for height 2.8% for LDL, 11.4% for log(TG) and 3.2% for TC over the best alternative method (**Supplementary Figure 9d**). RICE also showed marked improvements in non-EUR populations, with R^2 gains of 49.9% for TC in AFR, 41.7% for HDL in AMR, 34.7% for log(TG) in AMR, and 29.7% for LDL in AFR (**Supplementary Figure 9d**). Standardized effect size analyses showed that RICE-RV contributed significantly to most lipid traits ($p < 0.05$) across ancestries, with the exceptions of HDL and log(TG) in AFR, and TC in AMR (**Supplementary Figure 9c**).”

Results (Line 307, UKB Imputed + WES Results):

“Relative to the best alternative method, RICE (CV+RV) improved R^2 for lipid traits in EUR by 4.9% – 8.0% (**Supplementary Figure 11c**). Notable non-EUR gains included 29.8% for TC in AFR, 25.9% for log(TG) in AMR, and 25.5% for HDL in AMR (**Supplementary Figure 11c**). For height, the rare variant component was statistically significant but small (e.g., $\beta =$ 0.039 in EUR and 0.038 in AMR), and corresponding R^2 gains were marginal, leaving RICE’s performance not significantly different from the best alternative for this trait, consistent with a highly polygenic rare variant architecture that yields small effects at the population level.”

Results (Line 354, UKB WGS Results):

“For rare variants, RICE-RV showed significant effects ($p < 0.05$) for height and all lipid traits across ancestries, with BMI only being significant for SAS (**Supplementary Figure 16c**). Relative to the best alternative method, RICE (CV+RV) increased R^2 in EUR by 7.5% (height),

8.6% (HDL), 11.2% (LDL), 9.6% (log(TG)), and 10.6% (TC) (**Supplementary Figure 16d**). Notable non-EUR gains included 60.7% for log(TG) in AFR and 29.8% for HDL in AMR."

Results (Line 421, All of Us Results):

"For explained variance, the full RICE model (CV+RV) improved R^2 by an average of 1.7% over the best existing method across trait-ancestry pairs (**Supplementary Figure 23**). Notable gains included 14.2% for height in EUR, 43.2% for HDL in EAS, and 18.5% for TC in AFR. In contrast to UKB, we did not observe significant R^2 gains for lipid traits in EUR within AoU, likely reflecting smaller training sample sizes (average N = 65,549 per lipid trait in AoU vs. N = 91,356 in UKB)."

3. Addressing clinical/practical utility

We agree that the ability to identify high-risk individuals is essential for clinical relevance. While the net reclassification index (NRI) is one option, the binary traits in the available biobank datasets have limited case numbers and yielded much fewer genome-wide rare variant associations, making NRI estimates unstable and uninformative in this context.

Instead, we demonstrate clinical relevance through quantile-based stratification plots for continuous traits in UKB and AoU (e.g., **Figures 5; Supplementary Figures 10, 12, 17, 24**), which show clear phenotypic gradients across PRS quantiles. These illustrate that incorporating RICE-RV meaningfully shifts predicted trait distributions, particularly for lipid traits, highlighting its potential to identify individuals at phenotypic extremes. We have added a further context in the **Discussion** noting that in future work, when large-scale sequencing data for binary traits are available, we plan to incorporate NRI to further evaluate high-risk reclassification.

Discussion (Line 564):

"Fourth, smaller case counts for binary outcomes limit the utility of current biobank data for rare variant PRS. Future applications of RICE in large disease-specific consortia may better capture rare variant contributions to binary traits, and could incorporate complementary metrics such as net reclassification index to assess improvements in high-risk individual identification and clinical decision-making."

Meanwhile, following reviewer 2's comment 3, we have added the analyses that showing identifying individuals with low polygenic risk but elevated disease likelihood due to rare variants. We examined all six continuous traits, focusing on whether group 3 (low CV + high RV; (1) Low CV / Low RV, (2) High CV / Low RV, and (4) High CV / High RV) individuals captured additional extreme phenotypes beyond those identified by CV PRS alone. For HDL (provided

below, added as **Figure 5**), among individuals in the top 10% of observed HDL values, 6.3% fell into group 3, meaning they were missed by CV PRS alone but captured by RV PRS. Moreover, mean standardized HDL levels were significantly higher in groups 2, 3, and 4 compared to group 1, with group 3 showing a clear elevation despite low CV PRS ($p = 3.41 \times 10^{-14}$). Similar but more modest patterns were observed for LDL, TC, and log(TG), whereas results for BMI were not significant (**Supplementary Figure 13**), consistent with our earlier effect-size findings where RICE-RV showed no gain for BMI (**Figure 4**).

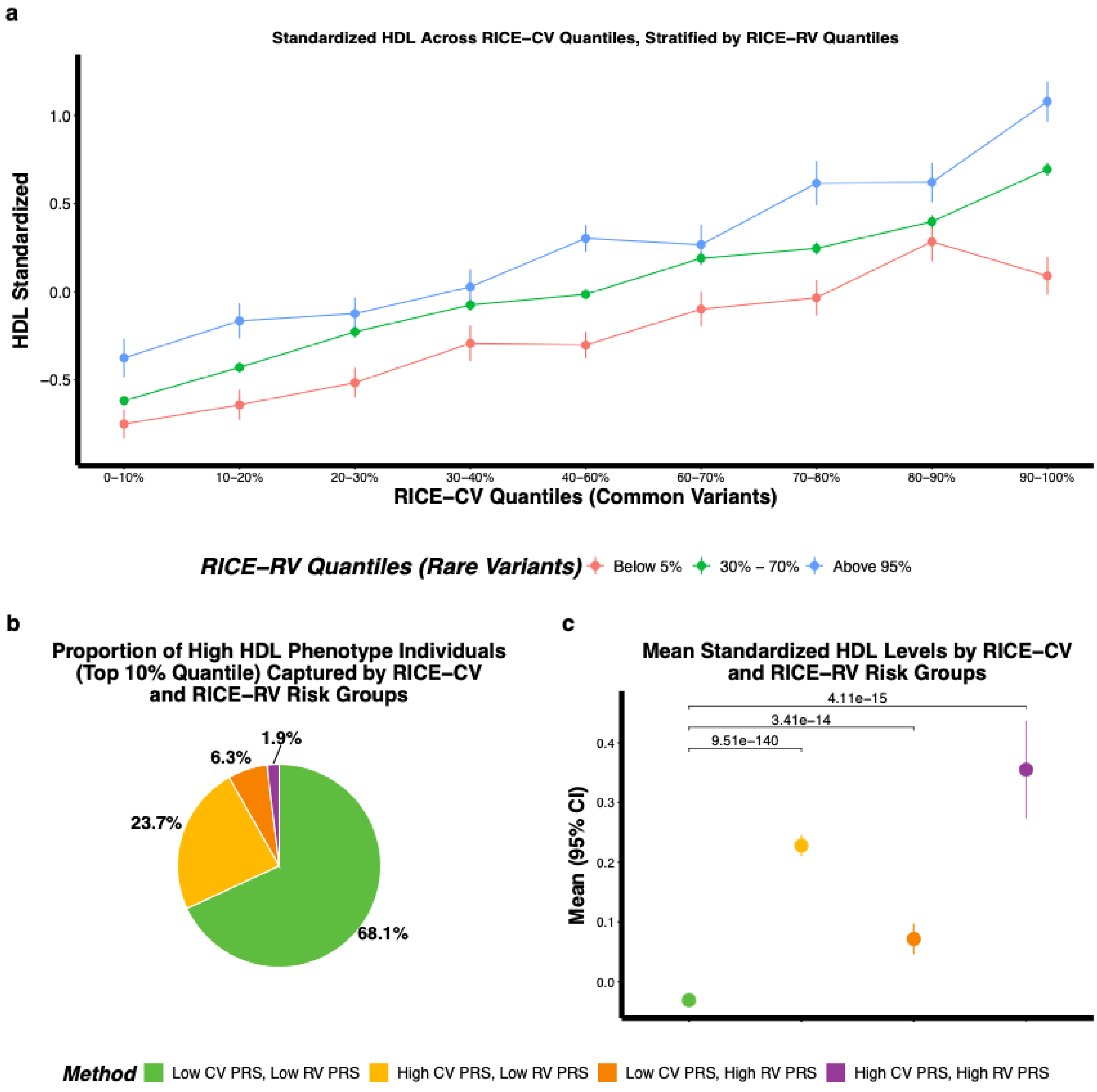

Figure 5. Joint stratification of common and rare variant PRS identifies discrepant high-risk individuals. Individuals in the UKB imputed + WES validation dataset were stratified based on rare variant PRS (RV; high risk = top 5%). (a) mean standardized HDL levels $\pm 1 \times$ standard error (SE) are plotted stratified by PRS quantiles for RICE-CV (common variants) on the x-axis and RICE-RV (rare variants) by color (red: below 5%, green: 30–70%, blue: above 95%). (b) Further stratification by common variant PRS (CV; high risk = top 10%) created four strata: (1) Low CV / Low RV, (2) High CV / Low RV, (3) Low CV / High RV, and (4) High CV / High RV. Proportion of individuals in each group among the top 10% of observed HDL values. (c) Under same grouping as (b), mean standardized HDL levels with 95% confidence intervals and pairwise p-values across the four groups.

We have added the following to the manuscript:

Results (Line 319, UKB Imputed + WES Results):

"To test whether rare variants flag individuals missed by common variant PRS, we cross-classified participants by RICE-CV (top 10% vs. bottom 90%) and RICE-RV (top 5% vs. bottom 95%), yielding four strata (**Methods**). For HDL, stratifying by RICE-RV quantiles shows significant differences across RICE-CV quantiles (**Figure 5a**). Further, 6.3% of individuals in the top phenotype decile were captured only by high RICE-RV despite low RICE-CV (**Figure 5b**). Group-level comparisons confirmed that the low-CV/high-RV stratum had significantly higher HDL than the low-CV/low-RV group ($p = 3.41 \times 10^{-14}$; **Figure 5c**). Similar patterns were seen for other lipid traits but not for BMI, consistent with the standardized effect-size results (**Supplementary Figure 13**). These findings show that the rare variant component of RICE can identify individuals with extreme lipid profiles who would be missed by common variant PRS alone."

Comment 2: It appears that in the UK Biobank and All of Us analyses, RICE-CV was applied to WES/WGS data rather than imputed genotype data. I think this substantially limited the performance of CV PRS, especially for WES, due to the relatively small number of common variants captured. As a result, this may have biased the comparison of prediction accuracy between CV and RV PRS. Given that imputed data from UK Biobank are readily available, I see no reason not to use them and adhere to the best practices for CV PRS construction. In All of Us, WGS data can be filtered to common variants and used for CV PRS construction. It would also be interesting to evaluate the performance of RICE on external large-scale GWAS for selected traits and diseases.

Thank you for the comment. We agree that using imputed data for common variants adheres to best practices and could mitigate any potential bias in CV-RV PRS comparisons due to limited number of common variants captured in WES. Accordingly, we have added a full analysis combining UKB imputed genotypes for RICE-CV with WES for RICE-RV, as described in the subsection **UKB Imputed + WES Results** (**Line 299**). This expands the common variant set (~1.48 million vs. 137K in WES-only), improving CV baseline performance by an average of 20%–34% in standardized effect size across ancestries (**Figure 6**), while preserving overall trends and rare variant gains. We have updated the **Results** section as follows:

Results (Line 299, UKB Imputed + WES Results):

"Using imputed genotypes for common variants (while retaining WES-based rare variants) expanded the variant set and improved baseline PRS performance; overall trends mirrored the WES-only results (standardized effect sizes in **Figure 4** and **Supplementary Figure 11a**; R^2/AUC in **Supplementary Figures 11b–c**; quantile differences in **Supplementary Figure 12**). Among common variant methods, RICE-CV generally achieved the largest standardized effect sizes across continuous traits (**Figure 4**). For rare variants, RICE-RV showed significant standardized effect sizes ($p < 0.05$) for all lipid traits across ancestries and for height in EUR and AMR (**Figure 4**). Relative to the best alternative method, RICE (CV+RV) improved R^2 for lipid traits in EUR by 4.9% – 8.0% (**Supplementary Figure 11c**). Notable non-EUR gains included 29.8% for TC in AFR, 25.9% for log(TG) in AMR, and 25.5% for HDL in AMR (**Supplementary Figure 11c**). For height, the rare variant component was statistically significant but small (e.g., $\beta = 0.039$ in EUR and 0.038 in AMR), and corresponding R^2 gains were marginal, leaving RICE's performance not significantly different from the best alternative for this trait, consistent with a highly polygenic rare variant architecture that yields small effects at the population level. Quantile stratification across ancestries showed clear gradients for lipid traits in EUR, with significant differences between extreme quantiles (**Supplementary Figure 12b and 12d–f**)."

Regarding the All of Us analyses, we apologize for any lack of clarity. As suggested, common variants were already filtered from the WGS dataset (MAF > 0.01 or allele count > 100 in any ancestry, further restricted to MAF > 0.01 in AFR, AMR, or EUR for training), while rare variant analyses were constrained to the exome region for efficiency, aligning with best practices for CV PRS construction from WGS. This is now explicitly stated in both the **Results** and **Methods** sections.

Results (Line 398, All of Us Results):

"Common variants were defined as those with MAF > 0.01 in any of the three ancestries (AFR, AMR, or EUR) in the training dataset. Rare variant analyses were constrained to the exome region for computational efficiency (**Methods**)."

Methods (Line 1319, AoU Analysis):

"We analyzed the AoU dataset using WGS data version 7.1. For common variants, we used the Allele Count/Allele Frequency (ACAF) Threshold callset provided in the AoU Researcher Workbench, which include variants with MAF > 0.01 or allele count > 100 in any of the computed ancestry groups. Given that the training dataset included only AFR, AMR, and EUR ancestries, we further restricted common variants to those with MAF > 0.01 in any of these three ancestries. For rare variants, we focused on exonic regions using the "Exome" callset from the AoU Researcher Workbench."

While we agree that applying RICE to external large-scale GWAS would be interesting, particularly for diseases such as cancer, current public access to individual-level WES/WGS data remains limited. As a methodological manuscript, our UKB and AoU analyses demonstrate the framework's potential, and we look forward to extending RICE to such datasets through future consortium collaborations.

Comment 3: The manuscript focused on a scenario where individual-level training data are available, with the RV PRS being trained conditioned on the CV PRS constructed on the same sample. In practice, however, most users would not run their own GWAS and RV association tests, but calculate CV and RV PRS using existing summary statistics, such as large-scale CV GWAS and RV association results generated by the STAARpipeline. These CV and RV summary statistics may not be derived from the same set of individuals. I was wondering whether the RICE pipeline is still applicable in such cases, and what the potential challenges might arise (e.g., CV and RV PRS may not be independent in this scenario).

Thank you for this insightful comment highlighting a practical use case for RICE. We agree that many users will rely on existing summary statistics rather than generating their own from individual-level data, and addressing this scenario strengthens the manuscript's applicability.

RICE is primarily designed for biobank-scale sequencing data with individual-level access, enabling direct conditioning of rare variant association testing on the common variant PRS (via RICE-CV in the null model). However, the framework can be adapted for scenarios where

common variant GWAS summaries are used to construct RICE-CV (as with existing PRS methods like LDpred2 or Lassosum2), while rare variant analysis retains individual-level data. In this hybrid setup, STAARpipeline can still be run on individual genotypes to generate rare variant p-values and burden annotations, adjusting for the summary-based RICE-CV in the null model to maintain independence. This allows users to leverage existing PGS Catalog entries for common variant PRS or develop powerful common variant PRS without full individual-level data limitations, while preserving the conditioning step for rare variants.

If both common variant PRS and rare variant PRS are derived entirely from summary statistics without mutual adjustment in the training step (e.g., using pre-computed STAARpipeline results unconditioned on CV PRS), potential challenges include the lack of guaranteed independence between CV and RV components, which could overestimate PRS effects due to shared signals. To mitigate this, users could perform approximate conditioning by regressing RV burdens on CV PRS in their tuning dataset (if individual genotypes are available) before the ensemble learning step; however, we acknowledge that full independence may not be achievable without individual-level adjustment, representing a limitation in purely summary-based workflows. We have added a note to the **Discussion** to elaborate on this adaptation and its considerations:

Discussion (Line 569):

"Fifth, while RICE leverages individual-level data for optimal conditioning, it can adapt to external summary statistics (e.g., large-scale GWAS for CV and STAARpipeline results for RV); challenges may arise if summaries are from disparate samples, potentially compromising independence between components, and future extensions could incorporate orthogonalization techniques to address this in summary statistic-based workflows."

Comment 4: All evaluations in this work were conducted by splitting a single dataset (UK Biobank or All of Us) into training, tuning and validation sets. It would be interesting to assess the performance of RICE across different datasets, e.g., training in UK Biobank and testing in All of Us, and vice versa. In practice, training sets often differ from testing cohorts, so it's important to evaluate the robustness of RICE in this context. This scenario may pose additional challenges, particularly for RV PRS as the observed set of rare variants could differ between datasets due to variations in sequencing technologies and sample characteristics.

Thank you for this valuable suggestion. We fully agree that evaluating RICE across distinct datasets is essential to demonstrate its robustness in real-world scenarios, where training and testing cohorts often differ in sample composition, sequencing technologies, and variant

ascertainment, particularly challenging for rare variants due to their population-specific nature and sensitivity to platform differences.

To address this, we conducted a cross-dataset portability analysis by applying PRSs trained on AoU data (using AFR, AMR, and EUR individuals for training, as described in the **AoU Analysis** section of **Methods**) to the UKB validation dataset (imputed genotypes for common variants + WES for rare variants). We focused on the six continuous traits shared across datasets, evaluating performance in AFR, AMR, EUR, and SAS ancestries (sample sizes in **Supplementary Tables 3** and **6**). This setup quantifies the generalizability from AoU (diverse WGS-based training) to UKB (imputed + WES validation), mimicking practical differences in cohorts and technologies.

The results, now detailed in a new subsection, **Evaluation of AoU PRS on UKB** (**Results**, **Line 436**), show strong portability (standardized effect size in **Figure 8**; R^2 in **Supplementary Figure 25**). Average standardized effect sizes for RICE-CV were comparable (0.241 in AoU vs. 0.247 in UKB across traits/ancestries), while RICE-RV maintained similar values (0.059 in AoU vs. 0.053 in UKB). Significance persisted for key traits ($p < 0.05$), including lipids and height in a majority of ancestries, despite a reduction in variant overlap (e.g., ~5.47 million common variants in AoU vs. ~1.48 million in UKB imputed; **Supplementary Table 11**). This confirms RICE's robustness despite the anticipated issues with rare variant differences across datasets, though platform-specific capture led to minor attenuation in RV effects as expected. R^2 was often higher in UKB than in AoU (average 45% relative increase for the full RICE model), possibly due to superior phenotype quality or measurement consistency in that cohort.

We did not perform the reverse (UKB-trained applied to AoU) due to UKB's predominant EUR training set, which would limit multi-ancestry representation in AoU testing; however, the current direction validates cross-biobank transferability. We have updated the manuscript as follows:

Results (Line 436, Evaluation of AoU PRS on UKB):

"To assess the cross-dataset portability of RICE PRSs, we applied models trained on AoU data (using AFR, AMR, and EUR individuals for training; Methods) to the UKB validation dataset and compared performance against validation within AoU (standardized effect sizes in **Figure 8**; R^2 in **Supplementary Figure 25**). Analyses focused on six continuous traits across AFR, AMR, EUR, and SAS ancestries (full sample sizes in **Supplementary Tables 3 and 6**). RICE PRSs demonstrated strong portability, with effect sizes from AoU-trained models remaining robust when evaluated in UKB (**Figure 8**). Average standardized effect sizes for RICE-CV were 0.241

in AoU and 0.247 in UKB across traits and ancestries, while those for RICE-RV were 0.059 in AoU and 0.053 in UKB. RICE-RV retained significant associations ($p < 0.05$) in UKB for many of the same traits as in AoU, including HDL, height, LDL, and log(TG) in EUR; HDL, height, log(TG), and TC in AFR; HDL, height, LDL, and log(TG) in AMR; and log(TG) in SAS (**Figure 8**). No significance was observed for BMI in either dataset. Notably, R^2 values were often higher in UKB than in AoU, with an average relative increase of 45.0% for the full RICE model (**Supplementary Figure 25**). This enhanced performance in UKB may reflect superior phenotype quality or measurement consistency in that cohort, highlighting RICE's generalizability across diverse datasets."

Discussion (Line 530):

"Our cross-dataset evaluation further demonstrated RICE's robustness, with AoU-trained PRSs performing comparably or better when applied to UKB, despite differences in sequencing technologies (WGS in AoU vs. imputed + WES in UKB) and reduced variant overlap. Effect sizes remained stable, and R^2 often increased in UKB (**Figure 8**; **Supplementary Figure 25**), potentially due to phenotype quality or measurement consistency in UKB, while rare variant signals showed consistent results with only minor attenuation, highlighting RICE's consistent predictive performance across real-world datasets. This portability supports RICE's utility in federated or multi-biobank settings, where training and application cohorts differ."

Methods (Line 1361, Cross-Dataset Portability Analysis):

"To evaluate PRS portability, we applied AoU-trained models (using AFR, AMR, and EUR ancestries for training, as described above) to the UKB validation dataset. Common variants were scored using UKB imputed genotypes, and rare variants using UKB WES data, with the same quality control and covariate adjustments (top 10 PCs, sex, age, age squared). Only overlapping variants between AoU and UKB were retained (details in **Supplementary Table 11**). Performance was assessed on the six shared continuous traits (BMI, height, HDL, LDL, log(TG), TC) across AFR, AMR, EUR, and SAS ancestries, using the standardized effect size, R^2, and bootstrap significance (10,000 resamples) as in other evaluations."

We appreciate this feedback, as it enhances the manuscript's demonstration of RICE's practical utility.

Comment 5: RICE requires a relatively large tuning dataset to fit penalized regressions and perform ensemble learning. Is the method still applicable if an independent tuning sample is difficult to acquire especially for non-European populations?

Thank you for raising this important point regarding the tuning dataset requirements, particularly for underrepresented non-European populations where acquiring independent samples can be challenging. We agree that minimizing dependency on large tuning sets enhances RICE's practicality, and we have refined the ensemble learning step accordingly to improve stability with smaller samples.

As part of revisions based on reviewer feedback (Reviewer 3, comment 12), we updated the ensemble algorithm to jointly model optimal predictions from LASSO and ridge regression using a generalized linear model on the tuning PRSs (**Methods**, **Line 1030**), reimplementing it with glmnet to support binary outcomes (previously not supported in SuperLearner R package). For common variant PRSs (from methods like CT, LDpred2, etc.), we use cross-validation to select hyperparameters (λ_1 for Lasso, and λ_2 for ridge), ensuring efficiency. For rare variants, we now directly select the optimal regularization parameter based on R^2 or AUC on the full tuning dataset, addressing sparsity issues and reducing overfitting risks compared to cross-validation (**Methods**, **Line 1129**). This streamlined approach requires fewer resources than the original multi-learner stacking while maintaining performance, as validated in our simulations with halved sample sizes (N=49,173 training; **Figure 3**), where results remained robust.

We acknowledge that RICE still requires a tuning dataset for final PRS calibration, similar to methods like CT or LDpred2. For stable LASSO/ridge convergence, we recommend N > 500–1,000 per ancestry in the tuning set; smaller sizes (especially in non-European populations) may lead to reduced performance or instability. Ongoing efforts in common variant PRS development, such as PUMAS (Zhao, Z. et al., Genome Biology, 2021), PUMAS-ensemble (Zhao, Z. et al., Genome Biology, 2024, Zhao Z. et al., Biorxiv, 2024) and PRStuning (Jiang, W. et al. Nature Communications, 2024), which fine-tune models using only GWAS summary statistics without individual-level tuning data, offer promising directions to alleviate this. Future extensions of RICE could incorporate similar summary statistic-based tuning to enhance applicability in data-limited settings.

To address the comments, we have updated the manuscript as follows:

Discussion (Line 574):

"Meanwhile, summary statistics-based fine-tuning approaches such as PUMAS[80], PUMAS-ensemble[81,82], and PRStuning[83] could be incorporated to reduce the requirement of independent tuning data."

Methods (Line 1030):

"Similarly for binary traits, the ensemble model combines predictions from LASSO and ridge regression using a generalized linear regression model. For LASSO logistic regression, we minimize the objective function:

$$\min_{\boldsymbol{w}^{(1)}} -\frac{1}{n}\sum_{i=1}^{n}[Y_i \log(p_i) + (1 - Y_i)\log(1 - p_i)]^2 + \lambda_1 \sum_{j=1}^{J} |w_j^{(1)}|,$$

where $p_i = \sigma(\sum_{j=1}^{J} w_j^{(1)} PRS_{ij})$ is the predicted probability for individual i, with $\sigma(x) = exp(x)/\{1 + \exp(x)\}$ being the logistic function and Y_i is the binary phenotype (0 or 1) for individual i.

For ridge logistic regression, we minimize the objective function:

$$\min_{\boldsymbol{w}^{(2)}} -\frac{1}{n}\sum_{i=1}^{n}[Y_i \log(p_i) + (1 - Y_i)\log(1 - p_i)]^2 + \lambda_2 \sum_{j=1}^{J} (w_j^{(2)})^2,$$

where $p_i = \sigma\left(\sum_{j=1}^{J} w_j^{(2)} PRS_{ij}\right)$. Optimal regularization parameters λ_1 and λ_2 are selected by minimizing the cross-validated AUC (default fold = 10). Using the optimal weights for each base learner, the predicted log odds are generated for LASSO and ridge ($\widehat{\boldsymbol{Y}}^{(1)}$ and $\widehat{\boldsymbol{Y}}^{(2)}$ respectively). The ensemble learning algorithm estimates the optimal weights of the predictions, $\boldsymbol{\alpha} = (\alpha_1, \alpha_2)^T$, by minimizing the objective function:

$$\min_{\boldsymbol{\alpha}} -\frac{1}{n}\sum_{i=1}^{n}[Y_i \log(p_i) + (1 - Y_i)\log(1 - p_i)]^2,$$

where $p_i = \sigma\left(\sum_{j=1}^{2} \alpha_j \widehat{Y}_i^{(j)}\right)$. The final prediction of the ensemble learning algorithm is then: $\widehat{\boldsymbol{Y}}_{CV} = \alpha_1 \widehat{\boldsymbol{Y}}^{(1)} + \alpha_2 \widehat{\boldsymbol{Y}}^{(2)} = \alpha_1 \boldsymbol{w}^{(1)} \boldsymbol{PRS}_{CV} + \alpha_2 \boldsymbol{w}^{(2)} \boldsymbol{PRS}_{CV}$, where $\boldsymbol{PRS}_{CV} = [\boldsymbol{PRS}_1, \boldsymbol{PRS}_2, \ldots, \boldsymbol{PRS}_J]$."

Methods (Line 1129):

“After computing the burden score PRSs, we follow a similar ensemble learning step on the tuning dataset as in RICE-CV to compute the final PRS for RICE-RV. Using a generalized linear regression model, we combine predictions from LASSO and ridge regression to optimizing predictive performance. For rare variants, we select regularization parameters λ_1(for Lasso) and λ_2 (for ridge) via a grid search on the full tuning dataset. We use default grid values provided by glmnet[85] and choose the optimal regularization parameter based on R^2 or AUC. The final PRS for RICE-RV is then denoted as: $\widehat{\boldsymbol{Y}}_{RV} = \boldsymbol{PRS}_{RV}\, \boldsymbol{w}_{RV}$, where $\widehat{\boldsymbol{Y}}_{RV}$ is the predicted response from the rare variants. $\boldsymbol{PRS}_{RV} = [\boldsymbol{PRS}_1, \boldsymbol{PRS}_2, \dots, \boldsymbol{PRS}_J]$ represents the matrix of PRSs generated by based on the burden score, where the jth PRS, $\boldsymbol{PRS}_j = \left(PRS_{1j}, .., PRS_{nj}\right)^T$, corresponds to a PRS derived from a specific penalized regression model for n individuals. $\boldsymbol{w}_{RV}$ is the vector of optimal weights derived from the ensemble learning model.”

Comment 6: As the authors presented and discussed, a major contribution to the RV PRS came from established high-penetrance genes such as LDLR, APOB, and PCSK9 for lipids and coronary artery disease. Existing studies already examined the interactions between these genes and CV PRS (see e.g., https://www.nature.com/articles/s41467-020-17374-3). It would be interesting to examine and quantify the benefit of including additional genome-wide RV signals above and beyond known monogenic risk variants.

Thank you for this insightful comment and for highlighting the relevant prior study on monogenic-common variant interactions. We agree that quantifying the added value of genome-wide rare variant signals beyond established high-penetrance genes is crucial to demonstrate RICE-RV’s novelty.

To address this, we performed a comparative analysis using UKB imputed + WES data for four lipid traits: high-density lipoprotein (HDL), low-density lipoprotein (LDL), total cholesterol (TC), and log-transformed triglycerides (log(TG)). We compared RICE-RV (as described in the manuscript) to a baseline using only burden scores from the high-penetrance genes *LDLR*, *APOB*, and *PCSK9*, with identical ensemble learning (LASSO and ridge regression via glmnet). The results, now presented as **Supplementary Figure 14** (provided below for reference), show the standardized effect size (Beta per SD of PRS) across ancestries.

For lipid traits, RICE-RV consistently outperforms the gene-specific baseline, with 196% higher effect sizes in EUR (e.g., HDL: 0.11330 vs. 0.04455; LDL: 0.06721 vs. 0.02732) and similar gains in other ancestries, reflecting contributions from additional modest-effect rare variant sets.

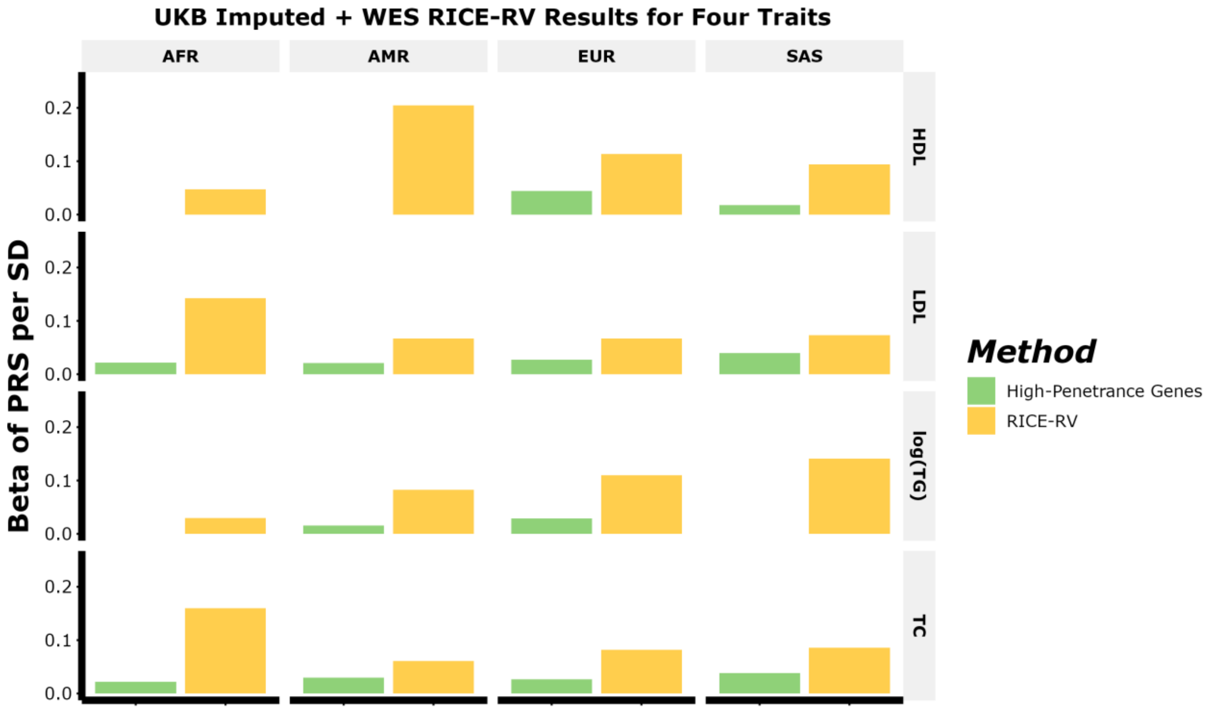

We have added this additional results in the **Results** and **Discussion** section:

Results (Line 331, UKB Imputed + WES Results):

"To quantify the benefit of genome-wide rare variant signal beyond established high-penetrance genes, we compared RICE-RV to a gene-restricted baseline that used only burden scores from *LDLR*, *APOB*, and *PCSK9* (with identical ensemble learning) for HDL, LDL, log(TG), and TC. Across ancestries, RICE-RV consistently outperformed this baseline, yielding 196% higher standardized effect size for Europeans (β per SD) (**Supplementary Figure 14**), indicating contributions from additional modest-effect rare variant sets."

Discussion (Line 547):

"A comparison to high-penetrance genes alone (*LDLR, APOB, PCSK9*) showed that RICE-RV's genome-wide approach adds substantial value for lipids (**Supplementary Figure 14**)."

This extension aligns with prior work while underscoring RICE's benefits, and the software flexibility allows domain experts to incorporate known genes explicitly. We appreciate this feedback for strengthening the manuscript.

Comment 7: The causal variants proportions in the simulations (0.01, 0.05, and 0.2) focused on relatively sparse genetic architectures. What about a more polygenic model with 1% or more causal variants?

Thank you for the comment. The causal proportion in our simulations is defined as the fraction of variants (for common) or variant sets (for rare) that are causal relative to the total analyzed. Thus, a proportion of 0.01 corresponds to 1% causal common variants and 1% causal rare variant sets, while 0.05 and 0.2 equate to 5% and 20%, respectively, covering a range from moderately to highly polygenic architectures, as detailed the **Methods** section. Our evaluations across these settings (**Figure 3**; **Supplementary Figure 1**) show robust performance, with no indication that even higher polygenicity (e.g., > 20%) would substantially alter trends.

To clarify this, we have updated the manuscript as follows:

Results (Line 208, Simulation Study Results):

"RICE was evaluated across various simulation settings, including different causal variant proportions (1%, 5%, and 20%), causality structures of causal rare variant sets (a proportion or all rare variants within a set are causal), training sample size (N = 49,173 and 98,343), and effect size distributions (strong negative selection and no negative selection, **Methods**)."

Methods (Line 1239):

"The proportion of causality varied among three levels: 1%, 5%, and 20%."

Comment 8: Would that be possible to add confidence intervals to all reported CV and RV PRS associations, e.g., those in figures 3, 4, 6 and 7? The authors claimed that RV PRS significantly improve prediction but no statistical test was performed to assess the 'significance'.

Thanks for this helpful suggestion. Because our current effect-size plots stack RICE-CV and RICE-RV bars together, adding visible CIs directly on the same bars would be visually challenging. Instead, we have incorporated statistical testing and confidence intervals in two complementary ways. For the standardized effect size (Beta per SD of PRS or log OR per SD), we tested whether the coefficient for RICE-RV differs significantly from zero. For R^2 (continuous traits) and AUC (binary traits), estimated from a joint model (RICE-CV + RICE-RV trained on tuning data and projected to validation), we computed pairwise differences between RICE and the best common variant method per trait/ancestry. Significance is denoted as ** ($p < 0.05$) or *** ($p < 0.01$) if intervals exclude zero, with full details in the updated **Evaluation of RICE** section (**Methods, Line 1143**). We have added these significance indicators to all relevant bar

plots (**Figures 3, 4, 7, 8**; **Supplementary Figures 1, 9, 11, 16, 23**) and provided full 95% CIs for all standardized effect sizes, R^2, and AUC values in **Supplementary Tables 7–10**.

We have revised the manuscript as follows:

Methods (Line 1159, Evaluation of RICE):

"We assessed the significance of the standardized effect size, R^2, and AUC using bootstrap confidence intervals. For the standardized effect-size of RICE-RV, we performed 10,000 bootstrap resamples, and tested whether the coefficient differed significantly from zero at the 95% and 99% confidence levels. For R^2 and AUC, we conducted pairwise comparisons with RICE and the best alternative method for each trait-ancestry combination. Using 10,000 bootstrap resamples, we tested whether the pairwise difference in R^2 or AUC were significantly different from zero at the 95% and 99% confidence levels."

Comment 9: Computational cost of each component of RICE should be described and reported.

Thank you for this valuable comment regarding the computational cost of RICE. We agree that reporting these details enhances the framework's transparency and usability. We have recorded the computational time for each component of RICE based on the UKB Imputed + WES analysis, as outlined in the **UKB WES, Imputed, and WGS Analysis** section (**Results, Line 358**). These timings are now available in **Supplementary Table 12**, which details the duration for tasks including rare variant association testing, ensemble learning, and other steps across traits. We have added discussion of these timings in the **UKB Imputed + WES Results** subsection of the **Results** section and provided a copy of the **Supplementary Table 12** below.

Supplementary Table 12: Timing of each step of RICE for the UKB Imputed + WES analysis in compute hours

Trait[1]	GWAS	CT	LDpred2/Lassosum2	RICE-CV	STAARpipeline	RICE-RV	RICE	Total
BMI	8.55(5.37%)	3.06(1.92%)	30.03(18.86%)	0.16(0.10%)	116.64(73.25%)	0.40(0.25%)	0.40(0.25%)	159.24
Height	7.52(5.65%)	3.04(2.28%)	27.57(20.69%)	0.17(0.13%)	94.11(70.65%)	0.43(0.32%)	0.38(0.28%)	133.21
LDL	12.64(9.02%)	2.79(1.99%)	27.16(19.38%)	0.20(0.14%)	95.59(68.21%)	0.78(0.56%)	0.98(0.70%)	140.14
HDL	9.85(6.88%)	2.96(2.07%)	28.39(19.83%)	0.18(0.13%)	99.83(69.72%)	0.92(0.64%)	1.06(0.74%)	143.19
logTG	8.91(6.93%)	2.80(2.18%)	27.27(21.20%)	0.20(0.16%)	88.09(68.51%)	0.90(0.70%)	0.42(0.32%)	128.59
TC	7.04(4.53%)	2.69(1.73%)	27.79(17.85%)	0.16(0.10%)	117.13(75.25%)	0.30(0.19%)	0.53(0.34%)	155.65
Asthma	12.46(6.92%)	3.11(1.73%)	33.13(18.39%)	1.40(0.78%)	127.44(70.74%)	1.19(0.66%)	1.41(0.79%)	180.15
Breast	10.78(7.31%)	3.04(2.06%)	32.77(22.20%)	1.60(1.09%)	94.60(64.11%)	1.84(1.24%)	2.95(2.00%)	147.57
CAD	11.47(6.03%)	3.12(1.64%)	19.13(10.07%)	1.22(0.64%)	150.79(79.35%)	2.17(1.14%)	2.14(1.13%)	190.04
Prostate	11.68(9.52%)	3.11(2.53%)	33.34(27.17%)	0.78(0.64%)	69.68(56.79%)	1.32(1.08%)	2.79(2.28%)	122.70
T2D	12.72(7.65%)	3.12(1.87%)	25.81(15.52%)	1.59(0.95%)	120.21(72.28%)	1.17(0.70%)	1.69(1.02%)	166.31

[1] Eleven unique traits are included in the analyses. Six continuous traits and five binary traits were analyzed in the UKB Imputed + WES analysis.

Results (Line 343):

“We also assessed the computational efficiency of the RICE framework (**Supplementary Table 12**). Ensemble learning for RICE-CV and RICE-RV required on average 1.73 compute hours per trait (~1.1% of total compute time). Rare variant association testing accounted for the largest compute share (106.7 hours, 381 parallel jobs, 68.6%), followed by LDpred2/Lassosum2 modeling (28.4 hours, 18.9%).”

Comment 10: I may have missed but the authors discussed two approaches for standardizing PRS in the main text -- (1) a PC-based regression approach and (2) standardizing within each ancestry. The authors stated that performance of the two approaches was consistent and the regression-based approach was presented to avoid categorizing individuals into discrete ancestry groups. However, I can't find a description of the regression-based approach in the methods section, and it seems that in many analyses PRS were still standardized separately within each ancestry. Please clarify. In additional, a mixed-ancestry tuning dataset seemed to be used in this work. Does the performance of the final PRS depend on the ancestry composition of the tuning set?

Thank you for this comment. We apologize for any oversight. The PC-based regression approach for PRS standardization is detailed in the **Supplementary Note** (**Ancestry Adjusted PRS** section, **Page 92-93**), where it adjusts for principal components to account for ancestry without discrete categorization. This method aligns with prior approaches (e.g., Ge T. et al., Genome Med., 2022; Chen T. et al., EBioMedicine, 2024). We compared it to ancestry-specific standardization (using group-specific means and SDs), finding comparable performance (**Supplementary Figure 18**), and adopted the regression-based method to avoid ancestry binning. Results are reported by ancestry to reflect population-specific effects, though the regression approach was consistently applied across analyses.

For both UKB and AoU, we employed a mixed-ancestry tuning approach (**Supplementary Tables 2 - 4** and **6**) for all evaluated methods. The performance of the final PRS can depend on the tuning set’s ancestry composition; for example, tuning solely on EUR data may underperform in East Asian validation due to ancestry mismatch, whereas tuning toward a specific ancestry can optimize performance for that group. However, this comes with a trade-off: a mixed-ancestry tuning yields a single, shareable PRS with slightly lower performance compared to ancestry-specific optimization but enhances convenience for broader use. Our cross-dataset analysis (**Evaluation of AoU PRS on UKB** subsection of the **Results**) demonstrates this robustness: despite differing ancestry compositions between AoU tuning (AFR, AMR, EUR) and UKB validation (AFR, AMR, EUR, SAS), RICE-CV and RICE-RV

performed robustly on UKB, with standardized effect sizes (**Figure 8**) and R^2 (**Supplementary Figure 25**).

Comment 11: Is there any justification for the 1x10^(-3) p-value threshold used for STAAR-Burden?

Thank you for this comment. We initially chose the 1×10^{-3} as a relaxed threshold to balance statistical validity with computational feasibility, avoiding the full genome-wide burden (e.g., analyzing all ~20,000 genes would significantly increase runtime for penalized regression and ensemble steps).

To further evaluate this choice, we explored additional thresholds: 1×10^{-5}, 1×10^{-4}, 1×10^{-3}, and 1×10^{-2} in the UKB Imputed + WES analysis for six continuous traits, modifying only the selection criterion while keeping other parameters identical. The figure below shows the standardized effect size for RICE-RV across ancestries and traits.

There is no consistent trend of improved performance with looser thresholds; for HDL and log(TG), the highest threshold (1×10^{-2}) led to decreased performance, likely due to overwhelming the ensemble regression with high-dimensional noise. Overall, 1×10^{-3} performs comparably well without noticeable drawbacks, so we retained it for all analyses. However, we recognize this may limit capture of weaker signals, and including all rare variant sets could enhance predictive power in future extensions.

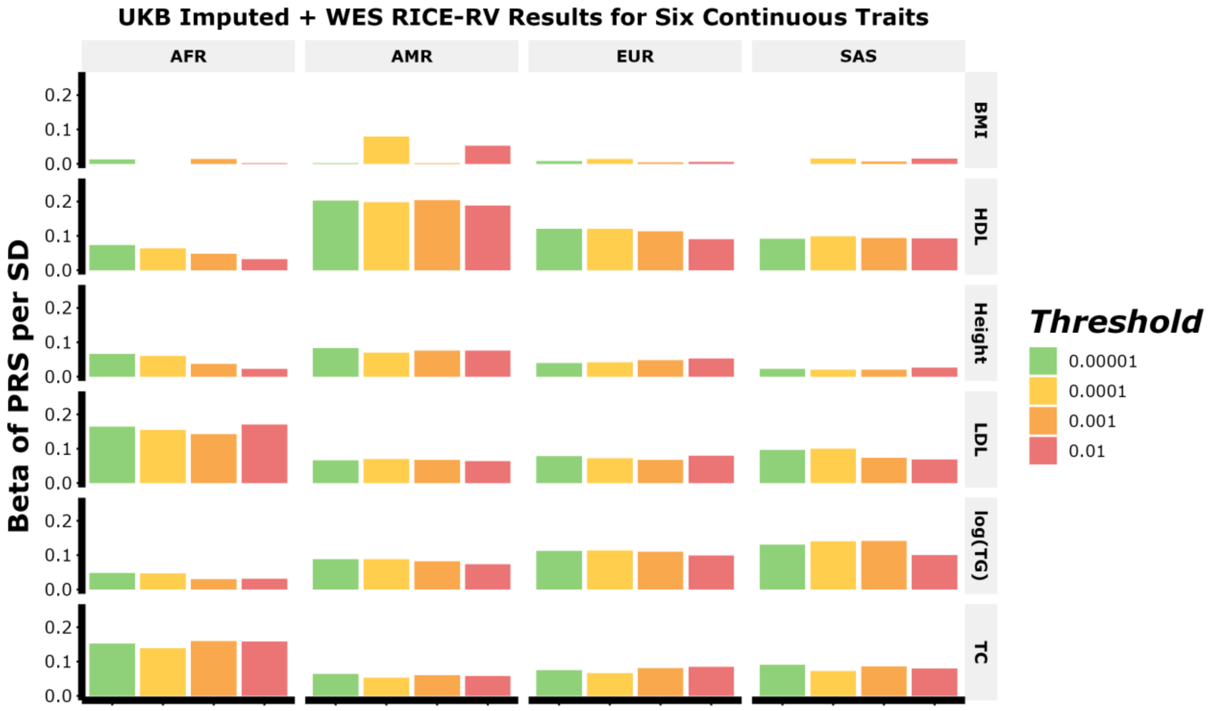

We have incorporated this change in the Results and Discussion Session as follows:

Results (Line 337, UKB Imputed + WES Results):

"We assessed sensitivity to the STAAR-Burden inclusion threshold by varying the p-value cut-off from 1×10^{-5} to 1×10^{-2}; performance was stable with no monotonic improvement at more liberal thresholds (**Supplementary Figure 15**). Balancing computational cost and signal capture, we retained 1×10^{-3} for the main analyses."

Discussion (Line 559):

"Third, rare variant sets included in the construction of RICE-RV were limited to those with a STAAR-Burden p-value less than 1×10^{-3} due to computational demands. Sensitivity analyses across alternative thresholds (1×10^{-5} to 1×10^{-2}) showed stable performance with no clear gains from looser cutoffs (**Supplementary Figure 15**), though results are limited to six continuous traits and full inclusion of all sets warrants further exploration in diverse trait types."

Comment 12: Line 269: "This suggests that rare variants can have both protective and deleterious effects, influencing an individual's risk status." I'm not sure this statement is supported by the data presented. Even if all rare variants are deleterious, similar effects can still be observed when stratifying individuals based on RV PRS quantiles, right?

We thank you for this keen observation. We agree that the statement overinterprets the quantile stratification results. To address this, we have removed the sentence from the manuscript. The revised text now focuses on the demonstrated impact:

Results (Line 292, UKB WES Results):

“Stratification by RICE-RV quantiles (below 5%, 30%–70%, and above 95%) revealed phenotypic gradients for lipid traits in EUR individuals. For HDL, the lowest quantile showed an average decrease of 0.28 standardized units, while the highest showed an increase of 0.27 units relative to the middle group (**Supplementary Figure 10b**). Similar patterns held for log(TG) (decreases of 0.25 in the lowest quantile; increase of 0.10 units in the highest; **Supplementary Figures 10e**).”

Reviewer #2 (Remarks to the Author):

This paper introduces a new PRS framework (RICE) to integrate both common and rare variants, demonstrating the benefits of including rare variants in complex trait prediction using WES and WGS datasets in UK Biobank and All of Us. With the increasing availability of WGS data, PRS methods that fully leverage WGS will become essential. Their approach of incorporating rare variants through burden scores is a reasonable attempt, and it's encouraging to see it works for some traits. However, some results may be misleading without further clarification.

We thank you for the positive feedback on RICE's effectiveness in leveraging WGS data for rare variant integration. We appreciate the insights and have revised the manuscript to clarify potential misleading aspects, as detailed below.

Comment 1: The use of rare variant burden scores for trait prediction is not novel. The authors should provide a brief background in Introduction and cite relevant prior work, such as PMID: 34615865; PMID: 36309498.

We sincerely thank you for this helpful suggestion. You are correct that rare variant burden scores have been applied to trait prediction in prior work. We have now added this background and cited the two studies you mentioned in the Introduction (PMID: 34615865; PMID: 36309498). This addition clarifies the relationship between prior work and our study, while highlighting how RICE advances beyond earlier approaches by integrating rare and common variants across ancestries and systematically evaluating them across multiple traits and two large biobanks.

We have updated the introduction as follows:

Introduction (Line 78):

"In parallel, several studies have explored the use of rare variant burden scores for risk prediction. For example, Lali et al. developed RV-EXCALIBER to integrate rare variant burden into cardiovascular disease prediction[51], while Chan et al. introduced the Genome-wide Rare Variant Score for autism spectrum disorders[52]. These approaches highlight the potential of rare variant burden scores in disease risk stratification, but they have not been broadly extended to integrate rare and common variants within a unified, multi-ancestry framework, nor have they been systematically evaluated across multiple large-scale biobanks and a wide range of complex traits."

Comment 2: Quantifying prediction accuracy using the regression slope of standardized phenotypes on standardized PRS is also not new. The lower regression slope due to imperfect (standardized) predictor is known as attenuation bias (e.g., PMID: 37491308). Such a slope is still a “average-based metric” at the cohort level - it can be shown that in this setting the slope is the correlation between y and PRS, which is the usual way of assessing prediction accuracy. It is unclear how using the standardized PRS unveil insights beyond the standard correlation, as claimed in “providing a more nuanced assessment of predictive performance”.

Thank you for this insightful comment and for highlighting the relevant reference on attenuation bias in polygenic indices (PMID: 37491308). We agree that the regression slope of standardized phenotypes on standardized PRS is equivalent to the correlation between the trait and PRS and thus represents an average-based metric at the cohort level, as you noted. In response to your comment, we have revised the manuscript to clarify that we now present this metric as a complementary and interpretable measure alongside R^2/AUC to highlight the effect size per SD increase in PRS, particularly useful for rare variants, where commonly used R^2/AUC metrics may undervalue the effects due to low carrier frequency and data sparsity (as discussed in **Results**, **Line 166**, and **Figure 2**).

Our claim of providing a “more nuanced assessment” refers to the ability of this metric to emphasize individual-level effects of rare variants. For instance, rare variant PRSs may result in substantial per-SD shifts in trait values among carriers, despite contributing minimally to overall variance explained at the population level. This individualized perspective enhances clinical interpretability and complements average-based metrics such as R^2 or AUC.

To clarify and avoid any implication of novelty, we have revised the **Results** and **Discussion** as follows:

Results (Line 166, Evaluation of Prediction Performance):

“To better capture the contribution of rare variants, we report an additional, complementary metric: the estimated regression coefficient of the standardized PRS on the standardized trait (Methods). For continuous traits, this coefficient represents the expected change in standardized phenotype per standard deviation (SD) increase in PRS. For binary traits, it corresponds to the log odds ratio (log OR) per SD increase in PRS, a widely used and interpretable measure in clinical genetics and genetic epidemiology. Throughout this manuscript, we refer to this as the standardized effect size of PRS, denoted as “Beta per SD of PRS” for continuous traits and “log OR per SD of PRS” for binary traits. As detailed in the

Supplementary Note, this coefficient can also be expressed as the square root of the heritability explained by the PRS. We use this metric to quantify the effect sizes of both common and rare variant PRSs, particularly when rare variants may have limited influence on average-based metrics, offering an interpretable per-SD effect that complements standard PRS measures like R^2 or AUC. RICE's predictive performance was evaluated using three complementary metrics: (1) standardized effect size, for assessing statistical significance and relative improvements; (2) R^2 (continuous traits) or AUC (binary traits), for absolute predictive accuracy; and (3) trait differences across PRS quantiles, to illustrate potential clinical relevance in the UKB and AoU datasets."

Discussion (Line 481):

"Traditional evaluation metrics like R^2 may underestimate the impact of rare variants because they focus on average effects across the population. The regression coefficient of the standardized PRS on the standardized trait provides a direct measure of the PRS effect size (equivalent to correlation and subject to attenuation bias due to PRS measurement error[60]), offering an interpretable assessment of predictive performance (e.g., per-SD effect). Our analyses demonstrated that, despite affecting fewer individuals, rare variants can have significant effects on trait values, reinforcing the importance of including them in PRS models."

Comment 3: A key advantage of including rare variants is the potential to identify individuals with low common variant PRS but high disease risk due to rare variants (e.g., PMID: 33110269). The authors should examine whether such individuals exist for the traits analyzed. For example, Figure 2 shows individuals with low RICE-CV PRS but high HDL phenotype. The question is whether their method can effectively capture cases with low CV PRS but high RV PRS.

We sincerely thank you for this excellent suggestion and for referencing the study by Lu et al. (2021), which highlights the importance of identifying individuals with low polygenic risk but elevated disease likelihood due to rare variants. We fully agree that this is a critical advantage of rare variant integration, enabling prioritized screening that would otherwise be missed by common variant PRS alone.

We examined all six continuous traits, focusing on whether group 3 (low CV + high RV; (1) Low CV / Low RV, (2) High CV / Low RV, and (4) High CV / High RV) individuals captured additional extreme phenotypes beyond those identified by CV PRS alone. For HDL (provided below, added as **Figure 5b,c**), among individuals in the top 10% of observed HDL values, 6.3% fell into group 3, meaning they were missed by CV PRS alone but captured by RV PRS. Moreover,

mean standardized HDL levels were significantly higher in groups 2, 3, and 4 compared to group 1, with group 3 showing a clear elevation despite low CV PRS ($p = 3.41 \times 10^{-14}$). Similar but more modest patterns were observed for LDL, TC, and log(TG), whereas results for BMI were not significant (**Supplementary Figure 13**), consistent with our earlier effect-size findings where RICE-RV showed no gain for BMI (**Figure 4**).

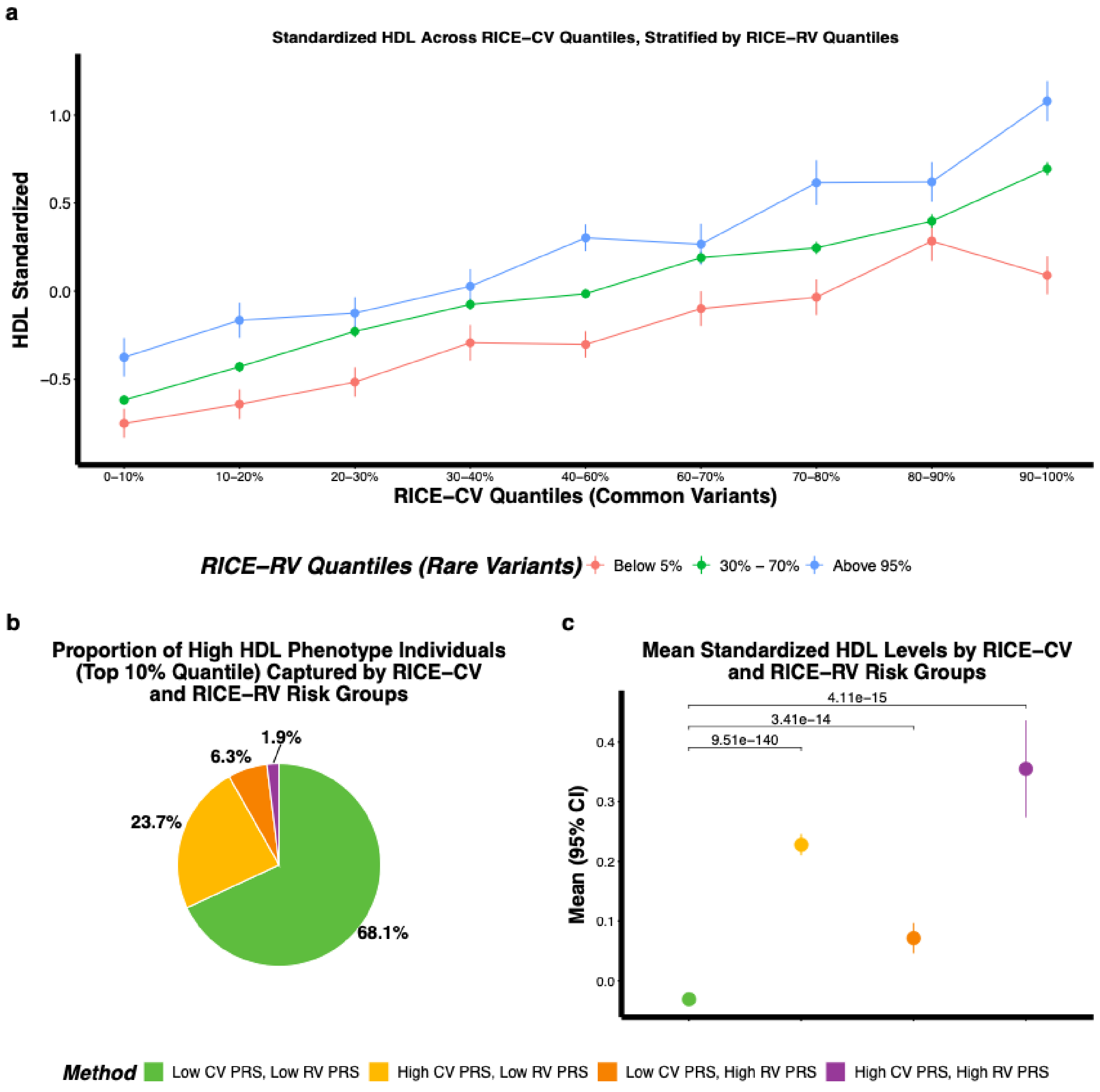

Figure 5. Joint stratification of common and rare variant PRS identifies discrepant high-risk individuals. Individuals in the UKB imputed + WES validation dataset were stratified based on rare variant PRS (RV; high risk = top 5%). (a) mean standardized HDL levels $\pm 1\ \times$ standard

error (SE) are plotted stratified by PRS quantiles for RICE-CV (common variants) on the x-axis and RICE-RV (rare variants) by color (red: below 5%, green: 30–70%, blue: above 95%). (b) Further stratification by common variant PRS (CV; high risk = top 10%) created four strata: (1) Low CV / Low RV, (2) High CV / Low RV, (3) Low CV / High RV, and (4) High CV / High RV. Proportion of individuals in each group among the top 10% of observed HDL values. (c) Under same grouping as (b), mean standardized HDL levels with 95% confidence intervals and pairwise p-values across the four groups.

We have added this to the manuscript:

Results (Line 319, UKB Imputed + WES Results):

"To test whether rare variants flag individuals missed by common variant PRS, we cross-classified participants by RICE-CV (top 10% vs. bottom 90%) and RICE-RV (top 5% vs. bottom 95%), yielding four strata (**Methods**). For HDL, stratifying by RICE-RV quantiles shows significant differences across RICE-CV quantiles (**Figure 5a**). Further, 6.3% of individuals in the top phenotype decile were captured only by high RICE-RV despite low RICE-CV (**Figure 5b**). Group-level comparisons confirmed that the low-CV/high-RV stratum had significantly higher HDL than the low-CV/low-RV group ($p = 3.41 \times 10^{-14}$; **Figure 5c**). Similar patterns were seen for other lipid traits but not for BMI, consistent with the standardized effect-size results (**Supplementary Figure 13**). These findings show that the rare variant component of RICE can identify individuals with extreme lipid profiles who would be missed by common variant PRS alone."

Comment 4: The burden scores approach implicitly assumes that causal rare alleles are proportional to the non-causal rare alleles within the region. This method relies on the assumption that regions with higher rare variant counts (high SNP burden score) also contain proportionally more causal variants (high causal burden score). They should evaluate whether high burden scores introduce systematic bias when causal burden scores do not proportionally align with SNP burden scores. They could do a simulation with varying proportions of causal rare variants across regions/SNP sets.

Thank you for this thoughtful comment. We agree that the burden scores approach assumes proportionality between causal and non-causal rare alleles within regions, and evaluating potential bias under varying causality structures is important for robustness. To address this, we expanded our simulations to include scenarios where only a proportion of rare variants within causal rare variant sets are causal (**Methods**, **Line 1240**), allowing us to test non-proportional

alignments between SNP burden and causal burden. Results for these new scenarios are shown in **Supplementary Figures 1e–1h**. Comparing them to the original simulations (**Figure 3** and **Supplementary Figures 1a–1d**), RICE-RV's predictive performance remains consistent across causality structures, suggesting that both STAARpipeline and the rare variant ensemble learning are robust to such variations.

We have updated the manuscript as follows:

Results (Line 208, Simulation Study Results):

"RICE was evaluated across various simulation settings, including different causal variant proportions (1%, 5%, and 20%), causality structures of causal rare variant sets (a proportion or all rare variants within a set are causal), training sample size (N = 49,173 and 98,343), and effect size distributions (strong negative selection and no negative selection, **Methods**)."

Methods (Line 1240, Simulation Study):

"We explored two different strategies of assigning causality to rare variants within a causal rare variant set. First, all rare variants within a rare variant set are causal and used in the construction of the causal burden score. Second, a proportion ($\pi_i \sim Uniform\ (0.1, 0.9)$) of rare variants within causal rare variant set i are used in construction of the causal burden score."

Comment 5: Their claim of adequate quality control is not convincingly supported by the data. Line 273 "Quality control assessments for UKB WES association analyses were performed using Manhattan and QQ plots, confirming well-calibrated p-values and controlled inflation factors (Supplementary Figures 7–9)" However, Supplementary Figure 8 shows substantial inflations in the QQ plots, as observed P-values deviate significantly from expected values at the beginning.

Thank you for raising this important point. We agree that careful assessment of potential inflation is critical for association studies underlying PRS analyses. In the revised **Supplementary Figures**, the original **Supplementary Figure 8** is now **Supplementary Figure 7**. It is important to note that deviations in QQ plots do not necessarily indicate uncontrolled inflation. As shown by Bulik-Sullivan et al. (2015, PMID: 25642630), such deviations can arise from (1) the polygenic nature of complex traits, particularly in large samples (e.g., height), or (2) unadjusted population stratification. To formally evaluate inflation, we reported two standard metrics: (i) the adjusted genomic inflation factor (λ_{1000}, scaled to a study with 1000 subjects or 1000 cases and 1000 controls for continuous and binary traits respectively), which accounts for

large sample sizes, and (ii) the LD-score regression intercept, which helps distinguish polygenicity from confounding. Across all UKB and AoU analyses, λ_{1000} values were < 1.018, and LDSC intercepts were < 1.2, except for height in the EUR WES analysis (1.27; **Supplementary Table 5**), indicating minimal population stratification.

In response to this and other reviewer suggestions (Reviewer 3, Comment 7), we additionally compared multiple association models: 1. REGENIE, a whole genome regression model designed to control for population stratification (Mbatchou J., et al., Nat. Genet., 2021, PMID: 34017140), 2. linear regression with varying numbers of PCs, 3. Linear regression with rank-based inverse normal transformation). We have provided the results in the table below. Inflation metrics were stable across models, with the exception of height in the EUR WES data. As LDSC was originally developed for common variants across the genome and has not been benchmarked on exome-only analyses, the elevated intercept for height may reflect a limitation of LDSC in this context rather than true stratification. Notably, LDSC intercepts were well controlled for height in UKB imputed (1.15) and WGS (1.15) data.

Trait	Method	Intercept (SE)
BMI	regenie 10 PCs	1.1266 (0.0431)
BMI	plink 10 PCs	1.1225 (0.0409)
BMI	plink 20 PCs	1.1245 (0.0415)
BMI	plink 40 PCs	1.1216 (0.0411)
BMI	plink rank normal 10 PCs	1.1273 (0.0387)
Height	regenie 10 PCs	1.2730 (0.0462)
Height	plink 10 PCs	1.2270 (0.0441)
Height	plink 20 PCs	1.2074 (0.0452)
Height	plink 40 PCs	1.2066 (0.0444)
Height	plink rank normal 10 PCs	1.2273 (0.0442)
HDL	regenie 10 PCs	1.0904 (0.0397)
HDL	plink 10 PCs	1.0564 (0.0351)
HDL	plink 20 PCs	1.0554 (0.0350)
HDL	plink 40 PCs	1.0566 (0.0352)
HDL	plink rank normal 10 PCs	1.0549 (0.0366)
LDL	regenie 10 PCs	1.0434 (0.0413)
LDL	plink 10 PCs	1.0303 (0.0389)
LDL	plink 20 PCs	1.0296 (0.0390)
LDL	plink 40 PCs	1.0293 (0.0389)
LDL	plink rank normal 10 PCs	1.0330 (0.0387)
logTG	regenie 10 PCs	1.1183 (0.0404)
logTG	plink 10 PCs	1.1057 (0.0389)
logTG	plink 20 PCs	1.1063 (0.0388)
logTG	plink 40 PCs	1.1061 (0.0385)
logTG	plink rank normal 10 PCs	1.1100 (0.0401)
TC	regenie 10 PCs	1.0256 (0.0359)
TC	plink 10 PCs	1.0155 (0.0336)
TC	plink 20 PCs	1.0146 (0.0337)
TC	plink 40 PCs	1.0147 (0.0336)
TC	plink rank normal 10 PCs	1.0167 (0.0334)

To ensure robustness, and following Reviewer 3's suggestion, we updated all GWAS analyses in UKB and AoU to use REGENIE (Mbatchou J., et al., Nat. Genet., 2021, PMID: 34017140). The revised Results now emphasize both λ_{1000} and LDSC intercepts, alongside Manhattan and QQ plots, to show that inflation was well controlled across traits and ancestries.

We have updated the manuscript as follows:

Results (Line 244, UKB WES, Imputed and WGS Results):

"Genomic control metrics indicated well-calibrated analyses, with λ_{1000} ranging between 1 to 1.018. LD score regression intercepts[56] further confirmed minimal population stratification (intercept < 1.2) across all traits, with the exception of height in the EUR WES analysis (LDSC intercept = 1.27, **Supplementary Table 5**). Manhattan and QQ plots for common and rare variant association tests are available for all three analyses: WES (**Supplementary Figures 3-4**), Imputed + WES (**Supplementary Figures 5-6**), and WGS (**Supplementary Figures 7-8**)."

Results (Line 401, All of Us Results):

"The genomic inflation factor was well controlled, with λ_{1000} ranging from 1 to 1.003 (**Supplementary Table 5**), with LD score regression intercepts[56] confirming minimal population stratification across traits and ancestries. Manhattan and QQ plots for common and rare variant association tests are provided in **Supplementary Figures 21-22**."

Methods (Line 961, Phenotype and PRS Standardization):

"Association testing of common variants was performed with REGENIE[84]."

Comment 6: Some results are unclear and difficult to interpret:
Comment 6a: In UKB WES analysis, what explains the large variation in RICE-RV between ancestries, such as AFR and SAS for BMI (Figure 4)? The discrepancies are much larger than those in the simulation.

Thank you for the comment. We have since updated our GWAS analysis from a linear model using PLINK to a whole genome regression model using REGENIE based on comments from reviewers and subsequently reran all UKB and AoU analyses in the manuscript. Further, we have moved the UKB WES analysis results to the Supplementary material due to added analyses and restructure of the manuscript. The referred **Figure 4** from the prior version is now moved to **Supplementary Figure 9c** (standardized effect size of six continuous traits in UKB WES analyses).

The primary reason for the large discrepancies between ancestries are due to limited sample size in non-European ancestries in UKB analyses. The effect-size estimate itself could have wide confidence intervals (**Supplementary Table 7**). For example, the 95% confidence interval for the estimated coefficient of the standardized PRS for RICE-RV is nearly 3 times wider for AFR than for EUR and over 2 times wider for SAS than EUR. Following Reviewer 1's suggestion, we have added significance testing to test the standardized effect size of RICE-RV in all analyses. As shown in this analysis, **Supplementary Figure 9c**, the rare variant effects of BMI are non-significant.

We have revised the manuscript and emphasized the assessed of significance in the **Results** section

Results (Line 286, UKB WES Results):

"For anthropometric traits, RICE significantly improved R^2 for both height and BMI in EUR, but the rare variant component was significant only for height (**Supplementary Figure 9c**)."

Comment 6b: Why is prediction seemingly driven entirely by RICE-RV for some trait, such as Breast cancer in AFR and Prostate cancer in AMR (Supplementary Figure 3)?

Thank you for this helpful observation. In the revised manuscript, the original **Supplementary Figure 3** is now **Supplementary Figure 9a** (standardized effect sizes of binary traits in UKB WES analyses). While some binary traits appeared to show stronger contribution from RICE-RV in the prior version, our updated analyses with added significance testing indicate that the standardized effect sizes of RICE-RV are not significant for any binary trait across ancestries. This is largely due to the limited number of cases available for binary traits in UK Biobank, which substantially reduces power for rare variant PRS analyses. Our primary goal in including binary traits here was to demonstrate that the RICE framework can be applied to both continuous and binary traits, rather than to make definitive claims for specific diseases. We agree that meaningful evaluation of binary traits will require substantially larger sample sizes, such as those available through disease-specific consortia.

We have clarified these points in the manuscript:

Results (Line 289, UKB WES analyses):

"In contrast, binary traits exhibited minimal improvements (**Supplementary Figures 9a–b**), likely due to limited number of cases."

Discussion (Line 564):

"Fourth, smaller case counts for binary outcomes limit the utility of current biobank data for rare variant PRS. Future applications of RICE in large disease-specific consortia may better capture rare variant contributions to binary traits, and could incorporate complementary metrics such as net reclassification index to assess improvements in high-risk individual identification and clinical decision-making."

Comment 6c: why does RICE-RV contribute substantially to BMI in UKB WES data (Figure 4) but not in UKB WGS data (Supplementary Figure 10), given that UKB WGS include WES?

Thank you for the thoughtful question. In the revised manuscript, the original **Figure 4** is now **Supplementary Figure 9c** (UKB WES, standardized effect sizes for continuous traits), the original **Supplementary Figure 10** is now **Supplementary Figure 16c** (UKB WGS,

standardized effect sizes for continuous traits), and we additionally include **Figure 4** for the Imputed + WES configuration. After re-running all GWAS with REGENIE and adding formal significance testing, we find that the RICE-RV standardized effect size for BMI is not significant in almost all configurations, WES, Imputed + WES, or WGS. Thus, the earlier appearance of a "substantial" rare variant contribution to BMI in WES reflects sampling variability and the previous analysis setup rather than a robust signal. The WGS results are consistent with the updated WES findings.

The corresponding results have been incorporated into the manuscript as follows:

Results (Line 286, UKB WES Results):

"For anthropometric traits, RICE significantly improved R^2 for both height and BMI in EUR, but the rare variant component was significant only for height (**Supplementary Figure 9c**)."

Results (Line 326, UKB Imputed + WES Results):

"Similar patterns were seen for other lipid traits but not for BMI, consistent with the standardized effect-size results (**Supplementary Figure 13**)."

Results (Line 354, UKB WGS Results):

"For rare variants, RICE-RV showed significant effects ($p < 0.05$) for height and all lipid traits across ancestries, with BMI only being significant for SAS (**Supplementary Figure 16c**)."

Comment 6d: RICE-RV have nearly zero effects on breast cancer in both UKB WES and WGS data of European ancestry (Supplementary Figure 3 and 10), despite the known contributions of large-effect alleles (e.g., BRACA1/2). How do the authors reconcile this?

Thank you for this helpful observation. In the revised manuscript, the original **Supplementary Figure 3** is now **Supplementary Figure 9a** (UKB WES, standardized effect sizes for binary traits) and the original **Supplementary Figure 10** is now **Supplementary Figure 16a** (UKB WGS, standardized effect sizes for binary traits).

After re-running GWAS with REGENIE and adding formal uncertainty assessment, we find that RICE-RV standardized effect sizes for all binary traits are not significant in either WES or WGS. This reflects limited statistical power for rare variant PRS in UKB's breast cancer data rather than a contradiction with known biology. In our UKB WES analysis we have 71,494 unrelated participants including 3,458 breast cancer cases (remaining participants are controls). After random 3-way splitting, the training set contains 2,741 cases and 51,305 controls. With a

BRCA1/2 carrier frequency ~1:400-1:800 in the general population, we expect only around 3-7 carriers among cases in the training split. Such sparsity yields wide CIs and insufficient power to detect a cohort-wide per-SD effect for the rare variant PRS. UKB is not enriched for heredity of breast cancer.

However, even with limited case counts, some of the known breast cancer genes are still detected and included by RICE-RV at the 1×10^{-3} threshold, such as *BRCA2* ($p = 4.7 \times 10^{-4}$, putative loss of function category) and *PALB2* ($p = 1.6 \times 10^{-4}$, putative loss of function category) as shown in **Supplementary Data** (Breast UKB WGS tab). However, their extreme rarity limits the aggregate predictive effect, so the RICE-RV coefficient appears near zero in EUR WES/WGS despite known pathogenicity.

In summary, our aim is to show that RICE is applicable to both continuous and binary traits; in the current UKB/AoU datasets the largest, statistically robust gains occur for HDL, LDL, log(TG), TC, and height, whereas binary outcomes show limited improvements given small case counts and the rarity of high-penetrance alleles. We have updated the manuscript as follows:

Results (Line 289, UKB WES Results):

"In contrast, binary traits exhibited minimal improvements (**Supplementary Figures 9a–b**), likely due to limited number of cases."

Results (Line 359, UKB WGS Results):

"In contrast, binary traits again showed limited improvement."

Discussion (Line 564)

"Fourth, smaller case counts for binary outcomes limit the utility of current biobank data for rare variant PRS. Future applications of RICE in large disease-specific consortia may better capture rare variant contributions to binary traits, and could incorporate complementary metrics such as net reclassification index to assess improvements in high-risk individual identification and clinical decision-making."

Comment 6e: For some traits, both RICE-CV and RICE-RV have nearly zero prediction accuracy is nearly zero in both, such as Breast cancer in SAS and CAD in AFR (Supplementary Figure 10). Since their RICE-CV is based on ensemble learning that combines predictors from existing methods, how/why does it perform much worse than existing methods?

Thank you for raising this. In the revised manuscript, the original **Supplementary Figure 10** is now **Supplementary Figure 16a** (UKB WGS, standardized effect sizes for binary traits). For the two examples you highlight, breast cancer in SAS and CAD in AFR, the point estimates of prediction accuracy are close to zero for all common variant methods. As shown in **Supplementary Table 9**, the 95% CIs for CT, LDpred2, Lassosum2, and RICE-CV overlap and typically include zero.

This pattern is expected. These are binary traits with few cases and strong class imbalance in the relevant ancestry groups, so the underlying summary-statistic signal is weak and noisy. In such regimes, an ensemble (stacking with regularization) is estimated on a small, imbalanced tuning set; the weights shrink toward conservative combinations, and the ensemble will often match, but not exceed, the best base learner by more than sampling error. Cross-ancestry transfer also limits attainable accuracy for AFR/SAS because LD and allele-frequency differences reduce portability of effects derived largely from EUR discovery data. Where the signal is stronger (e.g., lipids, height), RICE-CV tracks or exceeds the alternatives as expected.

Comment 7: Line 269 “This suggests that rare variants can have both protective and deleterious effects, influencing an individual's risk status.” Line 286 “Incorporating RICE-RV helped to identify individuals whose trait values were significantly impacted by rare variants, which would have been missed by common variant PRSs alone.” However, the figures (Fig S6 and Fig 5) do not seem to directly support these conclusions. Again, a more direct approach could be to identify cases with low RICE-CV but high RICE-RV.

Thank you for pointing this out. We agree that our original wording overstated what the quantile plots support. We have removed the sentence “This suggests that rare variants can have both protective and deleterious effects, influencing an individual’s risk status.” and revised the text to describe the observed phenotypic gradients without over-interpreting directionality.

Results (Line 292, UKB WES Results):

“Stratification by RICE-RV quantiles (below 5%, 30%–70%, and above 95%) revealed phenotypic gradients for lipid traits in EUR individuals. For HDL, the lowest quantile showed an average decrease of 0.28 standardized units, while the highest showed an increase of 0.27 units relative to the middle group (**Supplementary Figure 10b**). Similar patterns held for log(TG) (decreases of 0.25 in the lowest quantile; increase of 0.10 units in the highest; **Supplementary Figures 10e**).”

To address your suggestion directly, we have added additional analyses to show the number of individuals identify by low RICE-CV and high RICE-RV. We examined all six continuous traits, focusing on whether group 3 (low CV + high RV; (1) Low CV / Low RV, (2) High CV / Low RV, and (4) High CV / High RV) individuals captured additional extreme phenotypes beyond those identified by CV PRS alone. For HDL (provided below, added as **Figure 5**), among individuals in the top 10% of observed HDL values, 6.3% fell into group 3, meaning they were missed by CV PRS alone but captured by RV PRS. Moreover, mean standardized HDL levels were significantly higher in groups 2, 3, and 4 compared to group 1, with group 3 showing a clear elevation despite low CV PRS ($p = 3.41 \times 10^{-14}$). Similar but more modest patterns were observed for LDL, TC, and log(TG), whereas results for BMI were not significant (**Supplementary Figure 13**), consistent with our earlier effect-size findings where RICE-RV showed no gain for BMI (**Figure 4**).

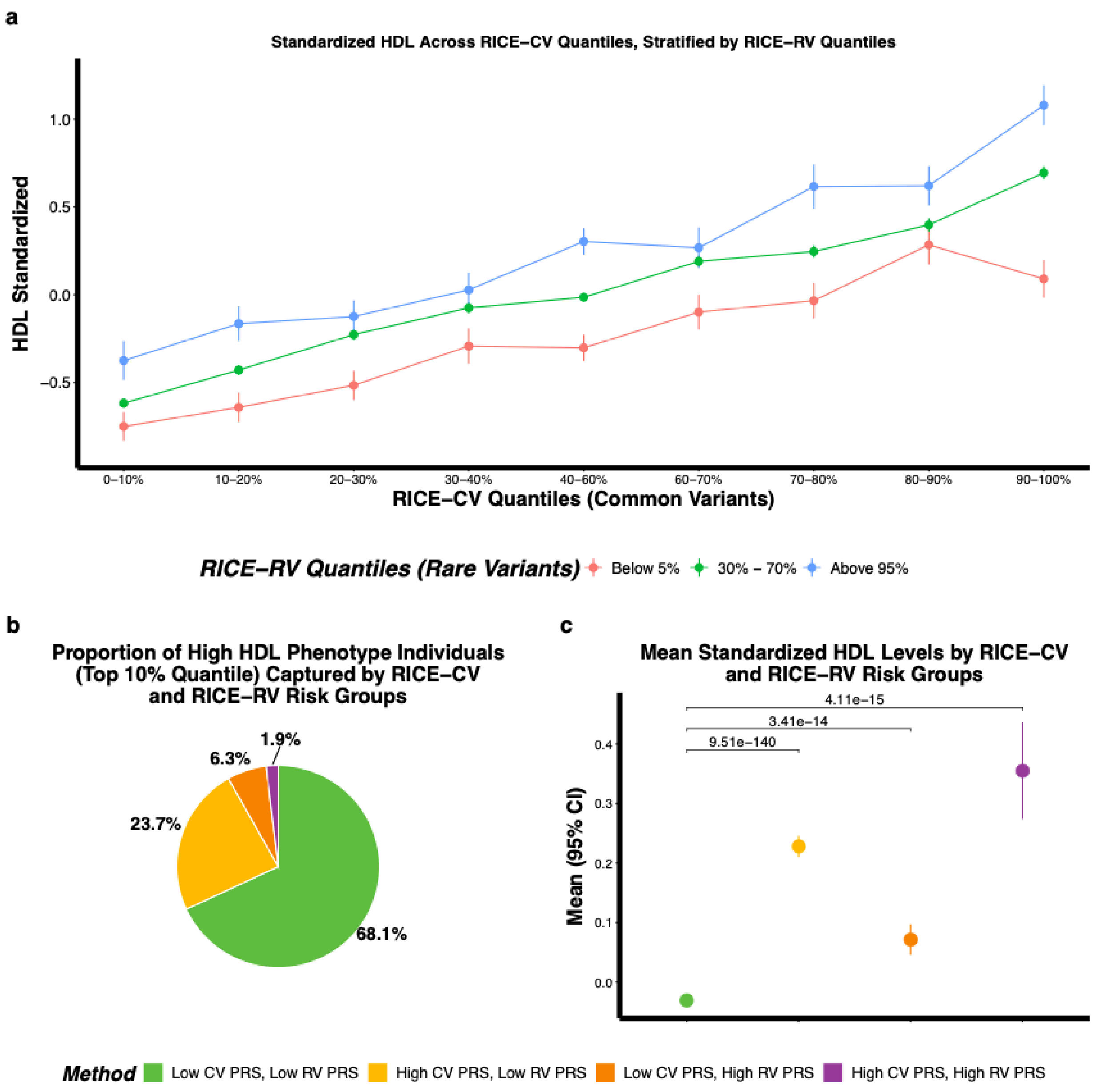

Figure 5. Joint stratification of common and rare variant PRS identifies discrepant high-risk individuals. Individuals in the UKB imputed + WES validation dataset were stratified based on rare variant PRS (RV; high risk = top 5%). (a) mean standardized HDL levels $\pm 1 \times$ standard error (SE) are plotted stratified by PRS quantiles for RICE-CV (common variants) on the x-axis and RICE-RV (rare variants) by color (red: below 5%, green: 30–70%, blue: above 95%). (b) Further stratification by common variant PRS (CV; high risk = top 10%) created four strata: (1) Low CV / Low RV, (2) High CV / Low RV, (3) Low CV / High RV, and (4) High CV / High RV. Proportion of individuals in each group among the top 10% of observed HDL values. (c) Under

same grouping as (b), mean standardized HDL levels with 95% confidence intervals and pairwise p-values across the four groups.

We have added this to the manuscript:

Results (Line 319, UKB Imputed + WES Results):

"To test whether rare variants flag individuals missed by common variant PRS, we cross-classified participants by RICE-CV (top 10% vs. bottom 90%) and RICE-RV (top 5% vs. bottom 95%), yielding four strata (**Methods**). For HDL, stratifying by RICE-RV quantiles shows significant differences across RICE-CV quantiles (**Figure 5a**). Further, 6.3% of individuals in the top phenotype decile were captured only by high RICE-RV despite low RICE-CV (**Figure 5b**). Group-level comparisons confirmed that the low-CV/high-RV stratum had significantly higher HDL than the low-CV/low-RV group ($p = 3.41 \times 10^{-14}$; **Figure 5c**). Similar patterns were seen for other lipid traits but not for BMI, consistent with the standardized effect-size results (**Supplementary Figure 13**). These findings show that the rare variant component of RICE can identify individuals with extreme lipid profiles who would be missed by common variant PRS alone."

Comment 8: They introduce multi-ancestry methods for RICE-CV PRS, but surprisingly, they do not include these methods in comparison. It's unclear whether RICE-CV outperforms the existing multi-ancestry methods.

Thank you for raising this. To clarify, we evaluated multi-ancestry PRS methods in the AoU analyses, where ancestry composition is more heterogeneous, and reported the corresponding results in **Figure 7** (standardized effect sizes) and **Supplementary Figure 23** (R^2). In contrast, the UK Biobank (UKB) analyses use discovery and training sets that are predominantly European; to avoid conflating method differences with ancestry imbalance, we kept UKB training EUR-only and therefore did not run multi-ancestry competitors in that setting.

In AoU, RICE-CV (multi-ancestry configuration) was compared directly with CT-SLEB, JointPRS, and PROSPER using identical GWAS summary statistics and matched tuning/validation splits. Across most trait-ancestry pairs, RICE-CV matched or modestly exceeded the best existing method (**Figure 7**). Two exceptions were HDL in MID and BMI in SAS, where JointPRS performed better, likely reflecting smaller validation sample sizes in those groups. The results are described in the manuscript as follows:

Results (Line 407, All of Us Results):

"Performance patterns largely mirrored UKB (standardized effect sizes in **Figure 7;** R^2 in **Supplementary Figure 23;** quantile differences in **Supplementary Figure 24**). Among common variant methods, RICE-CV showed robust performance across most trait–ancestry pairs, typically matching or modestly exceeding the best alternatives (**Figure 7**). Two exceptions were HDL in MID and BMI in SAS, where JointPRS performed better, likely reflecting smaller validation sample sizes in these populations. The rare variant component RICE-RV contributed significantly ($p < 0.05$) for several traits, with the clearest signals in lipids and height, though patterns varied by ancestry. Significance was observed in: EUR (HDL, height, LDL, log(TG)); AFR (HDL, height, log(TG), TC); AMR (HDL, height, LDL, log(TG)); EAS (HDL, height, log(TG)); MID (HDL, TC); and SAS (log(TG) only). No ancestry showed a significant RV effect for BMI. Consistent with this, the RV contribution to standardized effects was substantial for lipids, for example, for HDL, the RICE-RV coefficient was ~26-31% of the RICE-CV coefficient in EUR and ~24-40% across non-EUR ancestries; for log(TG), the corresponding ratios were ~22-36.5% (EUR) and ~32-70% (non-EUR). For explained variance, the full RICE model (CV+RV) improved R^2 by an average of 1.7% over the best existing method across trait-ancestry pairs (**Supplementary Figure 23**). Notable gains included 14.2% for height in EUR, 43.2% for HDL in EAS, and 18.5% for TC in AFR. In contrast to UKB, we did not observe significant R^2 gains for lipid traits in EUR within AoU, likely reflecting smaller training sample sizes (average N = 65,549 per lipid trait in AoU vs. N = 91,356 in UKB)."

Comment 9: Line 105: "independent individuals". More common to say "unrelated individuals".

Thank you for the suggestion. In the revised manuscript, the original Line 105 is now at **Line 96**. We have updated **Line 96** to the following:

"We evaluated RICE using extensive simulated datasets and large-scale WES, Imputed, and WGS data from UKB and AoU, with up to 740 million genetic variants from 361,939 unrelated individuals across African (AFR), Admixed American or Latino (AMR), European (EUR), Middle Eastern (MID) and South Asian (SAS) ancestries."

Comment 10: Line 954: "For the k-th rare variant" should be "k-th rare variant set".

Thank you for the suggestion. In the revised manuscript, the original Line 954 is now **Line 1099**. We have updated **Line 1099** to the following:

"For the k-th rare variant set ($k = 1, 2, \ldots, K$), let m_k be the number of rare variants in the kth set."

Comment 11: Line 989-992: The notation PRS_j is unclear. It appears to correspond to a specific lambda value in line 969 and 977, but this should be clarified, along with the range of lambda tested.

Thank you for the comment. In the revised manuscript, the original Line 989-992 is now **Line 1137-1140**.

The jth PRS corresponds to a PRS created from one of the existing methods under their grid of hyperparameters which we refer to as a tuning parameter set. We have updated the section as follows to clarify it:

Methods (Line 1136, Ensemble learning to combine PRSs for RICE-RV.)

"The final PRS for RICE-RV is then denoted as: $\widehat{\boldsymbol{Y}}_{RV} = \boldsymbol{PRS}_{RV}\, \boldsymbol{w}_{RV}$, where $\widehat{\boldsymbol{Y}}_{RV}$ is the predicted response from the rare variants. $\boldsymbol{PRS}_{RV} = [\boldsymbol{PRS}_1, \boldsymbol{PRS}_2, \ldots, \boldsymbol{PRS}_J]$ represents the matrix of PRSs generated by based on the burden score, where the jth PRS, $\boldsymbol{PRS}_j = \left(PRS_{1j}, .., PRS_{nj}\right)^T$, corresponds to a PRS derived from a specific penalized regression model for n individuals."

We have also added additional details on the grid of lambdas in the ensemble learning step.

Methods (Line 1133, Ensemble learning to combine PRSs for RICE-RV.)

"For rare variants, we select regularization parameters λ_1(for Lasso) and λ_2 (for ridge) via a grid search on the full tuning dataset. We use default grid values provided by glmnet[88] and choose the optimal regularization parameter based on R^2 or AUC."

Comment 12: The Methods section contains many subsections, which should be grouped to improve readability.

We thank the reviewer for this helpful suggestion. We agree that the original **Methods** section was overly fragmented, which could hinder readability. In response, we have reorganized the **Methods** into broader thematic categories while ensuring sufficient detail for reproducibility. Specifically, the section now begins with unified descriptions of data processing and modeling strategies, followed by evaluation procedures and dataset-specific applications. The revised structure is as follows:

1. Data Processing and Standardization (including dataset splitting, phenotype/PRS standardization)
2. Common Variant PRS Modeling (RICE-CV) (PRS training and ensemble learning)

3. Rare Variant PRS Modeling (RICE-RV) (null model and association testing, burden score construction and penalized regression, ensemble learning)
4. Evaluation of RICE (performance metrics, bootstrap testing, portability framework)
5. Data Analysis Sections, which include:
   - Existing PRS Methods
   - Simulation Studies
   - UKB WES, Imputed, and WGS Analysis
   - AoU Analysis
   - Cross-Dataset Portability Analysis

This restructuring reduces fragmentation in the **Methods** while keeping dataset-specific analyses distinct, as they involve different data sources and implementation details that would be less clear if fully merged. We believe this strikes a balance between improved readability and sufficient methodological transparency.

Reviewer #3 (Remarks to the Author):

Williams et al. present an approach for integrating rare variants with common variants for polygenic risk score estimation in large-scale genetic datasets. They introduce a pipeline tool, RICE, which incorporates the results of multiple existing tools to learn ensemble scores leveraging common and rare variants in two steps. RICE is utilized in the WGS and WES in the UK Biobank, as well as the All-of-Us dataset, and the authors demonstrate an overall performance improvement across almost all traits based on a novel metric proposed in the manuscript. We find the manuscript interesting but have major concerns regarding choices in modeling design and statistical approach.

Comment 1: First, a major issue is that the performance is reported on validation data and not on held-out test data. Here test data would usually be a completely unseen data set to evaluate how well the prediction models generalize and is standard for evaluation of machine learning models. Without evaluation on a held-out test set, it is not possible to compare the performance of RICE to other models.

We thank the reviewer for raising this important point. We apologize for any confusion caused by our terminology. In our study, we implemented RICE and all comparison PRS methods using a three-way data split into training, tuning, and validation sets. The training dataset (~70% of the sample) was used to compute GWAS summary statistics, fit rare variant association tests, and train initial PRS models. The tuning dataset (~15%) was used exclusively to tune hyperparameters and to fit the ensemble learning step. The validation dataset (~15%) was a held-out, fully independent dataset, never used in training or tuning, and served to evaluate final model performance (e.g., R^2/AUC, and standardized effect sizes).

Thus, our “validation set” corresponds to what is typically referred to as a held-out test set in machine learning. To avoid misunderstanding, we have revised the manuscript to explicitly state that the validation dataset is independent and held out from all model development steps.

Methods (Line 947, Data Processing and Standardization):

“All analyses employ a three-way data split into independent training, tuning, and validation datasets. The training dataset is used to compute GWAS summary statistics, train common variant PRS models, perform rare variant association testing, compute burden scores, and train rare variant PRS models using penalized regression (LASSO and ridge regression). The tuning dataset is used to train the ensemble learning model, combining PRSs generated by different

methods and tuning parameters, using LASSO and ridge regression as base learners. The final validation dataset is a fully independent, held-out test set used only to evaluate the performance of the final PRSs."

Comment 2: Related to this, we find that the choice of terminology regarding the data subsets is rather confusing. In the manuscript, the terms training, tuning, and validation are used. The standard is to use training, validation, and test. As mentioned above, the test data would be a completely unseen data set to evaluate how well the prediction models generalize.

We thank the reviewer for pointing this out. We recognize that there is variability in terminology across the PRS and statistical genetics literature. While the standard in machine learning is training / validation / test, several widely used PRS methods employ slightly different naming conventions: 1. CT-SLEB (Zhang H. et al. Nat. Genet., 2023) and PROSPER (Zhang J. et al., Nat Commun., 2024) use the terms training / tuning / validation, 2. JointPRS (Xu L., et al., Nat Commun., 2025) use the terms, training / tuning / testing, and 3. PRS-CSx (Ruan Y. et al., Nat. Genet., 2022) use the terms training / validation / testing.

Given this lack of consensus, we followed the terminology of CT-SLEB and PROSPER for consistency with recent PRS frameworks. For this manuscript, we have retained the terms training / tuning / validation to remain consistent with the literature and with how other reviewers have referred to our work. However, to prevent any misunderstanding, we have now clarified in the **Methods** that our "validation" dataset is a held-out, fully independent test set, never used in training or tuning. This revision should make the intended roles of the datasets clear to readers from both the genetics and machine learning communities. We hope it can address your concern.

Comment 3: The 'validation' set used in the manuscript cannot be understood as a held-out test set as the authors are using the 'validation' set to optimize the combined RICE-CV and RICE-RV model. Therefore, it is strictly part of the training (and tuning) dataset.

We thank the reviewer for raising this concern. We would like to clarify that the validation dataset is never used for model training or tuning, and serves only as a fully independent, held-out test set.

- All training of PRS models (including GWAS, rare variant association testing, and burden score modeling) is done in the training dataset.
- All hyperparameter selection and ensemble learning are performed in the tuning dataset.

- The validation dataset is used exclusively to evaluate predictive performance (e.g., R^2, AUC, and standardized effect sizes).

The regression analyses on the validation set are not used to improve or optimize model performance, but rather to generate standard performance metrics. This approach is directly parallel to conventional common variant PRS pipelines, where one must regress phenotypes on PRS in the held-out validation data to obtain R^2 or standardized effect sizes.

For RICE specifically:

- When reporting R^2 or AUC for the combined RICE model, we fit the joint model in the tuning set and then apply the estimated weights to the validation set to produce a single prediction score. Thus, performance is evaluated on unseen data.
- When reporting standardized effect sizes for RICE-CV and RICE-RV separately, we fit regressions in the validation dataset. This step is necessary only for interpretability of the component contributions and does not influence the final predictive model.

We have revised the **Methods** section to make this distinction explicit.

Methods (Line 1144, Evaluation of RICE):

"We evaluated RICE using standardized effect sizes and R^2/AUC. For continuous traits, the joint predictive model was modelled on the tuning dataset: $\boldsymbol{Y} = \hat{\theta}_1\widehat{\boldsymbol{Y}}_{CV} + \hat{\theta}_2\widehat{\boldsymbol{Y}}_{RV}$, and for binary traits: $logit(pr(\boldsymbol{Y} = 1)) = \alpha_0 + \hat{\theta}_1\widehat{\boldsymbol{Y}}_{CV} + \hat{\theta}_2\widehat{\boldsymbol{Y}}_{RV}$. Here $\widehat{\boldsymbol{Y}}_{CV}$ and $\widehat{\boldsymbol{Y}}_{RV}$ are the standardized PRSs from RICE-CV and RICE-RV, respectively. Using the estimated $\hat{\theta}_1$ and $\hat{\theta}_2$, we then projected a single combined RICE score onto the validation dataset. R^2 (for continuous traits) and AUC (for binary traits) were calculated in the validation dataset, adjusting for control covariates.

To report standardized effect sizes of RICE-CV and RICE-RV individually, we fit regression models directly in the validation dataset. For continuous traits, we fit linear regression: $\boldsymbol{Y} = \hat{\beta}_1\widehat{\boldsymbol{Y}}_{CV} + \hat{\beta}_2\widehat{\boldsymbol{Y}}_{RV}$. For binary traits, we fit logistic regression $logit(pr(\boldsymbol{Y} = 1)) = \alpha_0 + \hat{\beta}_1\widehat{\boldsymbol{Y}}_{CV} + \hat{\beta}_2\widehat{\boldsymbol{Y}}_{RV} + \boldsymbol{Q}_i\boldsymbol{\alpha}^{\boldsymbol{T}}$, where $\boldsymbol{Q}_i$ represent other covariates, such as age, sex, PCs. These estimated coefficients $\hat{\beta}_1$ and $\hat{\beta}_2$ are the reported standardized effect sizes."

Comment 4: When the ensemble model on common variants (RICE-CV) is trained (termed tuned) this represents extra training of the model. The performance of RICE-CV should therefore be compared to each of the PRS models that are trained on both training and tuning

datasets. For instance, for the simulated UKB dataset, the training data was 98k individuals, and the tuning data was 20k individuals. This means that RICE-CV is trained on 20k more individuals (20% increase in training data) compared to the single models that are only trained on the 98k. RICE-CV should be compared to single models trained on 98+20k individuals.

We thank the reviewer for raising this important point. We would like to clarify that use of the tuning dataset in RICE-CV is consistent with how existing PRS methods are evaluated. Specifically:

- Single-ancestry methods (CT, LDpred2, Lassosum2) each generate multiple PRSs across a grid of hyperparameters using the training dataset, and the optimal PRS is then selected based on performance in the tuning dataset.
- Multi-ancestry methods (CT-SLEB, PROSPER) explicitly fit ensemble regression algorithms in the tuning dataset to combine predictions across ancestries.
- JointPRS-auto is the only method we considered that does not require a tuning dataset, as its parameters are estimated automatically from training data.

In our UKB analyses (e.g., **Figure 4**), CT, LDpred2, and Lassosum2 all relied on the tuning dataset for optimization, while in our All of Us analyses (**Figure 7**), CT-SLEB and PROSPER also performed ensemble learning in the tuning dataset, analogous to RICE-CV. Across both settings, RICE-CV demonstrated consistently robust performance. Thus, while RICE-CV uses the tuning dataset to learn ensemble weights, this does not provide an unfair advantage relative to other methods, since they too rely on the tuning set for optimization or model selection. In all cases, final performance is evaluated exclusively in the independent held-out validation set.

To avoid any confusion, we have expanded the **Existing PRS Methods** section to more clearly describe the role of the tuning dataset in each baseline method and in RICE.

Methods (Line 1171, Existing PRS Methods, CT paragraph):

"The optimal PRS and corresponding p-value threshold are then chosen based on performance in the tuning dataset."

Methods (Line 1183, Existing PRS Methods, Lassosum2 paragraph):

"As in CT, the optimal combination of s and λ_1 was chosen based on predictive performance in the tuning dataset."

Methods (Line 1194, Existing PRS Methods, LDpred2 paragraph):

"The optimal values of h^2 and π were selected using the tuning dataset."

Methods (Line 1204, Existing PRS Methods, CT-SLEB paragraph):

"We further implemented the ensemble learning step on the tuning data to include both sets of PRSs, producing a single prediction for all ancestries."

Methods (Line 1228, Existing PRS Methods, PROSPER paragraph):

"Ensemble regression was performed on the tuning data using SuperLearner."

Comment 5: The authors introduce a new evaluation metric for the predictive performance of their tools due to issues in traditional metrics, which may overlook contributions from rare variants according to the authors. The new metric (regression coefficient of the standardized PRS on the standardized trait) is shown to correspond to the square root of the heritability in a final linear model for one term. However, the authors never show that this relationship holds, when including both the PRS_cv and the PRS_rv terms? We would expect the two terms to be somewhat correlated, making the additive assumption in their predictive performance metric potentially impossible. This could be one of the reasons why the performance gains using the authors' metric are so large. The authors should show the predictive performances of the models using the traditional metrics as well. The authors also never show or argue that their performance metric would hold in the binary case, where even more terms exist in the final model with a logistic link. The binary trait is kept on the original scale, which means the assumption of a standardized phenotype used in their proof of the metric does not hold.

We appreciate your careful reading and the thoughtful questions about our evaluation metric and its interpretation in the joint model of common and rare variant PRSs.

1. **Correlation between PRS_CV and PRS_RV / additivity in the joint model**

Our rare variant association testing (STAARpipeline) is performed conditional on the RICE-CV common variant PRS and standard covariates (**Line 1076**, **Methods**, Null model and association testing). Because the rare variant association are identified after projecting out PRS_CV, the downstream RICE-RV score is expected to be orthogonal to PRS_CV in held-out data. Under this orthogonality, the standardized linear model with both terms, $\boldsymbol{Y} = \beta_1 \widehat{\boldsymbol{PRS}}_{CV} + \beta_2 \widehat{\boldsymbol{PRS}}_{RV} + \boldsymbol{\epsilon}$, implies $var(Y_i) = \beta_1^2 + \beta_2^2 + \sigma^2$. Hence each standardized coefficient retains the

same interpretation as in the single-term case (i.e., its square approximates the trait variance explained by that orthogonal component).

To verify this empirically, we provided a bivariate scatterplot (HDL, UKB Imputed + WES validation set) and report Pearson correlations between PRS_CV and PRS_RV (r = 0.02). Correlations are near zero throughout, consistent with the design of our pipeline.

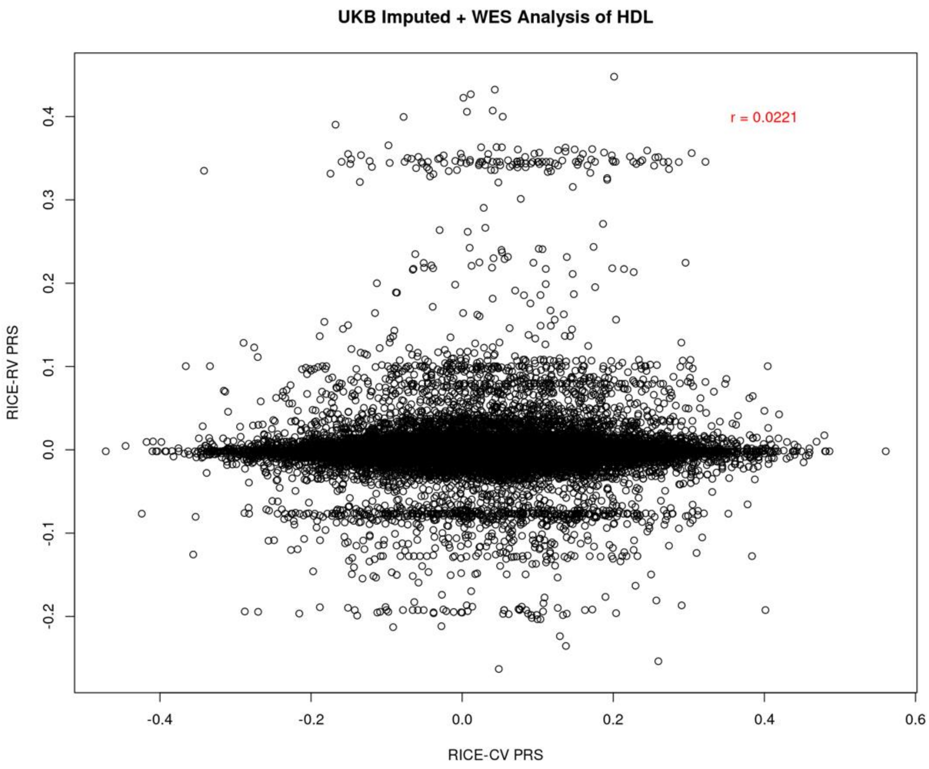

2. Traditional metrics (R^2/AUC) alongside standardized effect sizes

We fully agree that R^2 (continuous traits) and AUC (binary traits) are valuable population-level benchmarks. We have now included these metrics for all real and simulated analyses, derived from a joint model combining RICE-CV and RICE-RV PRSs (trained on the tuning set, evaluated on the validation set). Additional results are provided in the manuscript (detailed below). To assess significance, we used 10,000 bootstrap resamples for confidence intervals on RICE-RV's effect size (testing $\neq 0$) and pairwise R^2/AUC differences (RICE vs. best common variant method). Significance is denoted as ** (95%) or *** (99%) in figures.

We have updated the Results and Methods Section as follows:

Results (Line 179, Evaluation of Prediction Performance):

“RICE’s predictive performance was evaluated using three complementary metrics: (1) standardized effect size, for assessing statistical significance and relative improvements; (2) R^2 (continuous traits) or AUC (binary traits), for absolute predictive accuracy; and (3) trait differences across PRS quantiles, to illustrate potential clinical relevance in the UKB and AoU datasets.”

Results (Line 219, Simulation Study Results):

“RICE-RV also performed strongly, effectively identifying causal rare variant signals and predicting their effects across both EUR and non-EUR populations (**Figure 3 and Supplementary Figure 1**). When added to RICE-CV, RICE-RV significantly improved overall predictive performance. Across ancestries, it increased R^2 by an average of 35.9% compared to using RICE-CV alone.”

Results (Line 274, UKB WES Results):

“In the WES-only configuration, RICE (combining common and rare variants) achieved higher standardized effect sizes and R^2 compared to alternatives for continuous traits (**Supplementary Figure 9d, Supplementary Table 7**). On average, RICE-CV improved R^2 by 6.7% over the best alternative method for each trait-ancestry combination. The largest gains were observed for lipid traits. In EUR individuals, RICE increased R^2 by 10.1% for HDL, 3.2% for height 2.8% for LDL, 11.4% for log(TG) and 3.2% for TC over the best alternative method (**Supplementary Figure 9d)**. RICE also showed marked improvements in non-EUR populations, with R^2 gains of 49.9% for TC in AFR, 41.7% for HDL in AMR, 34.7% for log(TG) in AMR, and 29.7% for LDL in AFR (**Supplementary Figure 9d**). Standardized effect size analyses showed that RICE-RV contributed significantly to most lipid traits ($p < 0.05$) across ancestries, with the exceptions of HDL and log(TG) in AFR, and TC in AMR (**Supplementary Figure 9c**).”

Results (Line 307, UKB Imputed + WES Results):

“Relative to the best alternative method, RICE (CV+RV) improved R^2 for lipid traits in EUR by 4.9% – 8.0% (**Supplementary Figure 11c**). Notable non-EUR gains included 29.8% for TC in AFR, 25.9% for log(TG) in AMR, and 25.5% for HDL in AMR (**Supplementary Figure 11c**). For height, the rare variant component was statistically significant but small (e.g., $\beta =$ 0.039 in EUR and 0.038 in AMR), and corresponding R^2 gains were marginal, leaving RICE’s performance not significantly different from the best alternative for this trait, consistent with a highly polygenic rare variant architecture that yields small effects at the population level.”

Results (Line 356, UKB WGS Results):

"Relative to the best alternative method, RICE (CV+RV) increased R^2 in EUR by 7.5% (height), 8.6% (HDL), 11.2% (LDL), 9.6% (log(TG)), and 10.6% (TC) (**Supplementary Figure 16d**). Notable non-EUR gains included 60.7% for log(TG) in AFR and 29.8% for HDL in AMR."

Results (Line 421, All of Us Results):

"For explained variance, the full RICE model (CV+RV) improved R^2 by an average of 1.7% over the best existing method across trait-ancestry pairs (**Supplementary Figure 23**)."

3. **The relationship between log-OR and variance explained by PRS under logistic regression**

We thank the reviewer for raising this important point. Prior work (Park J. et al., Nature Genetics, 2010) has shown that for binary traits under Hardy-Weinberg equilibrium and an additive polygenic model, the standardized effect size, defined using allele frequency and logistic regression coefficient, corresponds to the square root of the locus's contribution to the genetic variance of the trait. This result provides theoretical justification for interpreting the standardized PRS coefficient in logistic models as an approximate measure of explained variance. We now cite this work and elaborate further in the **Supplementary Note** (**Page 92**) as follows.

"For binary traits modeled via logistic regression, a similar relationship holds. As shown by Park J. et al., Nat. Genet., 2010[7], under Hardy-Weinberg equilibrium and an additive polygenic model, the squared standardized effect size, defined using allele frequency and logistic regression coefficient, approximates the contribution of a locus to the trait's logit-scale variance. This implies that the standardized effect size of a PRS derived from logistic regression coefficients can also be interpreted as approximating the square root of the trait variance explained by the PRS, under similar assumptions."

Comment 6: In the initial association testing for both common and rare variants, the authors used the original phenotype values, which we assume are on the original scale as well. For continuous traits, a more common approach would be to utilize rank-based inverse normal transformed phenotype values or similar. One could theorize that the rare variants are gaining predictive power by being better at capturing the extreme outliers in the original phenotype scale, which would also persist after the residual standardization in the polygenic risk score

estimation. It would be great if the authors could address this and dispute that the predictive performance gain from rare variants arises from phenotype artifacts.

We appreciate your thoughtful point about phenotype scale and the potential influence of extreme values on rare variant signal. For skewed distributions, such as triglycerides, a log-transform was performed before downstream analyses. For GWAS analyses, we conducted association analysis using the original scale (except log(triglycerides)) and adjusted for control covariates. In our PRS construction pipeline, continuous phenotypes are first residualized on covariates and then standardized. Rare variant association testing (STAARpipeline) is conducted conditional on the common variant PRS (RICE-CV) and covariates, so the rare variant component (RICE-RV) is identified after removing variation captured by common variants. This design reduces the likelihood that RICE-RV simply reflects tail behavior aligned with PRS_CV.

Empirically, the patterns we observe are not consistent with tail-driven artifacts. For example, in **Supplementary Figure 12** (UKB Imputed + WES analyses), we plot the mean standardized trait (±SE) across RICE-CV deciles, color-coded by RICE-RV quantiles. If prediction gains from rare variants were predominantly due to a handful of extreme phenotypes on the original scale, we would expect curves that are flat in the middle deciles with abrupt spikes or drops restricted to the 0–10% and 90–100% tails. Instead, for traits where RICE-RV contributes meaningfully, we see graded, approximately linear separation across the middle deciles as well, indicating broad-based signal rather than tail effects.

We did not end up using a rank-based inverse normal transformation (INT) for continuous traits following previous works (CT-SLEB (Zhang H. et al. Nat. Genet., 2023), JointPRS (Xu L., et al., Nat Commun., 2025), and PRS-CSx (Ruan Y. et al., Nat. Genet., 2022)). While INT can temper distributional tails, it changes the measurement scale via a rank mapping that is not uniquely invertible to the original units, which limits clinical interpretability and complicates comparisons across cohorts. Instead, we prioritized analyses on a standardized residual scale. For continuous traits, coefficients from these standardized models have a direct interpretation, change in the outcome (in SD units) per 1-SD increase in PRS, and can be re-expressed in original units by multiplying by the phenotype SD in the validation cohort. Together with the PRS-quantile contrasts shown in the figures, this preserves interpretability while avoiding artifacts from rank-based transformations. We further conducted sensitivity analyses including INT in the response of the next comment.

Comment 7: It is also common practice to utilize a linear mixed model (LMM)-based approach (e.g. BOLT-LMM) or similar (REGENIE) for performing association testing to ensure proper correction for structure. The authors utilize linear models with only 10 PCs to correct for population structure, while the WGS paper of UK Biobank uses 20-40 PCs while also using a LMM-based approach. In Supplementary Figure 7, 8, 15, 16 and 20, it is very clear in the plots that the GWAS summary statistics of the common variants are highly inflated for all traits (as well as many of the WES rare variant association testing), which is highly likely due to improper correction for structure. Despite this observation, the authors state that their Manhattan and QQ plots confirm well-calibrated p-values and controlled inflation factors, which is contradictory. Effect sizes are inflated due to excessive residual confounding, which could further inflate the predictive performance of polygenic risk scores as they may be modelling non-genetic effects as well, potentially benefiting rare variants due to cryptic structure in the dataset.

We appreciate the reviewer's careful attention to calibration and population structure. We agree that mixed-model association is best practice for large, heterogeneous cohorts, and we have updated all UKB and AoU analyses accordingly. In the revised **Supplementary Figures**, the original Supplementary Figure 7, 8, 15, 16, 20 are now **Supplementary Figure 3, 7,** and **21**, respectively.

It is important to note that deviations in QQ plots do not necessarily indicate uncontrolled inflation. As shown by Bulik-Sullivan et al. (2015, PMID: 25642630), such deviations can arise from (1) the polygenic nature of complex traits, particularly in large samples (e.g., height), or (2) unadjusted population stratification. To formally evaluate inflation, we reported two standard metrics: (i) the adjusted genomic inflation factor (λ_{1000}, scaled to a study with 1000 subjects or 1000 cases and 1000 controls for continuous and binary traits respectively), which accounts for large sample sizes, and (ii) the LD-score regression intercept, which helps distinguish polygenicity from confounding. Across all UKB and AoU analyses, λ_{1000} values were < 1.018, and LDSC intercepts were < 1.2, except for height in the EUR WES analysis (1.27; **Supplementary Table 5**), indicating minimal population stratification.

In response to this comment, we additionally performed sensitivity analyses by comparing multiple association models: 1. REGENIE, a whole genome regression model designed to control for population stratification (Mbatchou J., et al., Nat. Genet., 2021, PMID: 34017140), 2. linear regression with varying numbers of PCs, 3. Linear regression with rank-based inverse normal transformation, as suggested in your comment above). We have provided the results in the table below. Inflation metrics were stable across models, with the exception of height in the

EUR WES data. As LDSC was originally developed for common variants across the genome and has not been benchmarked on exome-only analyses, the elevated intercept for height may reflect a limitation of LDSC in this context rather than true stratification. Notably, LDSC intercepts were well controlled for height in UKB imputed (1.15) and WGS (1.15) data.

Trait	Method	Intercept (SE)
BMI	regenie 10 PCs	1.1266 (0.0431)
BMI	plink 10 PCs	1.1225 (0.0409)
BMI	plink 20 PCs	1.1245 (0.0415)
BMI	plink 40 PCs	1.1216 (0.0411)
BMI	plink rank normal 10 PCs	1.1273 (0.0387)
Height	regenie 10 PCs	1.2730 (0.0462)
Height	plink 10 PCs	1.2270 (0.0441)
Height	plink 20 PCs	1.2074 (0.0452)
Height	plink 40 PCs	1.2066 (0.0444)
Height	plink rank normal 10 PCs	1.2273 (0.0442)
HDL	regenie 10 PCs	1.0904 (0.0397)
HDL	plink 10 PCs	1.0564 (0.0351)
HDL	plink 20 PCs	1.0554 (0.0350)
HDL	plink 40 PCs	1.0566 (0.0352)
HDL	plink rank normal 10 PCs	1.0549 (0.0366)
LDL	regenie 10 PCs	1.0434 (0.0413)
LDL	plink 10 PCs	1.0303 (0.0389)
LDL	plink 20 PCs	1.0296 (0.0390)
LDL	plink 40 PCs	1.0293 (0.0389)
LDL	plink rank normal 10 PCs	1.0330 (0.0387)
logTG	regenie 10 PCs	1.1183 (0.0404)
logTG	plink 10 PCs	1.1057 (0.0389)
logTG	plink 20 PCs	1.1063 (0.0388)
logTG	plink 40 PCs	1.1061 (0.0385)
logTG	plink rank normal 10 PCs	1.1100 (0.0401)
TC	regenie 10 PCs	1.0256 (0.0359)
TC	plink 10 PCs	1.0155 (0.0336)
TC	plink 20 PCs	1.0146 (0.0337)
TC	plink 40 PCs	1.0147 (0.0336)
TC	plink rank normal 10 PCs	1.0167 (0.0334)

To ensure robustness, we have followed the suggestions and updated all GWAS analyses in UKB and AoU to use REGENIE (Mbatchou J., et al., Nat. Genet., 2021, PMID: 34017140). We retained 10 PCs, as additional comparisons with 20 or 40 PCs did not meaningfully change LDSC intercepts. Our final model thus used REGENIE with 10 PCs, age, age squared, and sex as covariates. The revised **Results** section now emphasize both λ_{1000} and LDSC intercepts, alongside Manhattan and QQ plots, to show that inflation was well controlled across traits and ancestries.

We have updated the manuscript as follows:

Results (Line 244, UKB WES, Imputed and WGS Results):

"Genomic control metrics indicated well-calibrated analyses, with λ_{1000} ranging between 1 to 1.018. LD score regression intercepts[56] further confirmed minimal population stratification (intercept < 1.2) across all traits, with the exception of height in the EUR WES analysis (LDSC intercept = 1.27, **Supplementary Table 5**). Manhattan and QQ plots for common and rare variant association tests are available for all three analyses: WES (**Supplementary Figures 3-4**), Imputed + WES (**Supplementary Figures 5-6**), and WGS (**Supplementary Figures 7-8**)."

Results (Line 401, All of Us Results):

"The genomic inflation factor was well controlled, with λ_{1000} ranging from 1 to 1.003 (**Supplementary Table 5**), with LD score regression intercepts[56] confirming minimal population stratification across traits and ancestries. Manhattan and QQ plots for common and rare variant association tests are provided in **Supplementary Figures 21-22**."

Methods (Line 961, Phenotype and PRS Standardization):

"Association testing of common variants was performed with REGENIE[84]."

Comment 8: It is unclear how the principal components (PCs) are obtained in the different datasets and their subsets, as the authors adjust for the top 10 PCs in most steps of their analysis pipeline. We therefore assume that the authors are using the pre-computed PCs from the UK Biobank and All-of-Us. However, this means that the validation set (test set) has been used in the estimation of the PCs, such that it is not an unseen set, thus the test set evaluation will be biased. A way to avoid this would be to perform PCA in the training data to obtain their PCs and only afterward project the test individuals onto the PC space produced by the training data.

Thank you for your comment. We appreciate the reviewer's point about potential leakage when using cohort-provided principal PCs. To address this, we first clarify how the PCs were obtained: For UKB, we used the pre-computed top 10 PCs provided by the UK Biobank, which are derived from the full dataset. For AoU, we computed PCs based on pre-computed PCA loadings calculated from the samples of the 1000 Genomes Project reference panel, ensuring independence from the AoU data itself. We have added additional descriptions into the **Methods** section to clarify:

Methods (Line 1296, UKB WES, Imputed, and WGS Analysis):

"PCs used were the pre-computed ones provided by UKB (Field 22009)."

Methods (Line 1331, AoU Analysis):

"For AoU, we computed PCs based on pre-computed PCA loadings calculated from the samples of the 1000G reference panel."

To evaluate potential bias in UKB, we conducted a sensitivity analysis using the UKB imputed genotype data. Specifically, we compared GWAS results and clumping + thresholding (C+T) PRS performance for the six continuous traits when using (i) the UKB-provided 10 PCs versus (ii) 10 PCs computed separately for the training data, tuning and validation sets). Below are scatterplots comparing the observed -log10 p-values from REGENIE association testing under these two approaches. The figure demonstrates strong agreement between the results, with near-perfect correlation particularly for smaller p-values (i.e., stronger signals).

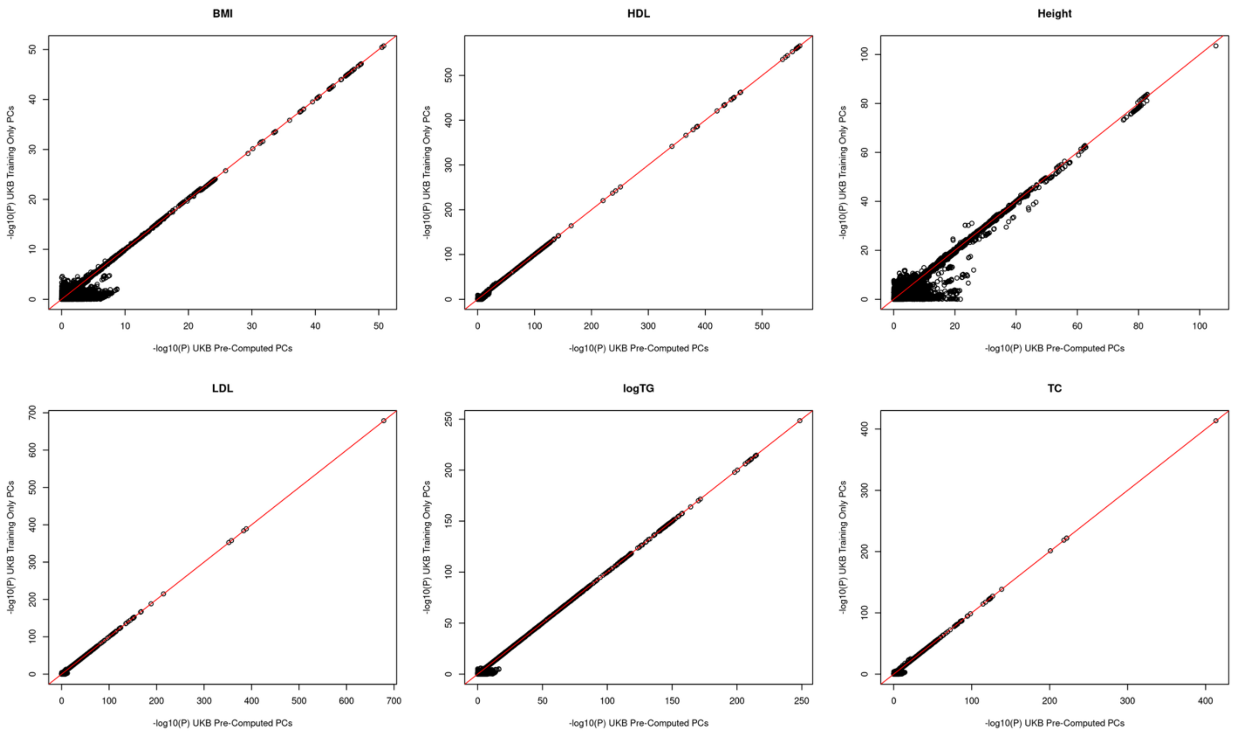

We further assessed downstream impacts by performing C+T PRS construction and evaluation, where PCs were computed separately within each subset (training, tuning, and validation). Below, we plot the estimated beta coefficients (standardized PRS per net SD) for the C+T results using pre-computed PCs compared against those using subset-specific PCs.

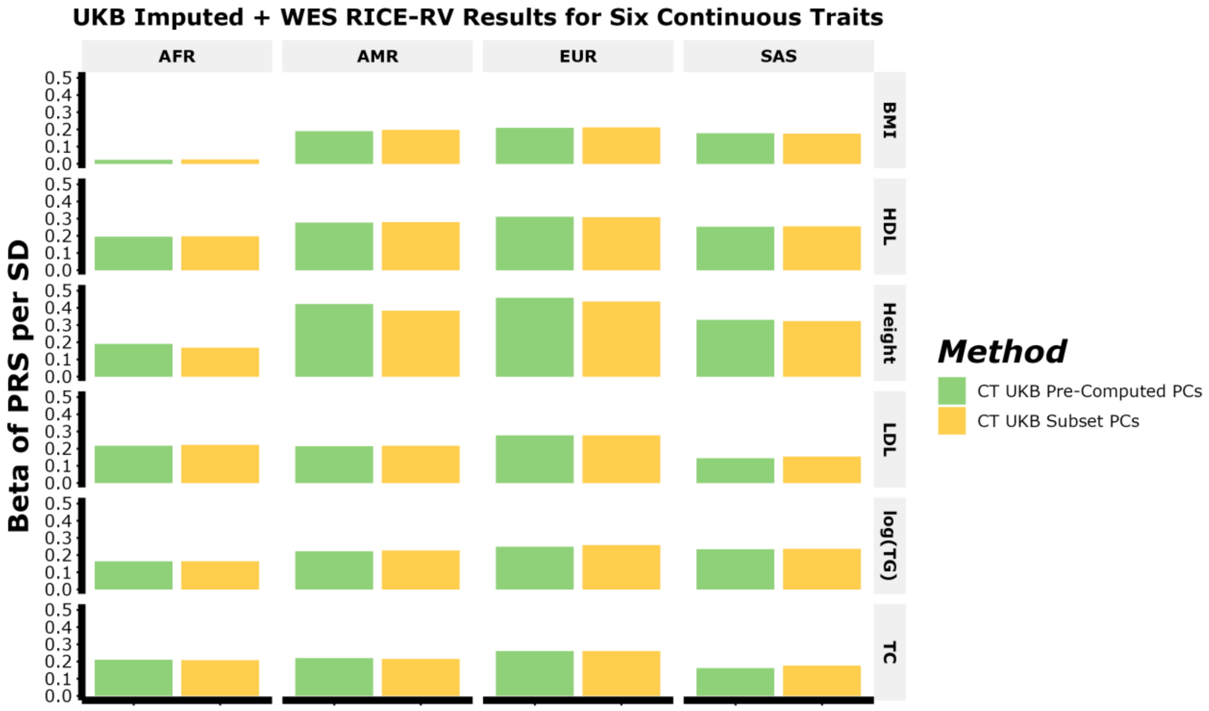

Similar to the p-value comparisons, there is excellent agreement between the two approaches, indicating minimal bias from using pre-computed PCs. Therefore, our updated UKB results continue to use the pre-computed PCs for consistency with standard practices in the field. As noted, the AoU analyses use 1000 Genomes-projected PCs, which are independent and thus unaffected by this concern.

Comment 9: The genetically-inferred ancestry labels in the UK Biobank were estimated based on a random forest classifier trained on the top 5 PCs estimated from the 1000 Genomes Project (1KGP). However, here you use the 3202 individuals in 1KGP, which also includes all related individuals in 1KGP and not the 2504 “unrelated” individuals from the phase 3 release. This would create a bias in the estimated PCs as it will somewhat capture relatedness and not population structure. The labels here are the super populations in 1KGP, but there are plenty of individuals with mixed ancestry, which will be completely disregarded using your classification approach as well. How was the ancestry labelling of individuals defined? Using the argmax or some probability threshold?

Thank you for your insightful comment. We apologize for the error in our initial submission regarding the training data for the random forest classifier. The classifier was indeed trained on the 2,504 unrelated individuals from the 1000 Genomes Project phase 3 release, rather than the

expanded set of 3,202 samples that includes related individuals. Using the unrelated subset ensures that the principal components primarily capture population structure without bias introduced by relatedness. We have updated the manuscript to reflect this correction.

Methods (Line 1276, UKB WES, Imputed, and WGS Analysis):

"Genetically inferred ancestry groups were defined using the 1000 Genomes Project Phase 3 (1000G) dataset[55], which includes 2,504 individuals across five ancestry groups: 661 AFR, 347 AMR, 504 EAS, 503 EUR, and 489 SAS."

Regarding individuals with mixed ancestry, our classification approach assigns labels based on the 1KGP super populations, which may result in admixed individuals being assigned to the most similar super population, potentially overlooking finer-scale admixture. Ancestry labeling was defined using the argmax method, i.e., selecting the ancestry group with the highest predicted probability from the random forest classifier. We did not impose an additional probability threshold for assignment, as the majority of assignments in the UK Biobank cohort exhibited high confidence (e.g., probabilities > 0.9 for the selected group). We recognize this as a limitation for highly admixed individuals, though their proportion in UK Biobank is relatively low, minimizing overall impact on our analyses.

Comment 10: It is not clear how the other methods are evaluated, if it is the same approach as for PRS_cv, where the single standardized PRS is used in a linear model with the standardized phenotype based on their new performance metric.

Thank you for this comment. We confirm that PRSs from all existing methods were evaluated using the identical approach as for RICE-CV and RICE-RV. Specifically, each PRS was standardized and incorporated into a linear model with the standardized phenotype to derive the performance metric (standardized effect size, R^2/AUC), ensuring consistent and comparable assessments across all methods. We have updated the **Data Processing and Standardization** section to explicitly emphasize this uniform evaluation framework.

Methods (Line 979, Data Processing and Standardization):

"All PRS methods evaluated in the manuscript followed an identical evaluation procedure."

Comment 11: Due to using multiple external computation tools and fitting multiple prediction models (ensemble) as well, it would be nice to have an estimate or overview of the full computational runtime for a given trait (both in the continuous and the binary case).

Thank you for this valuable comment regarding the computational cost of RICE. We agree that reporting these details enhances the framework's transparency and usability. We have recorded the computational time for each component of RICE based on the UKB Imputed + WES analysis, as outlined in the UKB WES, Imputed, and WGS Analysis section (**Results**, **Line 343**). These timings are now available in **Supplementary Table 12**, which details the duration for tasks such as rare variant association testing, ensemble learning, and other steps across traits. A copy of this table is provided below for reference. We also included a copy of this table below:

Supplementary Table 12: Timing of each step of RICE for the UKB Imputed + WES analysis in compute hours

Trait[1]	GWAS	CT	LDpred2/Lassosum2	RICE-CV	STAARpipeline	RICE-RV	RICE	Total
BMI	8.55(5.37%)	3.06(1.92%)	30.03(18.86%)	0.16(0.10%)	116.64(73.25%)	0.40(0.25%)	0.40(0.25%)	159.24
Height	7.52(5.65%)	3.04(2.28%)	27.57(20.69%)	0.17(0.13%)	94.11(70.65%)	0.43(0.32%)	0.38(0.28%)	133.21
LDL	12.64(9.02%)	2.79(1.99%)	27.16(19.38%)	0.20(0.14%)	95.59(68.21%)	0.78(0.56%)	0.98(0.70%)	140.14
HDL	9.85(6.88%)	2.96(2.07%)	28.39(19.83%)	0.18(0.13%)	99.83(69.72%)	0.92(0.64%)	1.06(0.74%)	143.19
logTG	8.91(6.93%)	2.80(2.18%)	27.27(21.20%)	0.20(0.16%)	88.09(68.51%)	0.90(0.70%)	0.42(0.32%)	128.59
TC	7.04(4.53%)	2.69(1.73%)	27.79(17.85%)	0.16(0.10%)	117.13(75.25%)	0.30(0.19%)	0.53(0.34%)	155.65
Asthma	12.46(6.92%)	3.11(1.73%)	33.13(18.39%)	1.40(0.78%)	127.44(70.74%)	1.19(0.66%)	1.41(0.79%)	180.15
Breast	10.78(7.31%)	3.04(2.06%)	32.77(22.20%)	1.60(1.09%)	94.60(64.11%)	1.84(1.24%)	2.95(2.00%)	147.57
CAD	11.47(6.03%)	3.12(1.64%)	19.13(10.07%)	1.22(0.64%)	150.79(79.35%)	2.17(1.14%)	2.14(1.13%)	190.04
Prostate	11.68(9.52%)	3.11(2.53%)	33.34(27.17%)	0.78(0.64%)	69.68(56.79%)	1.32(1.08%)	2.79(2.28%)	122.70
T2D	12.72(7.65%)	3.12(1.87%)	25.81(15.52%)	1.59(0.95%)	120.21(72.28%)	1.17(0.70%)	1.69(1.02%)	166.31

[1] Eleven unique traits are included in the analyses. Six continuous traits and five binary traits were analyzed in the UKB Imputed + WES analysis.

We have incorporated this change in the manuscript:

Results (Line 343):

"We also assessed the computational efficiency of the RICE framework (**Supplementary Table 12**). Ensemble learning for RICE-CV and RICE-RV required on average 1.73 compute hours per trait (~1.1% of total compute time). Rare variant association testing accounted for the largest compute share (106.7 hours, 381 parallel jobs, 68.6%), followed by LDpred2/Lassosum2 modeling (28.4 hours, 18.9%)."

Comment 12: How different is your approach that performs both LASSO and Ridge regression in the ensemble step (using the "SuperLearner" package) from an elastic net that optimizes both the L1 and L2 norm jointly? Ridge logistic regression is not used in your pipeline for binary traits due to the unavailability in the "SuperLearner" package, however, that should be available (alongside elastic net) in other popular machine learning packages. It is already a part of the "glmnet" package that is a requirement for your pipeline as an example.

Thank you for this insightful comment. Our original ensemble approach, implemented via the Super Learner package, differs from elastic net regression in that it combines predictions from separately trained LASSO and ridge regression models (as base learners) through a meta-

learner, potentially capturing complementary strengths of the individual regularizations. In contrast, elastic net jointly optimizes the L1 and L2 penalties within a single model. We also want to highlight that the ensemble step is flexible, users are not restricted to LASSO and ridge regression as base learners. If needed, methods like elastic net or non-linear approaches such as neural networks and random forests can be incorporated. We selected LASSO and ridge primarily for computational efficiency and to maintain linear forms, facilitating deposition of the final PRSs into PGS Catalogs and ease of sharing with the research community.

We initially selected SuperLearner due to its demonstrated success in prior methods like CT-SLEB (Zhang H. et al., Nat. Genet. 2023) and PROSPER (Zhang J. et al., Nat. Commun., 2024). However, as you noted, it lacks built-in support for ridge logistic regression for binary traits and offers limited overall flexibility. To address these limitations, we have transitioned away from SuperLearner and implemented a custom ensemble regression algorithm that incorporates both LASSO and ridge regression for continuous and binary traits. This new implementation leverages the glmnet package for the base models, enabling ridge logistic regression for binary outcomes. A detailed description of the updated ensemble algorithm for common variant PRS is provided in the **Ensemble Learning** section of the **Methods** as follows:

Methods (Line 1030):

"Similarly for binary traits, the ensemble model combines predictions from LASSO and ridge regression using a generalized linear regression model. For LASSO logistic regression, we minimize the objective function:

$$\min_{\boldsymbol{w}^{(1)}} -\frac{1}{n}\sum_{i=1}^{n}[Y_i \log(p_i) + (1-Y_i)\log(1-p_i)]^2 + \lambda_1 \sum_{j=1}^{J} |w_j^{(1)}| ,$$

where $p_i = \sigma(\sum_{j=1}^{J} w_j^{(1)} PRS_{ij})$ is the predicted probability for individual i, with $\sigma(x) = exp(x)/\{1+\exp(x)\}$ being the logistic function and Y_i is the binary phenotype (0 or 1) for individual i.

For ridge logistic regression, we minimize the objective function:

$$\min_{\boldsymbol{w}^{(2)}} -\frac{1}{n}\sum_{i=1}^{n}[Y_i \log(p_i) + (1-Y_i)\log(1-p_i)]^2 + \lambda_2 \sum_{j=1}^{J} (w_j^{(2)})^2 ,$$

where $p_i = \sigma\left(\sum_{j=1}^{J} w_j^{(2)} PRS_{ij}\right)$. Optimal regularization parameters λ_1 and λ_2 are selected by minimizing the cross-validated AUC (default fold = 10). Using the optimal weights for each base learner, the predicted log odds are generated for LASSO and ridge ($\widehat{\boldsymbol{Y}}^{(1)}$ and $\widehat{\boldsymbol{Y}}^{(2)}$ respectively). The ensemble learning algorithm estimates the optimal weights of the predictions, $\boldsymbol{\alpha} = (\alpha_1, \alpha_2)^T$, by minimizing the objective function:

$$\min_{\boldsymbol{\alpha}} -\frac{1}{n}\sum_{i=1}^{n}[Y_i \log(p_i) + (1 - Y_i)\log(1 - p_i)]^2,$$

where $p_i = \sigma\left(\sum_{j=1}^{2} \alpha_j \hat{Y}_i^{(j)}\right)$. The final prediction of the ensemble learning algorithm is then: $\widehat{\boldsymbol{Y}}_{CV} = \alpha_1 \widehat{\boldsymbol{Y}}^{(1)} + \alpha_2 \widehat{\boldsymbol{Y}}^{(2)} = \alpha_1 \boldsymbol{w}^{(1)} \boldsymbol{PRS_{CV}} + \alpha_2 \boldsymbol{w}^{(2)} \boldsymbol{PRS_{CV}}$, where $\boldsymbol{PRS_{CV}} = [\boldsymbol{PRS_1}, \boldsymbol{PRS_2}, \dots, \boldsymbol{PRS_J}]$."

Comment 13: The predictive performance for binary traits is usually reported on the liability scale using some pseudo R^2-measures to account for the case-control ratio between data and population prevalence. Pseudo R^2's are by no means a perfect metric, but how are the case-control ratio and population prevalence mitigated with your proposed metric?

Thank you for your comment. We appreciate the point regarding the common practice of reporting predictive performance for binary traits converted to the liability scale using pseudo-R^2 measures. This conversion adjusts for the study's case-control ascertainment ratio relative to the population prevalence, enabling more comparable estimates across datasets and approximating the proportion of variance explained on an underlying continuous liability scale. However, estimating population prevalence can introduce uncertainties, particularly for non-European ancestries, which may complicate liability-scale conversions.

In our study, the primary metric for binary traits is the coefficient from a logistic regression model of the original binary outcome on the standardized PRS, adjusting for covariates (top 10 PCs, sex, age, and age squared). This the log(odds ratio) per SD increase in PRS and serves as a direct, interpretable measure of association. β estimate from logistic regression is generally unbiased for the population log(OR) under rare disease approximation (Prentice R.L. and Pyke R., Biometrika, 1972), as oversampling of cases primarily affects the intercept without biasing the slope for the PRS covariate. Thus, our metric inherently accounts for the case-control ratio

without requiring explicit post-hoc adjustments for prevalence, providing a fair comparison even across ancestries with differing prevalences.

We acknowledge that in highly imbalanced case-control settings, log-odds ratio estimates for single variants may be inflated due to limited cases carrying alternative alleles, potentially violating asymptotic assumptions (Zhou W., et al. Nat. Genet. 2019). However, since our focus is on PRS, an aggregate of many variants, the statistical inference from logistic regression remains valid. Additionally, our significance testing relies on bootstrapping, which does not depend on asymptotic assumptions.

To provide a more comprehensive evaluation, we have added the AUC as another metric for binary traits. AUC measure's discriminative ability is robust to class imbalance and complements β by emphasizing classification performance.

Reviewer #4 (Remarks to the Author):

I co-reviewed this manuscript with one of the reviewers who provided the listed reports. This is part of the Nature Communications initiative to facilitate training in peer review and to provide appropriate recognition for Early Career Researchers who co-review manuscripts.

We sincerely thank you for co-reviewing the manuscript. We appreciate the collaborative effort in providing feedback to improve our work. Thank you so much!

Author Response Letter

We are grateful for the reviewers' very helpful and thoughtful comments. We have carefully incorporated these suggestions into the revised manuscript, which we believe has significantly strengthened the work. Our detailed, point-by-point responses to each of the reviewer's comments are provided below:

Reviewer #1 (Remarks to the Author):

This revision has enhanced the manuscript, and I commend the authors for their hard work. I have a few remaining comments and suggestions as detailed below.

We appreciate your positive and constructive feedback, which has been very instrumental in further refining the clarity and impact of this work.

Q1. I remain skeptical about using "Beta per SD of PRS" as a metric for rare variant PRS. As the authors point out, it is effectively the square root of variance explained and therefore does not really carry additional information beyond R2. In the rare variant PRS context, the metric is also not easily interpretable because the SD of PRS depends on the frequency of the variant. A more interpretable metric might be to report the effect per additional copy of the risk variant. My concern is that this choice of metric could overstate the contribution of rare variant PRS, which would look much smaller if beta is squared and expressed as variance explained. That said, I do not think this issue should prevent publication.

We sincerely thank you for these thoughtful considerations and for highlighting the statistical relationship between the standardized coefficient and variance explained. We fully appreciate your perspective that "Beta per SD" is mathematically related to the square root of R^2 and, as such, can appear visually larger than the proportion of variance explained would suggest. While we agree that R^2 is the superior metric for quantifying population-level variability, we have balanced this with the need for clinical interpretability. We respectfully propose retaining "Beta per SD" as a complementary primary metric alongside R^2 for the following reasons:

1. **Clinical Intuition**: In our experience, interpreting the magnitude of phenotypic shift is often more intuitive for clinical and translational audiences than variance explained. "Beta per SD" answers a practical question: "If a patient moves up by one standard deviation in the

polygenic risk distribution, how much does their predicted phenotype change?" We believe this offers a valuable perspective that complements the aggregate summary provided by R^2.

2. **Consistency with Binary Outcomes**: For binary traits, the log-odds ratio (or odds ratio) per SD is the standard in genetic epidemiology for evaluating risk stratification (e.g., for breast cancer or type 2 diabetes). It's worth noting that for binary traits, there is also a direct one-to-one mathematical relationship between the log-odds ratio per SD (σ) and the AUC, given by $AUC = \Phi(\sigma/\sqrt{2})$, where $\Phi(\cdot)$ is the cumulative distribution function of the standard normal distribution (we have provided the proof for this relationship at the end of this response). This is analogous to the relationship between Beta per SD and $\sqrt{R^2}$ for continuous traits. Despiste this mathematical link, the field standardly report both: AUC to describe discrimination and log-odds ratio per SD to describe the magnitude of risk stratification. By using "Beta per SD" for continuous traits, we maintain a consistent reporting framework across all 11 phenotypes in the manuscript, allowing readers to compare the relative strength of stratification between continuous and binary traits on a comparable scale.

3. **Aggregate Nature of PRS**: Regarding your helpful suggestion to report the "effect per additional copy", while this is ideal for single-variant associations (e.g., a *BRCA1* carrier), we found this challenging to implement for the aggregate RICE-RV score. The RICE-RV framework identifies significant rare variant sets across the genome (ranging from dozens to hundreds depending on the trait) and models them jointly. Because the final score is a weighted combination of these various burden scores, each encompassing numerous variants. As such, there is no single "risk variant copy" to reference for the aggregate RICE-RV score, making the "per SD" standardization the most viable approach for comparing distributions.

To address your concern about overstatement: We fully acknowledge your point that this metric can visually appear larger than the corresponding R^2. To ensure we do not mislead readers, we have revised the Discussion explicitly acknowledging this limitation:

Discussion (Line 481)

"We emphasize that the reporting of standardized effect sizes is intended to provide a measure of the expected phenotypic shift for individual carriers, an interpretation that is often more

intuitive in clinical settings than variance explained. However, it is important to acknowledge the mathematical coupling between these metrics; for a standardized PRS, the Beta per SD is equivalent to the square root of the heritability explained by the score. Consequently, for rare variants, these effect sizes should be interpreted with caution regarding population-level impact. A substantial per-SD effect can be observed even when the overall R^2 is modest, as the low frequency nature of rare variants inherently limits the total variance they can explain across the entire population."

We believe this addition balances the need for interpretable effect sizes with a transparent acknowledgment of the statistical properties that you highlighted.

Proof of the relationship between AUC and log-odds ratio per SD of PRS

Let D denote the disease status ($D = 1$ for cases, $D = 0$ for controls). In a standard polygenic model, the log-odds of disease is determined by sum of individual genetic effects: $\text{logit}(P) = \alpha + \sum_k \beta_k G_k$. We define u as the total genetic liability on the logit scale: $u = \sum_k \beta_k G_k$. By this definition probability of disease given u is:

$$P(D = 1|u) = \frac{\exp(\alpha + u)}{1 + \exp(\alpha + u)}.$$

We assume u follows a normal distribution in the control population: $(u|D = 0) \sim N(0, \sigma^2)$, where σ^2 is the variance of the PRS on the logit scale. For standardized PRS (PRS_{std}) with mean 0 and variance 1, we have $PRS_{std} = u/\sigma$. Substituting this into the logit function:

$$\text{logit}\big(P(D = 1)\big) = \alpha + \sigma PRS_{std},$$

Thus, the log-odds ratio per SD of PRS is equal to σ.

To find the distribution of genetic effects in cases $(u|D = 1)$, we apply the Bayes' theorem:

$$f(u|D = 1) = \frac{P(D = 1|u) f(u|D = 0)}{P(D = 1)}$$

Under rare disease assumption, the logistic likelihood can be approximated as $P(D = 1|u) \propto e^u$. Substituting the normal prior for controls ($f(u|D = 0) \propto e^{-\frac{u^2}{2\sigma^2}}$), the posterior distribution becomes:

$$f(u|D = 1) \propto e^u \cdot e^{-\frac{u^2}{2\sigma^2}} = e^{u - \frac{u^2}{2\sigma^2}}$$

By completing the square in the exponent:

$$u - \frac{u^2}{2\sigma^2} = -\frac{1}{2\sigma^2}(u^2 - 2\sigma^2 u) = -\frac{1}{2\sigma^2}((u - \sigma^2)^2 - (\sigma^2)^2)$$

This simplifies to a kernel of a normal distribution with mean σ^2 and variance σ^2, that is:

$(u|D = 1) \sim N(\sigma^2, \sigma^2)$. The AUC corresponds to the probability that a randomly selected case has a higher score than a randomly selected control ($u_{case} > u_{control}$). The difference variable $\Delta = u_{case} - u_{control}$ follows: $\Delta \sim N(\sigma^2, 2\sigma^2)$. The AUC is thus the probability that $\Delta > 0$:

$$AUC = P(\Delta > 0) = \Phi\left(\frac{\sigma}{\sqrt{2}}\right),$$

where $\Phi(\cdot)$ is the cumulative distribution function of the standard normal distribution. This demonstrates that the AUC is a direct function of σ (the log-odds ratio per SD) under plausible assumptions, confirming the mathematical mapping between the two metrics.

Q2. From Figure 1 and the accompanying text (line 150), it appears that another linear combination between common and rare PRS is learned during evaluation, which could imply overfitting. However, the Methods section seems to suggest that the combination weights were trained using the tuning set. This should be clarified. The authors might consider adjusting Figure 1 to avoid giving the impression that additional model fitting was performed on evaluation data.

We sincerely thank you for raising this point and allowing us to clarify the evaluation procedure. We agree that the distinction between model training and metric estimation was not sufficiently clear in Figure 1.

We hope to clarify that there is a slight procedural difference how we estimate the overall R^2 (for the combined RICE model) versus the standardized effect sizes (Beta per SD) for RICE-CV and RICE-RV. Neither of which involves overfitting:

1. **Evaluation of R^2 (combined RICE model):** For the overall performance of RICE, we treat the combination as a pre-trained model. We fit the linear combination weights for RICE-CV and RICE-RV exclusively in the tuning dataset. These estimated weights ($\hat{\theta}_1, \hat{\theta}_2$) are then fixed and applied to the validation dataset to compute a single predictive score ($S =$

$\hat{\theta}_1 \, PRS_{CV} \; + \; \hat{\theta}_2 PRS_{RV}$). The reported R^2 is the squared correlation between this fixed score and the phenotype in the validation data. No weights are learned in the validation step.

2. **Evaluation of Beta per SD (for RICE-CV and RICE-RV):** To report the specific contributions of common vs. rare variants, we report the coefficients for RICE-CV and RICE-RV separately. To obtain these, we fit a regression model directly on the validation dataset: $Y \sim \beta_1 PRS_{CV} + \beta_2 PRS_{RV}$. This step is solely for reporting metrics (estimating the effect size in an independent sample), which is analogous to the standard practice of running a regression ($Y \sim PRS$) in a validation cohort to estimate effect sizes for traditional common variant PRSs.

Independence of Components: We want to further highlight that RICE-CV and RICE-RV are designed to be statistically independent, ensuring that the joint regression in the validation set is stable. As detailed in the Methods (Line 1061), our rare variant association testing (using STAARpipeline) is performed conditional on the RICE-CV score. Because the rare variant associations are identified after projecting out the common variant signal, the downstream RICE-RV score is expected to be orthogonal to RICE-CV. Empirically, we verified this in the UKB Imputed + WES validation set, observing a Pearson correlation of only $r = 0.02$ (Figure attached). This confirms that the coefficients estimated in the validation set reflect distinct, additive genetic signals and are not artifacts of collinearity.

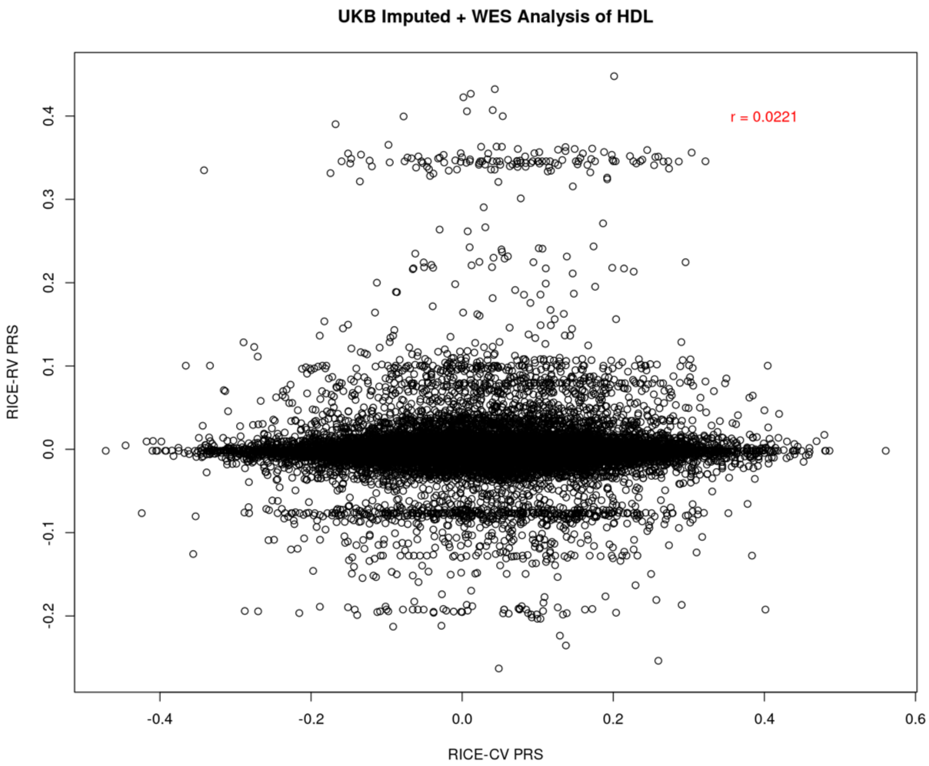

To address your concern and prevent the impression of overfitting, we have revised the Figure 1 Legend and Method Section as follows:

Figure 1 Legend (Line 630):

"c) Final evaluation: The pre-trained RICE-CV and RICE-RV models (with combination weights fixed from the tuning step) are applied to the validation set to evaluate overall predictive performance (R^2 or AUC). Additionally, a regression model is used solely to estimate individual standardized effect sizes (Beta per SD) of the components, adjusting for covariates such as age, sex, and principal components."

Methods (Line 1155, Section Evaluation of RICE)

"We evaluated RICE using standardized effect sizes and R^2/AUC. First, to establish the combined RICE score, we model the joint prediction on the tuning dataset. For continuous traits, we fit: $\boldsymbol{Y} = \hat{\theta}_1 \widehat{\boldsymbol{Y}}_{CV} + \hat{\theta}_2 \widehat{\boldsymbol{Y}}_{RV}$, and for binary traits: $\text{logit}(P(\boldsymbol{Y} = 1)) = \alpha_0 + \hat{\theta}_1 \widehat{\boldsymbol{Y}}_{CV} + \hat{\theta}_2 \widehat{\boldsymbol{Y}}_{RV}$. Here $\widehat{\boldsymbol{Y}}_{CV}$ and $\widehat{\boldsymbol{Y}}_{RV}$ are the standardized RICE-CV and RICE-RV PRSs, respectively. Crucially, the weights $\hat{\theta}_1$ and $\hat{\theta}_2$ are estimated exclusively in the tuning dataset and fixed. These fixed weights are

then applied to the validation dataset to compute the final score. R^2 (for continuous traits) and AUC (for binary traits) are calculated in the validation dataset, adjusting for covariates.

Second, to report standardized effect sizes of RICE-CV and RICE-RV, we fit regression models directly in the validation dataset. This step is intended solely to quantify component effect sizes in an independent sample. For continuous traits, we fit linear regression: $\boldsymbol{Y} = \hat{\beta}_1 \widehat{\boldsymbol{Y}}_{CV} + \hat{\beta}_2 \widehat{\boldsymbol{Y}}_{RV}$. For binary traits, we fit logistic regression $\mathrm{logit}(P(\boldsymbol{Y} = 1)) = \alpha_0 + \hat{\beta}_1 \widehat{\boldsymbol{Y}}_{CV} + \hat{\beta}_2 \widehat{\boldsymbol{Y}}_{RV} + \boldsymbol{Q}_i \boldsymbol{\alpha}^{\boldsymbol{T}}$, where $\boldsymbol{Q}_i$ represent other covariates, such as age, sex, PCs. These estimated coefficients $\hat{\beta}_1$ and $\hat{\beta}_2$ are the reported standardized effect sizes."

Q3. The STAAR framework uses kernel-based methods (e.g., SKAT) to detect variant set associations, while RICE aggregates rare variants into burden scores, which may fail to capture nonlinear effects that contribute to association signals. It would be helpful to discuss this limitation explicitly.

Thank you for this insightful suggestion. We agree that while the full STAAR framework includes kernel-based methods (e.g., SKAT [47]) to detect non-linear associations, our current RICE framework utilizes burden-based testing (specifically STAAR-B) to align with the additive nature of the PRS model. This design choice ensures consistency between identification and prediction but effectively trades off the ability to capture non-linear or epistatic effects.

As requested, we have revised the Discussion to explicitly acknowledge this limitation. We clarify that the linear aggregation may miss complex signals and suggest that future extensions could explore non-linear integration methods. To improve clarity and address this point alongside other limitations, we have also merged this point into a streamlined limitations paragraph. We also updated the Methods section to clarify that we specifically prioritized STAAR-B p-values for identification to ensure alignment with the downstream additive model.

Discussion (Line 564)

"Furthermore, while the STAAR framework can detect non-linear signals (e.g., via SKAT), RICE prioritizes STAAR-Burden (STAAR-B) to align with its additive prediction model; this ensures consistency but may fail to capture complex epistatic interactions, suggesting a need for future non-linear integration methods."

Methods (Line 1101, Section Null Model and Association Testing)

"Within RICE-RV, we specifically restrict our identification to the p-values derived from the annotation-weighted burden tests, referred to as STAAR-B(1,1) p-values in STAARpipeline to ensure that the identified rare variant signals align with the additive burden-based architecture used in the downstream RICE prediction model."

Q4. The simulation settings do not appear realistic. For example, assuming that common and rare variants on chromosome 22 explain 5% and 1.25% heritability, respectively, is overly optimistic. All simulation scenarios focus on unrealistically strong genetic signals and do not examine performance in settings where GWAS/EWAS power is limited. In reality, for many traits and diseases, genome-wide rare variants collectively explain <1% of heritability.

We thank you for this critical assessment of our simulation parameters. We agree that the initial rare variant heritability ($h^2_{RV} = 1.25\%$) was optimistic relative to the common variant heritability ($h^2_{CV} = 5\%$), yielding a ratio (1:4) that is higher than typically observed for complex traits.

To address this, we have completely re-conducted our simulation study (100 independent replicates across all settings) using updated, empirically grounded parameters.

1. **Justification for Chromosome 22 Design**: First, regarding the choice of limiting simulations to Chromosome 22: conducting 100 replicates of whole-genome simulations with both genotype data and rare variant burden testing is computationally prohibitive. Consequently, we must concentrate the "signal density" of a polygenic trait into a single chromosome to evaluate the statistical properties of the method (e.g., overfitting, weight estimation) within a manageable sample size. While $h^2_{CV} = 5\%$ is not intended as a realistic estimate for Chromosome 22 specifically, we chose this value to ensure a sufficiently high signal-to-noise ratio on a single chromosome. This operational choice provides the signal strength necessary to rigorously evaluate model mechanics, such as weight estimation and the prevention of overfitting, within a computationally manageable framework.

2. **Adopting Realistic Ratios**: However, we fully agree that the relative contribution of rare variants was too high. To select a realistic parameter, we followed the recent burden heritability estimates from Weiner D. et al., Nature, 2023. Their analysis of 22 complex traits (Figure 2c)

indicated that the median ratio of burden heritability to common variant heritability is approximately 1:12. Accordingly, we updated our simulation settings to reflect this ratio:

- Common Variant h^2_{CV}: Retained at 5% (to maintain baseline signal).
- Rare Variant h^2_{RV}: Reduced to 0.42% (4.17×10^{-3}), reflecting the realistic 1:12 ratio.

As expected, the absolute predictive performance in these new simulations is lower than in the original draft, but the relative performance trends remain consistent. Importantly, the results under these stricter settings are now much closer to the empirical results we observed in the UK Biobank and All of Us real data analyses. We have updated the Simulation Results and Methods section and Figure 3, Supplementary Figure 1-2 to reflect these new, rigorous settings.

Results (Line 212, Simulation Study Results)

"To evaluate RICE's performance under realistic genetic architectures, we calibrated the relative contribution of rare variants by adopting a 1:12 ratio of rare variant burden heritability to common variant heritability, consistent with recent median estimates based on 22 complex traits [37]. For our single-chromosome simulation framework, we set the common variant heritability (h^2_{CV}) at 5% to ensure sufficient signal strength on chromosome 22 for evaluating model mechanics, such as weight estimation and prevention of overfitting, within a manageable computational burden. Accordingly, the rare variant heritability (h^2_{RV}) was set at 0.42%."

Methods (Line 1258, Simulation Study)

"The heritability was kept constant across simulations, with the common variant heritability (h^2_{CV}) to 5% to ensure sufficient statistical power to evaluate model mechanics within a single-chromosomal simulation framework. To reflect realistic genetic architectures, we calibrated the rare variant heritability based on recent empirical estimates of the ratio between burden and common variant heritability (median around 1:12[37]). Accordingly, we set rare variant heritability $h^2_{RV} = 0.42\%$."

Q5. In Figure 3, the "Beta per SD" for rare variant PRS reaches ~0.2, implying ~4% variance explained, whereas the simulated variance explained by rare variants is only 1.25%. Please clarify this discrepancy. I'm not sure if I missed anything here.

We thank you for this careful examination of the results. You are correct that in the previous version of **Figure 3** (attached below for reference), the Beta per SD approached ~0.2 (implying ~4% variance explained).

We wish to clarify that this high value was specific to the African (AFR) ancestry group. As detailed in **Original Supplementary Figure 2** (attached below for reference), this occurred because the randomly selected causal variants in our simulation happened to have higher cumulative allele frequencies (burden) in the AFR population compared to the European (EUR) population. Consequently, the realized heritability in the AFR subset was effectively higher than the global target parameter (1.25%), leading to higher Beta. In contrast, the Beta per SD for the EUR group in the original figure was approximately 0.1 (explaining ~1% variance), which aligned closely with the specified parameters.

Update with New Simulation Parameters: While this explains the discrepancy in the original draft, we have since recalibrated our simulation parameters to better align with recent empirical estimates of rare variant heritability. As detailed in our response to Q4, we have re-conducted the simulations with a more rigorous rare variant heritability (0.42%). In the revised **Figure 3** (now in the manuscript), the absolute Beta values have decreased to realistic levels across all ancestries. However, the relative trend (AFR > EUR) persists due to the population genetic architecture described above. We have highlighted this explanation in the Results section:

Results (Line 242, Simulation Study Results):

"Notably, RICE-RV showed particularly strong performance in AFR individuals, as the randomly selected causal variants and rare variant sets across the genome led to higher average burden scores (combined allele frequencies) in this ancestry (**Supplementary Figure 2**), driving more notable rare variant prediction performance."

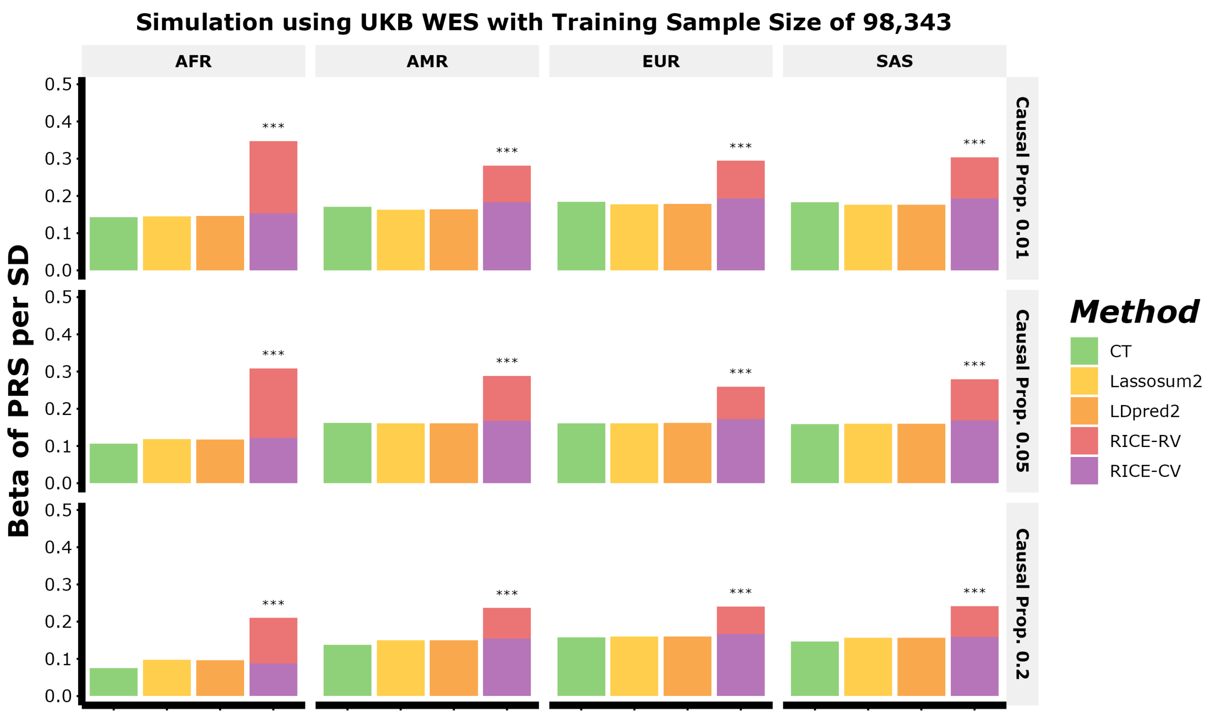

Original Figure 3. Simulation results comparing the predictive performance of PRSs for four ancestral groups from the UK Biobank (UKB). The training data (N = 98,343) included only individuals of European ancestry (EUR), while the tuning (N = 20,869) and validation datasets (N = 20,868) contained individuals of African (AFR), Admixed American or Latino (AMR), European (EUR), and South Asian (SAS) ancestries (**Supplementary Table 1**). Simulations assumed a common variant heritability of 0.05 and a rare variant set heritability of 0.0125, under the assumption of no negative selection effect size distribution (**Methods**). Causal proportions for both common variants and rare variant sets varied across three levels: 0.01 (top), 0.05 (middle), and 0.2 (bottom). All rare variants with causal rare variant sets were assumed to be causal. Data were generated using unrelated individuals from UK Biobank whole-exome sequencing data (WES), with simulation based on chromosome 22. PRS performance is reported as the "Beta of PRS per standard deviation (SD)", derived from the regression model $Y \sim PRS \times \beta$, with β representing the effect of standardized PRS on the standardized outcome (**Methods**). For RICE, the model used was $Y \sim PRS_{CV} \times \beta_{CV} + PRS_{RV} \times \beta_{RV}$. Significance was assessed using bootstrap confidence intervals and tested the alternative hypothesis $\beta_{RV} \neq 0$. *** denotes significance at the 99% level and ** denotes significance at the 95% level.

Original Supplementary Figure 2. Average heritability of rare variant burden scores for each ancestry by simulation design. Simulations assumed a common variant heritability of 0.05 and a rare variant set heritability of 0.0125. Causal proportions for both common variants and rare variant sets varied across three levels: 0.01 (top), 0.05 (middle), and 0.2 (bottom). Data was simulated under both strong negative selection (Scaled: Yes) and no negative selection (Scaled: No). Rare variants within causal rare variant sets were either all assigned as causal (Supp. Fig. 2a.) or a proportion of them were assigned as causal (proportion $\sim Uniform(0.2, 0.9)$, Supp. Fig. 2b.). Data were generated using unrelated individuals from UK Biobank whole-exome sequencing data (WES), with simulation based on chromosome 22.

a) Average heritability of rare variant burden scores for multiple simulation designs assuming all rare variants within causal rare variant sets are causal.

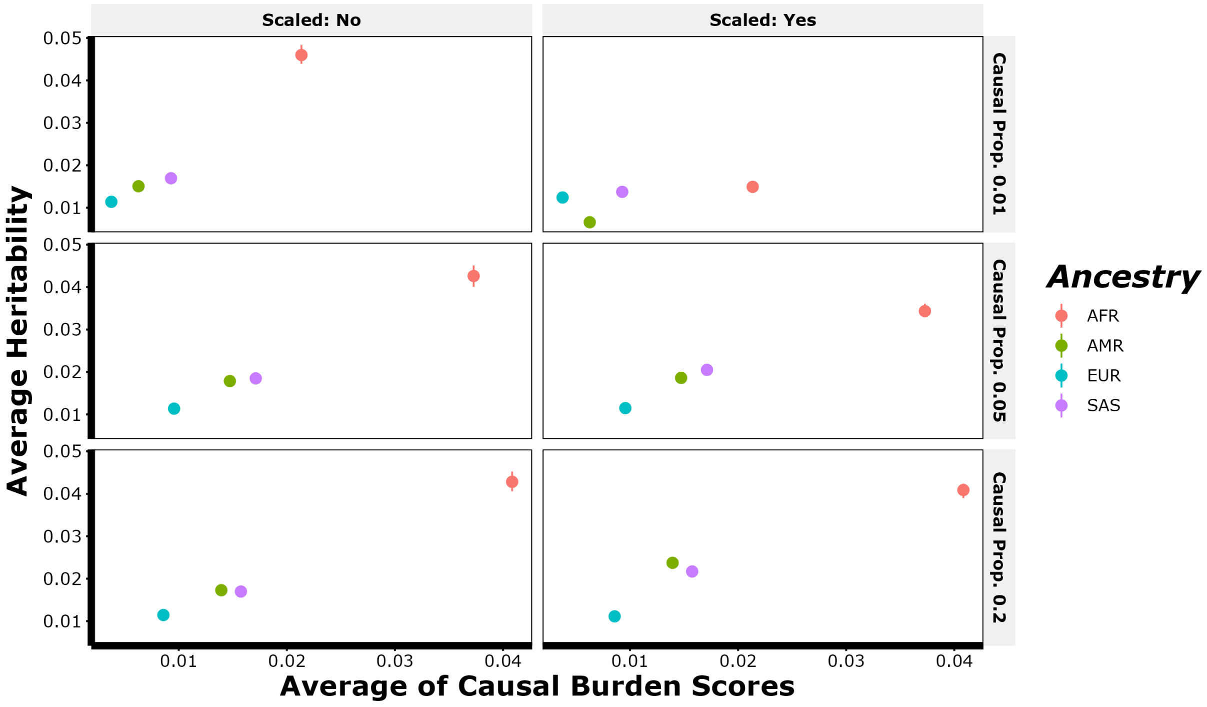

Original Supplementary Figure 2 continued: b) Average heritability of rare variant burden scores for multiple simulation designs assuming a proportion of rare variants within causal rare variant sets are causal.

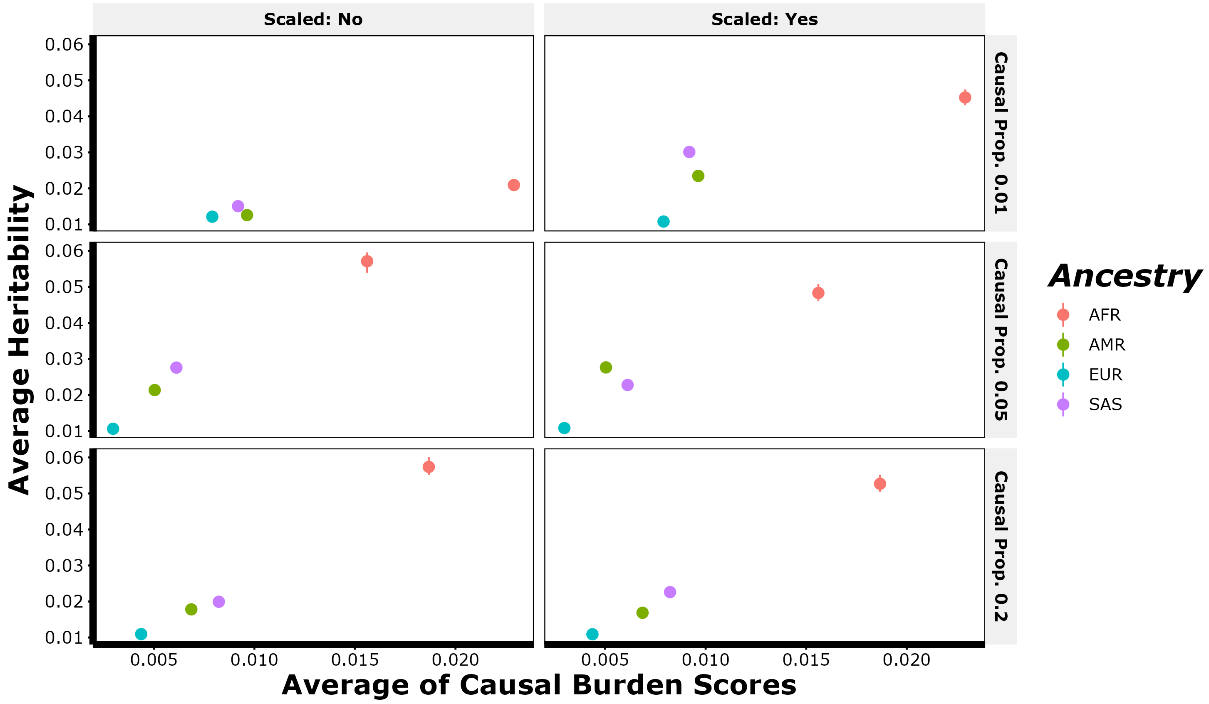

Q6. Statements such as RICE-RV increased R² by XXX% compared to RICE-CV are not particularly meaningful because the relative contributions of common and rare variants were predetermined and set by authors and were completely artificial.

We thank you and agree with your comment. In a simulation setting where the heritability parameters (h_{CV}^2 and h_{RV}^2) are fixed by the study design, the relative improvement in R^2 is indeed a function of those input parameters rather than an intrinsic measure of the method's utility. Accordingly, we have revised the Simulation Study Results section to remove these relative percentage comparisons.

Q7. I would suggest the authors take out the WES-only configuration for RICE-CV as it's not what is used in practice. This would simplify the manuscript.

We thank you for this very helpful suggestion. We agree that constructing a common variant PRS solely from WES data (the "WES-only" configuration) does not reflect standard practice, given that genome-wide array data or summary statistics are typically utilized. Accordingly, we have removed the WES-only RICE-CV configuration from the manuscript, figures, and supplementary materials, and revised the manuscript thoroughly.

Q8. The manuscript focuses primarily on lipid traits, for which rare variant PRS is known to have large contributions to the traits. However, for most other complex traits and diseases examined, rare variant PRS seems to contribute little or no predictive value. This limitation should be discussed more clearly, and the overall conclusions tuned down accordingly. It remains unclear whether rare variant PRS improves prediction beyond common variant PRS for complex traits or diseases in general, which depends on the genetic architecture of the traits and diseases.

We thank you for this insightful comment. We fully agree that the predictive utility of rare variants is not universal and is strictly governed by both the genetic architecture of the trait and the statistical power available to detect it. We have revised the manuscript to explicitly discuss these two distinct limiting factors:

Genetic Architecture (e.g., BMI): We acknowledge that for highly polygenic traits like BMI, the genetic architecture appears to be dominated by small-effect common variants. In these cases,

rare variants may not cluster in specific genes with sufficient effect sizes to provide additive predictive value beyond common variants, regardless of sample size.

Statistical Power (Binary Diseases): For binary traits/diseases, we emphasize that our findings are constrained by the study design. Since UK Biobank and All of Us are prospective population-based cohorts, the number of prevalent/incident cases for diseases is relatively small compared to quantitative trait sample sizes. Consequently, the study likely lacks sufficient statistical power to identify and weight rare variant burden signals effectively, unlike in lipid traits where the effective sample size is the full cohort.

Following your suggestion, we have tuned down our general conclusions in the Abstract and Introduction to reflect this heterogeneity. We also added a dedicated Discussion paragraph detailing why RICE-RV performance varies across trait types, explicitly noting the sample size bottleneck for binary diseases.

Abstract (Line 30)

"In real data analysis, RICE improved predictive accuracy compared to leading common variant methods for traits with distinct rare variant architectures, particularly lipids and height. For lipid traits, incorporating rare variants increased R^2 by up to ~11.2% in Europeans and ~60.7% in African ancestry compared to common variant PRS alone. Notably, for lipid traits, RICE captured substantial predictive signal beyond established high-penetrance genes, validating its ability to leverage the broader polygenic architecture of rare variation. While predictive gains were trait-dependent, reflecting differences in genetic architecture and statistical power, RICE provides a unified, genome-wide approach to maximize the utility of sequencing data for inclusive risk prediction."

Introduction (Line 109)

"In real data analyses, RICE improved predictive accuracy compared to top existing common variant PRS methods for traits with distinct rare variant architectures, particularly lipid traits and height, demonstrating the utility of a comprehensive common and rare variant PRS framework.**"**

Discussion (Line 450)

“Importantly, RICE-RV enhanced predictive power by incorporating rare variants, particularly improving risk prediction for lipid-related traits, while showing limited gains for BMI (**Figure 4**, **Figure 7**, **Supplementary Figures 7, 12, and 19**). These results highlight that the predictive value of rare variants is not universal but strongly dependent on both the underlying genetic architecture and available statistical power. For lipid traits, our findings align with previous heritability studies indicating that rare variants contribute more significantly to certain traits[37]. For instance, Weiner et al[37]. estimated that the ratio between exome burden heritability and common variant heritability was approximately 0.231 for LDL, but only 0.048 for BMI. In our models, this substantial improvement reflected RICE’s ability to capture distinct rare variant architectures, aggregating signals from both established high-impact genes (e.g., *APOB*, *LDLR*) and broader polygenic rare variation (**Supplementary Figure 10**). In contrast, the negligible improvements for BMI suggest that its genetic architecture is likely dominated by infinitesimal common variant effects, or that rare variant effects are too diffuse to be effectively captured by gene-centric burden testing. Furthermore, for binary traits or diseases, the limited gains should be interpreted in the context of statistical power. Unlike quantitative traits where the effective sample size includes the entire cohort, analyses in population-based cohorts like the UK Biobank are restricted to the subset of cases. The resulting lower case counts substantially reduce the power to accurately estimate rare variant weights, limiting the utility of RICE-RV in this specific study design.”

Q9. The newly added Figure 5 is helpful, but I would still like to see an analysis aligned with how PRS is used in practice, e.g., quantifying how many additional cases are identified or the improvement in OR when stratifying individuals by the integrated score compared with common variant PRS alone.

We appreciate your perspective on clinical implementation. Incorporating rare variant PRS for risk prediction is an emerging field, and the optimal way to integrate these signals into practice remains an area of active investigation.

In our manuscript, we initially employed a two-dimensional (2D) classification (stratifying by both RICE-CV and RICE-RV). This approach was inspired by current clinical protocols for high-penetrance genes, such as identifying *BRCA1/2* carriers independently of their common-variant polygenic background, to ensure that high-impact rare variant signals are not "diluted" by common variation.

However, we agree that a single integrated score may be more aligned with certain clinical workflows. To address this, we conducted additional analyses using a one-dimensional (1D) integrated RICE score (CV + RV) to identify high-risk individuals (top 10% quantile) for the six continuous traits (Figures attached) in the UKB imputed + WES analyses. We compared this to a traditional common-variant-only approach (RICE-CV top 10%):

1. Our analysis confirms that the integrated score identifies a distinct subset of high-phenotype individuals that common-variant PRS alone would miss. For example, in our HDL analysis, the integrated score identified 2.8% of individuals in the top phenotypic decile who were not flagged by the RICE-CV score alone. We observed similar net reclassification yields for other lipid traits, including LDL, log(TG), and TC, confirming the framework's consistent ability to capture extreme phenotypes driven by rare variants.
2. As illustrated in the provided dot plots, individuals identified as high-risk by the integrated score alone (Low CV / High Integrated Score) exhibit significantly higher mean trait values compared to baseline group ($P = 5.16 \times 10^{-19}$ for HDL). For these continuous traits, the observed standardized phenotypic shift serves as the quantitative analog to the improved odds ratios typically reported for binary disease outcomes.
3. By moving to an integrated framework, we improved the sensitivity of identifying high-phenotype individuals for HDL, log(TG), TC, and height compared to using common-variant PRS alone. For LDL, the integrated score captured a similar total number of individuals (1.2%), while for BMI, the number of identified high-phenotype individuals was nearly identical (a negligible 0.1% decrease). This consistency across diverse architectures reinforces our conclusion that RICE's added value is trait-dependent and specifically optimized for traits with meaningful rare-variant contributions.

We believe these results, along with the 2D stratification in **Figure 5** and **Supplementary Figure 9**, provide a comprehensive view of RICE's utility. While the 2D approach highlights the biological independence of rare signals, this 1D analysis demonstrates the “net reclassification” benefit relevant to clinical practice.

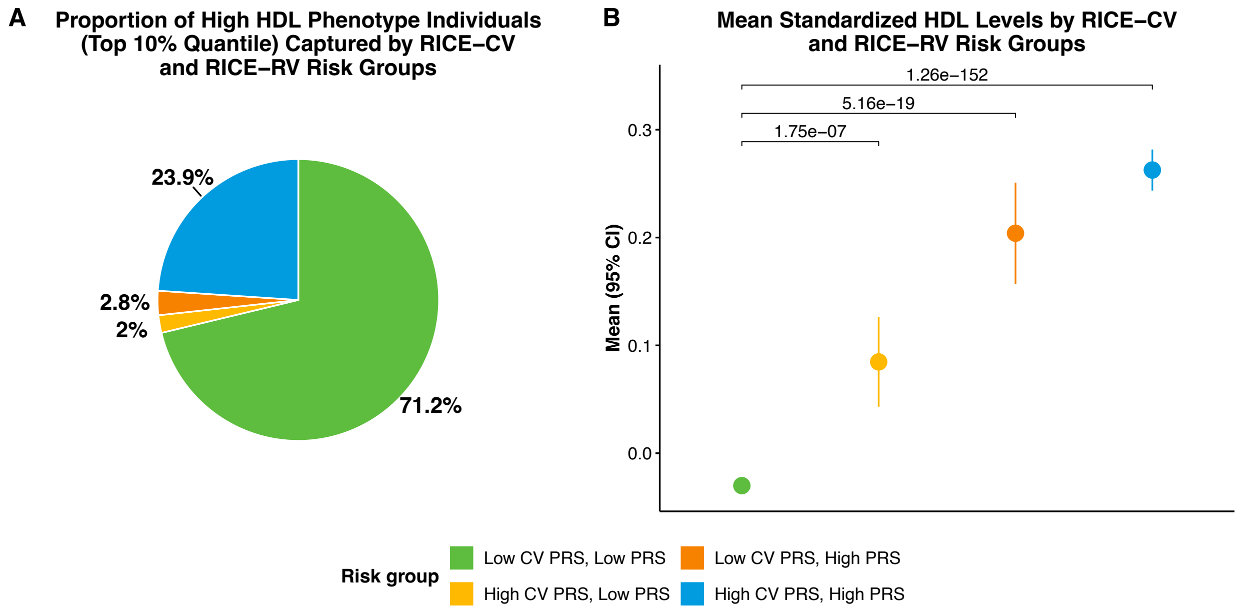

Response Figure for HDL. Comparison of risk stratification between common-variant PRS (RICE-CV) and the integrated RICE score for HDL. Analyses were conducted in the UKB imputed + WES validation dataset (N = 20,868). High-risk groups for both the common-variant PRS (RICE-CV) and the integrated framework (RICE) were defined using the top 10% quantile of their respective distributions. (A) Proportion of individuals with extreme phenotypes captured by integrated risk modeling. The pie chart illustrates the distribution of individuals within the top 10% of observed HDL values across four risk categories: (1) Low CV / Low RICE (identified as high-risk by neither method, baseline group) , (2) High CV / Low RICE (identified only by RICE-CV), (3) Low CV / High RICE (identified only by the integrated score) , and (4) High CV / High RICE (identified by both methods). (B) Phenotypic separation by risk group. Mean standardized outcome levels with 95% CI are shown for the four groups defined in (A). Pairwise p-values indicate the statistical significance of phenotypic differences between the stratified risk groups.

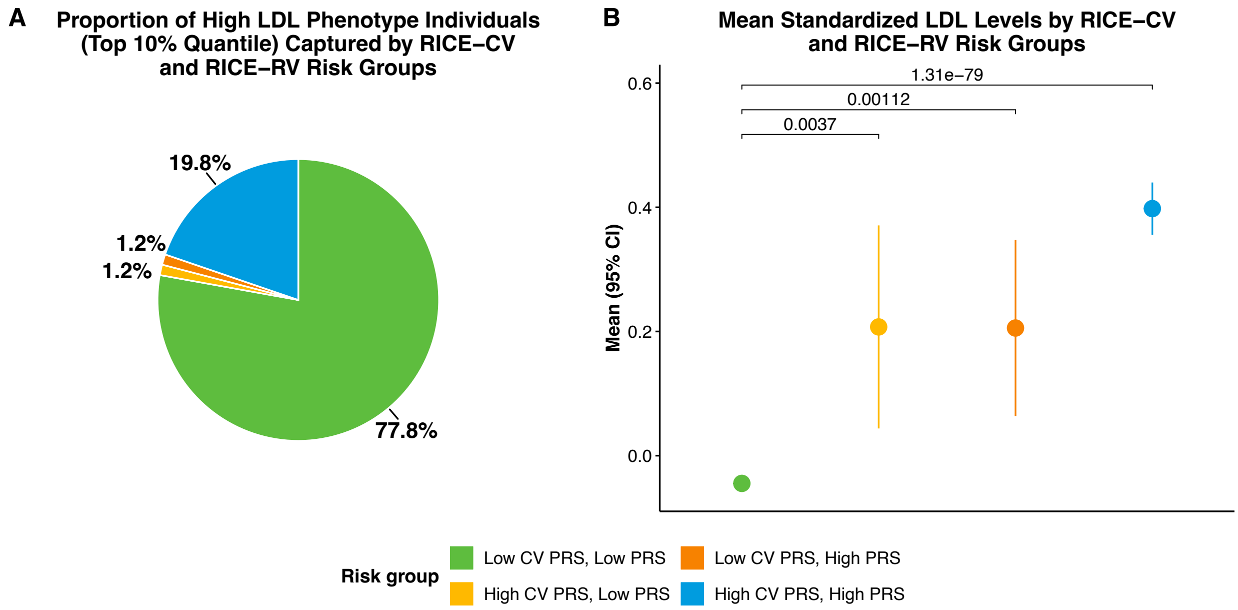

Response Figure for LDL. Comparison of risk stratification between common-variant PRS (RICE-CV) and the integrated RICE score for LDL. Analyses were conducted in the UKB imputed + WES validation dataset (N = 20,868). High-risk groups for both the common-variant PRS (RICE-CV) and the integrated framework (RICE) were defined using the top 10% quantile of their respective distributions. (A) Proportion of individuals with extreme phenotypes captured by integrated risk modeling. The pie chart illustrates the distribution of individuals within the top 10% of observed outcome values across four risk categories: (1) Low CV / Low RICE (identified as high-risk by neither method, baseline group) , (2) High CV / Low RICE (identified only by RICE-CV), (3) Low CV / High RICE (identified only by the integrated score) , and (4) High CV / High RICE (identified by both methods). (B) Phenotypic separation by risk group. Mean standardized outcome levels with 95% CI are shown for the four groups defined in (A). Pairwise p-values indicate the statistical significance of phenotypic differences between the stratified risk groups.

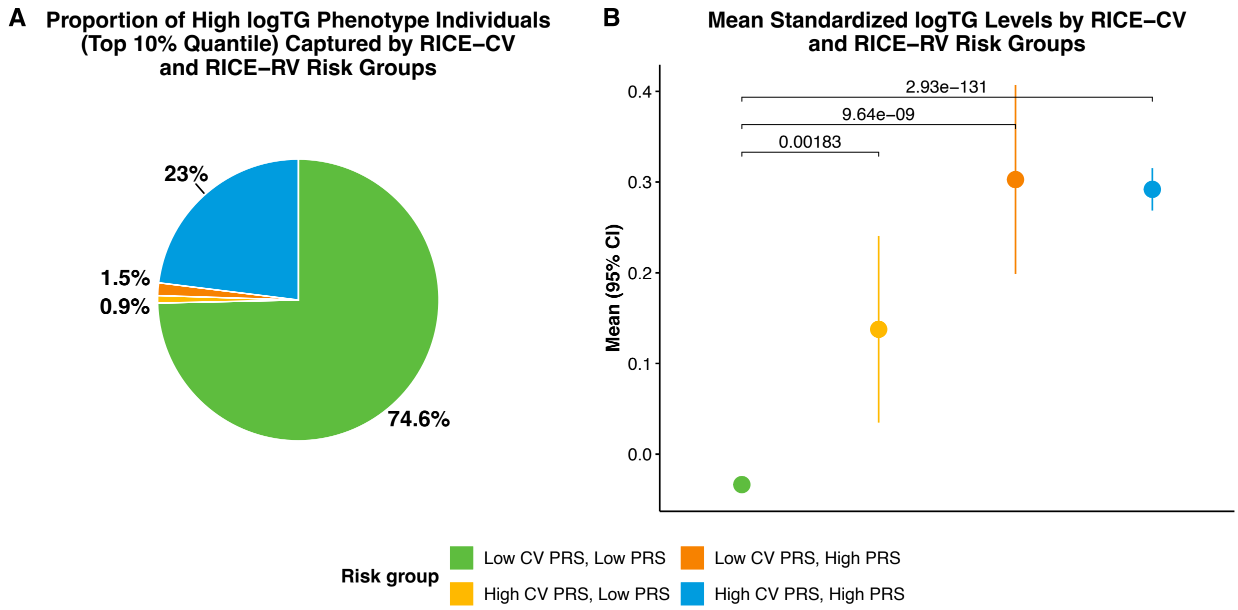

Response Figure for log(TG). Comparison of risk stratification between common-variant PRS (RICE-CV) and the integrated RICE score for log(TG). Analyses were conducted in the UKB imputed + WES validation dataset (N = 20,868). High-risk groups for both the common-variant PRS (RICE-CV) and the integrated framework (RICE) were defined using the top 10% quantile of their respective distributions. (A) Proportion of individuals with extreme phenotypes captured by integrated risk modeling. The pie chart illustrates the distribution of individuals within the top 10% of observed outcome values across four risk categories: (1) Low CV / Low RICE (identified as high-risk by neither method, baseline group) , (2) High CV / Low RICE (identified only by RICE-CV), (3) Low CV / High RICE (identified only by the integrated score) , and (4) High CV / High RICE (identified by both methods). (B) Phenotypic separation by risk group. Mean standardized outcome levels with 95% CI are shown for the four groups defined in (A). Pairwise p-values indicate the statistical significance of phenotypic differences between the stratified risk groups.

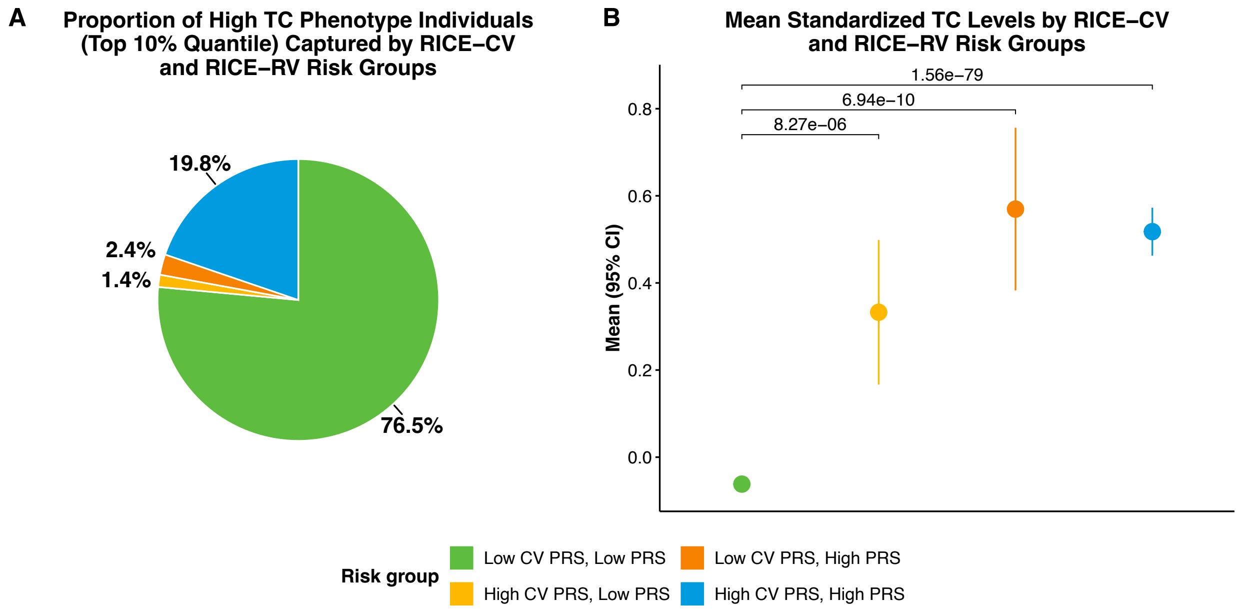

Response Figure for TC. Comparison of risk stratification between common-variant PRS (RICE-CV) and the integrated RICE score for TC. Analyses were conducted in the UKB imputed + WES validation dataset (N = 20,868). High-risk groups for both the common-variant PRS (RICE-CV) and the integrated framework (RICE) were defined using the top 10% quantile of their respective distributions. (A) Proportion of individuals with extreme phenotypes captured by integrated risk modeling. The pie chart illustrates the distribution of individuals within the top 10% of observed outcome values across four risk categories: (1) Low CV / Low RICE (identified as high-risk by neither method, baseline group) , (2) High CV / Low RICE (identified only by RICE-CV), (3) Low CV / High RICE (identified only by the integrated score) , and (4) High CV / High RICE (identified by both methods). (B) Phenotypic separation by risk group. Mean standardized outcome levels with 95% CI are shown for the four groups defined in (A). Pairwise p-values indicate the statistical significance of phenotypic differences between the stratified risk groups.

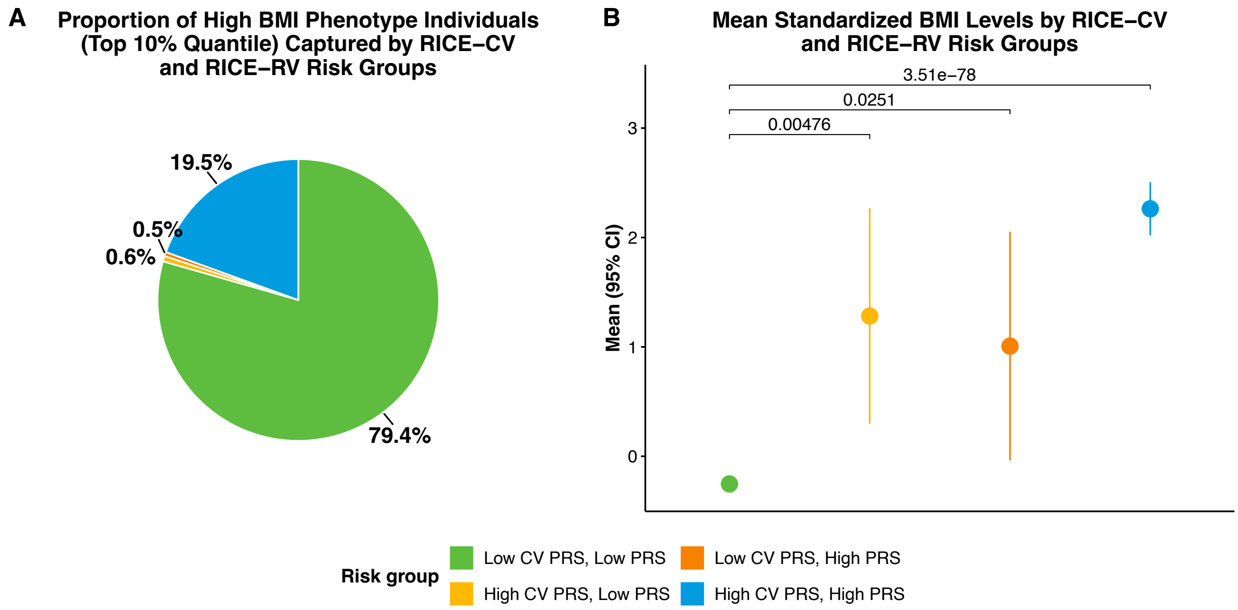

Response Figure for BMI. Comparison of risk stratification between common-variant PRS (RICE-CV) and the integrated RICE score for BMI. Analyses were conducted in the UKB imputed + WES validation dataset (N = 20,868). High-risk groups for both the common-variant PRS (RICE-CV) and the integrated framework (RICE) were defined using the top 10% quantile of their respective distributions. (A) Proportion of individuals with extreme phenotypes captured by integrated risk modeling. The pie chart illustrates the distribution of individuals within the top 10% of observed outcome values across four risk categories: (1) Low CV / Low RICE (identified as high-risk by neither method, baseline group) , (2) High CV / Low RICE (identified only by RICE-CV), (3) Low CV / High RICE (identified only by the integrated score) , and (4) High CV / High RICE (identified by both methods). (B) Phenotypic separation by risk group. Mean standardized HDL levels with 95% CI are shown for the four groups defined in (A). Individuals identified as high-risk by the integrated RICE score (including those missed by RICE-CV alone) exhibit significantly higher mean outcome levels compared to the baseline population. Pairwise p-values indicate the statistical significance of phenotypic differences between the stratified risk groups.

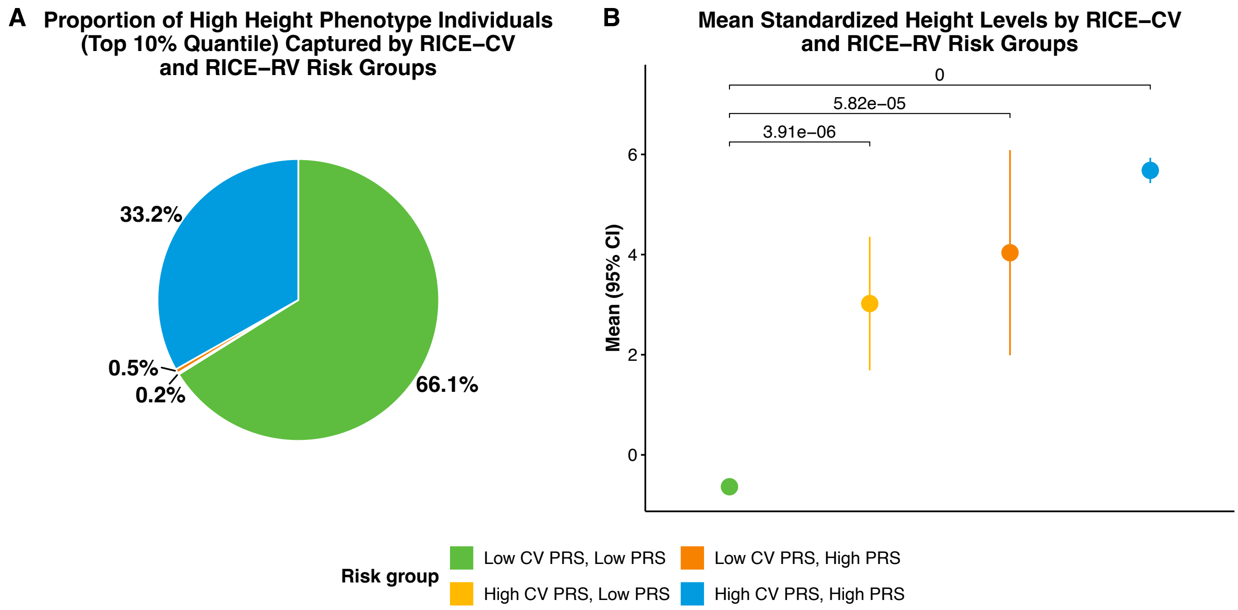

Response Figure for Height. Comparison of risk stratification between common-variant PRS (RICE-CV) and the integrated RICE score for height. Analyses were conducted in the UKB imputed + WES validation dataset (N = 20,868). High-risk groups for both the common-variant PRS (RICE-CV) and the integrated framework (RICE) were defined using the top 10% quantile of their respective distributions. (A) Proportion of individuals with extreme phenotypes captured by integrated risk modeling. The pie chart illustrates the distribution of individuals within the top 10% of observed outcome values across four risk categories: (1) Low CV / Low RICE (identified as high-risk by neither method, baseline group) , (2) High CV / Low RICE (identified only by RICE-CV), (3) Low CV / High RICE (identified only by the integrated score) , and (4) High CV / High RICE (identified by both methods). (B) Phenotypic separation by risk group. Mean standardized outcome levels with 95% CI are shown for the four groups defined in (A). Individuals identified as high-risk by the integrated RICE score (including those missed by RICE-CV alone) exhibit significantly higher mean HDL levels compared to the baseline population. Pairwise p-values indicate the statistical significance of phenotypic differences between the stratified risk groups.

Q10. Please clarify whether the cross-dataset analysis was limited to overlapping variants between UKB and AoU, or if it was performed on all available variants in AoU and then applied to those present in UKB. The latter would be a more realistic scenario, but either way this should be clarified.

We confirm that we followed the more realistic scenario as you described. The RICE models were trained using all available variants in the All of Us training dataset to maximize the information captured from the discovery cohort. When applying these pre-trained models to the UK Biobank (UKB), we utilized the subset of variants that were present in the UKB target dataset (imputed genotypes for common variants and WES for rare variants.

We have revised the Cross-Dataset Portability Analysis section in the Methods to clarify it:

Methods (Line 1388, Cross-Dataset Portability):

“RICE models were trained using all available variants in the AoU dataset. When applying these models to the UKB validation dataset, we utilized the subset of variants present in both datasets (details on variant counts in **Supplementary Table 9**).”

I was also asked to assess whether the authors have sufficiently addressed the concerns raised by Reviewer 2, who is no longer available.

We would like to extend a special thank you to you. In addition to providing your own insightful feedback, we deeply appreciate your time and effort in reviewing our responses to the unavailable Reviewer #2.

Q1. Reviewer 2 had raised a similar concern about the metric “Beta per SD of PRS.” Since the slope does not provide additional information beyond R2, I find the statements that this metric “emphasizes individual-level effects of rare variants” and offers “an individualized perspective” unconvincing.

We appreciate this critical feedback and the opportunity to clarify our terminology. We acknowledge that for a standardized PRS, the Beta coefficient is mathematically equivalent to the square root of R^2 and thus carries the same statistical information. However, we argue that they represent two different interpretive frameworks:

Population Perspective (R^2): This is a unitless ratio that quantifies what proportion of the total phenotypic variance in the population is explained by the PRS. For rare variants, R^2 is inherently constrained to be small because the variants themselves are infrequent across the whole population.

Individual/Clinical Perspective (Beta): This metric describes the magnitude of effect in the actual units of the trait. It answers the question: "If a specific individual moves one standard deviation up the polygenic risk distribution, how much is their predicted phenotype expected to change?".

When we refer to an “individualized perspective,” we are highlighting that while a rare variant PRS might explain very little population variance (low R^2), it can still represent a clinically significant phenotypic shift for the individuals who carry those variants. For a single patient, the "fraction of population variance" is less informative than the "expected change in my specific trait value."

Please see our detailed response to your Question 1, where we also discuss the consistency with binary trait metrics (AUC vs. OR) and include a new Discussion paragraph acknowledging limitation of "Beta per SD".

Q2. Reviewer 2 also raised a similar concern about whether cases with low CV PRS but high RV PRS can be effectively captured. As I mentioned, Figure 5 is informative in showing the distribution of risk profiles among individuals with extreme phenotypes, but it does not demonstrate how such individuals can be identified in practice by thresholding CV and RV PRS.

Thank you for the summary. As detailed in our response to your (Reviewer 1) Question 9, we have provided a new set of analyses for all six continuous traits to demonstrate exactly how these individuals are identified in practice using a unified integrated score.

To summarize: our manuscript maintains the two-dimensional (2D) classification in Figure 5 to emphasize the biological independence of rare and common variant signals. This approach is particularly relevant for identifying individuals where extreme rare-variant risk might be masked or "diluted" if only a single score were evaluated. However, we recognize that a one-dimensional (1D) integrated score may be more compatible with certain existing clinical decision-support systems that utilize a single risk threshold.

Our new analysis demonstrates that even within a 1D framework (using a top 10% threshold), RICE effectively "rescues" high-risk individuals missed by common-variant PRS alone. For example, in our HDL analysis, the integrated score correctly identified an additional 2.8% of individuals in the top phenotypic decile who were not flagged by the RICE-CV score alone. This confirms that RICE provides meaningful clinical utility regardless of whether a 2D or 1D implementation is selected.
